# Catalytic Enantioselective Synthesis of Oxazolines with Vinylogous Isocyano Esters

**DOI:** 10.1021/acs.orglett.5c02729

**Published:** 2025-08-25

**Authors:** Cristian Guzmán-Cedillo, Alicia Monleón-Ventura, Amparo Sanz-Marco, Carlos Vila, Gonzalo Blay

**Affiliations:** Departament de Química Orgànica, Facultat de Química, 16781 Universitat de València, C. Dr. Moliner 50, 46100 Burjassot, Spain

## Abstract

Vinylogous isocyano esters were prepared for the first time. They react with aldehydes to give chiral oxazolines bearing a pendant conjugated ester under synergistic silver/organocatalysis. The reaction is carried out using a bifunctional squaramide in combination with silver oxide and performs well with a number of aryl, heteroaryl, and cycloalkyl aldehydes, providing the expected heterocycles in good yields with good diastereoselectivity and enantiomeric excesses ranging from 60% to 95% ee.

## Graphical abstract

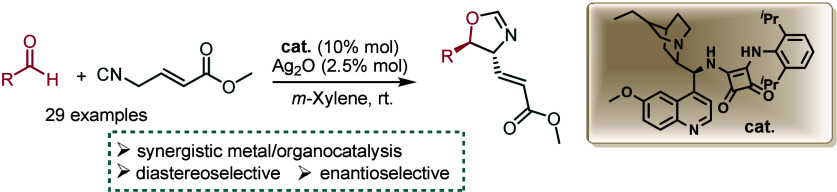

The term vinylogous refers to functional groups in which standard moieties are separated by one or more conjugated double bonds.^1^ This extended conjugation allows electronic information and functional group reactivity to be transmitted across the distances. This concept has enabled the creation of unique reactivity patterns that often parallel those of the parent functional group, allowing remote electrophilic^2^ or nucleophilic functionalization.^3^ Among vinylogous intermediates, vinylogous enolates and vinylogous anions that typically arise from deprotonation of α,β- or β,γ-unsaturated carbonyl groups exhibit extended nucleophilicity due to conjugation, enabling γ-selective addition to a broad range of electrophiles. More recently, the development of asymmetric catalytic systems that harness the reactivity of vinylogous anions has opened new avenues for enantioselective synthesis. In this regard, considerable development of the asymmetric vinylogous aldol reaction has occurred, mainly with enals and enones and, to lesser extent, with amides and esters.^4^ Focusing on the latter, examples include asymmetric catalytic reactions with butenolides,^5^ conjugated heterocycles^6^ and, less frequently, acyclic esters and amides (Scheme 1a).^7^

**Scheme 1:**
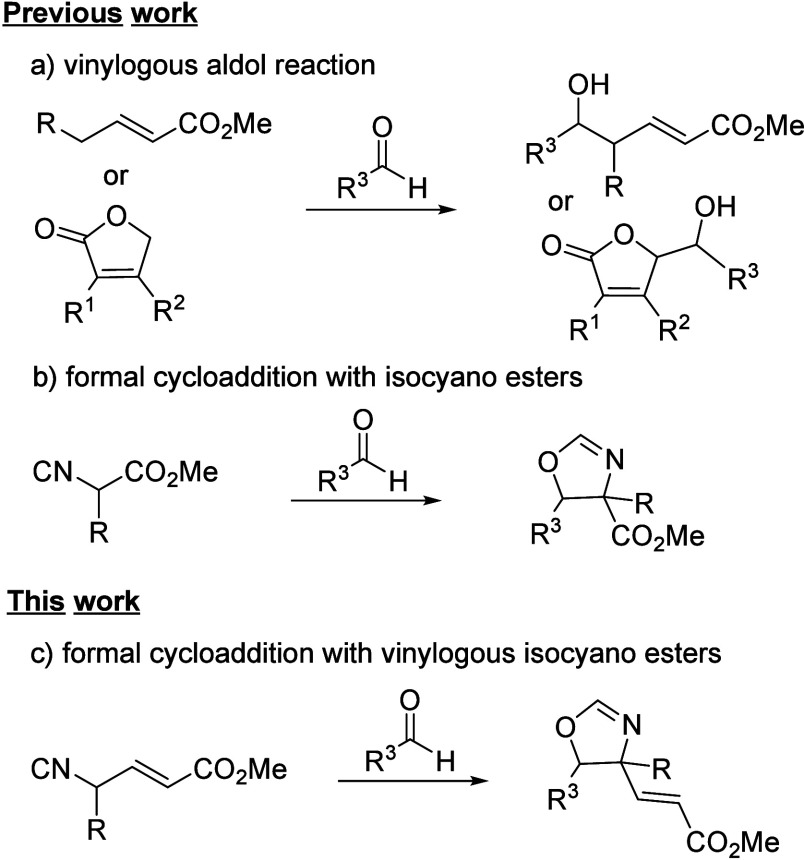


On the other hand, α-isocyanide carbanions have been employed as formal 1,3-dipoles in the synthesis of oxazolines with carbonyl compounds.^8^ The formal cycloaddition reaction involves the aldol reaction of the carbanion with a carbonyl compound, followed by cyclization. Unfunctionalyzed isocyanides require strong bases (LDA) to be deprotonated, which renders their application in asymmetric reactions troublesome. However, activated isocyanides such as α-isocyano esters are more prone to deprotonation and have been extensively used in catalytic asymmetric reactions with carbonyl and other electron-poor double bonds (Scheme 1b).^9,10^ Despite this, vinylogous versions of these reactions have not been described so far, to the best of our knowledge. As a part of our ongoing research, we have developed γ-isocyano unsaturated esters, and herein we describe their reaction with aldehydes to give chiral oxazolines with elongated substituents.

In the onset of our research, we studied the reaction of benzaldehyde (**1a**) and methyl 4-isocyanobut-2-enoate (**2a**). Isocyano ester **2a** was prepared from allylamine according to Scheme 2, which also represents the general strategy for the synthesis of the vinylogous isocyano esters used in this work.

**Scheme 2:**
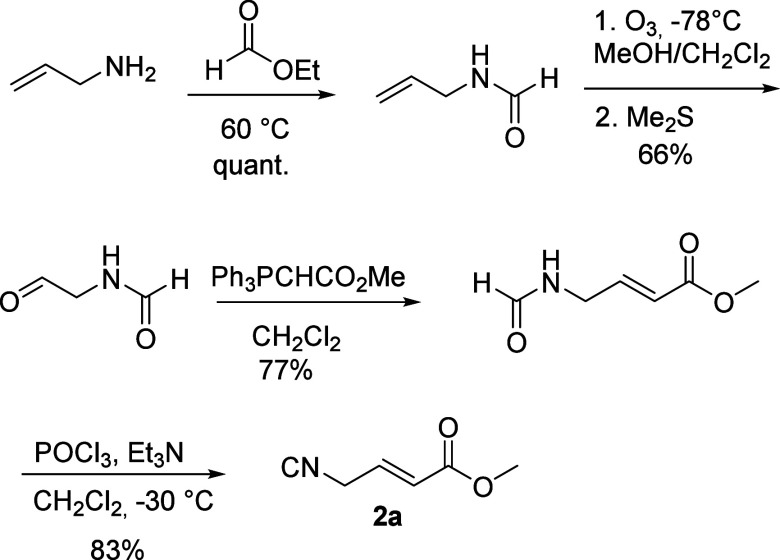
Synthesis of Vinylogous Isocyano Ester **2a**

The reaction of **1a** and **2a** was used as a test reaction for the optimization process. Representative results are outlined in Table 1 (see also SI for additional experiments). Bifunctional quinine-derived thiourea **I** and squaramide **II** (Figure 1) could not activate the reaction. Fortunately, upon the addition of silver oxide as an additive,^10e,10f^ the reaction was completed in less than 1 h (Table 1, entry 1 vs entry 2). Thiourea **I** provided the cycloaddition adduct **3aa** in almost racemic form, while squaramide **II** gave it in 67% yield and 38% ee. Rawal’s squaramide, Takemoto’s thiourea, and a cupreine ether were also tested, yielding the reaction product with inferior results (see SI). A number of squaramides derived from *Cinchona* alkaloids were tested. The best result was obtained with squaramide **VII**, which allowed compound **3aa** to be obtained in 71% yield and 78% ee as only one diastereomer. Next, we explored the effect of the solvents. The use of ethyl acetate or toluene increased both the yield and the enantioselectivity. On the other hand, ether-type solvents such as THF or MTBE gave high enantiomeric excesses but lower yields. The effect of temperature and concentration was evaluated in toluene. However, lowering the concentration had no effect on the outcome of the reaction, while decreasing the temperature to 0 °C reduced the yield without any positive effect on the enantioselectivity. The use of *m*-xylene permitted a slight increase in the ee up to 88%. Finally, a reduction in the amount of silver oxide from 5 to 2.5 mol % allowed us to obtain **3aa** in 74% yield and 90% ee. It is worth mentioning that compound **3aa** was obtained as a single diastereoisomer. The *trans* stereochemistry was assigned according to the coupling constant value (*J* = 8.1 Hz) observed in the ^1^H NMR spectrum.

**Figure 1:**
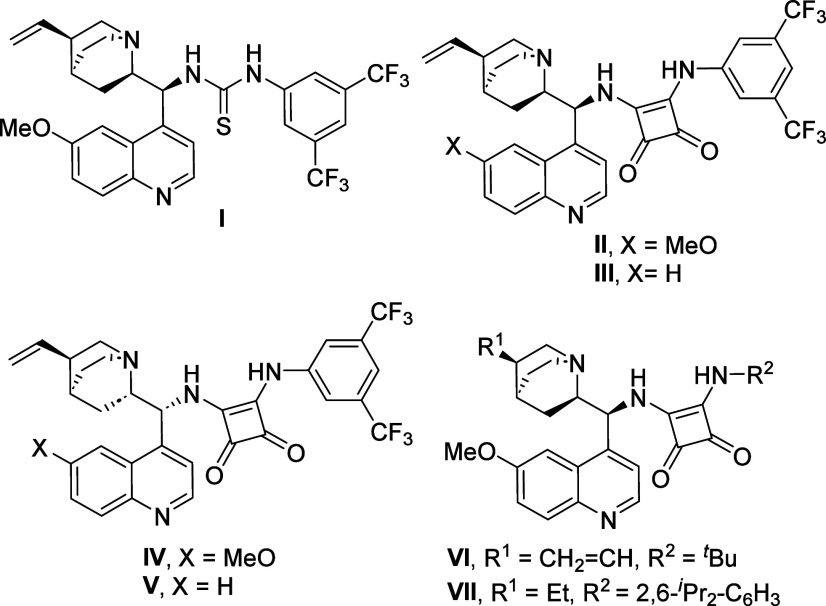
Some organocatalysts used in this study.

**Table 1:**
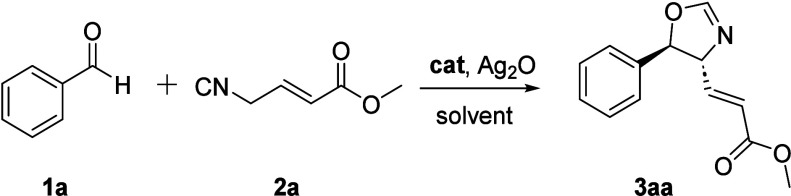
Enantioselective Reaction of Benzaldehyde **1a** with Isocyano Ester **2a**: Optimization Process^a^

entry	cat.	solvent	*t* (min)	yield^b^	ee^c^
1^d^	**I** or **II**	DCM	40	n.r.	
2	**I**	DCM	40	45	1
3	**II**	DCM	20	67	38
4	**III**	DCM	40	57	22
5	**IV**	DCM	20	59	–29
6	**V**	DCM	40	56	–34
7	**VI**	DCM	20	68	54
8	**VII**	DCM	20	71	78
9	**VII**	EtOAc	30	72	82
10	**VII**	THF	80	51	84
11	**VII**	MTBE	20	61	85
12	**VII**	toluene	20	70	86
13^e^	**VII**	toluene	90	67	86
14^f^	**VII**	toluene	20	70	86
14	**VII**	*o*-xylene	20	63	87
16	**VII**	*m*-xylene	20	70	88
17	**VII**	*p*-xylene	20	70	81
18^g^	**VII**	*m*-xylene	40	74	90

a Reaction conditions: **1a** (0.1 mmol), **2a** (0.12 mmol), **cat** (0.01 mmol), Ag_2_O (0.005 mmol), solvent (1 mL), rt.

b Yield of isolated product after column chromatography.

c Determined by chiral HPLC. Negative values indicate the opposite enantiomer.

d Reaction carried out in the absence of Ag_2_O.

e Reaction carried out at 0 °C.

f 3 mL of toluene was used.

g 0.0025 mmol Ag_2_O was used.

Under the best conditions available (Table 1, entry 18), we studied the scope of the reaction by initially focusing on the aldehyde (Scheme 3). Both electron-donating (**1b**, **1c**) and electron-withdrawing substituents (**1d**, **1e**) in the *para*-position of the aromatic ring of the aldehyde performed well in the reaction, thus yielding their respective products (**3ba**–**3ea**) in good yields, very good enantioselectivity, and excellent diastereoselectivity. It was found, however, that the presence of a strong electron-withdrawing nitro group led to a decrease in the enantioselectivity (**3fa**). Substituents in *meta*- and *ortho*-positions were well tolerated, generating the cycloaddition products **3ga**–**3ja** in good yields, high enantioselectivity, and excellent diastereoselectivity. This protocol could be extended to heteroaromatic aldehydes (**3ka**–**3ma**). A bulky naphthyl group in the aldehyde (**1n**) gave results similar to those obtained with phenyl derivatives.

**Scheme 3:**
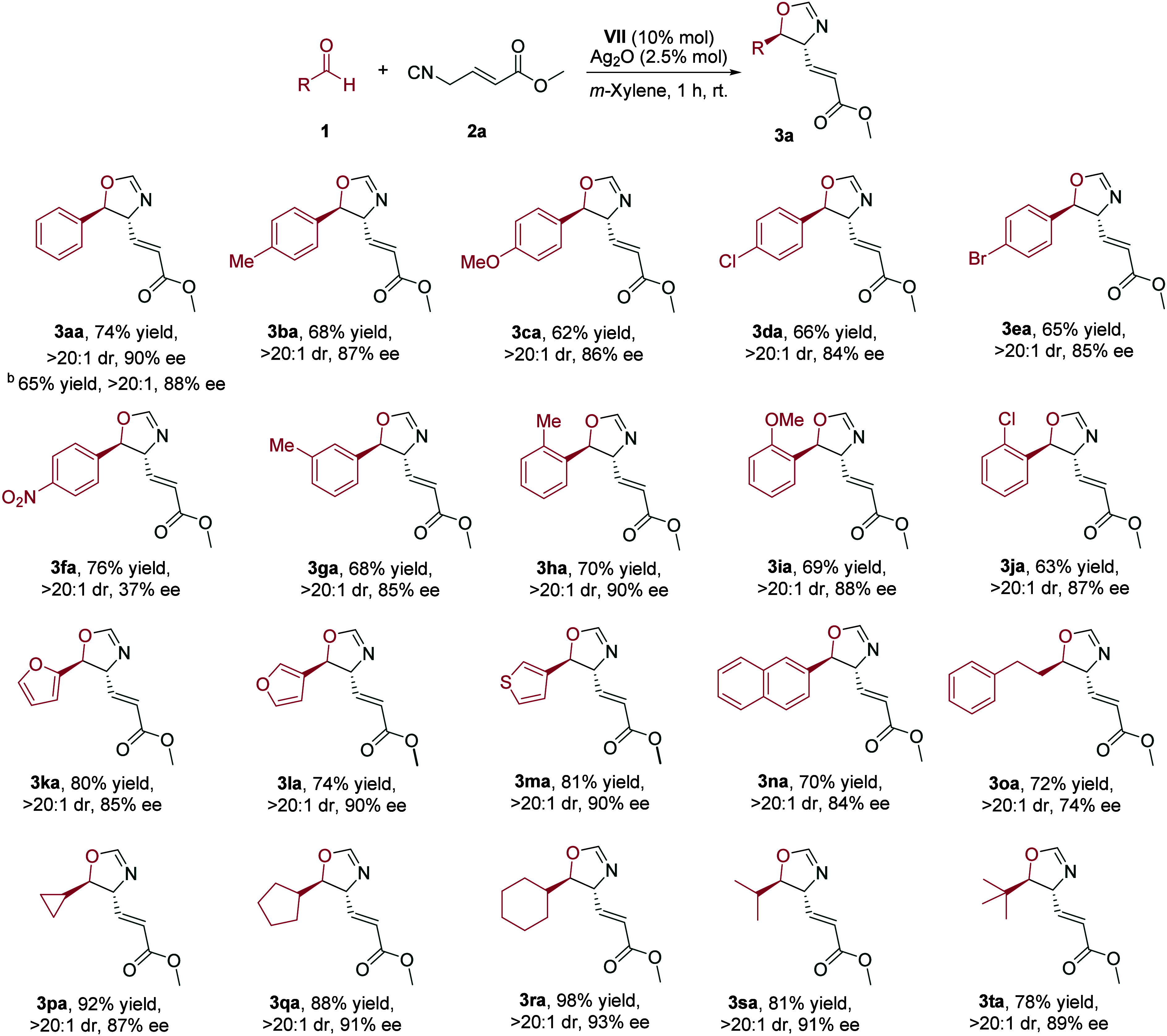
Scope of the Catalytic Enantioselective Reaction of Aldehydes **1** with Isocyano Ester **2a** ^a^

a Reaction conditions: **1** (0.1 mmol), **2a** (0.11 mmol), **VII** (0.01 mmol), Ag_2_O (0.005 mmol), *m*-xylene (1 mL); yield of isolated product after column chromatography; dr determined by ^1^H NMR; ee determined by chiral HPLC over chiral stationary phases.
b
**1a** (1.0 mmol), **2a** (1.1 mmol), **VII** (0.1 mmol), Ag_2_O (0.05 mmol), *m*-xylene (10 mL).

The utilization of dihydrocinnamaldehyde afforded the corresponding oxazoline **3oa** with similar results, albeit in lower enantiomeric excess. Notably, cyclic aliphatic aldehydes **1p**–**1r** led to excellent yields, diastereoselectivity, and enantioselectivity; in particular, the use of cyclohexanecarbaldehyde provided the expected product **3ra** in quantitative yield and 93% ee. Other acyclic branched aldehydes such as 2-methylpropanal and pivalaldehyde also reacted to give oxazolines **3sa** and **3ta** in good yields with 91% and 89% ee, respectively.

This reaction was amenable to 10-fold scale-up (1 mmol), exhibiting good yield and the same high level of diastereo- and enantioselectivity (Scheme 3, **3aa,** footnote b).

We then turned our attention to the vinylogous isocyano esters **2** (Scheme 4). The reaction was initially performed with ethyl 4-isocyanobut-2-enoate, bearing an ethyl instead of a methyl group (**2b**). It proceeded in high yield and excellent diastereoselectivity; however, the enantioselectivity was lower than that with **2a**. The utilization of methyl 4-isocyano-2-methylbut-2-enoate (**2c**) having a methyl on the α-carbon relative to the carbonyl group was evaluated in the cycloaddition with benzaldehyde (**1a**) and cyclohexanecarbaldehyde (**1r**). The corresponding oxazolines **3ac** and **3rc** were obtained in high to excellent yields, respectively, although with moderate enantiomeric excess. On the contrary, the utilization of the vinylogous isocyano ester containing a methyl group on the β-carbon (**2d**) led to the cycloaddition products **3ad** and **3rd** in high to excellent enantiomeric excesses. Interestingly, the reaction could also be performed with an α-substituted isocyanide, such as methyl 4-isocyanopent-3-enoate (**2e**). Although the diastereoselectivity was poor and the enantioselectivity was moderate, the employment of this isocyano ester **2e** led to the construction of oxazolines **3ae**, **3me**, and **3re** containing a quaternary stereocenter.

**Scheme 4:**
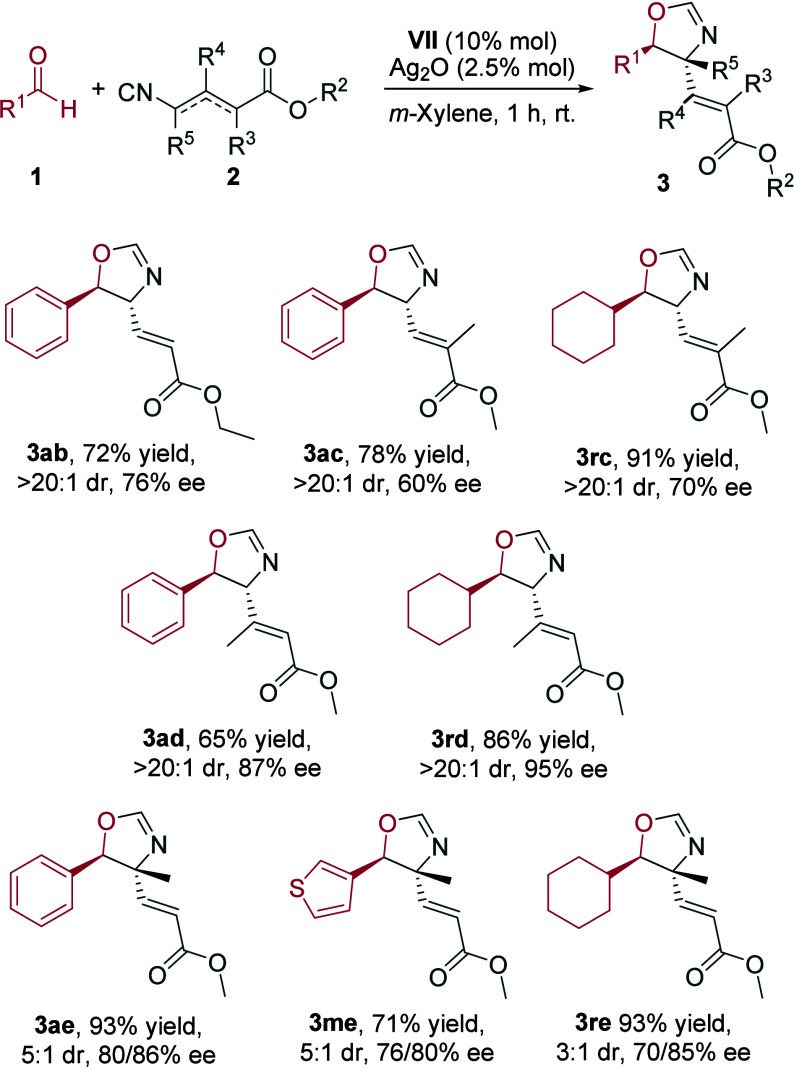
Scope of the Catalytic Enantioselective Reaction between Aldehydes **1** and Different Isocyano Esters **2** ^a^

a Reaction conditions: **1** (0.1 mmol), **2** (0.11 mmol), **VII** (0.01 mmol), Ag_2_O (0.005 mmol), *m*-xylene (1 mL); yield of isolated product after column chromatography; dr determined by ^1^H NMR; ee determined by chiral HPLC over chiral stationary phases.

As a control reaction to demonstrate the requirement of vinylogy for the formation of the α-isocyanide carbanion, the reaction between benzaldehyde (**1a**) and methyl 4-isocyanobutanoate (**2f**) was attempted (Scheme 5). As anticipated, no reaction was observed under the optimized conditions after 24 h, thus confirming the necessity of the conjugated double bond.

**Scheme 5:**
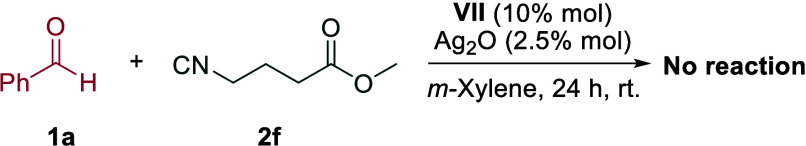
Formal Cycloaddition Attempt with Benzaldehyde (**1a**) and the Non-Vinylogous Isocyano Ester **2f**

Scheme 6 outlines some synthetic transformations of oxazoline **3aa**. The first reaction shows the selective reduction of the double bond to give oxazoline **4** with some deterioration of the enantiomeric excess. Next, we performed acid hydrolysis of the oxazoline ring in 2 M HCl/Et_2_O to afford the β-hydroxy formamide **5aa** in high yield, where no erosion of the ee was observed. Oxazoline **3ra** bearing a cyclohexyl substituent reacted similarly to give hydroxyformamide **5ra**. Treatment of compound **3aa** with 6 M aqueous HCl in methanol, which is usually used to hydrolyze the oxazoline to amino alcohol, led unexpectedly to the β-hydroxy ketone **6**. The absolute configuration of the stereogenic center in compound **6** was determined to be *R* by comparison with literature data.^11^ Assuming a retention of the configuration during the hydrolysis, the absolute configuration of that carbon in **3aa** was considered to be *R*. The configuration of the rest of compounds **3** was assigned presuming a uniform stereochemical mechanism.

**Scheme 6:**
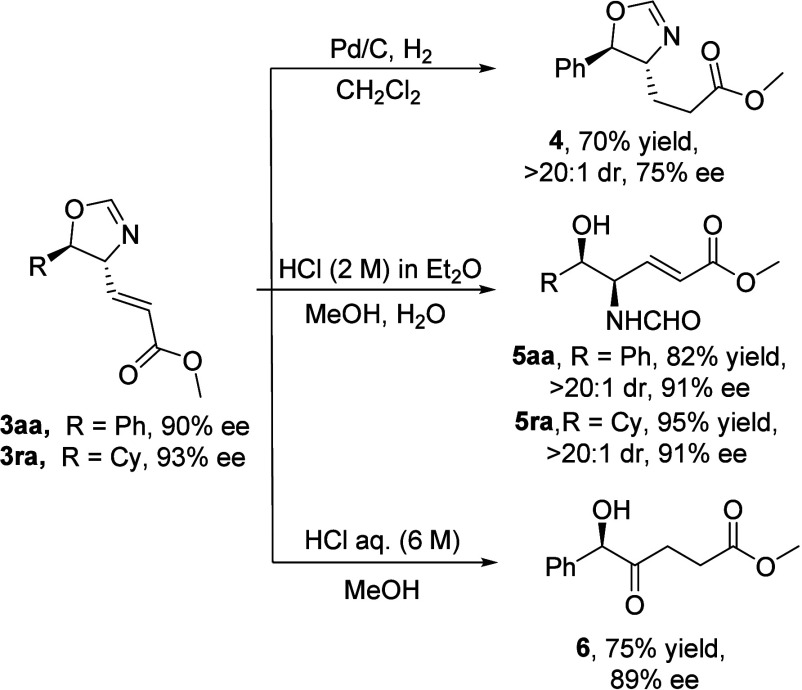
Synthetic Transformations of Compounds **3**

In conclusion, vinylogous isocyano esters have been prepared for the first time and successfully utilized in the enantioselective synthesis of chiral oxazolines bearing a pendent conjugated ester. The reaction, promoted by a synergistic silver/organocatalysis system combining a bifunctional squaramide and silver oxide, proceeds efficiently with a wide variety of aryl, heteroaryl, and cycloalkyl aldehydes. The methodology affords the desired heterocycles in good yields, with excellent diastereoselectivity and enantiomeric excesses ranging from 60% to 95% *ee*. The developed methodology enables the formation of quaternary stereogenic centers and is scalable. Moreover, useful synthetic transformations of the products were demonstrated. This approach opens new avenues for the asymmetric synthesis of functionalized heterocycles and provides a valuable tool for synthetic and medicinal organic chemistry. Further research with other electrophiles is underway in our laboratory (see SI).

## Supplementary Material



## Data Availability

The data underlying this study are available in the published article, in its Supporting Information, and openly available in DRYAD at https://doi.org/10.5061/dryad.w9ghx3g2t.

## Notes

Experimental procedures, characterization data, NMR spectra and HPLC traces (PDF)
