# Supplementary material for: Catalytic Enantioselective Synthesis of Oxazolines with Vinylogous Isocyano Esters
Source: Org Lett. 2025 Aug 25;27(35):9625–30. doi: 10.1021/acs.orglett.5c02729 (PMC12418506; doi:10.1021/acs.orglett.5c02729)
Supplement: Supplementary file 1 [file ol5c02729_si_001.pdf]

Catalytic enantioselective synthesis of oxazolines with vinylogous isocyano esters

Cristian Guzmán-Cedillo, Alicia Monleón-Ventura, Amparo Sanz-Marco, Carlos Vila, Gonzalo Blay*

Departament de Química Orgànica, Facultat de Química, Universitat de València, C. Dr. Moliner 50, 46100-Burjassot, Spain

Table of contents

General experimental methods……………………………………………………………S2

Synthesis and characterization data of isocyano esters **2**……………………….…………S2

Synthesis and characterization data of oxazolines **3**……………………………………….. S7

Synthesis of compound **3aa** at 1 mmol scale... S18

Synthetic transformations and characterization of compounds **4-6**……………………… S18

^{1}H and ^{13}C NMR spectra……………………………………………………………….....S21

HPLC traces for compounds **3** to **6**………..…………………………………………… S69

Optimization of the reaction conditions. Additional experiments……………………......S103

Synergistic action of silver and bifunctional squaramide (Scheme S1).........................S107

Unsuccesful transformation of compound **6** (Scheme S2)...S107

Reaction of acetophenone with compound **2a** under the reaction conditions................S107

References……………………………………………………………………………….....S108

General experimental methods

Unless it is stated otherwise, reactants were obtained from commercial sources and used directly without further purification. Reactions were monitored by TLC with Merck 60 F254 (reference 5554 Merck) silica gel plates. The eluted TLC plates were visualized under 254 nm UV light and revealed with stain solutions of cerium molybdate, potassium permanganate or *p*-anisaldehyde. Flash column chromatography was carried out using a Silica Gel Merck 60 stationary phase (reference 109385 Merck), with a 0.040- 0.063 mm particle range size. The eluent was made to flow through the column with an air pump. NMR spectra were recorded using a Bruker Avance III 300 or Bruker Neo500 at, respectively, 300 MHz, or 500 MHz for ^{1}H, or 75 MHz, or 125 MHz for ^{13}C. Residual non-deuterated solvent was used as internal standard (7.26 ppm for ^{1}H and 77.16 ppm for ^{13}C in $CDCl_3$). Chemical shifts (δ) are expressed in ppm and coupling constants (*J*), in Hz. HRMS were recorded using a Waters QTOF spectrometer equipped with an electrospray source with a capillary voltage of 3.3 kV (ESI). Specific optical rotations were measured using a Bellingham+Stanley ADP430 polarimeter equipped with a LED light source, measuring at the sodium wavelength (D line, 589 nm) and a 1 dm path length cuvette. Concentrations are expressed in g/100 mL. Enantiomeric excesses were measured through HPLC analysis, using a Hitachi Elite Lachrom chromatograph with a Hitachi L-4500 or L-2455U UV diode array detectors. Daicel or Phenomenex columns with chiral stationary phases were employed, and the samples were eluted with mixtures of HPLC-grade hexane and isopropyl alcohol. Catalyst **VII** was synthesized according to literature procedures.[1]

Synthesis and characterization data of isocyano esters 2

Synthesis of isocyano ester 2a

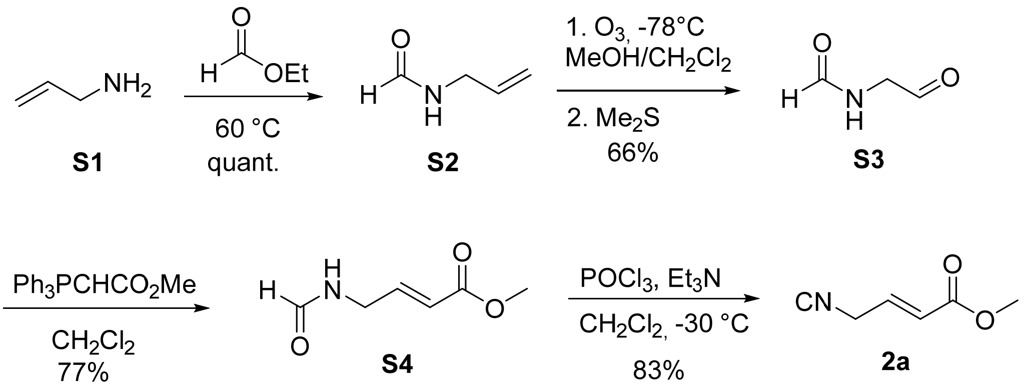

N-allylformamide (S2)[2]

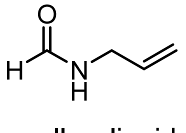

A solution of allyl amine (**S1**) (7.9 mL, 0.105 mol, 1 equiv.) in ethyl formate (42.5 mL, 0.526 mol, 5 equiv.) was stirred at reflux (oil bath) for 6 h. After that time, the mixture was concentrated to dryness at reduced pressure. The *N*-allylformamide (**S2**) (8.95 g, 0.105 mol, 100%) was obtained pure as a yellow liquid. **^{1}H NMR** (300 MHz, $CDCl_3$, 9:1 rotamer mixture): δ = 8.00 (s, 1H, CHO, major

rot.), 7.83 (d, *J* = 12 Hz, 1H, CHO, minor rot.), 7.22 (br s, 1H, NH major rot.), 6.77 (br s, 1H, NH minor rot.), 5.77 – 5.56 (m, 1H, CH, both rot.), 5.18 – 4.84 (m, 2H, CH_2, both rot.), 3.71 (m, 2H, CH_2-N, both rot.). **^{13}C NMR** (75 MHz, $CDCl_3$, major rotamer): δ = 161.5 (C=O), 133.4 (CH), 115.9 (CH_2), 40.1 (CH_2). **^{13}C NMR** (75 MHz, $CDCl_3$, minor rotamer): 164.9 (C=O), 134.2 (CH), 116.2 (CH_2), 43.6 (CH_2). Spectroscopic data matched with those reported in literature.[2]

Methyl (*E*)-4-amino-4-oxobut-2-enoate (S4)

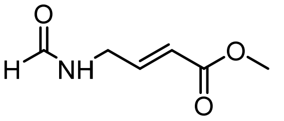

To a solution of *N*-allylformamide (**S2**) (3,0 g, 35.3 mmol, 1 equiv.) with an in-let adapter for ozonolysis in dry CH_2Cl_2 (45 mL) and CH_3OH (180 mL), a gentle stream of ozone is passed through and the flask is immediately cooled to −78°C. Ozonolysis is continued until the distinctive blue color of excess ozone is first observed. Ozonolysis is then terminated, and the excess ozone is removed by purging with a stream of nitrogen for 5-10 min. The solution is allowed to warm to room temperature, the ozonolysis adapter is replaced with a rubber septum, and neat dimethyl sulfide (13 mL, 176 mmol, 5 equiv.) is added via syringe. The solution is allowed to stir at room temperature for 24 h during which time the solution changes in color from pale yellow to dark red. The resulting solution is concentrated under reduced pressure. Then, the crude was chromatographed on silica gel eluting with CH_2Cl_2/MeOH (8:2) to give 2.43 g of the corresponding amino aldehyde **S3** which was used immediately in the next step. The amino aldehyde **S3** was dissolved in CH_2Cl_2 (150 mL) and methyl (triphenylphosphoranylidene)acetate (11.2 g, 33.4 mmol) were added. The mixture was stirred for 72 h, concentrated under reduced pressure and chromatographed on silica gel, eluting with CH_2Cl_2/MeOH (98:2 to 92:8) to give 3.04 g (77%) of methyl (*E*)-4-amino-4-oxobut-2-enoate (**S4**) as a mixture of rotamers and double bond isomers. **^{1}H NMR** (500 MHz, $CDCl_3$, major rotamer): δ = 8.20 (s, 1H, CHO), 6.85 (dt, *J* = 15.7, 5.1 Hz, 1H, CH), 6.59 (s, 1H, NH)), 5.91 (dt, *J* = 15.7, 1.9 Hz, 1H, CH), 4.03 (tdd, *J* = 5.2, 2.0, 0.6 Hz, 2H, CH_2), 3.69 (s, 3H CH_3). **^{13}C NMR** (125 MHz, $CDCl_3$): δ = 166.5 (C=O), 161.4 (C=O), 143.6 (CH), 121.6 (CH), 51.8 (CH_3), 38.7 (CH_2). **HRMS (ESI)** m/z 144.0652 $[M+H]^+$ $C_6H_{10}NO_3^+$ requires 144.0655.

Methyl (*E*)-4-isocyanobut-2-enoate (2a)

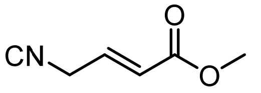

Methyl (*E*)-4-amino-4-oxobut-2-enoate (**S4**) (2.45 g, 17.2 mmol) was dissolved in dry CH_2Cl_2 (55 mL) and triethylamine (5.8 mL, 41.2 mmol, 2.4 equiv.) under nitrogen atmosphere. The solution was cooled to -30 °C and $POCl_3$ (1.8 mL, 18.9 mmol) was added dropwise. After 2 h, aqueous saturated Na_2CO_3 (50 mL) was added. The aqueous layer was extracted with CH_2Cl_2 (2x40 mL). The combined organic phases were washed with brine (2x40 mL) and dried over Na_2SO_4, filtered, and the solution was carefully concentrated under reduced pressure at 25 °C to avoid evaporation of isocyanide. Then, the product was purified by column chromatography eluting with pentane:diethyl ether mixtures to give 1.78 g (83%) of methyl (*E*)-4-isocyanobut-2-enoate (**2a**).[3] **^{1}H NMR** (300 MHz, $CDCl_3$): δ = 6.73 (dt, *J* = 13.2, 1.4 Hz, 1H, CH), 6.18 (dt, *J* = 13.2, 7.7 Hz, 1H, CH), 3.68 (s, 3H, CH_3), 3.18 (dd, *J* = 7.7, 1.4 Hz, 2H, CH_2). **^{13}C NMR** (75 MHz, $CDCl_3$): δ = 170.8 (C=O), 135.1 (CH), 127.9 (CH), 52.2 (CH_3), 35.4 (CH_2). **HRMS (ESI)** m/z 126.0542 $[M+H]^+$ $C_6H_8NO_2^+$ requires 126.0550.

Synthesis of isocyano ester 2b

The same procedure as described for the synthesis for methyl (*E*)-4-isocyanobut-2-enoate (**2a**) was used.

Ethyl (*E*)-4-formamidobut-2-enoate (S5)

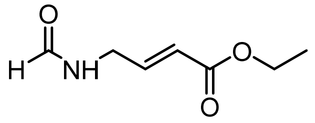

0.72 g (39%) of ethyl (*E*)-4-formamidobut-2-enoate (**S5**) were obtained from formylallyl amine (1,0 g, 11.8 mmol) as a 9:1 rotamer mixture. **^{1}H NMR** (500 MHz, $CDCl_3$, major rotamer): δ = 8.14 (s, 1H, CHO), 6.93 (s, 1H, NH), 6.80 (dt, *J* = 15.7, 5.0Hz, 1H, CH), 5.85 (dt, *J* = 15.7, 1.9 Hz, 1H, CH), 4.10 (q, *J* = 7.1 Hz, 2H, CH_2), 3.98 (dddd, *J* = 5.8, 5.0, 1.9, 0.7 Hz, 2H, CH_2), 1.20 (t, *J* = 7.1Hz, 3H, CH_3). **^{13}C NMR** (75 MHz, $CDCl_3$): δ = 166.0 (C=O), 161.6 (C=O), 143.4 (CH), 121.8 (CH), 60.6 (CH_2), 38.6 (CH_2), 14.1 (CH_3). **HRMS (ESI)** m/z 158.0807 $[M+H]^+$ $C_7H_{12}NO_3^+$ requires 158.0812.

Ethyl (*E*)-4-isocyanobut-3-enoate (2b)

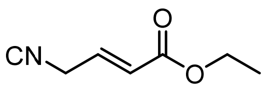

0.57 g (92%) of ethyl (*E*)-4-isocyanobut-3-enoate (**2b**) was obtained from 0.7 g of ethyl (*E*)-4-formamidobut-2-enoate, after column chromatography eluting with pentane:diethyl ether (8:2 to 7:3). **^{1}H NMR** (300 MHz, $CDCl_3$): δ = 6.85 – 6.78 (m, 1H, CH), 6.23 (dt, *J* = 13.2, 7.6 Hz, 1H, CH), 4.25 (d, *J* = 7.2 Hz, 2H, CH_2), 4.21 (d, *J* = 7.1 Hz, 2H, CH_2), 1.31 (t, *J* = 7.1 Hz, 3H, CH_3). **^{13}C NMR** (75 MHz, $CDCl_3$): δ = 165.3 (C=O), 136.7 (CH), 123.9 (CH), 61.1 (CH_2), 42.6 (t, *J* = 7.5 Hz, CH_2), 14.3 (CH_3). **HRMS (ESI)** m/z 140.0697 $[M+H]^+$ $C_7H_{10}NO_2^+$ requires 140.0706.

Synthesis of isocyano ester 2c

The same procedure as described for the synthesis for methyl (*E*)-4-isocyanobut-2-enoate (**2a**) was used.

Methyl (*E*)-4-formamido-2-methylbut-2-enoate (S6)

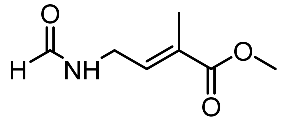

0.67 g (36%) of methyl (*E*)-4-formamido-2-methylbut-2-enoate (**S6**) were obtained from formylallyl amine (1,0 g, 11.8 mmol) as a 9.2:0.8 rotamer mixture. **^{1}H NMR** (300 MHz, $CDCl_3$, major rotamer): δ = 8.07 (s, 1H, CHO), 7.09 (s, 1H, NH), 6.54 (tt, *J* = 4.9, 2.6 Hz, 1H, CH), 3.93 (tt, *J* = 4.6, 2.4 Hz, 2H, CH_2), 3.62 (s, 3H, CH_3), 1.76 (s, 3H, CH_3). **^{13}C NMR** (75 MHz, $CDCl_3$, major rotamer): δ = 167.9 (C=O), 161.5 (C=O), 137.2 (CH), 129.6 (C), 51.8 (CH_3), 36.2 (CH_2), 12.5 (CH_3). **HRMS (ESI)** m/z 158.0807 $[M+H]^+$ $C_7H_{12}NO_3^+$ requires 158.0812.

methyl (E)-4-isocyano-2-methylbut-2-enoate **(2c)**

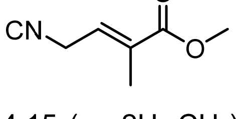

0.57 g (92%) of methyl (*E*)-4-isocyano-2-methylbut-2-enoate (**2c**) were obtained from 0.67 g of methyl (*E*)-4-formamido-2-methylbut-2-enoate, after column chromatography eluting with pentane:diethyl ether (8:2 to 7:3).**^{1}H NMR** (300 MHz, $CDCl_3$): δ = 6.72 - 6.66 (m, 1H, CH), 4.18 – 4.15 (m, 2H, CH_2), 3.76 (s, 3H, CH_3), 1.85 (q, *J* =1.2 Hz, 3H, CH_3). **^{13}C NMR** (75 MHz, $CDCl_3$): δ = 167.1 (C=O), 157.7 (CN), 132.1 (C), 131.5 (CH), 52.3 (CH_3), 39.6 (CH_2), 12.8 (CH_3). **HRMS (ESI)** m/z 113.0593 [M-CN] $C_6H_8O_2$ requires 113.0597.

Synthesis of isocyano ester 2d

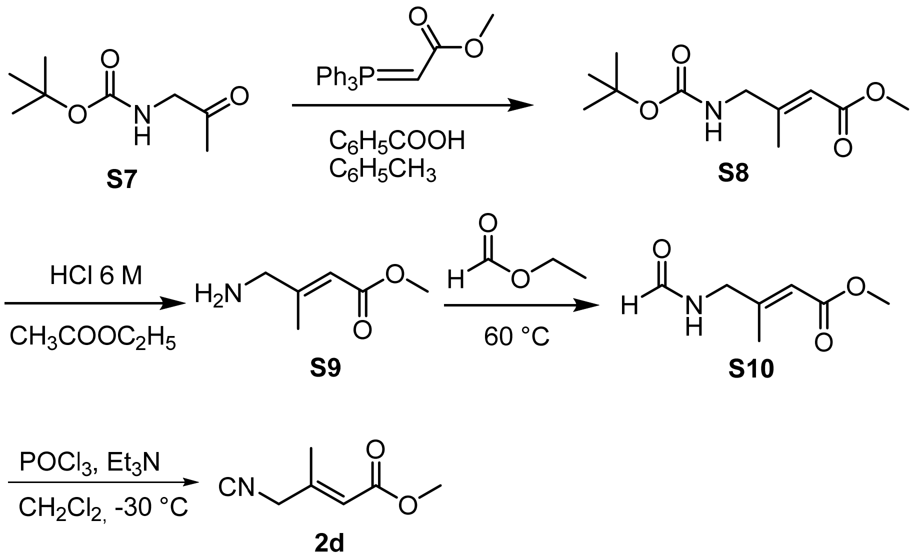

Methyl (*E*)-4-((*tert*-butoxycarbonyl)amino)-3-methylbut-2-enoate (S8)

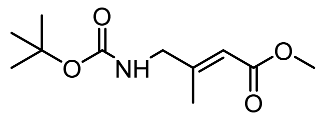

To a solution of *tert*-butyl (2-oxopropyl)carbamate (**S7**) (1,0 g, 5.76 mmol, 1 equiv.) in toluene (15 mL), under nitrogen atmosphere, methyl (triphenyl phosphoranilidene) acetate (2.90 g, 8.66 mmol, 1.5 equiv.) and benzoic acid (70 mg, 0.56 mmol) were added. The mixture was heated to 80 °C (oil bath) for 24 h. The solvent was evaporated under reduced pressure and the product was purified by column chromatography, eluting with hexane:EtOAc mixtures, to give methyl (*E*)-4-((*tert*-butoxycarbonyl)amino)-3-methylbut-2-enoate (**S8**) (0.80 g, 3.51 mmol, 61%). **^{1}H NMR** (300 MHz, $CDCl_3$): δ = 5.63 (s, 1H, CH), 5.31 (s, 1H, NH), 3.60 (s, 2H, CH_2), 3.53 – 3.50 (m, 3H, CH_3), 1.96 (d, *J* = 5.0 Hz, 3H, CH_3), 1.29 (d, *J* = 3.1 Hz, 9H, $3CH_3$). **^{13}C NMR** (75 MHz, $CDCl_3$): δ = 166.8 (C=O), 156.1 (C=O), 155.8 (C), 113.8 (CH), 79.3 (C), 50.7 (CH_3), 47.4 (CH_2), 28.1 ($3CH_3$), 16.4 (CH_3). **HRMS (ESI)** m/z 230.1378 $[M+H]^+$ $C_{11}H_{20}NO_4^+$ requires 230,1387.

Methyl (*E*)-4-formamido-3-methylbut-2-enoate (S10)

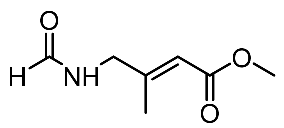

To a solution of methyl (*E*)-4-((tert-butoxycarbonyl)amino)-3-methylbut-2-enoate (**S8**) (0.8047 g, 3.51 mmol) in EtOAc, HCl 6 M (5.6 mL) were added. The mixture was stirred to room temperature for 6 h. After that time, the mixture was basified with saturated aqueous $NaHCO_3$ (1 mL), water was added (5 mL), phases were separated with EtOAc (3×10 mL), washed with brine (10 mL) and dried over $MgSO_4$. The crude was dissolved in ethyl formate (1.42 mL, 17.55 mmol, 5 equiv.) and stirred at reflux (oil bath) for 6 h. After that time, the mixture was concentrated to dryness at reduced pressure to give methyl (*E*)-4-formamido-3-methylbut-2-enoate (**S10**) (0.4967 g, 3.16 mmol, 90%) as a 8.5:1.5 rotamer mixture. Yellow oil**.** **^{1}H NMR** (300 MHz, $CDCl_3$, major rotamer): δ = 8.29 (d, *J* = 1.5 Hz, 1H, CHO), 5.83 (m, 1H, NH), 5.77 (q, *J* = 1.4Hz, 1H, CH), 3.97 (ddt, *J* = 6.4, 1.4, 0.7 Hz, 2H, CH_2), 3.7 (s, 3H, CH_3), 2.16-2.14 (m, 3H, CH_3).**^{13}C NMR** (75 MHz, $CDCl_3$, major rotamer): δ

= 166.8 (C=O), 161.2 (C=O), 154.2 (C), 115.3 (CH), 51.3 (CH_3), 45.0 (CH_2), 17.0 (CH_3). **HRMS (ESI)** m/z 158.0806 [M+H] $^+$ $C_7H_{12}NO_3^+$ requires 158.0812.

Methyl (*E*)-4-isocyano-3-methylbut-2-enoate (2d)

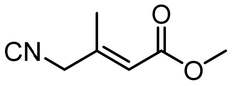

Following the general procedure for the dehydration of formamides, 0.249 g (70%) of methyl (*E*)-4-isocyano-3-methylbut-2-enoate (**2d**) were obtained from 0.401 g of methyl (*E*)-4-formamido-3-methylbut-2-enoate. Pale yellow oil. ^{1}**H NMR** (300 MHz, $CDCl_3$): δ = 5.70 (s, 1H, CH), 3.71 (s, 3H, CH_3), 3.31 (d, *J* = 0.8 Hz, 2H, CH_2), 1.84 (d, *J* = 1.7 Hz, 3H, CH_3). ^{13}**C NMR** (75 MHz, $CDCl_3$): δ = 169.5 (C=O), 165.2 (CN), 138.6 (C), 111.0 (CH), 52.3 (CH_3), 37.7 (CH_2), 20.0 (CH_3). **HRMS (ESI)** m/z 140.0709 $[M+H]^+$ $C_7H_{10}NO_2^+$ requires 140.0706.

Synthesis of isocyano ester 2e

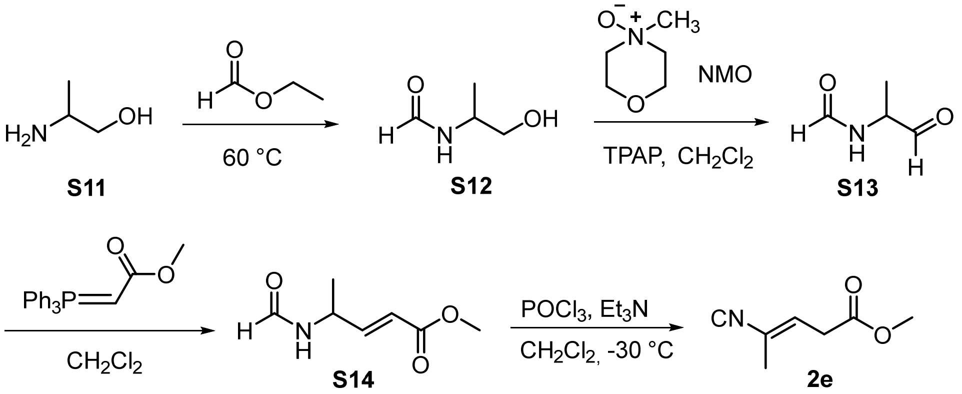

***N*-(1-hydroxypropan-2-yl)formamide (S12)**

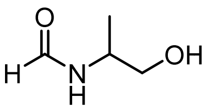

Following the procedure described for the synthesis of *N*-allylamine, 13.71 g (100%) of *N*-(1-hydroxypropan-2-yl)formamide (**S12**) were obtained from 10.6 mL (0.133 mol) of DL-Alaninol and 49.3 mL (0.665 mol) of ethyl formate as a 7.8:2.2 rotamer mixture. Yellow oil. ^{1}**H NMR** (300 MHz, $CDCl_3$, major rotamer): δ = 8.01 (d, *J* = 1.2 Hz, 1H, CHO), 7.04 (d, *J* = 7.5 Hz, 1H, NH), 4.48 (s, 1H, OH), 4.09 – 3.94 (m, 1H, CH), 3.55 – 3.38 (m, 2H, CH_2), 1.15 – 1.06 (dd, *J* = 8.3, 6.7Hz, 3H, CH_3). ^{13}**C NMR** (75 MHz, $CDCl_3$, major rotamer): δ = 162.1 (C=O), 65.5 (CH_2), 46.3 (CH), 16.8 (CH_3). **HRMS (ESI)** m/z 104.0702 $[M+H]^+$ $C_4H_{10}NO_2^+$ requires 104.0706.

Methyl (*E*)-4-formamidopent-2-enoate (S14)

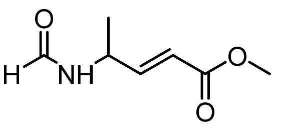

N-(1-hydroxypropan-2-yl)formamide (**S12**) (2.00 g, 19.4 mmol), NMO (4.55 g, 38.80 mmol) and 4 Å molecular sieves (6.00 g) were dissolved in CH_2Cl_2 (120 mL). After 5 min, TPAP (0.68 g, 1.94 mmol, 0.1 equiv.) was added and the solution was allowed to stir at room temperature for 4 h. The resulting product was concentrated under reduced pressure. Then, the crude was dissolved in CH_2Cl_2 (90 mL) under nitrogen atmosphere and methyl 2-(triphenyl-λ5-phosphaneylidene) acetate (7.7838 g, 23.28 mmol) was added. The mixture was allowed to stir at room temperature for 72 h. The solvent was evaporated under reduced

pressure and was purified by column chromatography, eluting with CH_2Cl_2: MeOH mixtures to give 0.2295 g (8%) of methyl (*E*)-4-formamidopent-2-enoate (**S14**) as an unstable mixture of rotamers and doble bond isomers. **^{1}H NMR** (300 MHz, $CDCl_3$, signals for the title compound): δ = 8.17 (t, *J* = 1.3 Hz, 1H, CHO), 6.86 (dd, *J* = 15.7, 5.1 Hz, 1H, CH), 6.11 (d, *J* = 8.4 Hz, 1H, NH), 5.91 (dd, *J* = 15.8, 1.7 Hz, 1H, CH), 4.82-4.75 (m, 1H, CH), 3.70 (s, 3H, CH_3), 1.31 (d, *J* = 7.0 Hz, 3H, CH_3). **^{13}C NMR** (75 MHz, $CDCl_3$, signals for the title compound): δ = 166.8 (C=O), 160.7 (C=O), 148.2 (CH), 120.6 (CH), 51.9 (CH_3), 51.5 (C), 19.9 (CH_3). **HRMS (ESI)** m/z 158.0807 [M+H] $^+$ $C_7H_{12}NO_3^+$ requires 158.0812.

Methyl (*E*)-4-isocyanopent-3-enoate (2e)

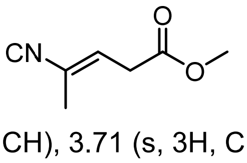

Following the general procedure for the dehydration of formamides, 0.0378 g (18%) of methyl (*E*)-4-isocyanopent-3-enoate (**2e**) were obtained from 0.2295 g (1.46 mmol) of methyl (*E*)-4-formamidopent-2-enoate**.** Yellow oil.**^{1}H NMR** (300 MHz, $CDCl_3$): δ = 6.00 – 5.93 (m, 1H, CH), 3.71 (s, 3H, CH_3), 3.10 (dd, *J* = 7.6, 1.1 Hz, 2H, CH_2), 1.93 (s, 3H, CH_3). **^{13}C NMR** (75 MHz, $CDCl_3$): δ = 170.3 (C=O), 161.1 (CN), 124.8 (C), 123.9 (CH), 52.4 (CH_3), 32.7 (CH_2), 17.2 (CH_3). **HRMS (ESI)** m/z 140.0703 [M+H]$^+$ $C_7H_{10}NO_2^+$ requires 140.0706.

Synthesis of methyl 4-isocyanobutanoate (2f)

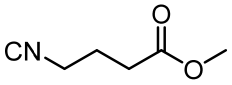

Following the general procedure for the dehydration of formamides, 0.1525 g (87%) of methyl 4-isocyanobutanoate (**2f**) were obtained from 0.2003 g (1.38 mmol) of methyl 4-formamidobutanoate. Pale yellow oil. **^{1}H NMR** (300 MHz, $CDCl_3$) δ = 3.69 (s, 3H, CH_3), 3.51 – 3.46 (m, 2H, CH_2), 2.52 – 2.48 (t, *J* = 7.1 Hz, 2H, CH_2), 2.04 – 1.93 (m, 2H, CH_2); **^{13}C NMR** (75 MHz, $CDCl_3$) δ = 172.7 (C=O), 156.9 (t, *J* = 5.5 Hz, CN), 51.9 (CH_3), 40.9 (t, *J* = 6,5 Hz, CH_2), 30.3 (CH_2), 24.4 (CH_2). Spectroscopic data matched with those reported in literature.[4]

Synthesis and characterization data of oxazolines 3

General procedure for the synthesis of racemic compounds 3

To a solution of the aldehyde **1** (0.12 mmol, 1.2 equiv.) and the achiral squaramide 3-((3,5-bis(trifluoromethyl)phenyl)amino)-4-((3-(dimethylamino)propyl)amino) cyclobutane-1,2-dione (0.01 mmol, 0.1 equiv.) in CH_2Cl_2 (1.0 mL), at room temperature, the isocyano ester **2** (0.1 mmol, 1.0 equiv.) and silver oxide (0.0025 mmol, 0.025 equiv.) were added and the reaction was stirred for 0.5 h. After that time, compound **3** was purified by column chromatography.

General procedure for the enantioselective synthesis of compounds 3

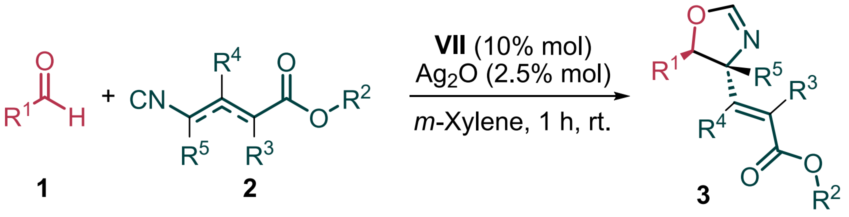

To a solution of the aldehyde **1** (0.12 mmol, 1.2 equiv.) and catalyst **VII** (5.8 mg, 0.01 mmol, 0.1 equiv.) in *m*-xylene (1.0 mL), at room temperature, the isocyano ester **2** (0.1 mmol, 1.0 equiv.) and silver oxide (0.58 mg, 0.0025 mmol, 0.025 equiv.) were added, and the reaction was stirred for 1 h. After that time, compound **3** was purified by column chromatography.

Methyl (*E*)-3-((4*R*,5*R*)-5-phenyl-4,5-dihydrooxazol-4-yl)acrylate (3aa)

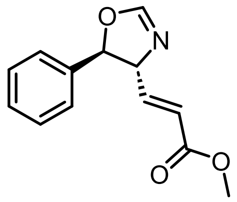

17.1 mg (74%) of **3aa** were obtained from **1a** and **2a** after column chromatography eluting with hexane: EtOAc (9:1 to 8:2). Enantiomeric excess (90%) was measured by HPLC (CHIRALPAK® IC), hexane:iPrOH 80:20, 1.0 mL/min, minor enantiomer: tr = 25.3 min, major enantiomer: tr = 38.7 min.

Pale yellow oil; $[\alpha]_D^{25}$ -150.5 (c 0.19, $CHCl_3$); **^{1}H NMR** (300 MHz, $CDCl_3$): δ = 7.41 – 7.38 (m, 3H, Ar), 7.31 – 7.28 (m, 2H, Ar), 7.09 (d, *J* = 2.0 Hz, 1H, N=CHO), 7.00 (ddd, *J* = 15.6, 6.3, 0.6 Hz, 1H, CH), 6.07 (dd, *J* = 15.6, 1.4 Hz, 1H, CH), 5.13 (d, *J* = 8.1 Hz, 1H, CH), 4.64 (dddd, *J* = 8.2, 6.2, 2.0, 1.4 Hz, 1H, CH), 3.76 (s, 3H, CH_3). **^{13}C NMR** (75 MHz, $CDCl_3$): δ = 166.6 (C=O), 155.6 (N=CHO), 145.7 (CH), 139.0 (C), 129.2 (Ar), 129.0 (Ar), 125.7 (Ar), 122.7 (CH), 84.9 (CH), 74.5 (CH), 51.9 (CH_3). **HRMS (ESI)** m/z 232.0972 $[M+H]^+$ $C_{13}H_{14}NO_3^+$ requires 232.0968.

Methyl (*E*)-3-((4*R*,5*R*)-5-(*p*-tolyl)-4,5-dihydrooxazol-4-yl)acrylate (3ba)

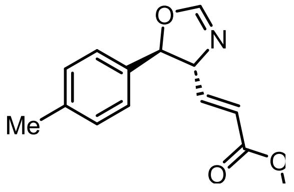

16.7 mg (68%) of **3ba** were obtained from **1b** and **2a** after column chromatography eluting with hexane:EtOAc (9:1 to 8:2). Enantiomeric excess (87%) was measured by HPLC (CHIRALPAK® IC), hexane:iPrOH 80:20, 1.0 mL/min, minor enantiomer: tr = 22.1 min, major enantiomer: tr = 35.2 min.

Pale yellow oil; $[\alpha]_D^{25}$ -142.9 (c 0.17, $CHCl_3$); **^{1}H NMR** (300 MHz, $CDCl_3$): δ = 7.20 – 7.19 (m, 4H, Ar), 7.07 (dt, *J* = 2.0, 0.5 Hz, 1H, N=CHO), 6.99 (ddd, *J* = 15.7, 6.2, 0.5 Hz, 1H, CH), 6.06 (dd, *J* = 15.6, 1.4 Hz, 1H, CH), 5.09 (d, *J* = 8.2 Hz, 1H, CH), 4.62 (dddd, *J* = 8.2, 6.2, 2.0, 1.5 Hz, 1H, CH), 3.76 (s, 3H, CH_3), 2.37 (s, 3H, CH_3). **^{13}C NMR** (75 MHz, $CDCl_3$): δ = 166.7 (C=O), 155.7 (N=CHO), 145.9 (CH), 139.0 (C), 136.0 (C), 130.0 (Ar), 125.8 (Ar), 122.6 (CH), 85.0 (CH), 74.4 (CH), 51.9 (CH_3), 21.3 (CH_3). **HRMS (ESI)** m/z 246.1114 $[M+H]^+$ $C_{14}H_{16}NO_3^+$ requires 246.1125.

Methyl (*E*)-3-((4*R*,5*R*)-5-(4-methoxyphenyl)-4,5-dihydrooxazol-4-yl)acrylate (3ca)

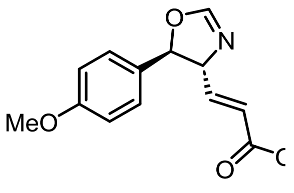

16.2 mg (62%) of **3ca** were obtained from **1c** and **2a** after column chromatography eluting with hexane:EtOAc (9:1 to 8:2). Enantiomeric excess (86%) was measured by HPLC (CHIRALPAK® IC), hexane:iPrOH 80:20, 1.0 mL/min, minor enantiomer: tr = 40.8 min, major enantiomer: tr = 49.9 min.

Pale yellow oil; $[\alpha]_D^{25}$ -132.1 (c 0.25, $CHCl_3$); **^{1}H NMR** (300 MHz, $CDCl_3$): δ = 7.24 – 7.21 (m, 2H, Ar), 7.05 (d, *J* = 2.0 Hz, 1H, N=CHO), 6.97 (ddd, *J* = 15.6, 6.3, 0.6 Hz, 1H, CH), 6.93 – 6.90 (m, 2H, Ar), 6.05 (dd, *J* = 15.6, 1.4 Hz, 1H, CH), 5.07 (d, *J* = 8.2 Hz, 1H, CH), 4.63 (dddd, *J* = 8.2, 6.2, 2.0, 1.5 Hz, 1H, CH), 3.82 (s, 3H, CH_3),

3.75 (s, 3H, CH_3).^{13}**C NMR** (75 MHz, $CDCl_3$): δ = 166.6 (C=O), 160.2 (C), 155.6 (N=CHO), 145.9 (CH), 130.8 (C), 127.4 (Ar), 122.6 (CH), 114.6 (Ar), 84.9 (CH), 74.2 (CH), 55.5 (CH_3), 51.9 (CH_3). **HRMS (ESI)** m/z 234.1124 [M-O-CH_3] $C_{13}H_{15}NO_3$ requires 234.1125.

Methyl (*E*)-3-((4*R*,5*R*)-5-(4-chlorophenyl)-4,5-dihydrooxazol-4-yl)acrylate (3da)

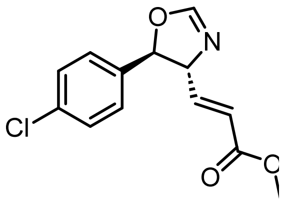

17.5 mg (66%) of **3da** were obtained from **1d** and **2a** after column chromatography eluting with Hexane:EtOAc 9:1, 8:2. Enantiomeric excess (84%) was measured by HPLC (CHIRALPAK® IC), hexane:iPrOH 80:20, 1.0 mL/min, minor enantiomer: tr = 26.7 min, major enantiomer: tr = 30.5 min.

Pale yellow oil; $[\alpha]_D^{25}$ -121.5 (c 0.20, $CHCl_3$); ^{1}**H NMR** (300 MHz, $CDCl_3$): δ = 7.38 (d, *J* = 8.6 Hz, 2H, Ar), 7.23 (d, *J* = 8.2 Hz, 2H, Ar), 7.07 (d, *J* = 2.0 Hz, 1H, N=CHO), 6.98 (ddd, *J* = 15.6, 6.3, 0.6 Hz, 1H, CH), 6.06 (dd, *J* = 15.6, 1.4 Hz, 1H, CH), 5.10 (d, *J* = 8.1 Hz, 1H, CH), 4.59 (dddd, *J* = 8.2, 6.3, 2.0, 1.4 Hz, 1H, CH), 3.76 (s, 3H, CH_3). ^{13}**C NMR** (75 MHz, $CDCl_3$): δ = 166.5 (C=O), 155.5 (N=CHO), 145.3 (CH), 137.5 (C), 134.9 (C), 129.4 (Ar), 127.1 (Ar), 123.0 (CH), 84.2 (CH), 74.5 (CH), 51.9 (CH_3). **HRMS (ESI)** m/z 266.0569 $[M+H]^+$ $C_{13}H_{13}ClNO_3^+$ requires 266.0578.

Methyl (*E*)-3-((4*R*,5*R*)-5-(4-bromophenyl)-4,5-dihydrooxazol-4-yl)acrylate (3ea)

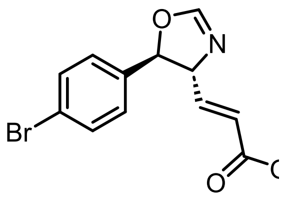

20.2 mg (65%) of **3ea** were obtained from **1e** and **3a** after column chromatography eluting with Hexane:EtOAc (9:1 to 8:2). Enantiomeric excess (85%) was measured by HPLC (CHIRALPAK® IC), hexane:iPrOH 80:20, 1.0 mL/min, minor enantiomer: tr = 20.2 min, major enantiomer: tr = 23.7 min.

Pale yellow oil; $[\alpha]_D^{25}$ -135.9 (c 0.18, $CHCl_3$); ^{1}**H NMR** (300 MHz, $CDCl_3$): δ = 7.55 – 7.52 (m, 2H, Ar), 7.18 – 7.15 (m, 2H, Ar), 7.07 (d, *J* = 2.0 Hz, 1H, N=CHO), 6.97 (dd, *J* = 15.6, 6.4 Hz, 1H, CH), 6.06 (dd, *J* = 15.6, 1.4 Hz, 1H, CH), 5.09 (d, *J* = 8.1 Hz, 1H, CH), 4.61 – 4.55 (m, 1H, CH), 3.77 (s, 3H, CH_3). ^{13}**C NMR** (75 MHz, $CDCl_3$): δ = 166.5 (C=O), 155.5 (N=CHO), 145.3 (CH), 138.0 (C), 132.4 (Ar), 127.3 (Ar), 123.1 (C), 123.0 (CH), 84.2 (CH), 74.5 (CH), 52.0 (CH_3). **HRMS (ESI)** m/z 310.0065 $[M+H]^+$ $C_{13}H_{13}BrNO_3^+$ requires 310.0073.

Methyl (*E*)-3-((4*R*,5*R*)-5-(4-nitrophenyl)-4,5-dihydrooxazol-4-yl)acrylate (3fa)

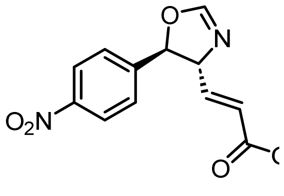

21.1 mg (76%) of **3fa** were obtained from **1f** and **2a** after column chromatography eluting with hexane:EtOAc (9:1 to 7:3). Enantiomeric excess (37%) was measured by HPLC (CHIRALPAK® IC), hexane:iPrOH 80:20, 1.0 mL/min, major enantiomer: tr = 40.9 min, minor enantiomer: tr = 43.1 min.

Pale yellow oil; $[\alpha]_D^{25}$ -134.2 (c 0.15, $CHCl_3$); ^{1}**H NMR** (300 MHz, $CDCl_3$): δ = 8.27 (d, *J* = 83Hz, 2H, Ar), 7.47 (d, *J* = 8.3 Hz, 2H, Ar), 7.12 (d, *J* = 2.0 Hz, 1H, N=CHO), 6.98 – 6.96 (m, 1H, CH), 6.09 (dd, *J* = 15.6, 1.4 Hz, 1H, CH), 5.24 (d, *J* = 8.0 Hz, 1H, CH), 4.59 (dddd, *J* = 7.9, 6.4, 2.0, 1.4 Hz, 1H, CH), 3.78 (s, 3H, CH_3).^{13}**C NMR** (75 MHz, $CDCl_3$): δ = 166.3 (C=O), 155.3 (N=CHO), 148.3 (C), 146.1 (C), 144.6 (CH), 126.3 (Ar), 124.5 (Ar), 123.5 (CH), 83.5 (CH), 74.7 (CH), 52.1 (CH_3). **HRMS (ESI)** m/z 277.0819 $[M+H]^+$ $C_{13}H_{13}N_2O_5^+$ requires 277.0819.

Methyl (*E*)-3-((4*R*,5*R*)-5-(*m*-tolyl)-4,5-dihydrooxazol-4-yl)acrylate (3ga)

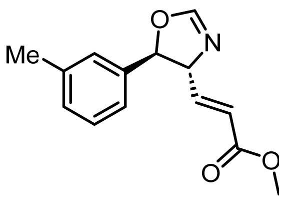

16.9 mg (68%) of **3ga** were obtained from **1g** and **2a** after column chromatography eluting with Hexane:EtOAc (9:1 to 8:2). Enantiomeric excess (85%) was measured by HPLC (CHIRALPAK® IC), hexane:iPrOH 80:20, 1.0 mL/min, minor enantiomer: tr = 21.0 min, major enantiomer: tr = 32.3 min.

Pale yellow oil; $[\alpha]_D^{25}$ -111.2 (c 0.22, $CHCl_3$); **^{1}H NMR** (300 MHz, $CDCl_3$): δ = 7.30 (d, *J* = 7.6 Hz, 1H, Ar), 7.17 (d, *J* = 7.8 Hz, 1H, Ar), 7.10 – 7.07 (m, 3H, 2Ar, N=CHO), 7.00 (ddd, *J* = 15.5, 6.2, 0.6 Hz, 1H, CH), 6.07 (dd, *J* = 15.6, 1.5 Hz, 1H, CH), 5.09 (d, *J* = 8.1 Hz, 1H, CH), 4.63 (dddd, *J* = 8.1, 6.2, 2.0, 1.4 Hz, 1H, CH), 3.76 (s, 3H, CH_3), 2.37 (s, 3H, CH_3). **^{13}C NMR** (75 MHz, $CDCl_3$): δ = 166.7 (C=O), 155.6 (N=CHO), 145.9 (CH), 139.1 (C), 139.0 (C), 129.8 (Ar), 129.1 (Ar), 126.3 (Ar), 122.8 (Ar), 122.6 (CH), 85.0 (CH), 74.4 (CH), 51.9 (CH_3), 21.6 (CH_3). **HRMS (ESI)** m/z 246.1122 $[M+H]^+$ $C_{14}H_{16}NO_3^+$ requires 246.1125.

methyl (*E*)-3-((4*R*,5*R*)-5-(*o*-tolyl)-4,5-dihydrooxazol-4-yl)acrylate (3ha)

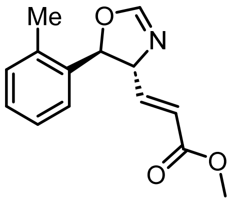

17.2 mg (70%) of 3ha were obtained from **1h** and **2a** after column chromatography eluting with hexane:EtOAc (9:1 to 8:2). Enantiomeric excess (90%) was measured by HPLC (CHIRALPAK® IC), hexane:iPrOH 80:20, 1.0 mL/min, minor enantiomer: tr = 21.2 min, major enantiomer: tr = 48.8 min.

Pale yellow oil; $[\alpha]_D^{25}$ -130.9 (c 0.18, $CHCl_3$); **^{1}H NMR** (300 MHz, $CDCl_3$): δ = 7.25 – 7.20 (m, 4H, Ar), 7.11 (dd, *J* = 1.9, 0.5 Hz, 1H, N=CHO), 7.01 (ddd, *J* = 15.5, 6.6, 0.5 Hz, 1H, CH), 6.07 (dd, *J* = 15.6, 1.4 Hz, 1H, CH), 5.39 (d, *J* = 7.4 Hz, 1H, CH), 4.61 (dddd, *J* = 7.4, 6.6, 1.9, 1.3 Hz, 1H, CH), 3.76 (s, 3H, CH_3), 2.34 (d, *J* = 0.6 Hz, 3H, CH_3). **^{13}C NMR** (75 MHz, $CDCl_3$): δ = 166.6 (C=O), 155.7 (N=CHO), 145.8 (CH), 136.9 (C), 135.1 (C), 131.1 (Ar), 128.7 (Ar), 126.8 (Ar), 125.4 (Ar), 122.9 (CH), 82.3 (CH), 73.6 (CH), 51.9 (CH_3), 19.7 (CH_3). **HRMS (ESI)** m/z 246.1122 $[M+H]^+$ $C_{14}H_{16}NO_3^+$ requires 246.1125.

Methyl (*E*)-3-((4*R*,5*R*)-5-(2-methoxyphenyl)-4,5-dihydrooxazol-4-yl)acrylate (3ia)

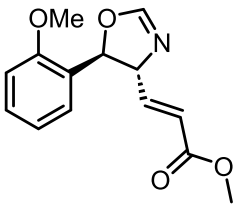

18.1 mg (69%) of **3ia** were obtained from **1i** and **2a** after column chromatography eluting with Hexane: EtOAc 9:1, 8:2. Enantiomeric excess (88%) was measured by HPLC (CHIRALPAK® IC), hexane:iPrOH 80:20, 1.0 mL/min, minor enantiomer: tr = 24.5 min, major enantiomer: tr = 40.7 min.

Pale yellow oil; $[\alpha]_D^{25}$ -232.7 (c 0.15, $CHCl_3$); **^{1}H NMR** (300 MHz, $CDCl_3$): δ = 7.31 (td, *J* = 7.8, 1.7 Hz, 1H, Ar), 7.25 (dd, *J* = 7.6, 2.8 Hz, 1H, Ar), 7.13 (ddd, *J* = 15.7, 5.6, 0.6 Hz, 1H, CH), 7.09 – 7.08 (m, 1H, N=CHO), 6.97 (td, *J* = 7.5, 0.8 Hz, 1H, Ar), 6.91 (dd, *J* = 8.2, 1.0 Hz, 1H, Ar), 6.07 (dd, *J* = 15.6, 1.6 Hz, 1H, CH), 5.44 (d, *J* = 7.0 Hz, 1H, CH), 4.58 (ddt, *J* = 7.2, 5.6, 1.7 Hz, 1H, CH), 3.86 (s, 3H, CH_3), 3.76 (s, 3H, CH_3). **^{13}C NMR** (75 MHz, $CDCl_3$): δ = 167.0 (C=O), 156.3 (C), 155.3 (N=CHO), 147.1 (CH), 129.6 (Ar), 127.7 (C), 125.7 (Ar), 121.8 (CH), 120.8 (Ar), 110.6 (Ar), 80.6 (CH), 73.3 (CH), 55.4 (CH_3), 51.8 (CH_3). **HRMS (ESI)** m/z 262.1072 $[M+H]^+$ $C_{14}H_{16}NO_4^+$ requires 262.1074.

Methyl (*E*)-3-((4*R*,5*R*)-5-(2-chlorophenyl)-4,5-dihydrooxazol-4-yl)acrylate (3ja)

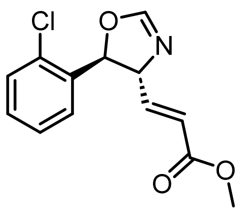

16.8 mg (63%) of **3ja** were obtained from **1j** and **2a** after column chromatography eluting with hexane:EtOAc (9:1 to 8:2). Enantiomeric excess (87%) was measured by HPLC (CHIRALPAK® IC), hexane:iPrOH 80:20, 1.0 mL/min, minor enantiomer: tr = 24.4 min, major enantiomer: tr = 36.9 min.

Pale yellow oil; $[\alpha]_D^{25}$ -122.4 (c 0.36, $CHCl_3$); **^{1}H NMR** (300 MHz, $CDCl_3$): δ = 7.39 – 7.35 (m, 1H, Ar), 7.27 – 7.22 (m, 3H, Ar), 7.10 (d, *J* = 1.8 Hz, 1H, N=CHO), 7.10 (dd, *J* = 15.6, 6.0 Hz, 1H, CH), 6.05 (dd, *J* = 15.7, 1.5 Hz, 1H, CH), 5.53 (d, *J* = 6.2 Hz, 1H, CH), 4.57 (tt, *J* = 6.1, 1.7 Hz, 1H, CH), 3.72 (s, 3H, CH_3). **^{13}C NMR** (75 MHz, $CDCl_3$): δ = 166.7 (C=O), 155.3 (N=CHO), 145.7 (CH), 136.9 (C), 131.7 (C), 130.1 (Ar), 129.8 (Ar), 127.5 (Ar), 126.3 (Ar), 122.8 (CH), 81.3 (CH), 73. 6 (CH), 51.9 (CH_3). **HRMS (ESI)** m/z 266.0580 $[M+H]^+$ $C_{13}H_{13}ClNO_3^+$ requires 266.0578.

Methyl (*E*)-3-((4*R*,5*S*)-5-(furan-2-yl)-4,5-dihydrooxazol-4-yl)acrylate (3ka)

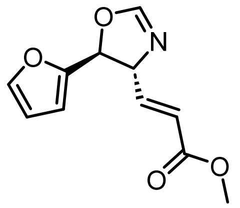

17.7 mg (80%) of **3ka** were obtained from **1k** and **2a** after column chromatography eluting with Hexane:EtOAc 9:1, 8:2. Enantiomeric excess (85%) was measured by HPLC (CHIRALPAK® IC), hexane:iPrOH 80:20, 1.0 mL/min, minor enantiomer: tr = 23.8 min, major enantiomer: tr = 33.0 min.

Pale yellow oil; $[\alpha]_D^{25}$ -170.6 (c 0.31, $CHCl_3$); **^{1}H NMR** (300 MHz, $CDCl_3$): δ = 7.47 (dd, *J* = 1.8, 0.8 Hz, 1H), 6.99 – 6.91 (m, 2H, N=CHO, CH), 6.45 (ddd, *J* = 3.3, 0.4 Hz, 1H, Ar), 6.39 (dd, *J* = 3.3, 1.8 Hz, 1H, Ar), 6.07 (dd, *J* = 15.6, 1.5 Hz, 1H, CH), 5.13 (d, *J* = 8.3 Hz, 1H, CH), 4.95 (dddd, *J* = 8.2, 6.0, 2.0, 1.5 Hz, 1H, CH), 3.75 (s, 3H, CH_3). **^{13}C NMR** (75 MHz, $CDCl_3$): δ = 166.5 (C=O), 155.2 (N=CHO), 150.0 (C), 145.3 (CH), 144.1 (Ar), 122.9 (CH), 110.8 (Ar), 110.2 (Ar), 77.8 (CH), 70.0, (CH), 51.9 (CH_3). **HRMS (ESI)** m/z 222.0753 $[M+H]^+$ $C_{11}H_{12}NO_4^+$ requires 222.0761.

Methyl (*E*)-3-((4*R*,5*R*)-5-(furan-3-yl)-4,5-dihydrooxazol-4-yl)acrylate (3la)

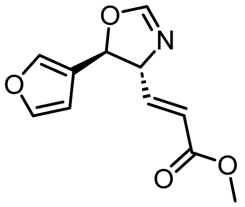

16.4 mg (74%) of **3la** were obtained from **1l** and **2a** after column chromatography eluting with hexane:EtOAc (9:1 to 8:2). Enantiomeric excess (90%) was measured by HPLC (CHIRALPAK® IC), hexane:iPrOH 80:20, 1.0 mL/min, minor enantiomer: tr = 29.0 min, major enantiomer: tr = 37.3 min.

Pale yellow oil; $[\alpha]_D^{25}$ -163.1 (c 0.41, $CHCl_3$); **^{1}H NMR** (300 MHz, $CDCl_3$): δ = 7.49 (td, *J* = 0.9, 0.4 Hz, 1H, Ar), 7.46 (td, *J* = 1.5, 0.8 Hz, 1H, Ar), 7.00 (dt, *J* = 2.0, 0.5 Hz, 1H, N=CHO), 6.95 (ddd, *J* = 15.6, 6.1, 0.5 Hz, 1H, CH), 6.39 (ddd, *J* = 1.9, 0.9, 0.4 Hz, 1H, CH), 6.06 (dd, *J* = 15.6, 1.5 Hz, 1H, CH), 5.11 (d, *J* = 8.1 Hz, 1H, CH), 4.64 (dddd, *J* = 8.1, 6.1, 2.0, 1.5 Hz, 1H, CH), 3.75 (s, 3H, CH_3). **^{13}C NMR** (75 MHz, $CDCl_3$): δ = 166.5 (C=O), 155.6 (N=CHO), 145.4 (CH), 144. 7 (Ar), 140.5 (Ar), 123.6 (C), 122.8 (CH), 108.1 (Ar), 77.9 (CH), 72.6 (CH), 51.9 (CH_3). **HRMS (ESI)** m/z 222.0752 $[M+H]^+$ $C_{11}H_{12}NO_4^+$ requires 222.0761.

Methyl (*E*)-3-((4*R*,5*R*)-5-(thiophen-3-yl)-4,5-dihydrooxazol-4-yl)acrylate (3ma)

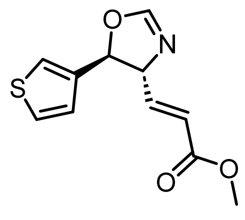

19.3 mg (81%) of **3ma** were obtained from **1m** and **2a** after column chromatography eluting with hexane:EtOAc (9:1 to 8:2). Enantiomeric excess (90%) was measured by HPLC (CHIRALPAK® IC), hexane:iPrOH 80:20, 1.0 mL/min, minor enantiomer: tr = 32.0 min, major enantiomer: tr = 44.4 min.

Pale yellow oil; $[\alpha]_D^{25}$ -134.7 (c 0.31, $CHCl_3$); **^{1}H NMR** (300 MHz, $CDCl_3$): δ = 7.39 (ddd, *J* = 5.1, 3.0, 0.4 Hz, 1H, Ar), 7.29 (ddd, *J* = 3.0, 1.4, 0.7 Hz, 1H, Ar), 7.05 (dd, *J* = 1.4, 0.4 Hz, 1H, Ar), 7.04 – 7.03 (m, 1H, N=CHO), 6.98 (ddd, *J* = 15.7, 6.2, 0.5 Hz, 1H, CH), 6.07 (dd, *J* = 15.6, 1.5 Hz, 1H, CH), 5.22 (d, *J* = 8.0 Hz, 1H, CH), 4.68 (dddd, *J* = 8.0, 6.1, 2.0, 1.4 Hz, 1H, CH), 3.76 (s, 3H, CH_3). **^{13}C NMR** (75 MHz, $CDCl_3$): δ = 166.6 (C=O), 155.5 (N=CHO), 145. 6 (CH), 139.7 (C), 127.8 (Ar), 125.0 (Ar), 123.0 (Ar), 122.8 (CH), 81.1 (CH), 73.3 (CH), 51.9 (CH_3). **HRMS (ESI)** m/z 238.0523 $[M+H]^+$ $C_{11}H_{12}NO_3S^+$ requires 238.0532.

Methyl (*E*)-3-((4*R*,5*R*)-5-(naphthalen-2-yl)-4,5-dihydrooxazol-4-yl)acrylate (3na)

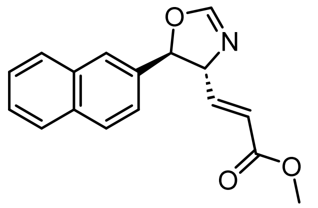

19.7 mg (70%) of **3na** were obtained from **1n** and **2a** after column chromatography eluting with hexane:EtOAc (9:1 to 8:2). Enantiomeric excess (84%) was measured by HPLC (CHIRALPAK® IC), hexane:iPrOH 80:20, 1.0 mL/min, minor enantiomer: tr = 36.2 min, major enantiomer: tr = 47.7 min.

Pale yellow oil; $[\alpha]_D^{25}$ -95.4 (c 0.28, $CHCl_3$); **^{1}H NMR** (300 MHz, $CDCl_3$): δ = 7.90 (d, *J* = 8.5 Hz, 1H, Ar), 7.86 – 7.83 (m, 2H, Ar), 7.77 – 7.76 (m, 1H, Ar), 7.54 – 7.51 (m, 2H, Ar), 7.38 (dd, *J* = 8.6, 1.8 Hz, 1H, Ar), 7.15 (dt, *J* = 2.0, 0.5 Hz, 1H, N=CHO), 7.05 (ddd, *J* = 15.6, 6.2, 0.5 Hz, 1H, CH), 6.09 (dd, *J* = 15.6, 1.4 Hz, 1H, CH), 5.30 (d, *J* = 8.1 Hz, 1H, CH), 4.73 (dddd, *J* = 8.2, 6.3, 2.0, 1.4 Hz, 1H, CH), 3.77 (s, 3H, CH_3). **^{13}C NMR** (75 MHz, $CDCl_3$): δ = 166.6 (C=O), 155.7 (N=CHO), 145.8 (CH), 136.1 (C), 133.5 (C), 133.2 (C), 129.5 (Ar), 128.2 (Ar), 128.0 (Ar), 126.9 (Ar), 126.8 (Ar), 125.3 (Ar), 122.8 (Ar), 122.8 (CH), 85.2 (CH), 74.4 (CH), 51.9 (CH_3). **HRMS (ESI)** m/z 282.1123 $[M+H]^+$ $C_{17}H_{16}NO_3^+$ requires 282.1125.

Methyl (*E*)-3-((4*R*,5*R*)-5-phenethyl-4,5-dihydrooxazol-4-yl)acrylate (3oa)

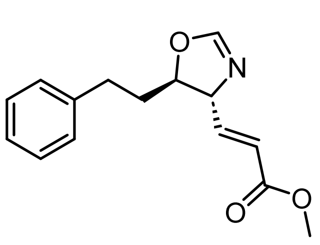

18.7 mg (72%) of **3oa** were obtained from **1o** and **2a** after column chromatography eluting with hexane:EtOAc (9:1 to 8:2). Enantiomeric excess (74%) was measured by HPLC (CHIRALPAK® IC), hexane:iPrOH 80:20, 1.0 mL/min, major enantiomer: tr = 19.4 min, minor enantiomer: tr = 25.6 min.

Pale yellow oil; $[\alpha]_D^{25}$ -100.0 (c 0.3, $CHCl_3$); **^{1}H NMR** (300 MHz, $CDCl_3$): δ = 7.29 (dd, *J* = 7.2, 1.2 Hz, 2H, Ar), 7.22 – 7.17 (m, 3H, Ar), 6.92 (d, *J* = 1.8 Hz, 1H, N=CHO), 6.84 (ddd, *J* = 15.6, 6.2, 0.5 Hz, 1H, CH), 6.00 (dd, *J* = 15.6, 1.4 Hz, 1H, CH), 4.34 (ddt, *J* = 7.5, 6.2, 1.7 Hz, 1H, CH), 4.21 (td, *J* = 7.6, 5.1 Hz, 1H, CH), 3.74 (s, 3H, CH_3), 2.81 – 2.70 (m, 2H, CH_2), 2.04 – 1.93 (m, 2H, CH_2). **^{13}C NMR** (75 MHz, $CDCl_3$): δ = 166.5 (C=O), 155.6 (N=CHO), 146.0 (CH), 140.4 (C), 128.6 (Ar), 128.4 (Ar), 126.3 (Ar), 122.1 (CH), 82.7 (CH), 70.9 (CH), 51.8 (CH_3), 36.6 (CH_2), 31.4 (CH_2). **HRMS (ESI)** m/z 260.1285 $[M+H]^+$ $C_{15}H_{18}NO_3^+$ requires 260.1281.

Methyl (*E*)-3-((4*R*,5*R*)-5-cyclopropyl-4,5-dihydrooxazol-4-yl)acrylate (3pa)

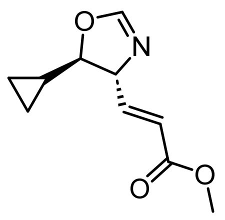

18.0 mg (92%) of **3pa** were obtained from **1p** and **2a** after column chromatography eluting with hexane:EtOAc (9:1 to 8:2). Enantiomeric excess (87%) was measured by HPLC (CHIRALPAK® IC), hexane:iPrOH 80:20, 1.0 mL/min, minor enantiomer: tr = 31.5 min, major enantiomer: tr = 39.0 min.

Yellow oil; $[\alpha]_D^{25}$ -114.6 (c 0.24, $CHCl_3$); **^{1}H NMR** (300 MHz, $CDCl_3$): δ = 6.90 – 6.83 (m, 2H, N=CHO, CH), 6.05 N=CHO (dd, *J* = 15.6, 1.5 Hz, 1H, CH), 4.50 (ddt, *J* = 7.7, 6.1, 1.7 Hz, 1H, CH), 3.73 (s, 3H, CH_3), 3.63 (t, *J* = 7.9 Hz, 1H, CH), 1.07 (qt, *J* = 8.2, 4.9 Hz, 1H, CH), 0.69 – 0.60 (m, 2H, CH_2), 0.46 – 0.32 (m, 2H, CH_2). **^{13}C NMR** (75 MHz, $CDCl_3$): δ = 166.7 (C=O), 155.7 (N=CHO), 146.6 (CH), 122.1 (CH), 88.1 (CH), 71.0 (CH), 51.8 (CH_3), 14.8 (CH), 3.3 (CH_2), 1.6 (CH_2). **HRMS (ESI)** m/z 214.0904 $[M+H_3O]^+$ $C_{10}H_{16}NO_4^+$ requires 214.1074.

Methyl (*E*)-3-((4*R*,5*R*)-5-cyclopentyl-4,5-dihydrooxazol-4-yl)acrylate (3qa)

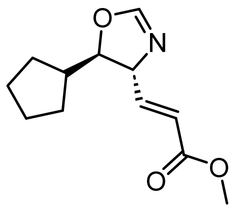

19.7 mg (88%) of **3qa** were obtained from **1q** and **2a** after column chromatography eluting with hexane:EtOAc (9:1 to 8:2). Enantiomeric excess (91%) was measured by HPLC (CHIRALPAK® IC), hexane:iPrOH 80:20, 1.0 mL/min, minor enantiomer: tr = 28.9 min, major enantiomer: tr = 37.2 min.

Yellow oil; $[\alpha]_D^{25}$ -134.1 (c 0.23, $CHCl_3$); **^{1}H NMR** (300 MHz, $CDCl_3$): δ = δ 6.88 (d, *J* = 1.9 Hz, 1H, N=CHO), 6.86 (ddd, *J* = 15.6, 6.2, 0.5 Hz, 1H, CH), 6.00 (dd, *J* = 15.6, 1.4 Hz, 1H, CH), 4.36 (ddt, *J* = 7.8, 6.3, 1.7 Hz, 1H, CH), 4.07 (t, *J* = 7.5 Hz, 1H, CH), 3.73 (s, 3H, CH_3), 2.11 (q, *J* = 8.0 Hz, 1H, CH), 1.81 – 1.54 (m, 6H, $3CH_2$), 1.40– 1.20 (m, 2H, CH_2). **^{13}C NMR** (75 MHz, $CDCl_3$): δ = δ 166.7 (C=O), 155.9 (N=CHO), 147.0 (CH), 122.0 (CH), 87.4 (CH), 70.0 (CH), 51.8 (CH_3), 44.2 (CH), 28.8, 27.9 (CH_2), 25.5 (CH_2). **HRMS (ESI)** m/z 224.1284 $[M+H]^+$ $C_{12}H_{18}NO_3^+$ requires 224.1281.

Methyl (*E*)-3-((4*R*,5*R*)-5-cyclohexyl-4,5-dihydrooxazol-4-yl)acrylate (3ra)

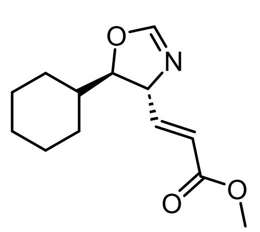

23.3 mg (98%) of **3ra** were obtained from **1r** and **2a** after column chromatography eluting with hexane:EtOAc (9:1 to 8:2). Enantiomeric excess (93%) was measured by HPLC (CHIRALPAK® IC), hexane:iPrOH 80:20, 1.0 mL/min, minor enantiomer: tr = 32.9 min, major enantiomer: tr = 37.0 min.

Yellow oil; $[\alpha]_D^{25}$ -198.3 (c 0.58, $CHCl_3$); **^{1}H NMR** (300 MHz, $CDCl_3$): δ = 6.87 (d, *J* = 1.8 Hz, 1H, N=CHO), 6.84 (ddd, *J* = 15.6, 6.1, 0.6 Hz, 1H, CH), 6.00 (dd, *J* = 15.6, 1.5 Hz, 1H, CH), 4.42 (ddt, *J* = 7.7, 6.1, 1.7 Hz, 1H, CH), 3.94 (t, *J* = 7.2 Hz, 1H, CH), 3.73 (s, 3H, CH_3), 1.84 – 1.51 (m, 6H, $3CH_2$), 1.30 – 0.97 (m, 3H, CH, $2CH_2$). **^{13}C NMR** (75 MHz, $CDCl_3$): δ = 166.8 (C=O), 155.7 (N=CHO), 147.3 (CH), 121.9 (CH), 87.7 (CH), 68.5 (CH), 51.8 (CH_3), 42.0 (CH), 28.3 (CH_2), 27.9 (CH_2), 26.3 (CH_2), 25.7 (CH_2), 25.6 (CH_2). **HRMS (ESI)** m/z 138.1442 $[M+H]^+$ $C_{14}H_{20}NO_3^+$ requires 238.1438.

Methyl (*E*)-3-((4*R*,5*R*)-5-isopropyl-4,5-dihydrooxazol-4-yl)acrylate (3sa)

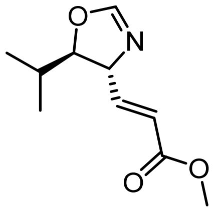

16.0 mg (81%) of **3sa** were obtained from **16** and **2a** after column chromatography eluting with hexane:EtOAc (9:1 to 8:2). Enantiomeric excess (91%) was measured by HPLC (CHIRALPAK® IC), hexane:iPrOH 80:20, 1.0 mL/min, minor enantiomer: tr = 19.2 min, major enantiomer: tr = 26.9 min.

Colorless oil; $[\alpha]_D^{25}$ -132.2 (c 0.32, $CHCl_3$); **^{1}H NMR** (400 MHz, $CDCl_3$): δ = 6.88 (d, *J* = 1.6 Hz, 1H, N=CHO), 6.85 (ddd, *J* = 15.6, 6.4, 0.5 Hz, 1H, CH), 6.01 (dd, *J* = 15.6, 1.2 Hz, 1H, CH), 4.39 (ddt, *J* = 7.7, 6.1, 1.6 Hz, 1H, CH), 3.96 (t, *J* = 6.8 Hz, 1H, CH), 3.73 (s, 3H, CH_3), 1.86 (hept, *J* = 6.8 Hz, 1H, *i*Pr), 0.96 (d, *J* = 6.8 Hz, 3H, *i*Pr), 0.94 (d, *J* = 6.8 Hz, 3H, *i*Pr). **^{13}C NMR** (100 MHz, $CDCl_3$): δ = 166.8 (C=O), 155.8 (N=CHO), 147.2 (CH), 121.9 (CH), 88.5 (CH), 68.4 (CH), 51.8 (CH_3), 32.3 (CH), 17.7 (CH_3), 17.4 (CH_3). **HRMS (ESI)** m/z 198.1127 $[M+H]^+$ $C_{10}H_{16}NO_3^+$ requires 198.1125.

Methyl (*E*)-3-((4*R*,5*R*)-5-(*tert*-butyl)-4,5-dihydrooxazol-4-yl)acrylate (3ta)

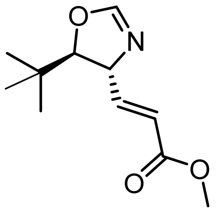

16.4 mg (78%) of **3ta** were obtained from **1t** and **2a** after column chromatography eluting with hexane:EtOAc (9:1 to 8:2). Enantiomeric excess (89%) was measured by HPLC (CHIRALPAK® IC), hexane:iPrOH 80:20, 1.0 mL/min, minor enantiomer: tr = 15.2 min, major enantiomer: tr = 25.8 min.

Colorless oil; $[\alpha]_D^{25}$ -194.2 (c 0.33, $CHCl_3$); **^{1}H NMR** (400 MHz, $CDCl_3$): δ = 6.89 (d, *J* = 2.0 Hz, 1H, N=CHO), 6.83 (dd, *J* = 15.6, 6.0 Hz, 1H, CH), 6.00 (dd, *J* = 15.6, 2.0 Hz, 1H, CH), 4.44 (ddt, *J* = 7.7, 6.0, 1.6 Hz, 1H, CH), 3.89 (d, *J* = 7.6 Hz, 1H, CH), 3.73 (s, 3H, CH_3), 0.92 (s, 9H, *t*Bu). **^{13}C NMR** (100 MHz, $CDCl_3$): δ = 166.8 (C=O), 155.8 (N=CHO), 147.7 (CH), 121.9 (CH), 91.3 (CH), 66.5 (CH), 51.8 (CH_3), 34.0 (C), 24.7 (CH_3). **HRMS (ESI)** m/z 212.1284 $[M+H]^+$ $C_{11}H_{18}NO_3^+$ requires 212.1281.

Ethyl (*E*)-3-((4*R*,5*R*)-5-phenyl-4,5-dihydrooxazol-4-yl)acrylate (3ab)

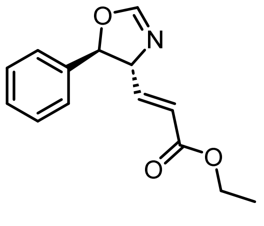

17.7 mg (72%) of **3ab** were obtained from **3a** and **2b** after column chromatography eluting with hexane:EtOAc (9:1 to 8:2). Enantiomeric excess (76%) was measured by HPLC (CHIRALPAK® IC), hexane:iPrOH 80:20, 1.0 mL/min, minor enantiomer: tr = 25.7 min, major enantiomer: tr = 35.9 min.

Pale yellow oil; $[\alpha]_D^{25}$ -114.6 (c 0.27, $CHCl_3$); **^{1}H NMR** (300 MHz, $CDCl_3$): δ = 7.41 – 7.36 (m, 3H, Ar), 7.31 (dd, *J* = 2.0, 0.6 Hz, 2H, Ar), 7.08 (dt, *J* = 1.9, 0.5 Hz, 1H, N=CHO), 6.99 (ddd, *J* = 15.6, 6.3, 0.6 Hz, 1H, CH), 6.06 (dd, *J* = 15.6, 1.4 Hz, 1H, CH), 5.14 (d, *J* = 8.1 Hz, 1H, CH), 4.64 (dddd, *J* = 8.2, 6.3, 2.0, 1.4 Hz, 1H, CH), 4.22 (q, *J* = 7.1 Hz, 2H, CH_2), 1.30 (t, *J* = 7.1 Hz, 3H, CH_3). **^{13}C NMR** (75 MHz, $CDCl_3$): δ = 166.2 (C=O), 155. 6 (N=CHO), 145.4 (CH), 139.1 (C), 129.2 (Ar), 129.0 (Ar), 125.7 (Ar), 123.2 (CH), 84.9 (CH), 74.5 (CH), 60.8 (CH_2), 14.4 (CH_3). **HRMS (ESI)** m/z 246.1122 $[M+H]^+$ $C_{14}H_{16}NO_3^+$ requires 246.1125.

Methyl (*E*)-2-methyl-3-((4*R*,5*R*)-5-phenyl-4,5-dihydrooxazol-4-yl)acrylate (3ac)

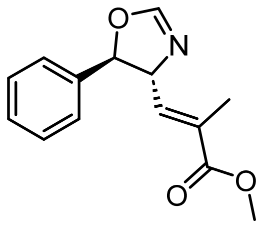

19.2 mg (78%) of **3ac** were obtained from **1a** and **2c** after column chromatography eluting with hexane:EtOAc (9:1 to 8:2). Enantiomeric excess (60%) was measured by HPLC (CHIRALPAK® IC), hexane:iPrOH 80:20, 1.0 mL/min, minor enantiomer: tr = 12.0 min, major enantiomer: tr = 18.4 min.

Pale yellow oil; $[\alpha]_D^{25}$ -62.4 (c 0.29, $CHCl_3$); **^{1}H NMR** (300 MHz, $CDCl_3$): δ = 7.43 – 7.35 (m, 3H, Ar), 7.28 (dd, *J* = 6.7, 1.3 Hz, 2H, Ar), 7.08 (d, *J* = 2.0 Hz, 1H, N=CHO), 6.70 (dd, *J* = 9.1, 1.5 Hz, 1H, CH), 5.12 (d, *J* = 7.9 Hz, 1H, CH), 4.80 (ddd, *J* = 9.1, 7.8, 2.0 Hz, 1H, CH), 3.77 (s, 3H, CH_3), 1.76 (d, *J* = 1.5 Hz, 3H, CH_3). **^{13}C NMR** (75 MHz, $CDCl_3$): δ = 168.1 (C=O), 155.5 (N=CHO), 139.6 (CH), 139.2 (C), 131.0 (C), 129.1 (Ar), 128.9 (Ar), 125.8 (Ar), 85.6 (CH), 72.1 (CH), 52.2 (CH_3), 13.4 (CH_3). **HRMS (ESI)** m/z 246.1120 $[M+H]^+$ $C_{14}H_{16}NO_3^+$ requires 246.1125.

Methyl (*E*)-3-((4*R*,5*R*)-5-cyclohexyl-4,5-dihydrooxazol-4-yl)-2-methylacrylate (3rc)

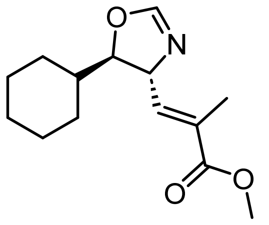

22.9 mg (91%) of **3rc** were obtained from **1r** and **2c** after column chromatography eluting with hexane:EtOAc (9:1 to 8:2). Enantiomeric excess (70%) was measured by HPLC (CHIRALPAK® IC), hexane:iPrOH 80:20, 1.0 mL/min, major enantiomer: tr = 19.6 min, minor enantiomer: tr = 25.6 min.

Pale yellow oil; $[\alpha]_D^{25}$ -74.7 (c 0.43, $CHCl_3$); **^{1}H NMR** (300 MHz, $CDCl_3$): δ = 6.86 (d, *J* = 1.9 Hz, 1H, N=CHO), 6.47 (dd, *J* = 9.4, 1.5 Hz, 1H, CH), 4.56 (ddd, *J* = 9.3, 7.1, 2.0 Hz, 1H, CH), 3.98 (t, *J* = 6.8 Hz, 1H, CH), 3.72 (s, 3H, CH_3), 1.97 (d, *J* = 1.5 Hz, 3H, CH_3), 1.83 – 1.48 (m, 6H, $3CH_2$), 1.25 – 0.95 (m, 5H, $2CH_2$, CH). **^{13}C NMR** (75 MHz, $CDCl_3$): δ = 168.2 (C=O), 155.6 (N=CHO), 140.2 (CH), 129.9 (C), 88.7 (CH), 65.8 (CH), 52.1 (CH_3), 41.7 (CH), 28.3 (CH_2), 28.2 (CH_2), 26.3 (CH_2), 25.8 (CH_2), 25.7 (CH_2), 13.2 (CH_3). **HRMS (ESI)** m/z 252.1591 $[M+H]^+$ $C_{14}H_{22}NO_3^+$ requires 252.1594.

Methyl (*E*)-3-((4*R*,5*R*)-5-phenyl-4,5-dihydrooxazol-4-yl)but-2-enoate (3ad)

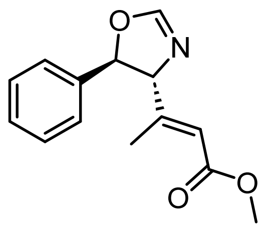

16.0 mg (65%) of **3ad** were obtained from **3a** and **2d** after column chromatography eluting with hexane:EtOAc (9:1 to 8:2). Enantiomeric excess (87%) was measured by HPLC (CHIRALPAK® IC), hexane:iPrOH 80:20, 1.0 mL/min, minor enantiomer: tr = 13.9 min, major enantiomer: tr = 22.8 min.

Pale yellow oil; $[\alpha]_D^{25}$ -123.4 (c 0.38, 7.13 (d, *J* = 1.5 Hz, 1H, N=CHO), 5.86 (dt, *J* = 2.2, 1.1 Hz, 1H, CH), 5.08 (d, *J* = 7.1 Hz, 1H, CH), 4.53 (ddd, *J* = 7.6, 2.0, 0.9 Hz, 1H, CH), 3.71 (s, 3H, CH_3), 2.15 (d, *J* = 1.4 Hz, 3H, CH_3). **^{13}C NMR** (75 MHz, $CDCl_3$): δ = 166.9 (C=O), 155.7 (C), 155.6 (N=CHO), 139.8 (C), 129.2 (Ar), 129.0 (Ar), 125.9 (Ar), 117.1 (CH), 84.5 (CH), 80.5 (CH), 51.3 (CH_3), 15.6 (CH_3). **HRMS (ESI)** m/z 246.1127 $[M+H]^+$ $C_{14}H_{16}NO_3^+$ requires 246.1125.

methyl (E)-3-((4R,5R)-5-cyclohexyl-4,5-dihydrooxazol-4-yl)but-2-enoate (3rd)

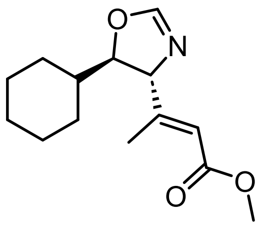

21.7 mg (86%) of **3rd** were obtained from **1r** and **2d** after column chromatography eluting with hexane:EtOAc (9:1 to 8:2). Enantiomeric excess (95%) was measured by HPLC (CHIRALPAK® IC), hexane:iPrOH 80:20, 1.0 mL/min, major enantiomer: tr = 15.0 min, minor enantiomer: tr = 23.4min.

Pale yellow oil; $[\alpha]_D^{25}$ -111.4 (c 0.5, $CHCl_3$); **^{1}H NMR** (300 MHz, $CDCl_3$): δ = 6.94 (d, *J* = 1.8 Hz, 1H, N=CHO), 5.83 – 5.81 (m, 1H, CH), 4.29 (ddd, *J* = 6.4, 1.8, 0.8 Hz, 1H, CH), 3.99 (t, *J* = 6.2 Hz, 1H, CH), 3.70 (s, 3H, CH_3), 2.08 (d, *J* = 1.4 Hz, 3H, CH_3), 1.80 – 1.64 (m, 6H, $3CH_2$), 1.27 – 1.01 (m, 5H, $2CH_2$, CH). **^{13}C NMR** (75 MHz, $CDCl_3$): δ = 167.0 (C=O), 156.8 (C), 155.9 (N=CHO), 116.9 (CH), 87.0 (CH), 74.5 (CH), 51.3 (CH_3), 42.4 (CH), 28.4 (CH_2), 27.6 (CH_2), 26.4 (CH_2), 25.9 (CH_2), 25.8 (CH_2), 15.3 (CH_3). **HRMS (ESI)** m/z 252.1588 $[M+H]^+$ $C_{14}H_{22}NO_3^+$ requires 252.1594.

Methyl (*E*)-3-((4*R*,5*R*)-4-methyl-5-phenyl-4,5-dihydrooxazol-4-yl)acrylate (3ae)

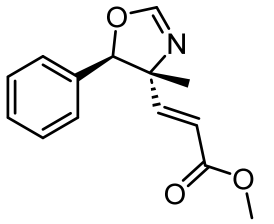

18.9 mg (77%) (major diasteromer) and 3.7 mg (15%) of **3ae** were obtained from **1a** and **2d** after column chromatography eluting with hexane:EtOAc (9:1 to 8:2). Diastereoisomeric ratio (5:1) was determined via ^{1}H NMR analysis.

Major diastereomer, enantiomeric excess (80%) was measured by HPLC (CHIRALPAK® OD-H), hexane:iPrOH 80:20, 1.0 mL/min, major enantiomer: tr = 8.0 min, minor enantiomer: tr = 9.4 min. Pale yellow oil; $[\alpha]_D^{25}$ -29.4 (c 0.32, $CHCl_3$); **^{1}H NMR** (300 MHz, $CDCl_3$): δ = 7.42 – 7.32 (m, 3H, Ar), 7.26 – 7.21 (m, 2H, Ar), 7.09 (dd, *J* = 15.6, 0.5 Hz, 1H, CH), 7.09 (s, 1H, N=CHO), 6.08 (d, *J* = 15.7 Hz, 1H, CH), 5.27 (s, 1H, CH), 3.78 (s, 3H, CH_3), 0.88 (s, 3H, CH_3). **^{13}C NMR** (75 MHz, $CDCl_3$): δ = 167.1 (C=O), 154.3 (N=CHO), 151.8 (CH), 135.5 (C), 128.7 (Ar), 128.6 (Ar), 125.9 (Ar), 120.4 (CH), 87.3 (CH), 73.4 (C), 52.0 (CH_3), 23.1 (CH_3).

Minor diastereomer, enantiomeric excess (86%) was measured by HPLC (CHIRALPAK® IC), hexane:iPrOH 80:20, 1.0 mL/min, minor enantiomer: tr = 26.1 min, major enantiomer: tr = 39.5 min. Pale yellow oil; $[\alpha]_D^{25}$ -35.6 (c 0.18, $CHCl_3$); **^{1}H NMR** (300 MHz, $CDCl_3$): δ = 7.35 – 7.30 (m, 3H, Ar), 7.16 – 7.11 (m, 3H, Ar, N=CHO), 6.25 (dd, *J* = 15.6, 0.5 Hz, 1H, CH), 5.89 (d, *J* = 15.6 Hz, 1H, CH), 5.18 (d, *J* = 0.7 Hz, 1H, CH), 3.60 (s, 3H, CH_3), 1.58 (s, 3H, CH_3). **^{13}C NMR** (75 MHz, $CDCl_3$): δ = 166.6 (C=O), 154.5 (N=CHO), 149.3 (CH), 135.5 (C), 128.9 (Ar), 128.8 (Ar), 126.1 (Ar), 120.1 (CH), 89.6 (CH), 73. 5 (C), 51.6 (CH_3), 28.0 (CH_3). **HRMS (ESI)** m/z 246.1121 $[M+H]^+$ $C_{14}H_{16}NO_3^+$ requires 246.1125.

Methyl (*E*)-3-((4*R*,5*R*)-4-methyl-5-(thiophen-3-yl)-4,5-dihydrooxazol-4-yl)acrylate (3me)

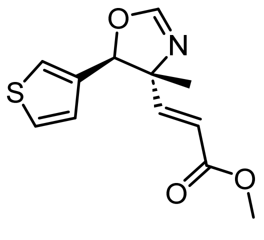

17.9 mg (71%) of **3me** were obtained from **1m** and **2e** after column chromatography eluting with hexane:EtOAc (9:1 to 8:2). Diastereoisomeric ratio (5:1), major diastereomer (15.2 mg, 60%), minor diastereomer (2.8 mg, 11%), was determined via ^{1}H NMR analysis.

Major diastereomer, enantiomeric excess (76%) was measured by HPLC (CHIRALPAK® AD-H), hexane:iPrOH 80:20, 1.0 mL/min, minor enantiomer: tr = 13.6 min, major enantiomer: tr = 19.9 min. Pale yellow oil; $[\alpha]_D^{25}$ -16.2 (c 0.26, $CHCl_3$); **^{1}H NMR** (300 MHz, $CDCl_3$): δ = 7.37 (dd, *J* = 5.0, 3.0 Hz, 1H, Ar), 7.23 – 7.20 (m, 1H, Ar), 7.10 – 7.04 (m, 2H, Ar, N=CHO), 6.97 (dd, *J* = 5.0, 1.3 Hz, 1H, CH), 6.07 (d, *J* = 15.6 Hz, 1H, CH), 5.33 (s, 1H, CH), 3.77 (s, 3H, CH_3), 0.97 (s, 3H, CH_3). **^{13}C NMR** (75 MHz, $CDCl_3$): δ = 167.1 (C=O), 154.3 (N=CHO), 151.4 (CH), 136.6 (C), 126.9 (Ar), 125.5 (Ar), 122.2 (Ar), 120.5 (CH), 84.5 (CH), 73.2 (C), 51.9 (CH_3), 22.7 (CH_3).

Minor diastereomer, enantiomeric excess (80%) was measured by HPLC (CHIRALPAK® AD-H), hexane:iPrOH 80:20, 1.0 mL/min, major enantiomer: tr = 13.0 min, minor enantiomer: tr = 21.0 min. Pale yellow oil; $[\alpha]_D^{25}$ -17.0 (c 0.2, $CHCl_3$); **^{1}H NMR** (300 MHz, $CDCl_3$): δ = 7.31 (dd, *J* = 5.0, 3.0 Hz, 1H, Ar), 7.16 (ddd, *J* = 3.0, 1.4, 0.7 Hz, 1H, Ar), 7.05 (s, 1H, N=CHO), 6.85 (dd, *J* = 5.1, 1.3 Hz, 1H, Ar), 6.39 (dd, *J* = 15.6, 0.5 Hz, 1H, CH), 5.93 (d, *J* = 15.6 Hz, 1H, CH), 5.26 (s, 1H, CH), 3.64 (s, 3H, CH_3), 1.55 (s, 3H, CH_3). **^{13}C NMR** (75 MHz, $CDCl_3$): δ = 166.7 (C=O), 154.3 (N=CHO), 149.0 (CH), 136.7 (C), 127.1 (Ar), 125.5 (Ar), 122.8 (Ar), 120.21 (CH), 86.3 (CH), 73.0 (C), 51.7 (CH_3), 27.7 (CH_3). **HRMS (ESI)** m/z 252.0690 $[M+H]^+$ $C_{12}H_{14}NO_3S^+$ requires 252.0689.

Methyl (*E*)-3-((4*R*,5*R*)-5-cyclohexyl-4-methyl-4,5-dihydrooxazol-4-yl)acrylate (3re)

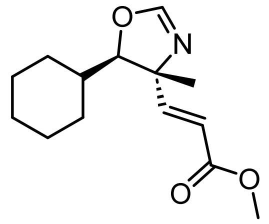

23.4 mg (93%) of **3re** were obtained from **1r** and **2e** after column chromatography eluting with hexane:EtOAc (9:1 to 8:2). Diastereoisomeric ratio (3:1), major diastereomer (0.0176 g, 70%), minor diastereomer (0.0058 g, 23%), was determined by ^{1}H NMR analysis. Major diastereomer, enantiomeric excess (-70%) was measured by HPLC (CHIRALPAK® OD-H), hexane:iPrOH 80:20, 1.0 mL/min, major enantiomer: tr = 19.8 min, minor enantiomer: tr = 32.2 min. Minor diastereomer, enantiomeric excess (85%) was measured by HPLC (CHIRALPAK® IC), hexane:iPrOH 80:20, 1.0 mL/min, major enantiomer: tr = 22.9 min, minor enantiomer: tr = 24.4 min.

Pale yellow oil; $[\alpha]_D^{25}$ -16.9 (c 0.29, $CHCl_3$); **^{1}H NMR** (300 MHz, $CDCl_3$): δ = 6.95 (d, *J* = 15.6 Hz, 1H, CH, major diastereomer), 6.93 (d, *J* = 16.2 Hz, 1H, CH, minor diastereomer), 6.86 (s, 1H, N=CHO, major diastereomer), 6.82 (s, 1H, N=CHO, minor diastereomer), 6.04 (d, *J* = 15.3 Hz, 1H, CH, minor diastereomer), 5.99 (d, *J* = 15.6 Hz, 1H, CH, major diastereomer), 3.78 (d, *J* = 8.5 Hz, 1H, CH, major diastereomer), 3.75 (d, *J* = 5.7 Hz, 1H, CH, minor diastereomer), 3.73 (s, 3H, CH_3, major diastereomer), 3.72 (s, 3H, CH_3, minor diastereomer), 1.80 – 1.60 (m, 3H, $3CH_2$, major and minor diastereomer), 1.43 (s, 3H, CH_3, major and minor diastereomer), 1.25 – 1.05 (m, 5H, $2CH_2$, CH, major and minor diastereomer).**^{13}C NMR** (75 MHz, $CDCl_3$, major diastereomer): δ = 166.9 (C=O), 154.4 (N=CHO), 147.9 (CH), 121.4

(CH), 92.6 (CH), 70.7 (C), 51.8 (CH_3), 38.5 (CH_2), 29.6 (CH_2), 29.4 (CH_2), 27.9 (CH_3), 26.2 (CH_2), 25.6 (CH_2), 25.4 (CH_2).**HRMS (ESI)** m/z 252.1598 $[M+H]^+$ $C_{14}H_{22}NO_3^+$ requires 252.1594.

Synthesis of compound 3aa at 1 mmol scale.

To a solution of the benzaldehyde (127.4 mg, 1.2 mmol, 1.2 equiv.) and catalyst **VII** (58.1 mg, 0.1 mmol, 0.1 equiv.) in *m*-xylene (10.0 mL), at room temperature, isocyano ester **2a** (125 mg, 1.0 mmol, 1.0 equiv.) and silver oxide (5.8 mg, 0.025 mmol, 0.025 equiv.) were added, and the reaction was stirred for 3 h. After that time, the crude was purified by column chromatography eluting with hexane: EtOAc (9:1 to 8:2) to give 150.4 mg (65%) of compound **3aa.** in 88% *ee*) of **3aa**. Enantiomeric excess (88%) was measured by HPLC (CHIRALPAK® IC), hexane:iPrOH 80:20, 1.0 mL/min, minor enantiomer: tr = 26.1min, major enantiomer: tr = 39.5 min.

Synthetic transformations and characterization of compounds 4-6

Reduction of 3aa to give methyl 3-((4*R*,5*R*)-5-phenyl-4,5-dihydrooxazol-4-yl)propanoate (4)

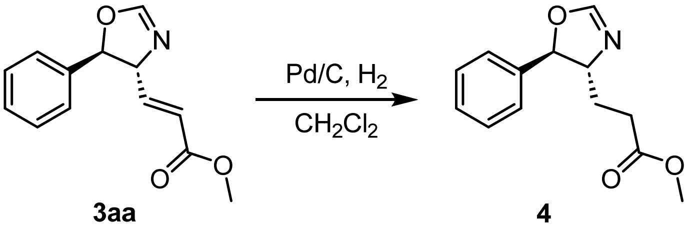

3aa **4**

16.4 mg (0.074 mmol) of methyl (*E*)-3-((4*R*,5*R*)-5-phenyl-4,5-dihydrooxazol-4-yl)acrylate (**3aa**) were dissolved in THF (2 mL). 10 mg of 5% Pd/C were added and the reaction was stirred under a H_2 atmosphere for 1 h. The reaction mixture was diluted with 10 mL of methanol and filtered through a layer of Celite. The solvent was removed under reduced pressure to give 12.2 mg (70%) of **4** after column chromatography eluting with hexane:EtOAc 5:5. Enantiomeric excess (75%) was measured by HPLC (CHIRALPAK® OD-H), hexane:iPrOH 95:5, 1.0 mL/min, minor enantiomer: tr = 44.6 min, major enantiomer: tr = 51.5 min. Pale yellow oil; $[\alpha]_D^{25}$ -112.1 (c 0.40, $CHCl_3$); **^{1}H NMR** (300 MHz, $CDCl_3$): δ = 8.11 (s, 1H, N=CHO), 7.32 – 7.26 (m, 2H, Ar), 7.24– 7.16 (m, 3H, Ar), 5.69 (d, *J* = 9.0 Hz, 1H, CH), 4.27 (ddt, *J* = 13.1, 10.2, 4.5 Hz, 1H, CH), 3.65 (s, 3H, CH_3), 2.82 (dd, *J* = 8.9, 6.6 Hz, 2H, CH_2), 2.37 (td, *J* = 7.5, 1.9 Hz, 2H, CH_2). **^{13}C NMR** (75 MHz, $CDCl_3$): δ = 174.1 (C=O), 161.0 (N=CHO), 137.4 (C), 129.5 (Ar), 128.6 (Ar), 126.8 (Ar), 51.8 (CH_3), 49.1 (CH), 41.2 (CH), 30.9 (CH_2), 28.9 (CH_2). **HRMS (ESI)** m/z 234.1131 $[M+H]^+$ $C_{13}H_{16}NO_3^+$ requires 234.1125.

Acidic hydrolysis of compounds 3 to give hydroxyformamides 5

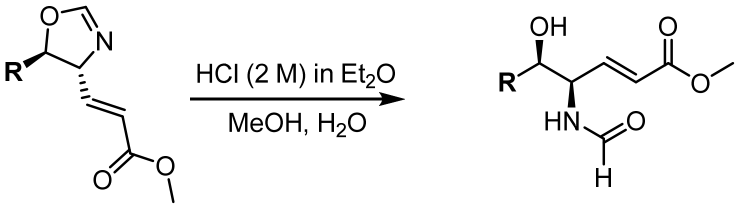

3aa, R = Ph **5aa**, R = Ph
3ra, R = Cy **5ra**, R = Cy

Methyl (4*R*,5*R*)-4-formamido-5-hydroxy-5-phenylpentanoate (5aa)

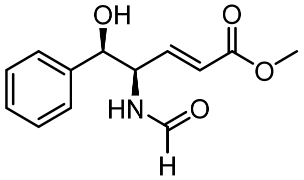

17.2 mg (0.074 mmol) of **3aa** were dissolved in 1 mL of MeOH. 2 drops of H_2O and 6 drops of HCl (2 M) solution in Et_2O were added. The reaction mixture was stirred for 4 h. Then, the mixture was basified with saturated aqueous $NaHCO_3$ (1 mL), water was added (10 mL), phases were separated with EtOAc (3 × 20 mL), washed with brine (20 mL) and dried over $MgSO_4$. The crude was concentrated under reduced pressure and purified by column chromatography eluting with hexane:EtOAc 8:2 to give 15.4 mg (82%) of **5**. Enantiomeric excess (91%) was measured by HPLC (CHIRALPAK® AD-H), hexane:iPrOH 90:10, 1.0 mL/min, major enantiomer: tr = 12.7 min, minor enantiomer: tr = 21.2 min.

Pale yellow oil; $[\alpha]_D^{25}$ -80.3 (c 0.37, $CHCl_3$); **^{1}H NMR** (300 MHz, $CDCl_3$): δ = 8.13 (d, *J* = 1.6 Hz, 1H, CHO), 7.39 – 7.29 (m, 5H, Ar), 7.03 (dd, *J* = 15.8, 4.8 Hz, 1H, CH), 6.10 – 6.08 (m, 1H, NH), 5.99 (dd, *J* = 15.7, 1.5 Hz, 1H, CH), 4.96 (d, *J* = 3.3 Hz, 2H, OH, CH), 3.75 (d, *J* = 3.7 Hz, 1H, CH), 3.74 (s, 3H, CH_3).**^{13}C NMR** (75 MHz, $CDCl_3$): δ = 166.4 (C=O), 160.8 (C=O), 144.7 (C), 139.8 (CH), 128.7 (Ar), 128.4 (Ar), 125.9 (Ar), 122.7 (CH), 74.2 (CH), 54.7 (CH), 51.8 (CH_3). **HRMS (ESI)** m/z 272.0890 $[M+Na]^+$ $C_{13}H_{15}NNaO_4^+$ requires 272.0893.

Methyl (4*R*,5*R*)-4-formamido-5-hydroxy-5-cyclohexyl (5ra)

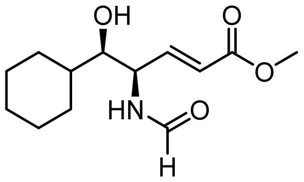

Following the same procedure as for the synthesis of **5aa**, 23.8 mg (95%) of **5ra** were obtained from 3ra (23.2 mg, 0.098 mmol). Enantiomeric excess (91%) was measured by HPLC (CHIRALPAK® AD-H), hexane:iPrOH 90:10, 1.0 mL/min, major enantiomer: tr = 8.1 min, minor enantiomer: tr = 16.4 min.

Yellow oil; $[\alpha]_D^{25}$ 61.6 (c 0.48, $CHCl_3$); **^{1}H NMR** (400 MHz, $CDCl_3$): δ = 8.25 (d, *J* = 1.7 Hz, 1H, CHO), 6.91 (dd, J = 15.7, 5.0 Hz, 1H, CH), 6.53 (d, J = 9.0 Hz, 1H, OH), 5.96 (dd, J = 15.7, 1.8 Hz, 1H, CH), 4.86 (t, J = 7.6 Hz, 1H, NH), 3.71 (s, 3H, CH_3), 3.44 (d, J = 8.4 Hz, 1H, CH), 2.88 (unres. d, 1H, CH), 1.95 (d, J = 12.8 Hz, 1H, CH), 1.80-1.60 (m, 4H), 1.43-1.35 (m, 1H), 1.23-1.09 (m, 3H), 0.99-0.91 (m, 2H).**^{13}C NMR** (100 MHz, $CDCl_3$): δ = 166.8 (C=O), 161.2 (CH=O), 147.0 (CH), 122.1 (CH), 77.0 (CH), 51.9 (CH), 50.3 (CH3), 40.3 (CH), 29.2 (CH_2), 29.0 (CH_2), 26.3 (CH_2), 25,9 (CH_2), 25.8 (CH_2). **HRMS (ESI)** m/z 256.1548 $[M+H]^+$ $C_{13}H_{22}NO_4^+$ requires 256.1543.

Hydrolysis of 3aa to give methyl (*R*)-5-hydroxy-4-oxo-5-phenylpentanoate (6)

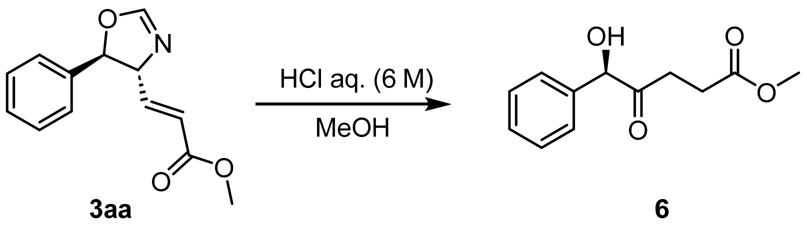

17.1 mg (0.074 mmol) of **3aa** were dissolved in 1 mL of MeOH. 6 drops of aqueous HCl (6 M) solution were added. The reaction mixture was stirred at rt for 2 h. Then, the mixture was basified with saturated aqueous $NaHCO_3$ (1 mL), water was added (10 mL), phases were separated with EtOAc (3 × 20 mL), washed with brine (20 mL) and dried over $MgSO_4$. The crude was concentrated under reduced pressure and purified by column chromatography eluting with hexane:EtOAc 8:2 to give 12.5 mg (75%) of **6**. Enantiomeric excess (89%) was measured by HPLC (CHIRALPAK® AD-H), hexane:iPrOH 90:10, 1.0 mL/min, minor enantiomer: tr = 14.0 min, major enantiomer: tr = 14.8 min. Pale yellow oil; $[\alpha]_D^{25}$ -194.7 (c 0.49, $CHCl_3$); **^{1}H NMR** (300 MHz, $CDCl_3$): δ = 7.42 – 7.31 (m, 5H, Ar), 5.16 (s, 1H, CH), 3.65 (s, 3H, CH_3), 2.76 – 2.44 (m, 4H, $2CH_2$). **^{13}C NMR** (75 MHz, $CDCl_3$): δ = 208.2 (C=O), 172.8 (C=O), 138.0 (C), 129.2 (Ar), 129.0 (Ar), 127.6 (Ar), 79.9 (CH), 52.1 (CH_3), 32.8 (CH_2), 27.9 (CH_2). Characterization data matched with those reported in literature.[5]

^{1}H and ^{13}C NMR spectra

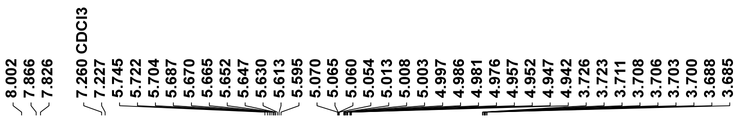

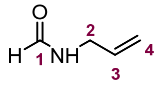

S2

^{1}H NMR ($CDCl_3$, 300 MHz)

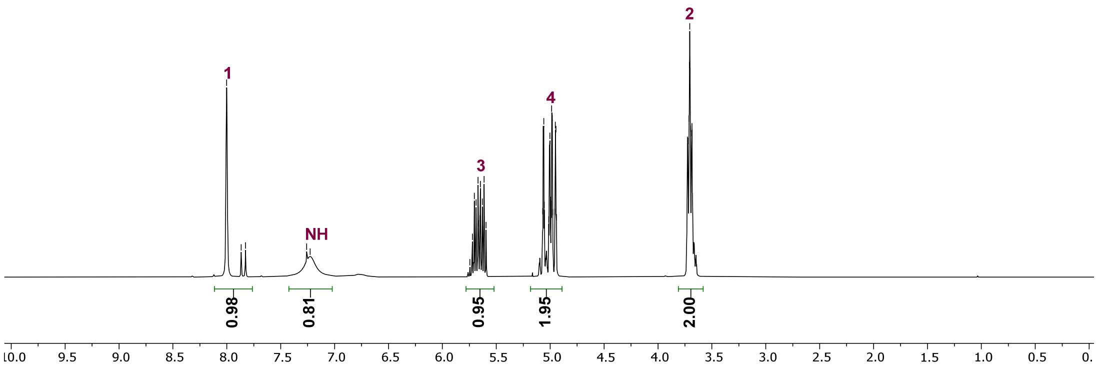

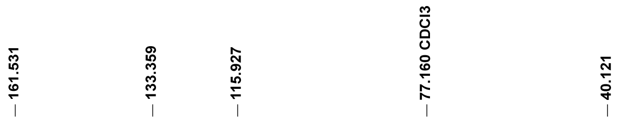

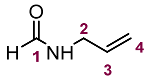

S2

^{13}C NMR ($CDCl_3$, 75 MHz)

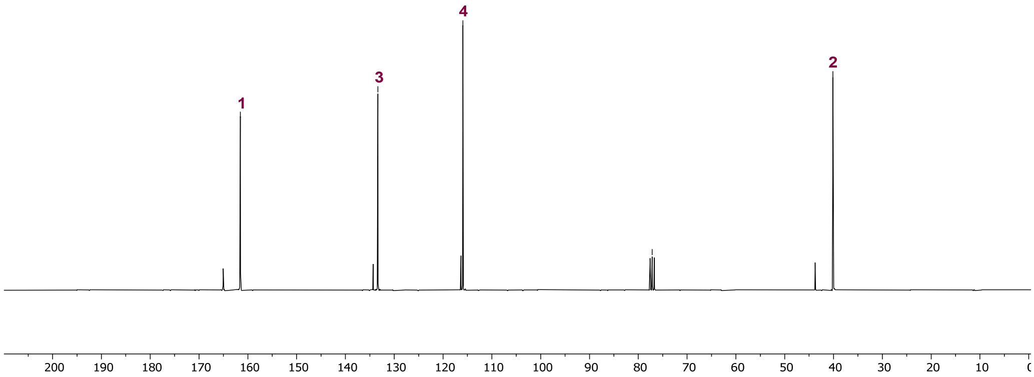

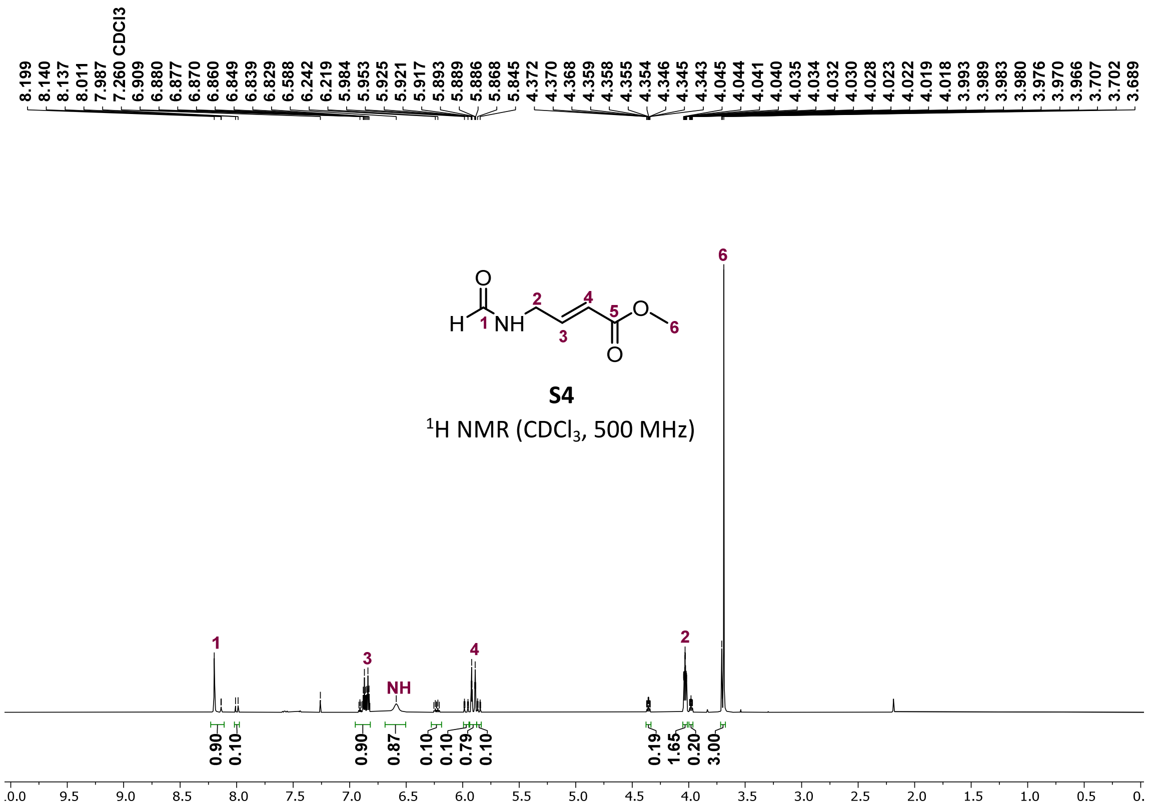

S4

^{1}H NMR ($CDCl_3$, 500 MHz)

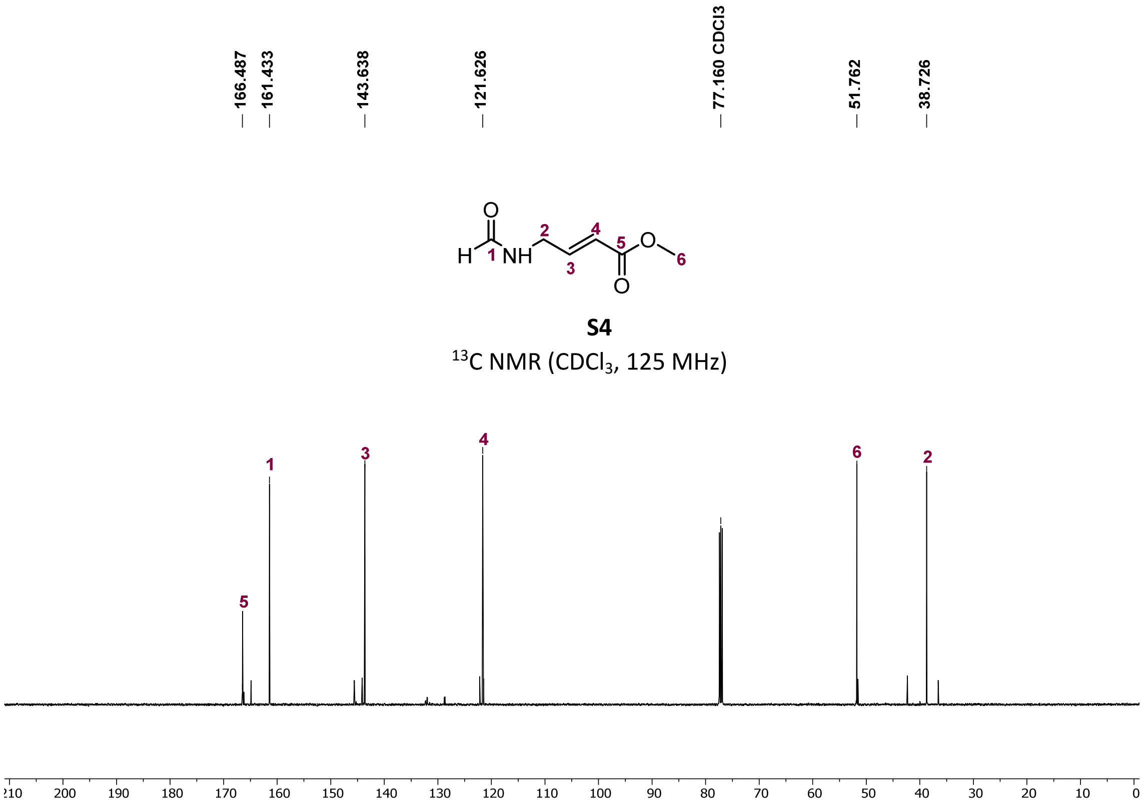

S4

^{13}C NMR ($CDCl_3$, 125 MHz)

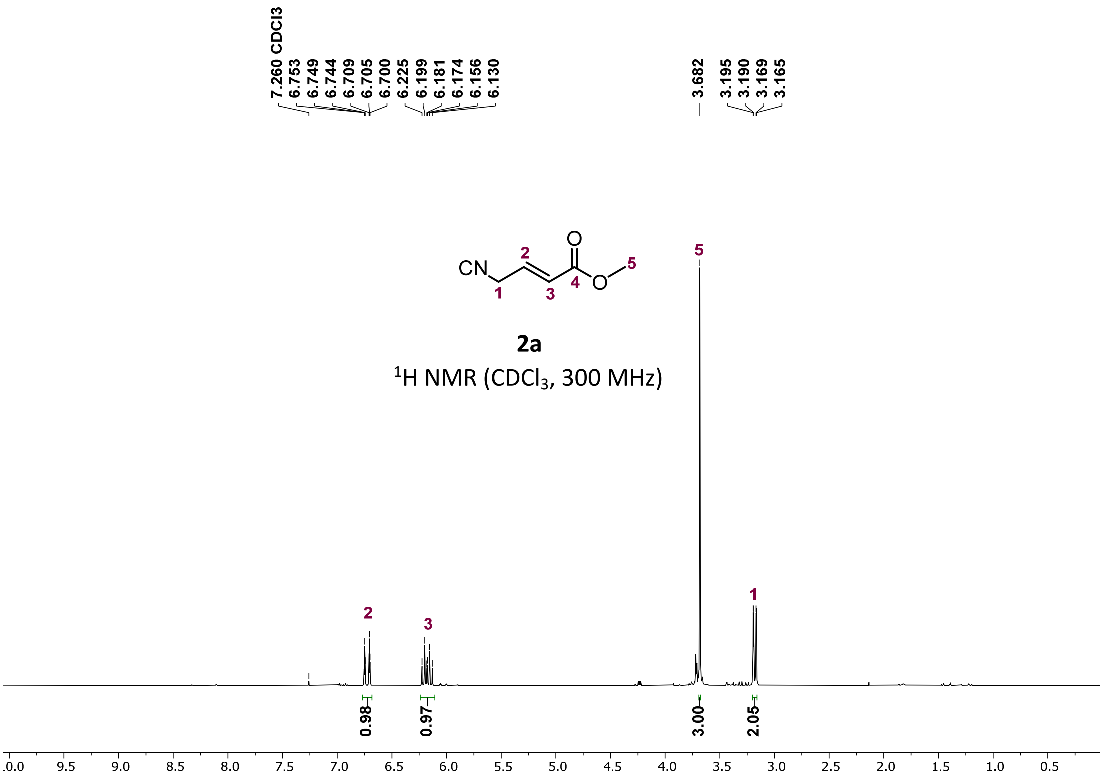
7.260 CDCl3
6.753
6.749
6.744
6.709
6.705
6.700
6.225
6.199
6.181
6.174
6.156
6.130
3.682
3.195
3.190
3.169
3.165
CN
O
2a
^{1}H NMR ($CDCl_3$, 300 MHz)
0.98
0.97
3.00
2.05

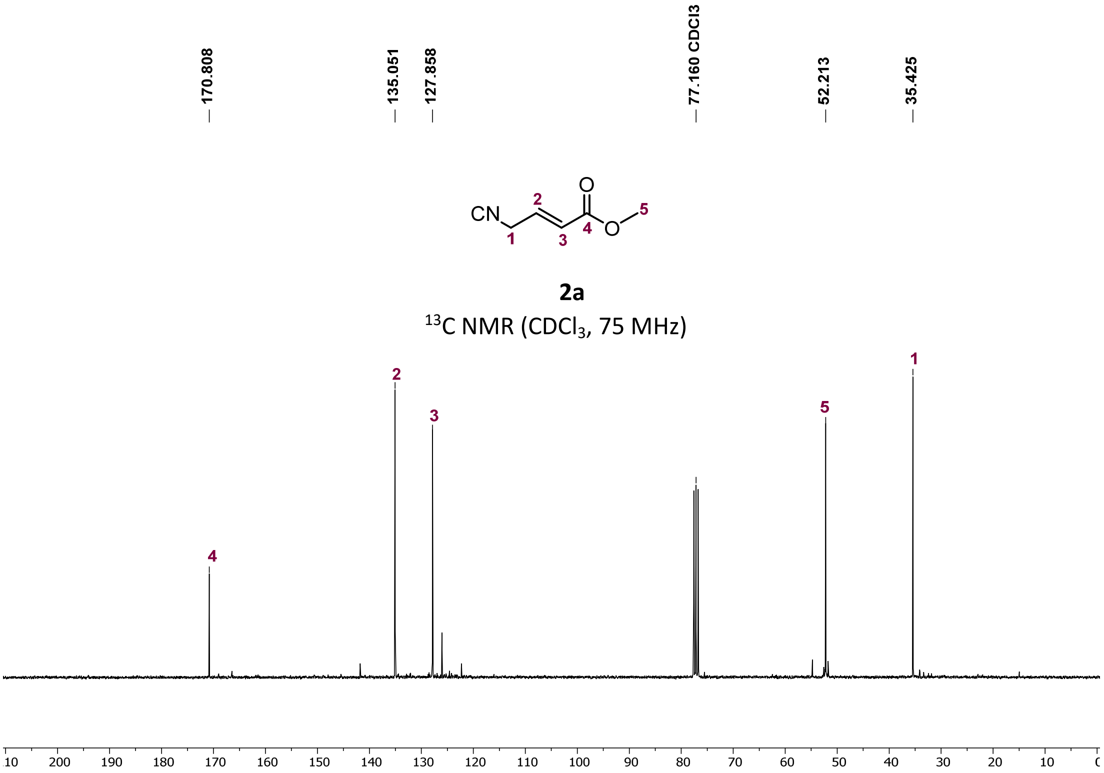
170.808
135.051
127.858
77.160 CDCl3
52.213
35.425
CN
O
2a
^{13}C NMR ($CDCl_3$, 75 MHz)

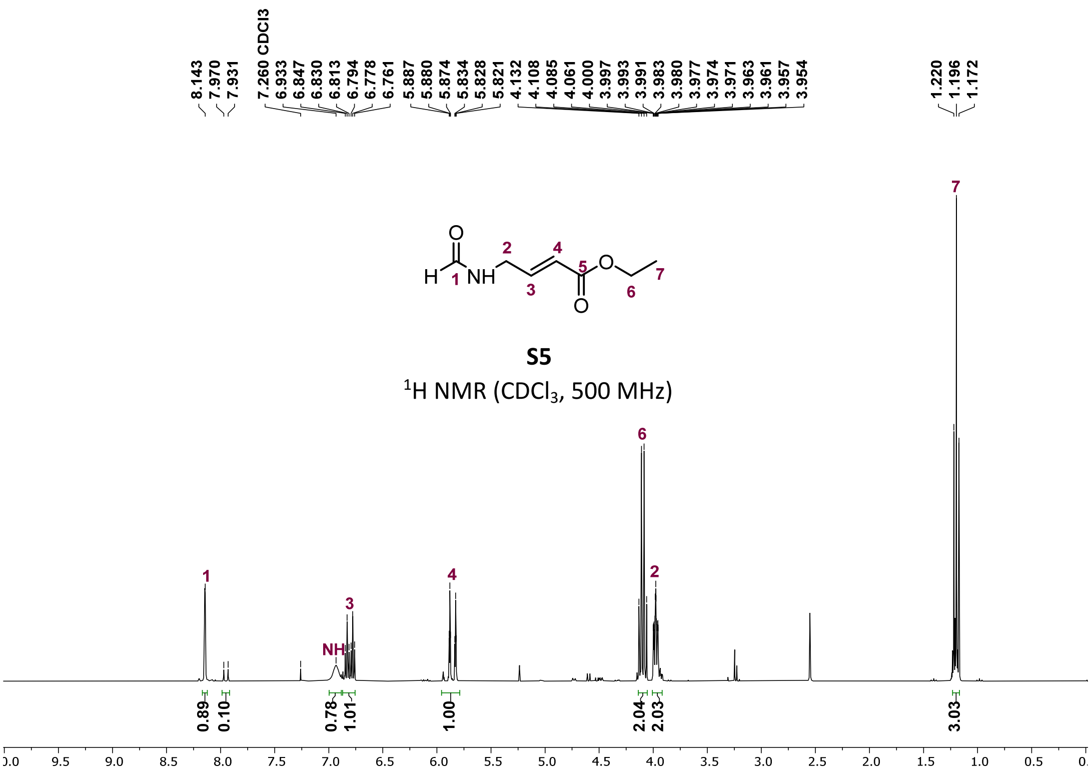

S5

^{1}H NMR ($CDCl_3$, 500 MHz)

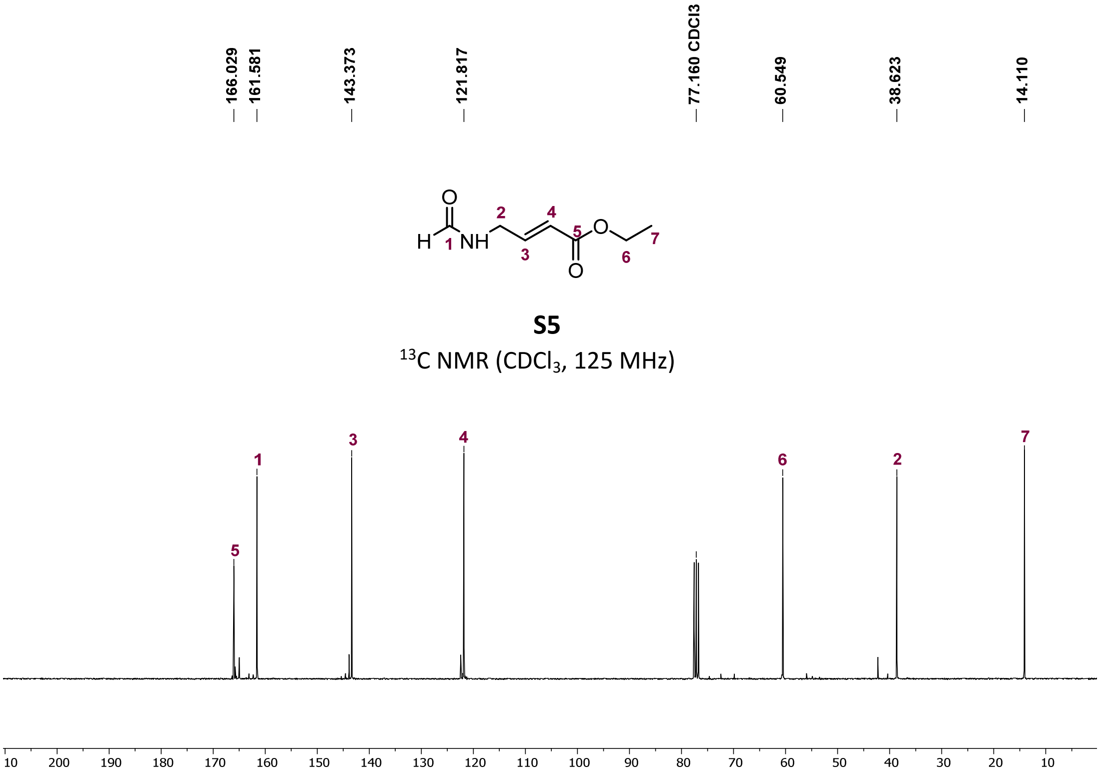

S5

^{13}C NMR ($CDCl_3$, 125 MHz)

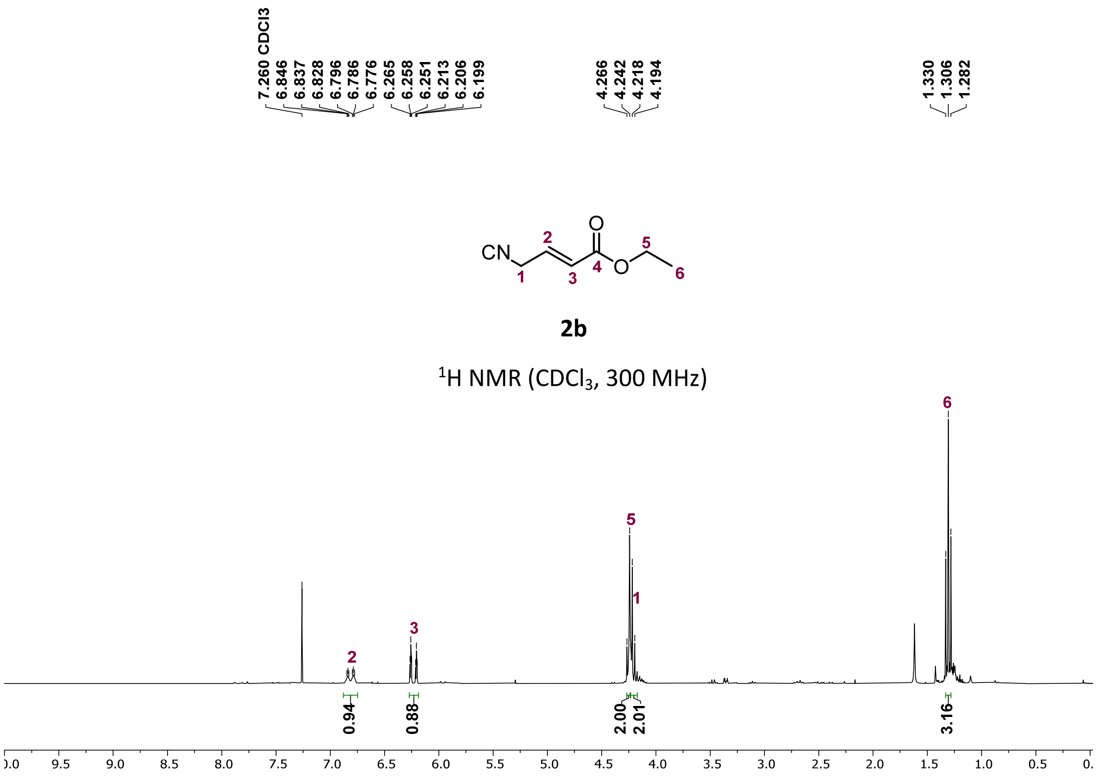
7.260 CDCl3
6.846
6.837
6.828
6.796
6.786
6.776
6.265
6.258
6.251
6.213
6.206
6.199
4.266
4.242
4.218
4.194
1.330
1.306
1.282
CN
O
O
2b
1H NMR (CDCl3, 300 MHz)
0.94
0.88
2.00
2.01
3.16

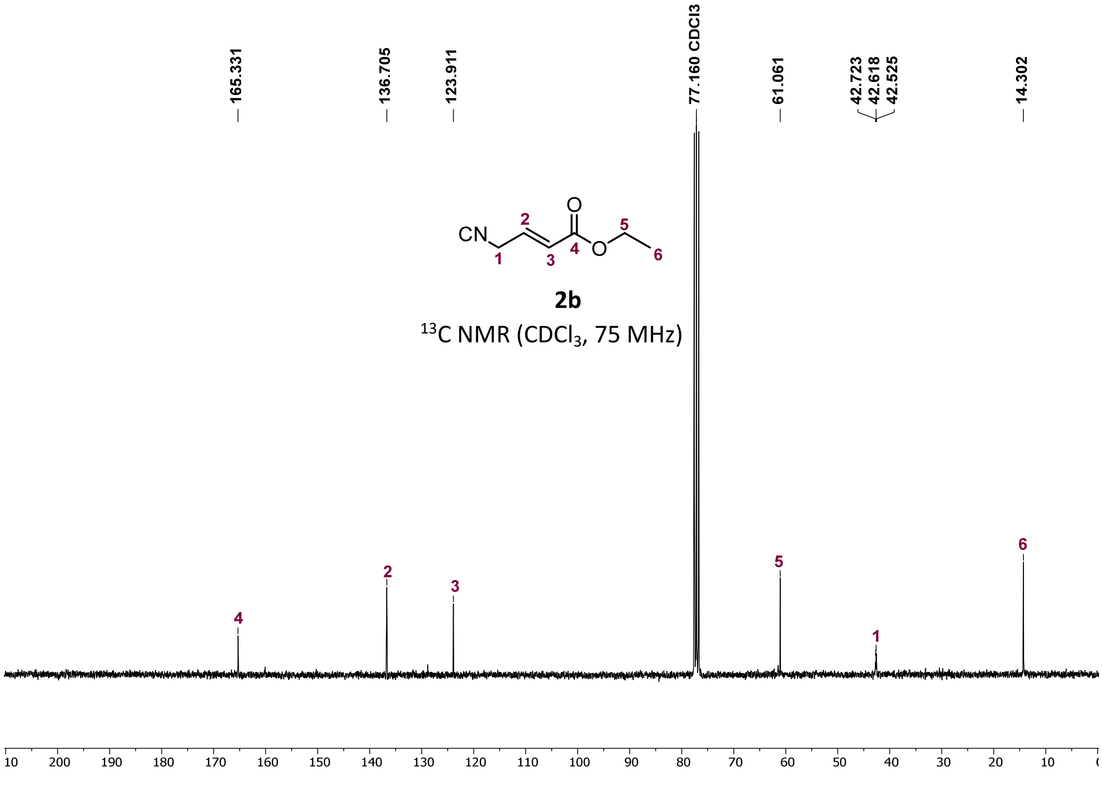
165.331
136.705
123.911
77.160 CDCl3
61.061
42.723
42.618
42.525
14.302
CN
O
O
2b
13C NMR (CDCl3, 75 MHz)

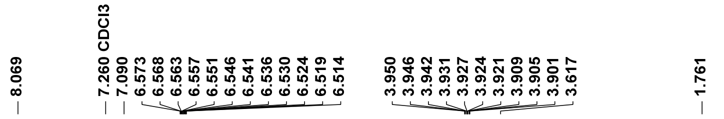

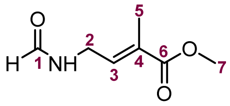

S6

^{1}H NMR ($CDCl_3$, 300 MHz)

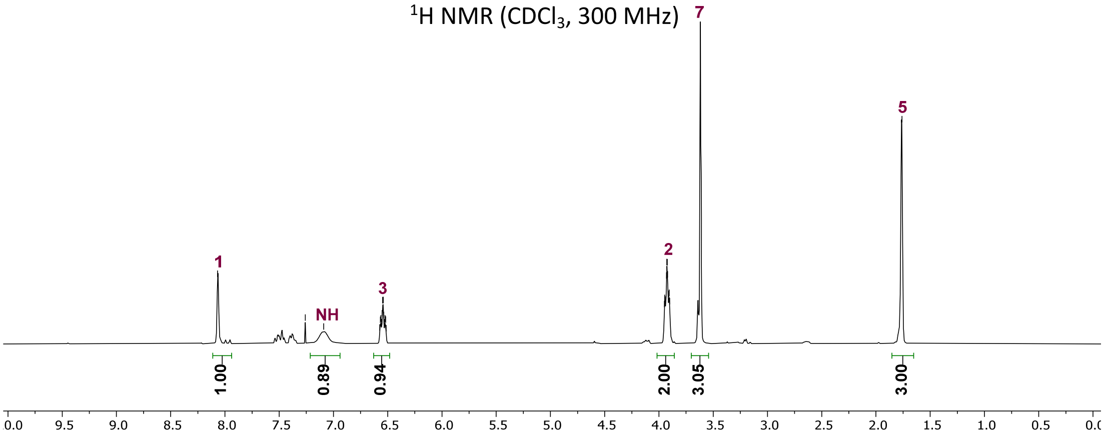

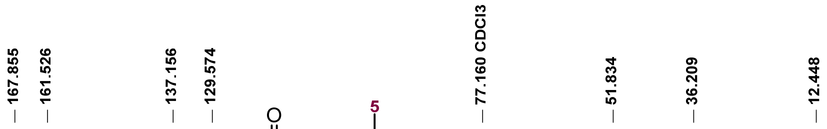

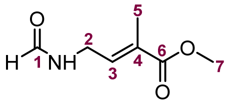

S6

^{13}C NMR ($CDCl_3$, 75 MHz)

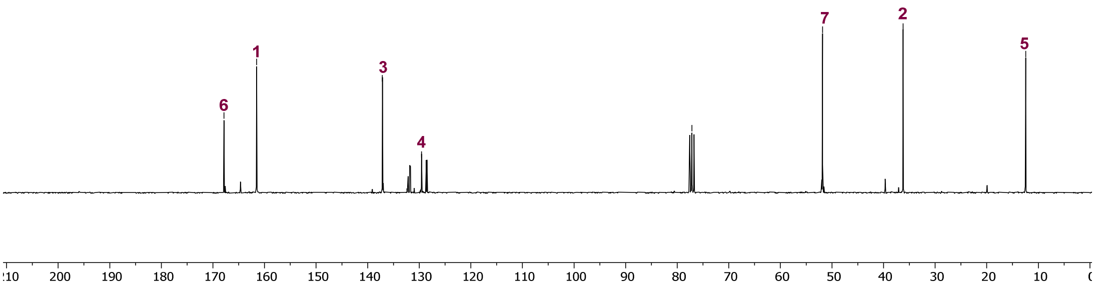

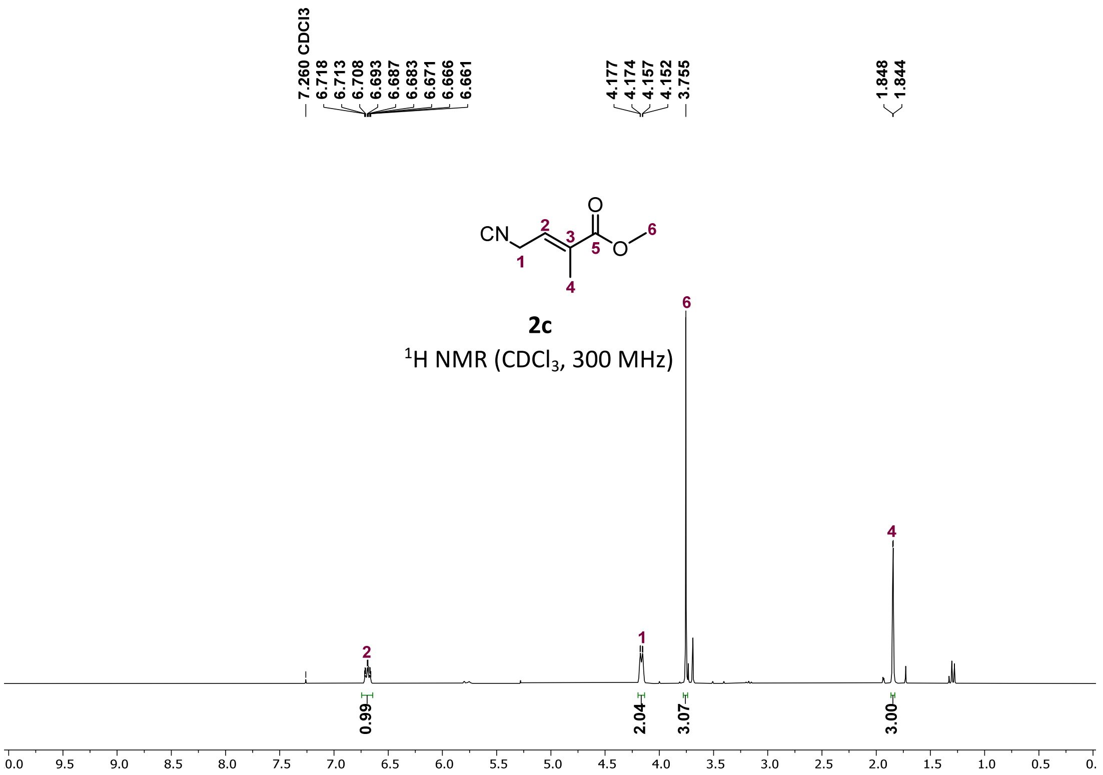
7.260 CDCl3
6.718
6.713
6.708
6.693
6.687
6.683
6.671
6.666
6.661
4.177
4.174
4.157
4.152
3.755
1.848
1.844
CN
O
O
2c
1H NMR (CDCl3, 300 MHz)
0.99
2.04
3.07
3.00

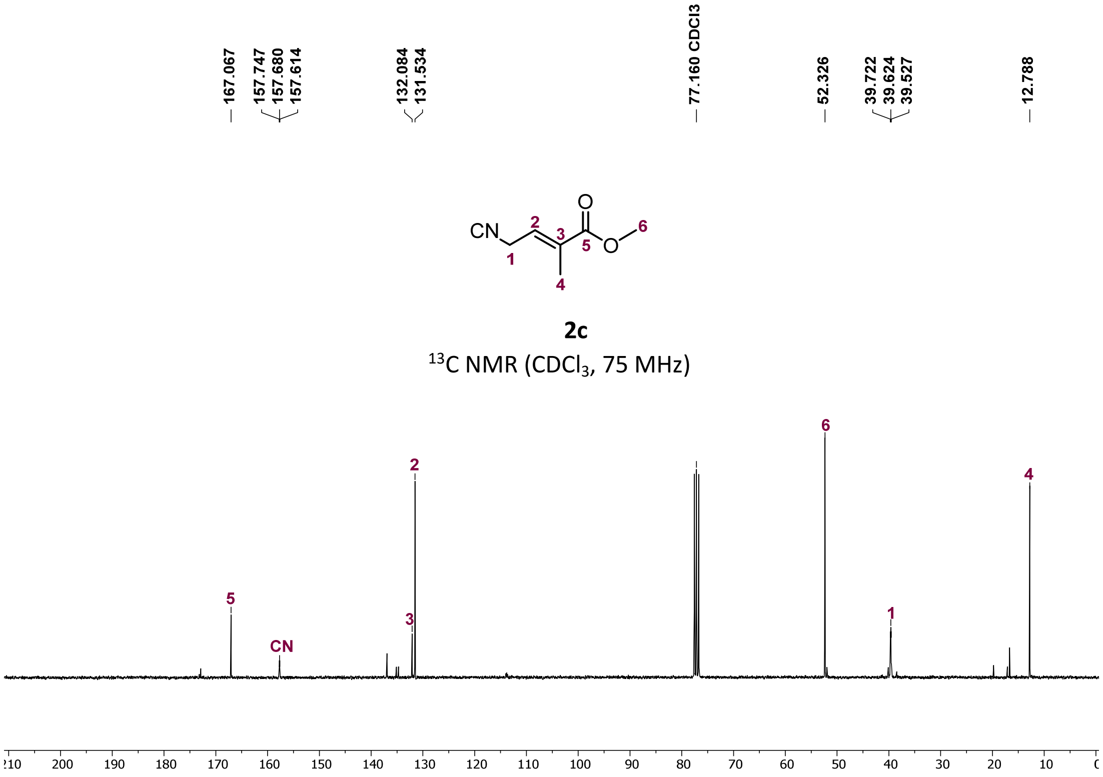
167.067
157.747
157.680
157.614
132.084
131.534
77.160 CDCl3
52.326
39.722
39.624
39.527
12.788
CN
O
O
2c
13C NMR (CDCl3, 75 MHz)
CN

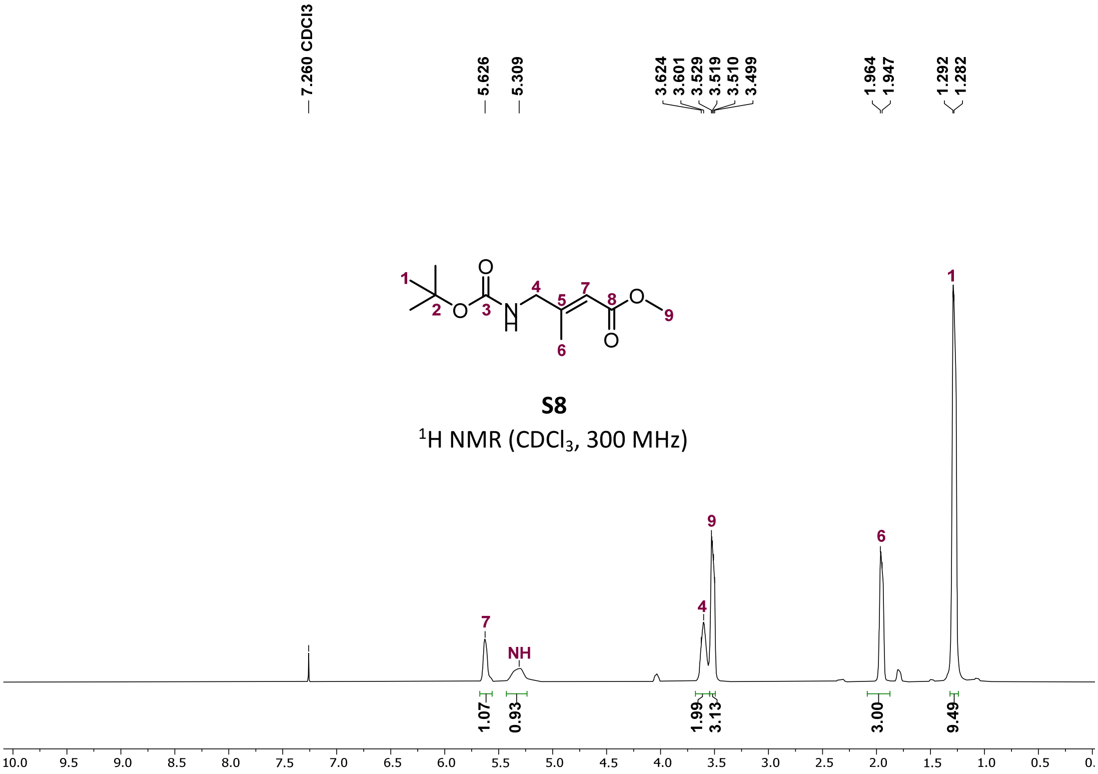
7.260 CDCl3
5.626
5.309
3.624
3.601
3.529
3.519
3.510
3.499
1.964
1.947
1.292
1.282
S8
^{1}H NMR ($CDCl_3$, 300 MHz)
NH
1.07
0.93
1.99
3.13
3.00
9.49

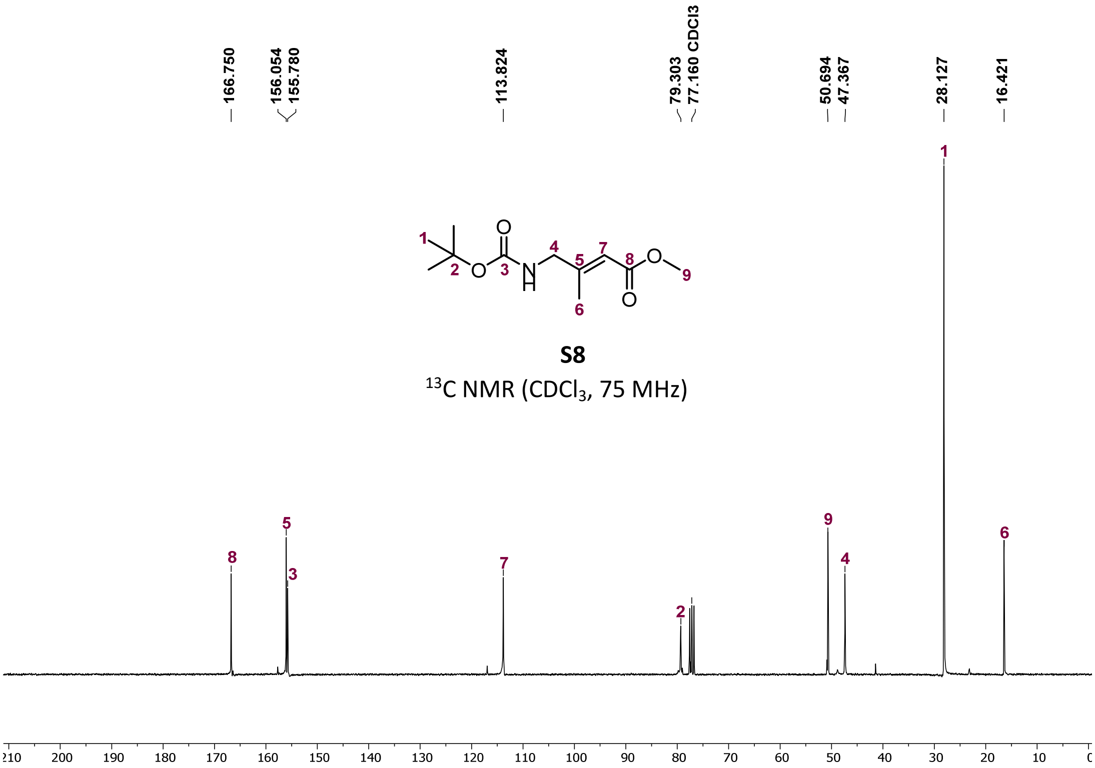
166.750
156.054
155.780
113.824
79.303
77.160 CDCl3
50.694
47.367
28.127
16.421
S8
^{13}C NMR ($CDCl_3$, 75 MHz)

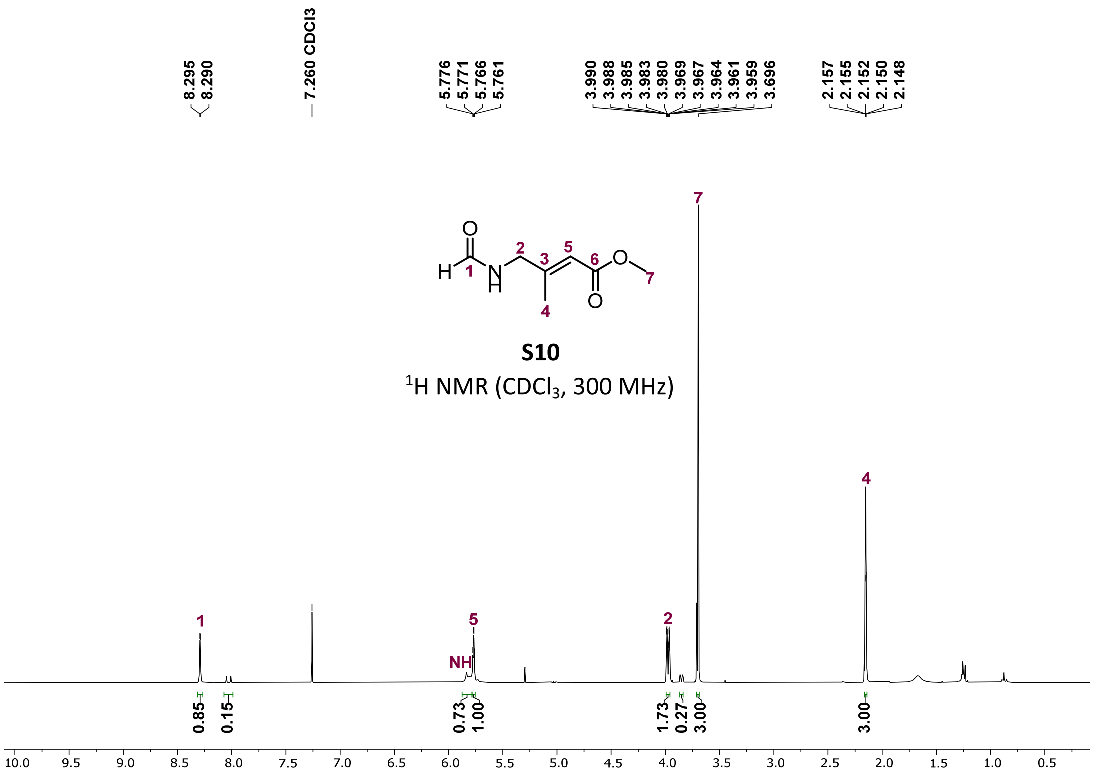

8.295
8.290
7.260 CDCl3
5.776
5.771
5.766
5.761
3.990
3.988
3.985
3.983
3.980
3.969
3.967
3.964
3.961
3.959
3.696
2.157
2.155
2.152
2.150
2.148
S10
^{1}H NMR ($CDCl_3$, 300 MHz)
NH
0.85
0.15
0.73
1.00
1.73
0.27
3.00
3.00
10.0 9.5 9.0 8.5 8.0 7.5 7.0 6.5 6.0 5.5 5.0 4.5 4.0 3.5 3.0 2.5 2.0 1.5 1.0 0.5

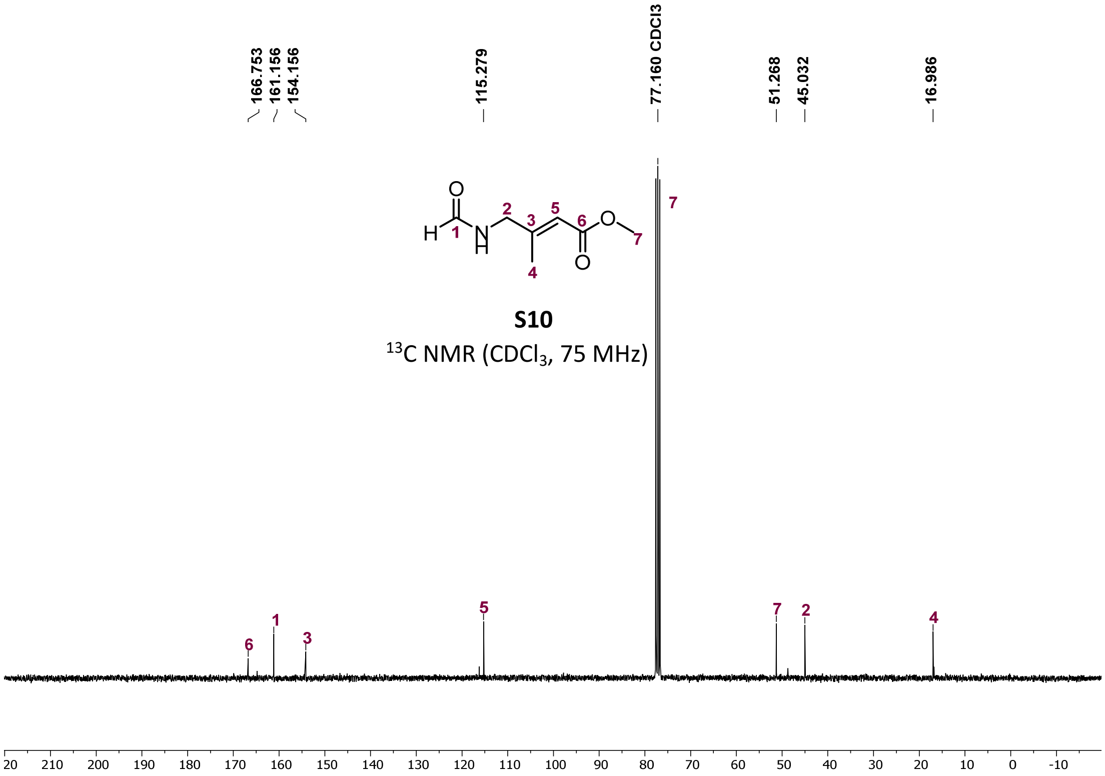

166.753
161.156
154.156
115.279
77.160 CDCl3
51.268
45.032
16.986
S10
^{13}C NMR ($CDCl_3$, 75 MHz)
20 210 200 190 180 170 160 150 140 130 120 110 100 90 80 70 60 50 40 30 20 10 0 -10

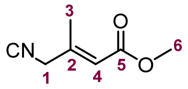

2d

^{1}H NMR ($CDCl_3$, 300 MHz)

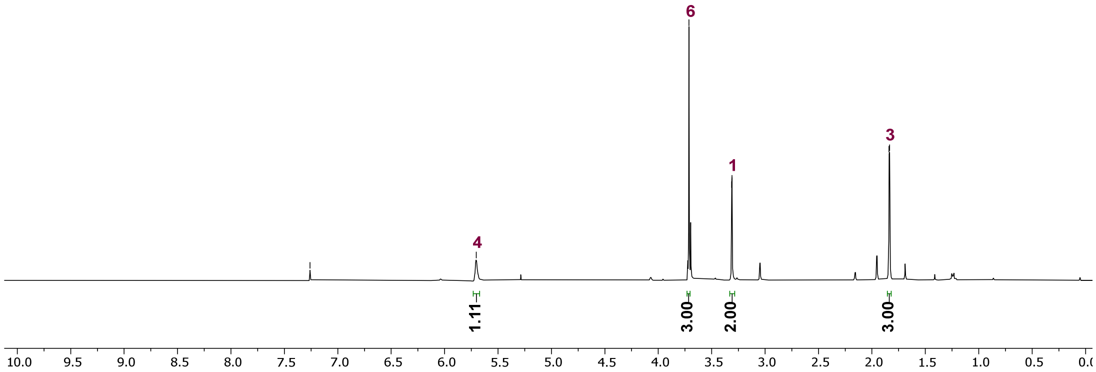

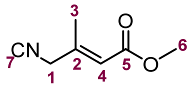

2d

^{13}C NMR ($CDCl_3$, 75 MHz)

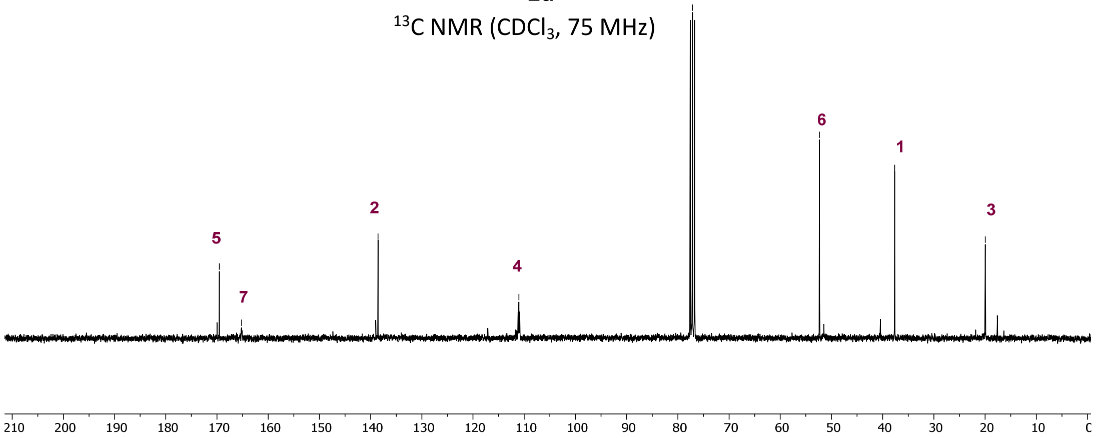

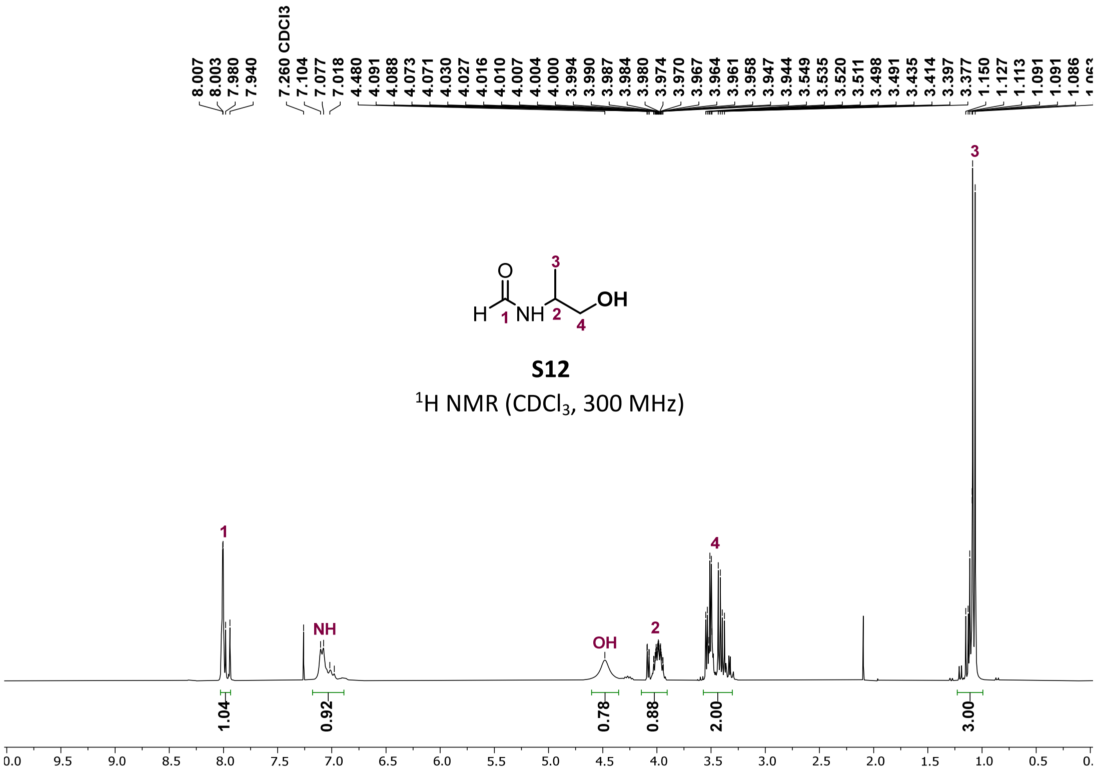
8.007
8.003
7.980
7.940
7.260 CDCl3
7.104
7.077
7.018
4.480
4.091
4.088
4.073
4.071
4.030
4.027
4.016
4.010
4.007
4.004
4.000
3.994
3.990
3.987
3.984
3.980
3.974
3.970
3.967
3.964
3.961
3.958
3.947
3.944
3.549
3.535
3.520
3.511
3.498
3.491
3.435
3.414
3.397
3.377
1.150
1.127
1.113
1.091
1.091
1.086
S12
1H NMR (CDCl3, 300 MHz)
1
NH
OH
2
4
3
1.04
0.92
0.78
0.88
2.00
3.00

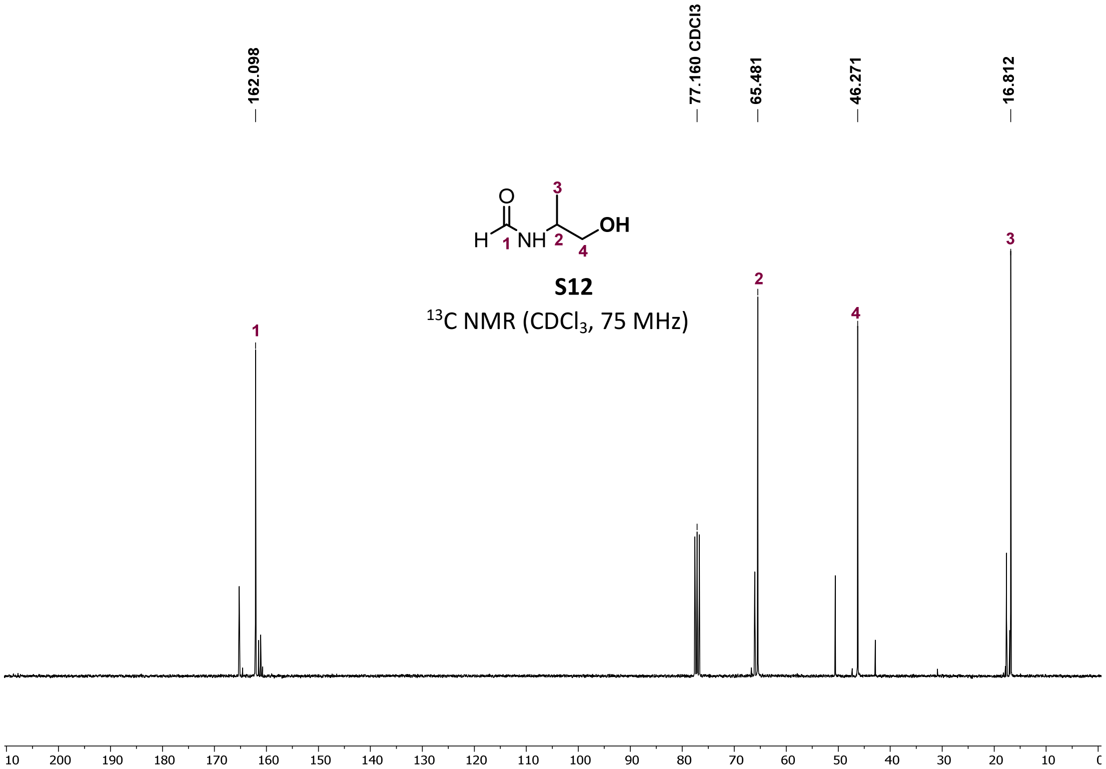
162.098
77.160 CDCl3
65.481
46.271
16.812
S12
13C NMR (CDCl3, 75 MHz)
1
2
4
3

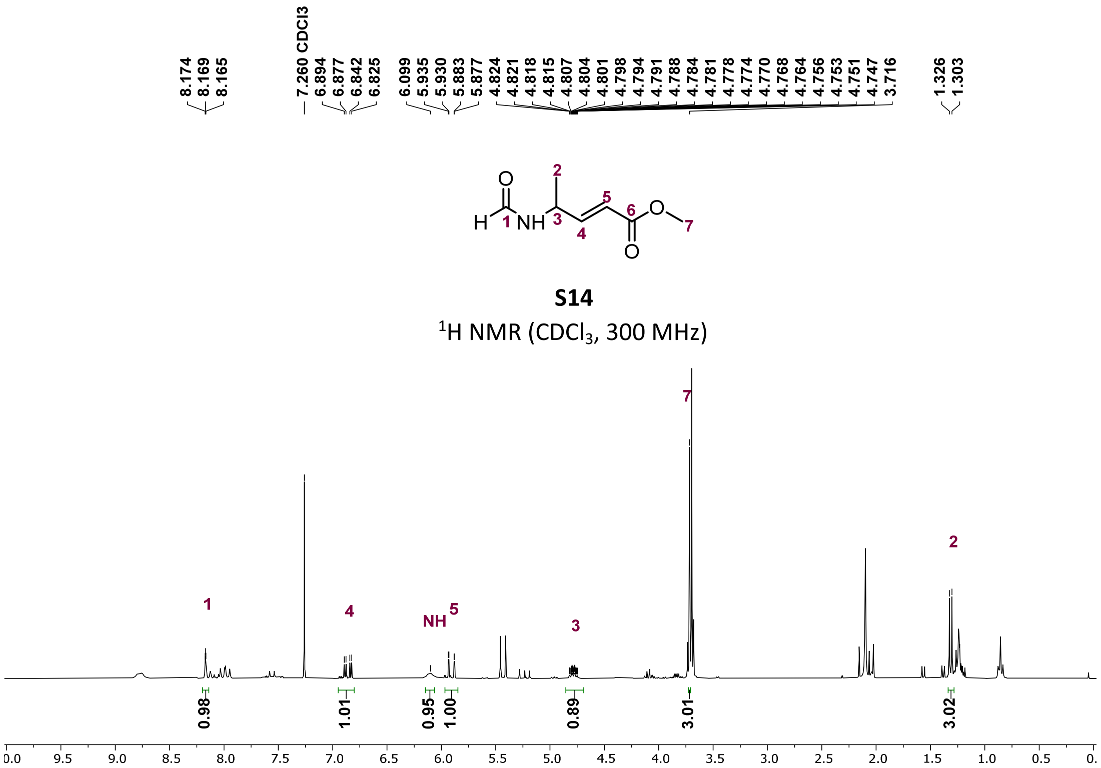

S14

^{1}H NMR ($CDCl_3$, 300 MHz)

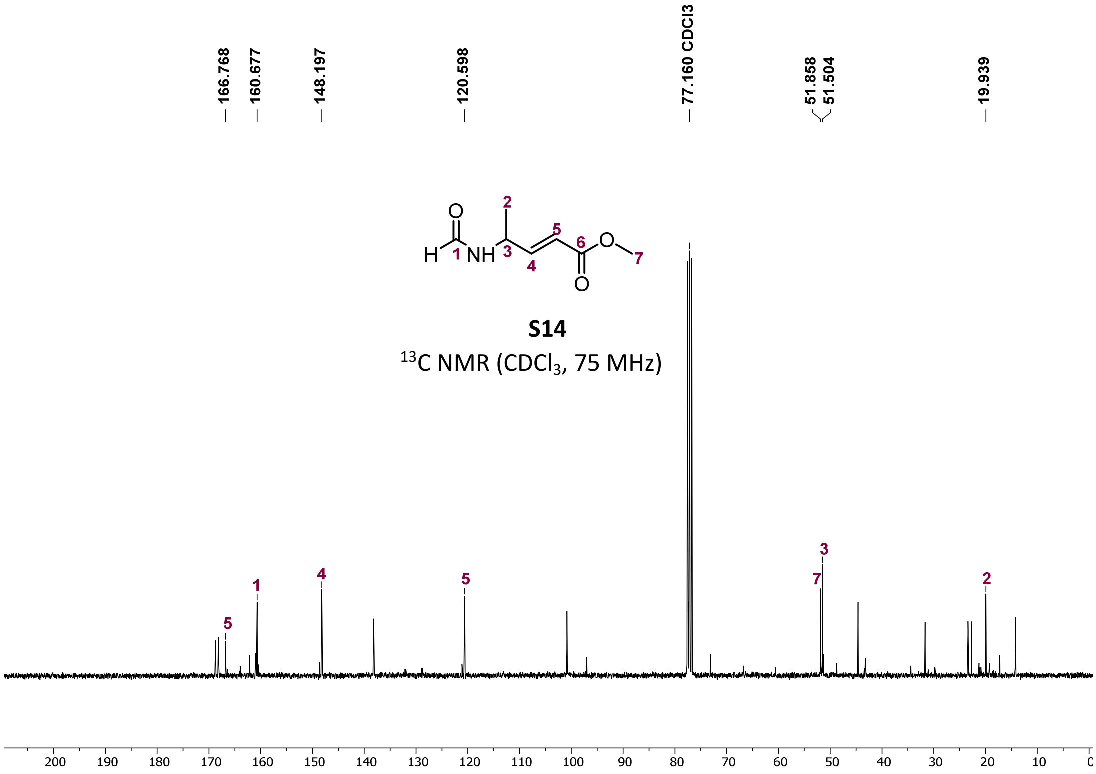

S14

^{13}C NMR ($CDCl_3$, 75 MHz)

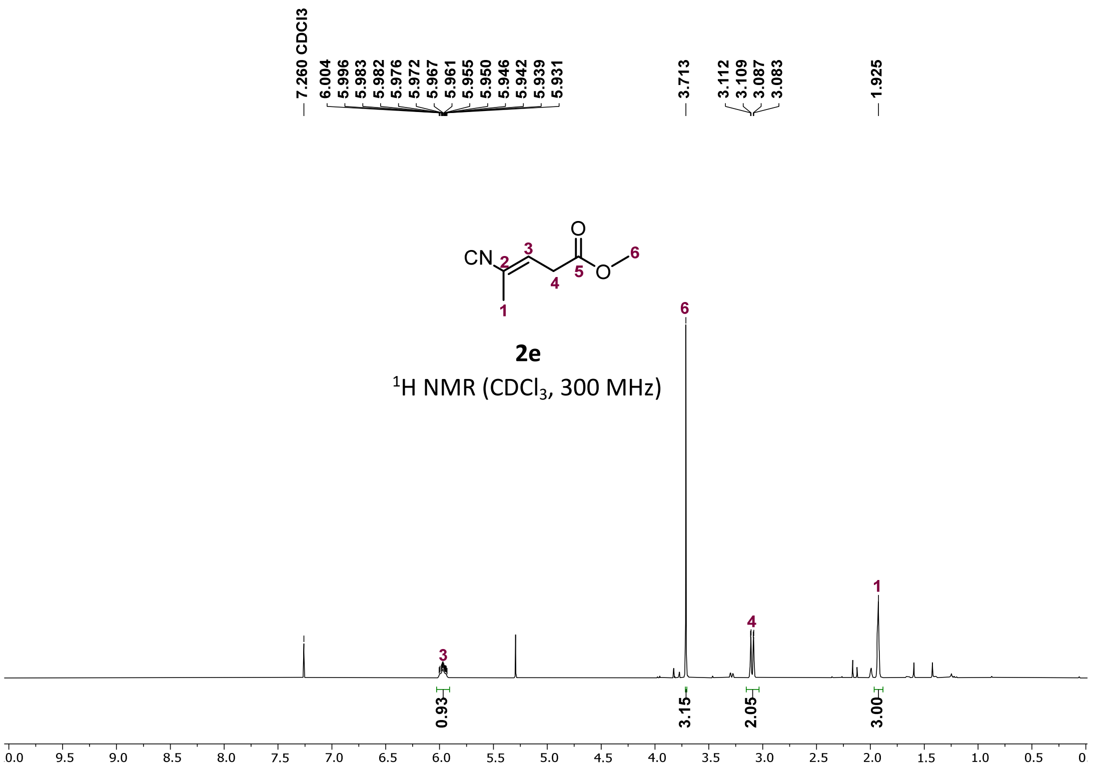
7.260 CDCl3
6.004
5.996
5.983
5.982
5.976
5.972
5.967
5.961
5.955
5.950
5.946
5.942
5.939
5.931
3.713
3.112
3.109
3.087
3.083
1.925
CN
O
O
2e
^{1}H NMR (CDCl$_3$, 300 MHz)
0.93
3.15
2.05
3.00
10.0
9.5
9.0
8.5
8.0
7.5
7.0
6.5
6.0
5.5
5.0
4.5
4.0
3.5
3.0
2.5
2.0
1.5
1.0
0.5
0

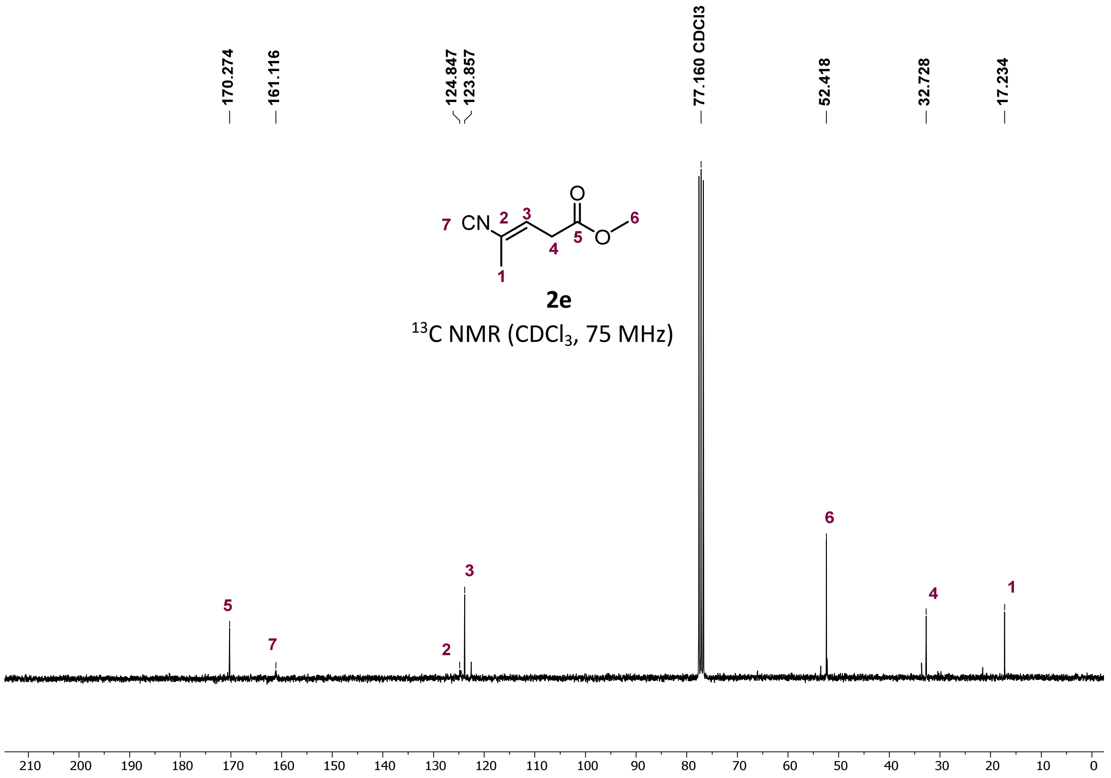
170.274
161.116
124.847
123.857
77.160 CDCl3
52.418
32.728
17.234
CN
O
O
2e
^{13}C NMR (CDCl$_3$, 75 MHz)
210
200
190
180
170
160
150
140
130
120
110
100
90
80
70
60
50
40
30
20
10
0

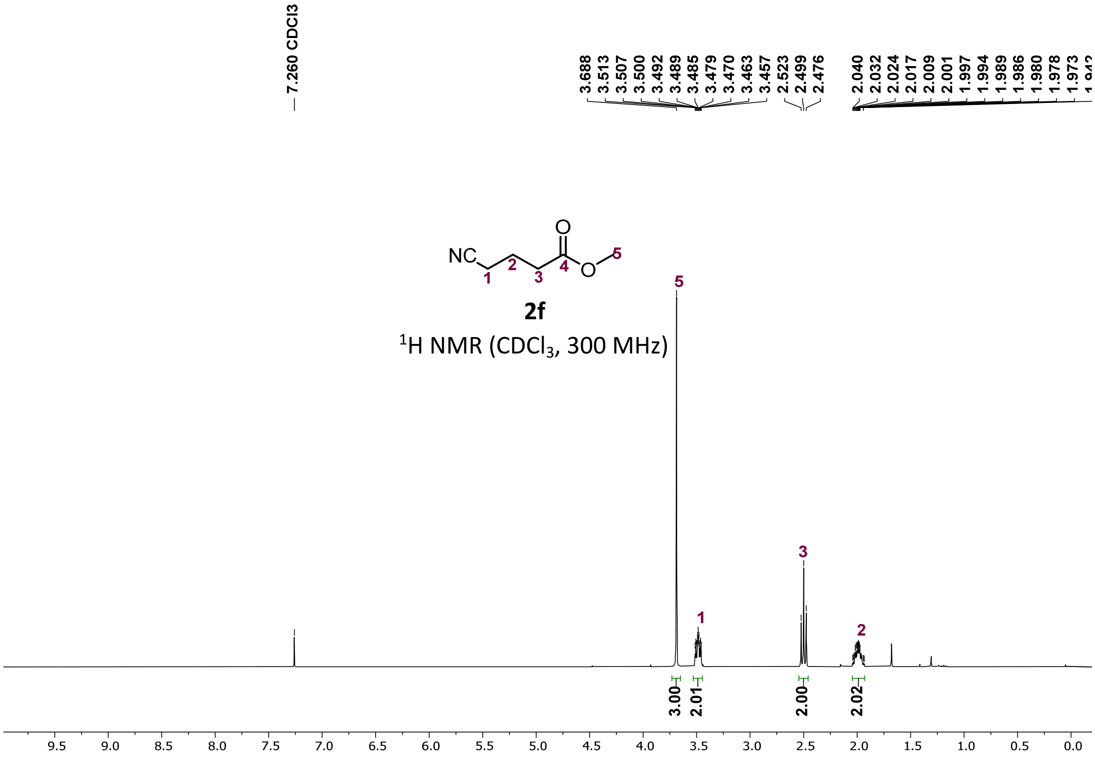

7.260 CDCl3
3.688
3.513
3.507
3.500
3.492
3.489
3.485
3.479
3.470
3.463
3.457
2.523
2.499
2.476
2.040
2.032
2.024
2.017
2.009
2.001
1.997
1.994
1.989
1.986
1.980
1.978
1.973
NC
1
2
3
4
O
O
5
2f
^{1}H NMR (CDCl$_3$, 300 MHz)
3.00
2.01
2.00
2.02
9.5
9.0
8.5
8.0
7.5
7.0
6.5
6.0
5.5
5.0
4.5
4.0
3.5
3.0
2.5
2.0
1.5
1.0
0.5
0.0

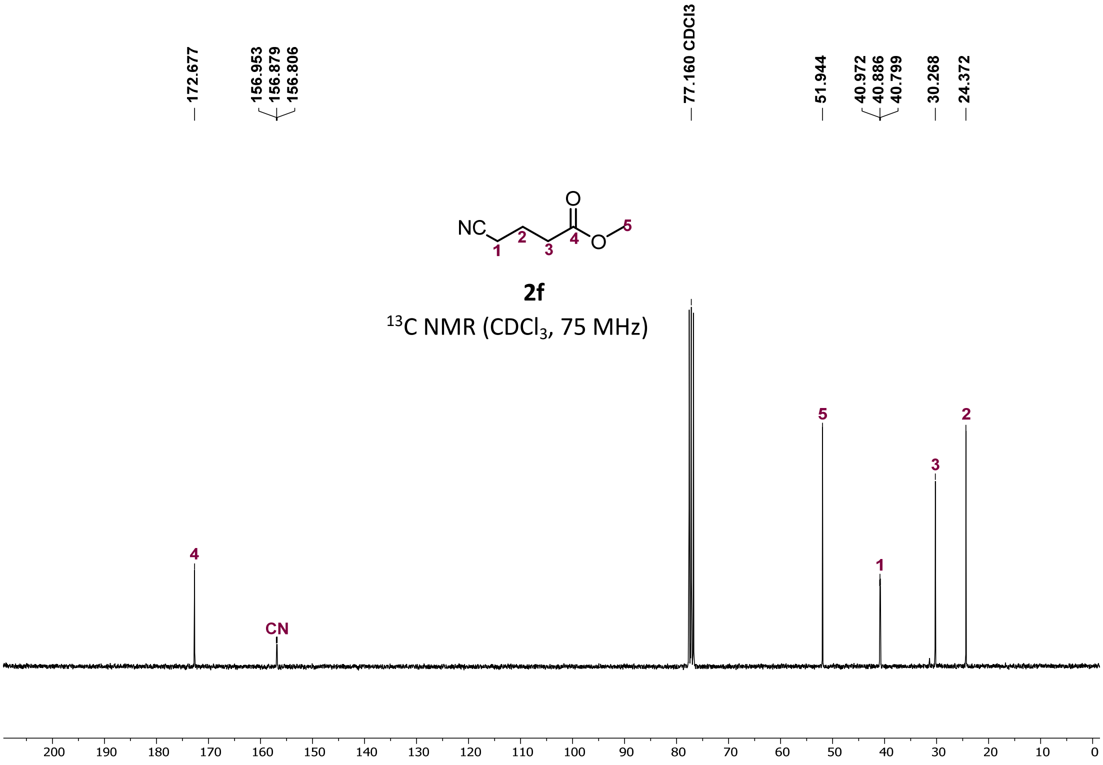

172.677
156.953
156.879
156.806
77.160 CDCl3
51.944
40.972
40.886
40.799
30.268
24.372
NC
1
2
3
4
O
O
5
2f
^{13}C NMR (CDCl$_3$, 75 MHz)
CN
200
190
180
170
160
150
140
130
120
110
100
90
80
70
60
50
40
30
20
10
0

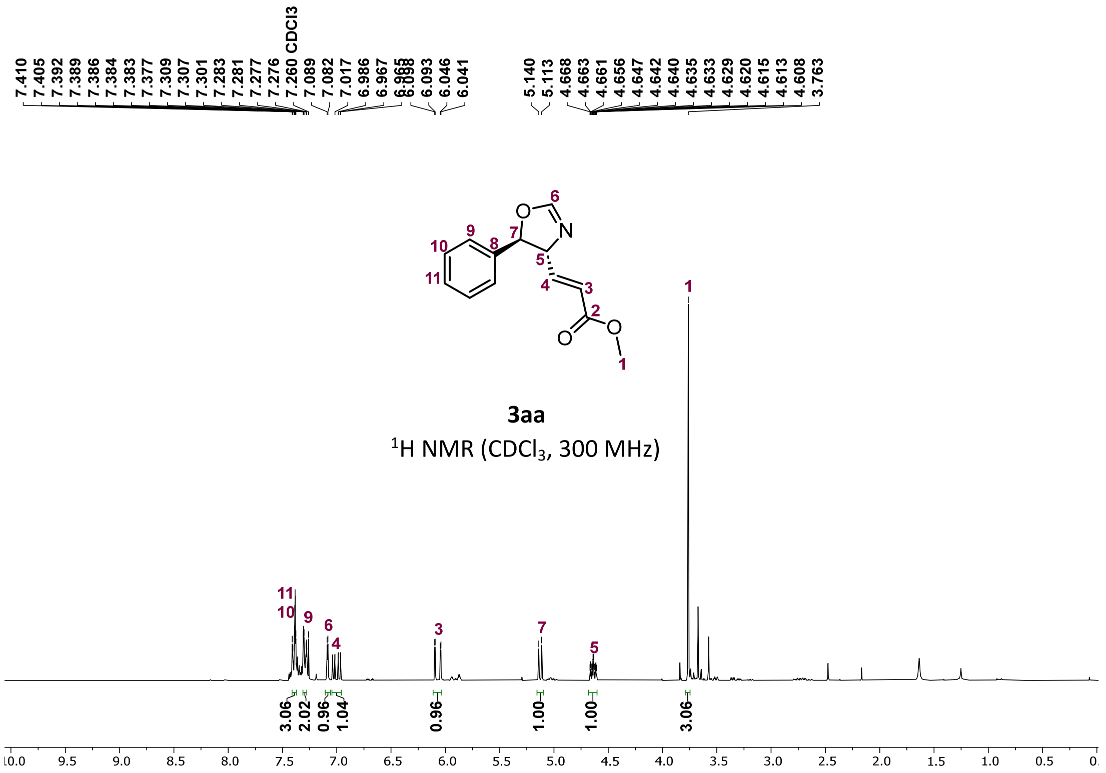

3aa

^1H NMR ($CDCl_3$, 300 MHz)

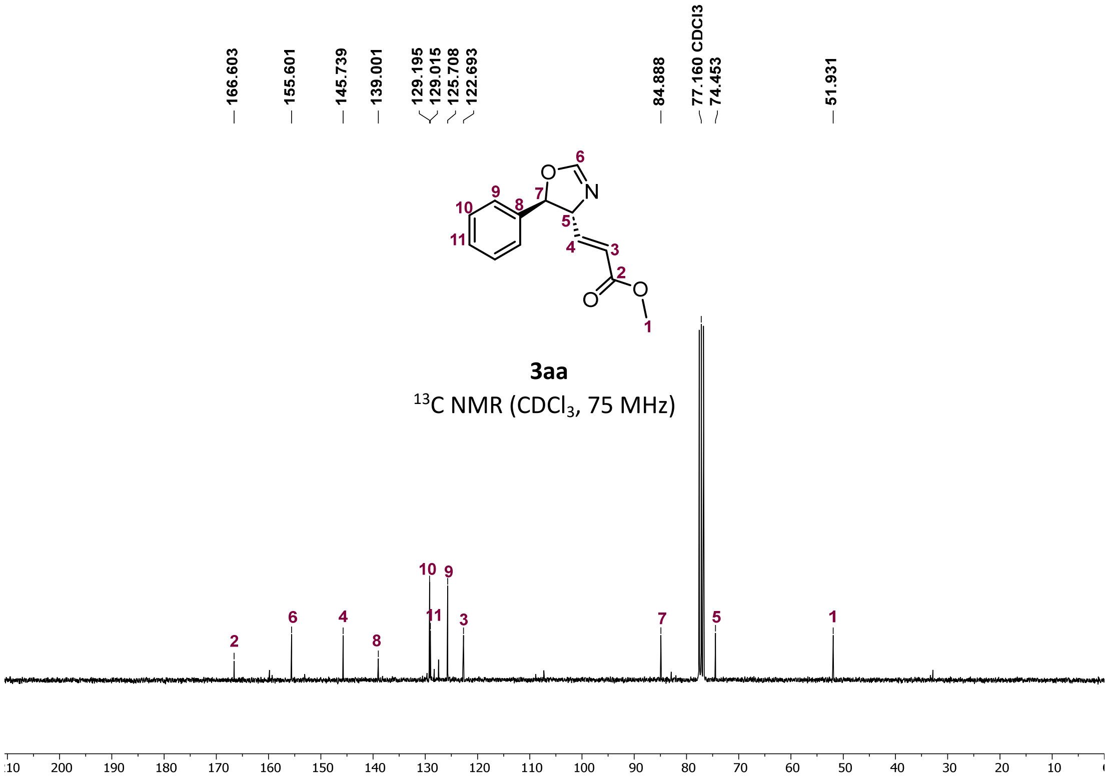

3aa

^{13}C NMR ($CDCl_3$, 75 MHz)

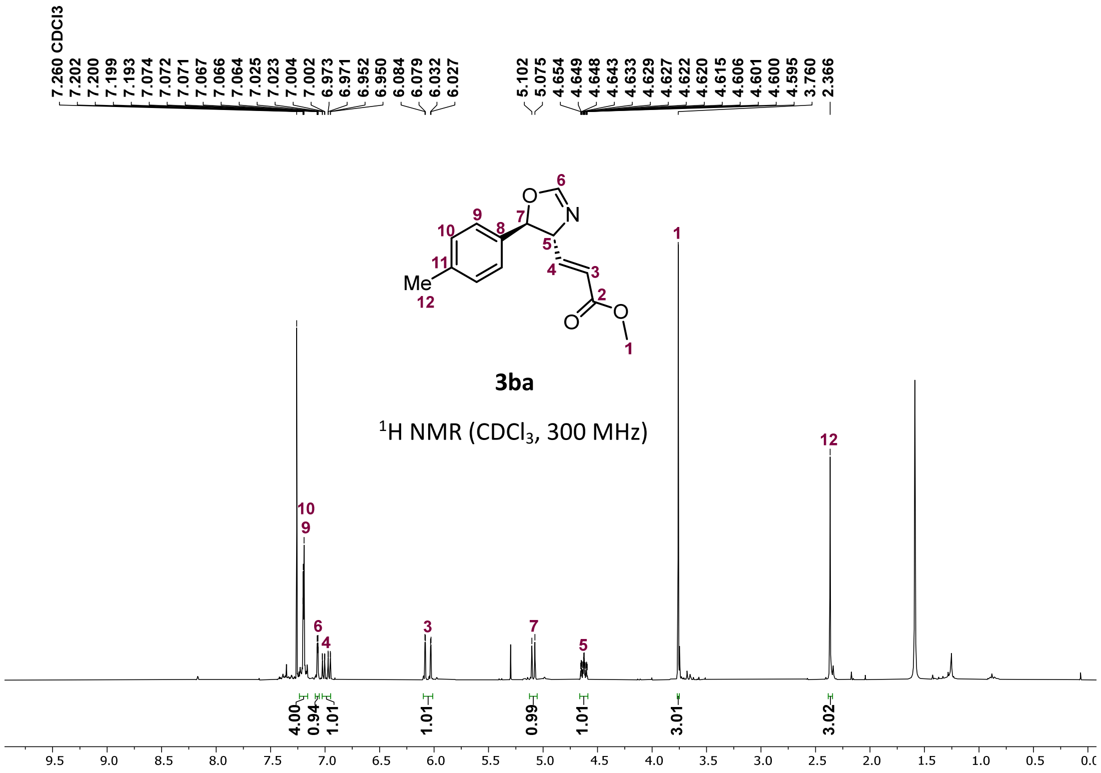
7.260 CDCl3
7.202
7.200
7.199
7.193
7.074
7.072
7.071
7.067
7.066
7.064
7.025
7.023
7.004
7.002
6.973
6.971
6.952
6.950
6.084
6.079
6.032
6.027
5.102
5.075
4.654
4.649
4.648
4.643
4.633
4.629
4.627
4.622
4.620
4.615
4.606
4.601
4.600
4.595
3.760
2.366
3ba
^{1}H NMR (CDCl$_3$, 300 MHz)
4.00
0.94
1.01
1.01
0.99
1.01
3.01
3.02

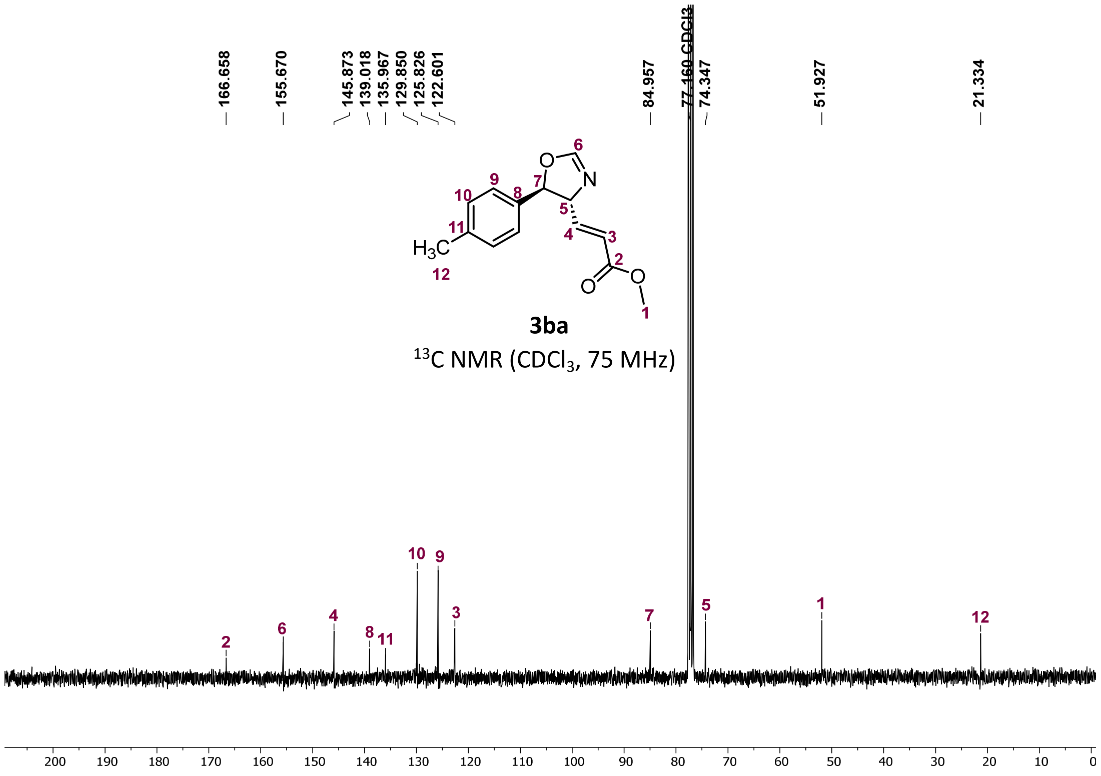
166.658
155.670
145.873
139.018
135.967
129.850
125.826
122.601
84.957
77.160 CDCl3
74.347
51.927
21.334
3ba
^{13}C NMR (CDCl$_3$, 75 MHz)

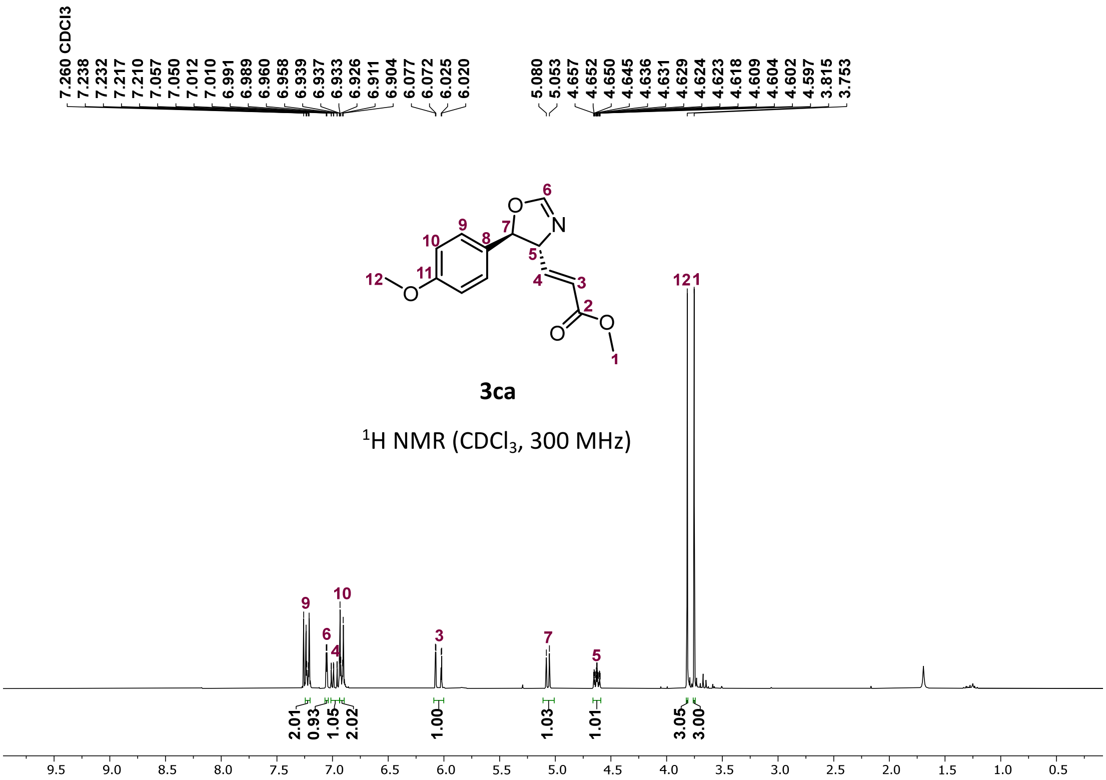
7.260 CDCl3
7.238
7.232
7.217
7.210
7.057
7.050
7.012
7.010
6.991
6.989
6.960
6.958
6.939
6.937
6.933
6.926
6.911
6.904
6.077
6.072
6.025
6.020
5.080
5.053
4.657
4.652
4.650
4.645
4.636
4.631
4.629
4.624
4.623
4.618
4.609
4.604
4.602
4.597
3.815
3.753
3ca
^{1}H NMR (CDCl$_3$, 300 MHz)
2.01
0.93
1.05
2.02
1.00
1.03
1.01
3.05
3.00
9.5
9.0
8.5
8.0
7.5
7.0
6.5
6.0
5.5
5.0
4.5
4.0
3.5
3.0
2.5
2.0
1.5
1.0
0.5

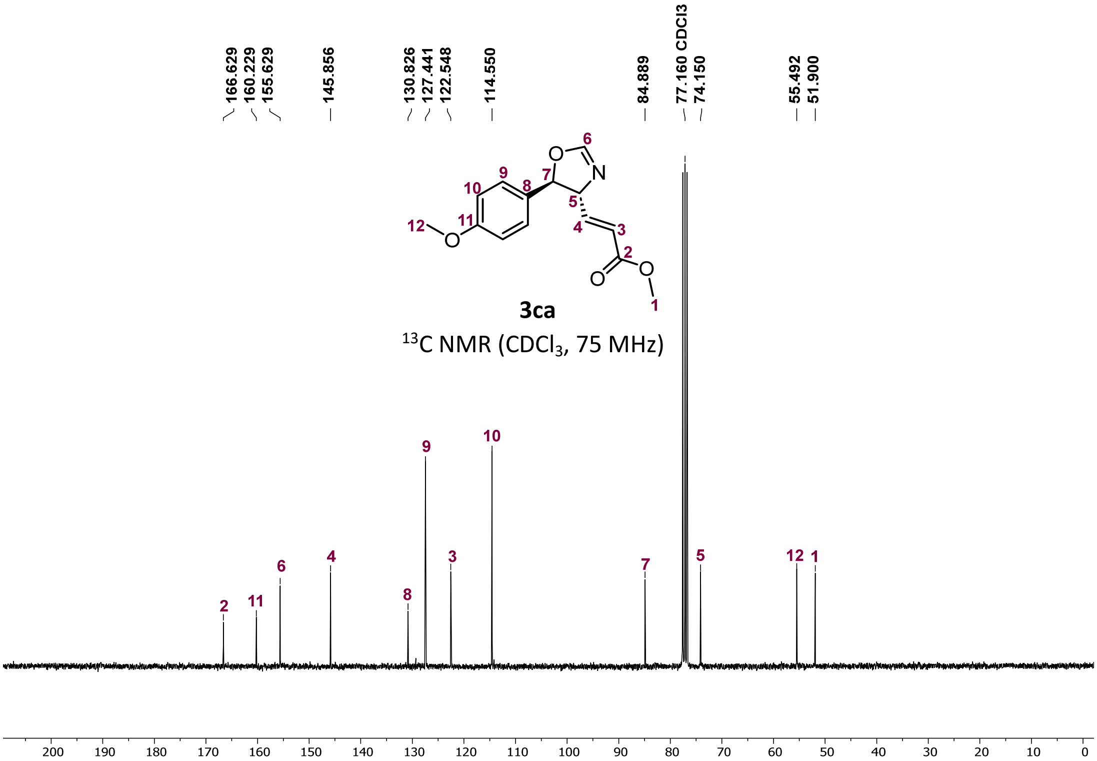
166.629
160.229
155.629
145.856
130.826
127.441
122.548
114.550
84.889
77.160 CDCl3
74.150
55.492
51.900
3ca
^{13}C NMR (CDCl$_3$, 75 MHz)
200
190
180
170
160
150
140
130
120
110
100
90
80
70
60
50
40
30
20
10
0

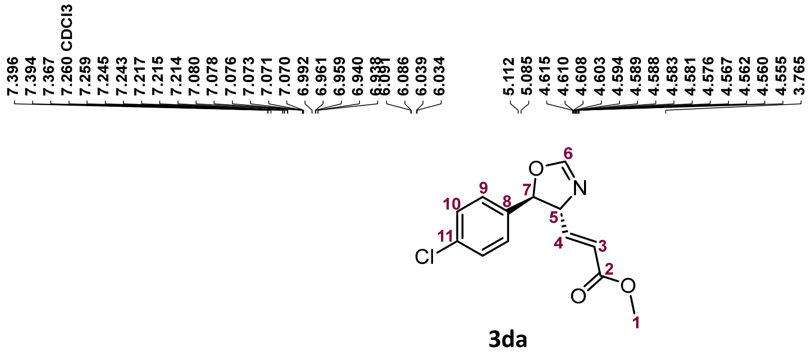

3da

^{1}H NMR (CDCl$_3$, 300 MHz)

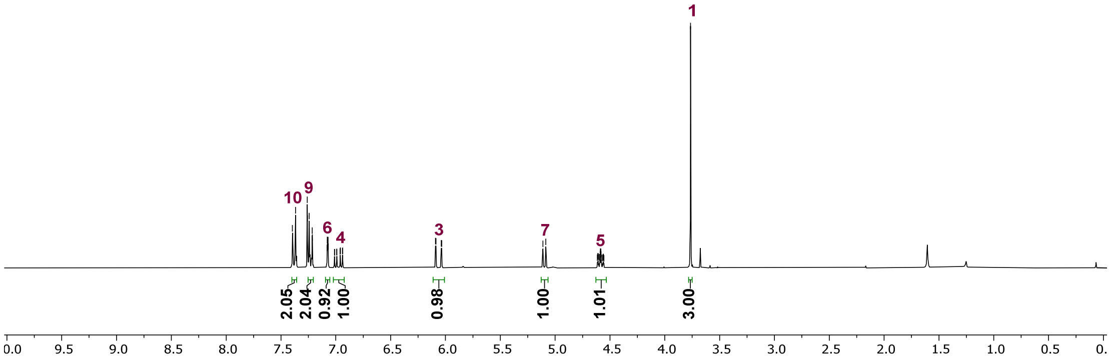

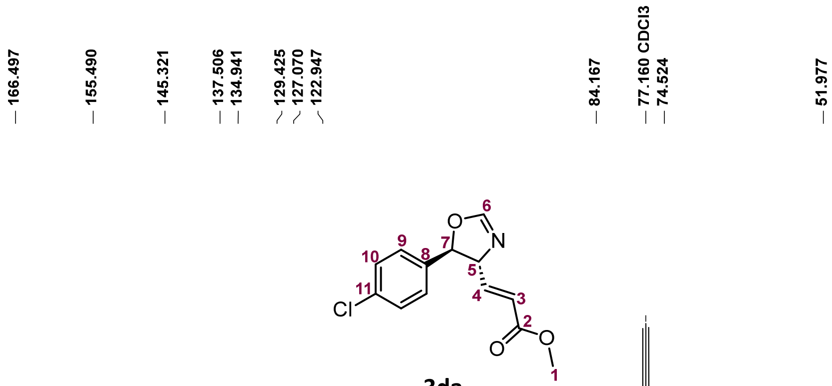

3da

^{13}C NMR (CDCl$_3$, 75 MHz)

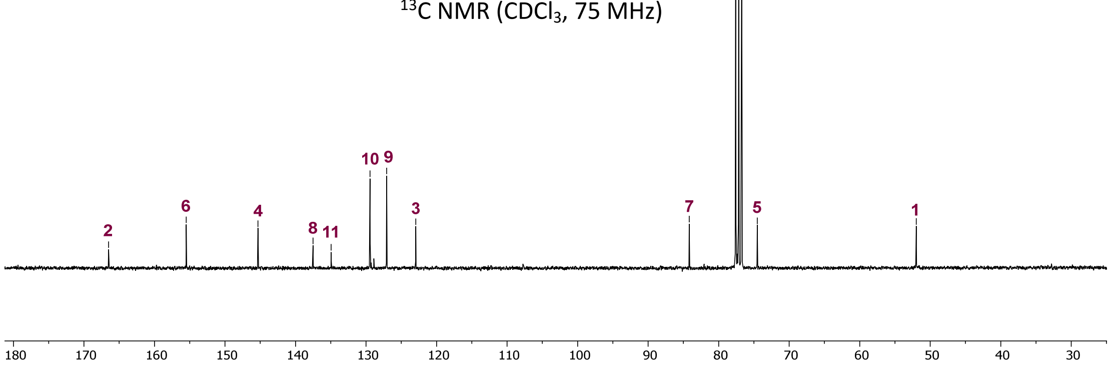

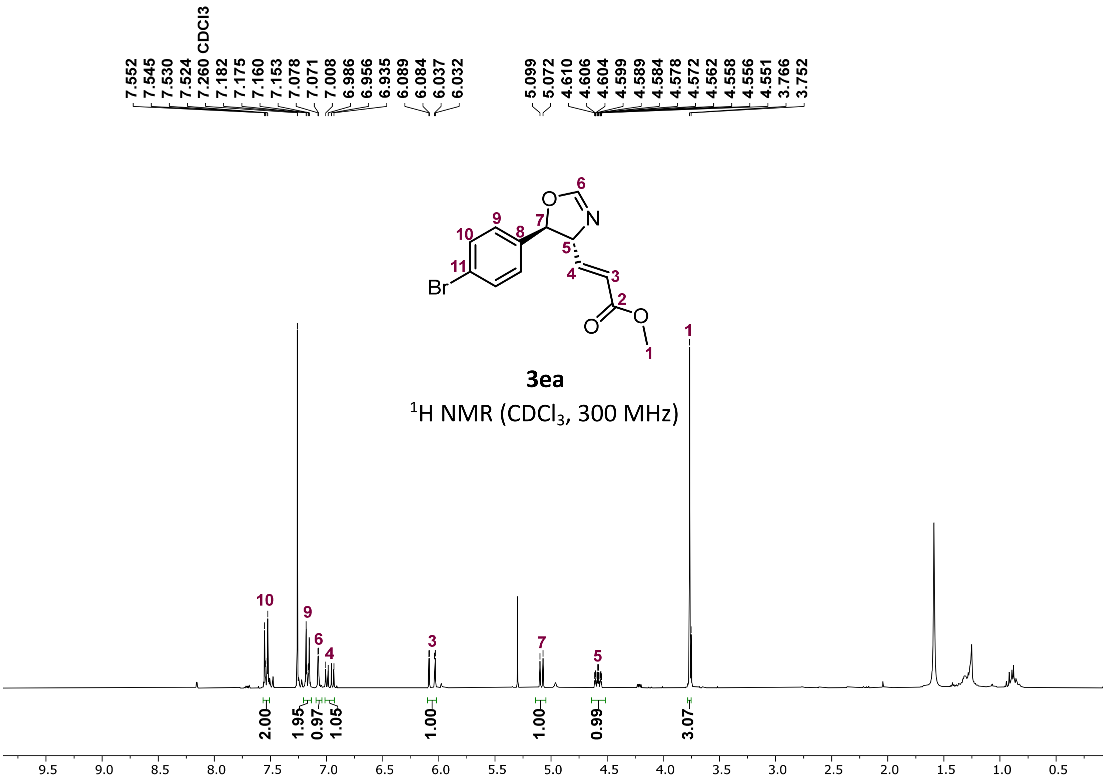
7.552
7.545
7.530
7.524
7.260 CDCl3
7.182
7.175
7.160
7.153
7.078
7.071
7.008
6.986
6.956
6.935
6.089
6.084
6.037
6.032
5.099
5.072
4.610
4.606
4.604
4.599
4.589
4.584
4.578
4.572
4.562
4.558
4.556
4.551
3.766
3.752
3ea
1H NMR (CDCl3, 300 MHz)
2.00
1.95
0.97
1.05
1.00
1.00
0.99
3.07

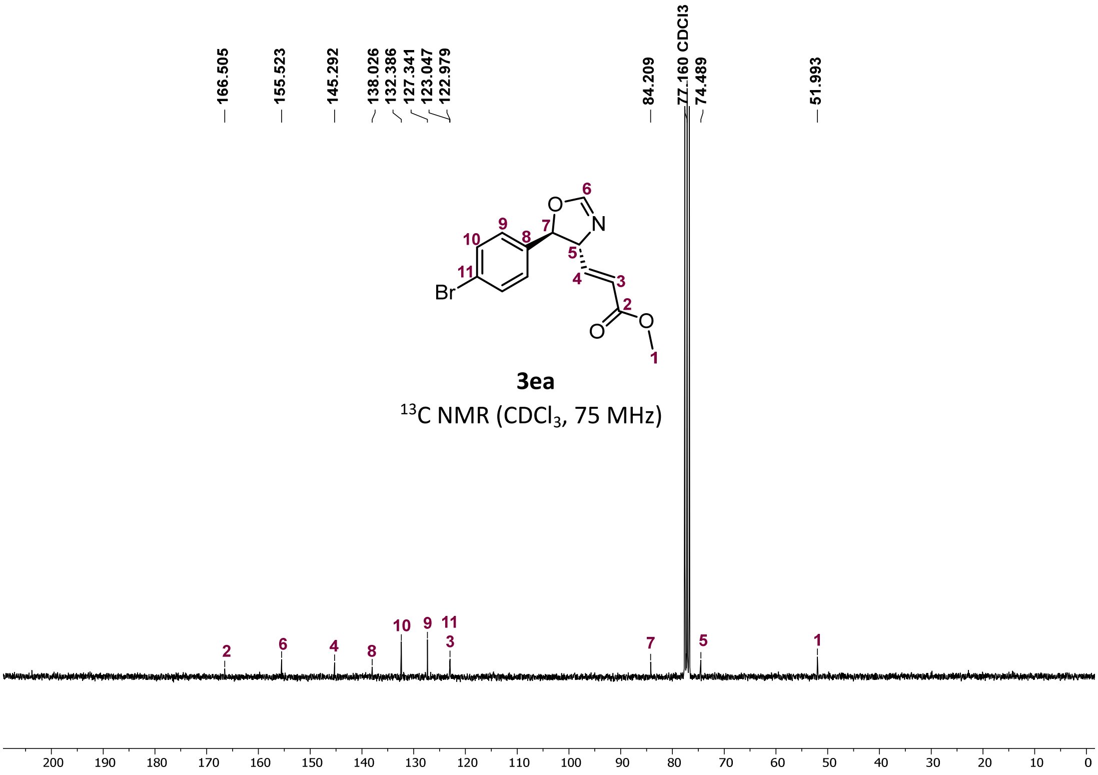
166.505
155.523
145.292
138.026
132.386
127.341
123.047
122.979
84.209
77.160 CDCl3
74.489
51.993
3ea
13C NMR (CDCl3, 75 MHz)

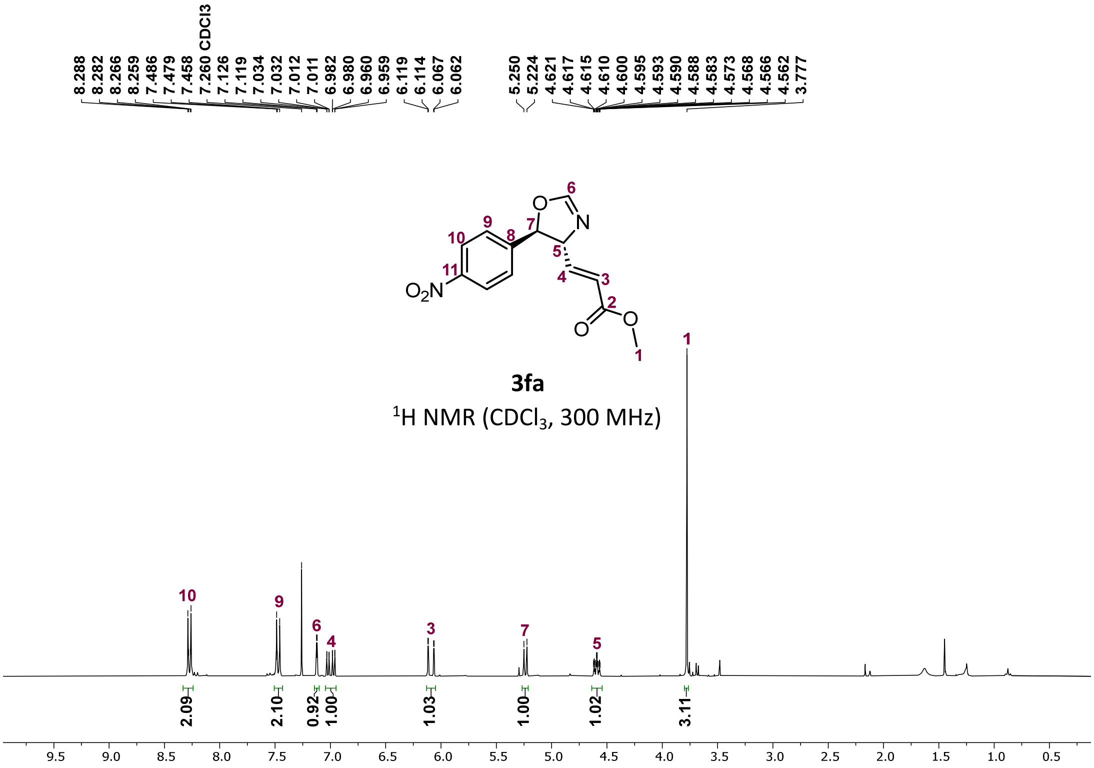
8.288 8.282 8.266 8.259 7.486 7.479 7.458 7.260 CDCl3 7.126 7.119 7.034 7.032 7.012 7.011 6.982 6.980 6.960 6.959 6.119 6.114 6.067 6.062
5.250 5.224 4.621 4.617 4.615 4.610 4.600 4.595 4.593 4.590 4.588 4.583 4.573 4.568 4.566 4.562 3.777
O
N
O2N
O
O
3fa
1H NMR (CDCl3, 300 MHz)
2.09 2.10 0.92 1.00 1.03 1.00 1.02 3.11
9.5 9.0 8.5 8.0 7.5 7.0 6.5 6.0 5.5 5.0 4.5 4.0 3.5 3.0 2.5 2.0 1.5 1.0 0.5

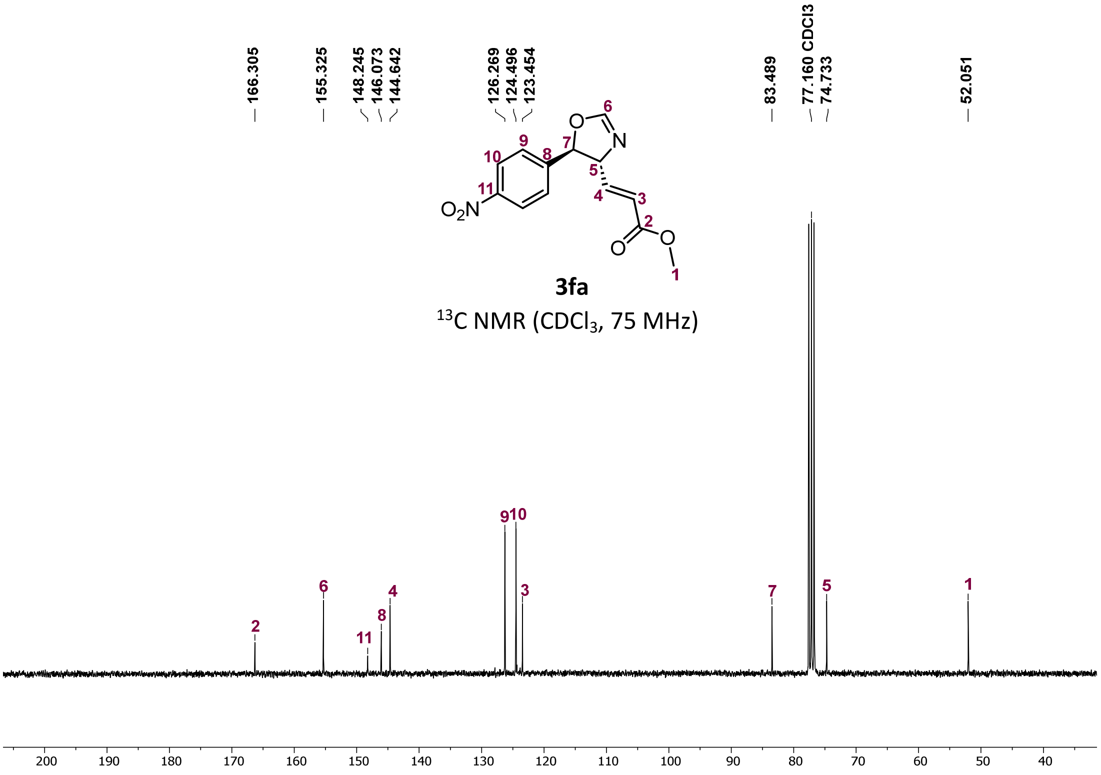
166.305
155.325
148.245 146.073 144.642
126.269 124.496 123.454
83.489
77.160 CDCl3 74.733
52.051
O
N
O2N
O
O
3fa
13C NMR (CDCl3, 75 MHz)
200 190 180 170 160 150 140 130 120 110 100 90 80 70 60 50 40

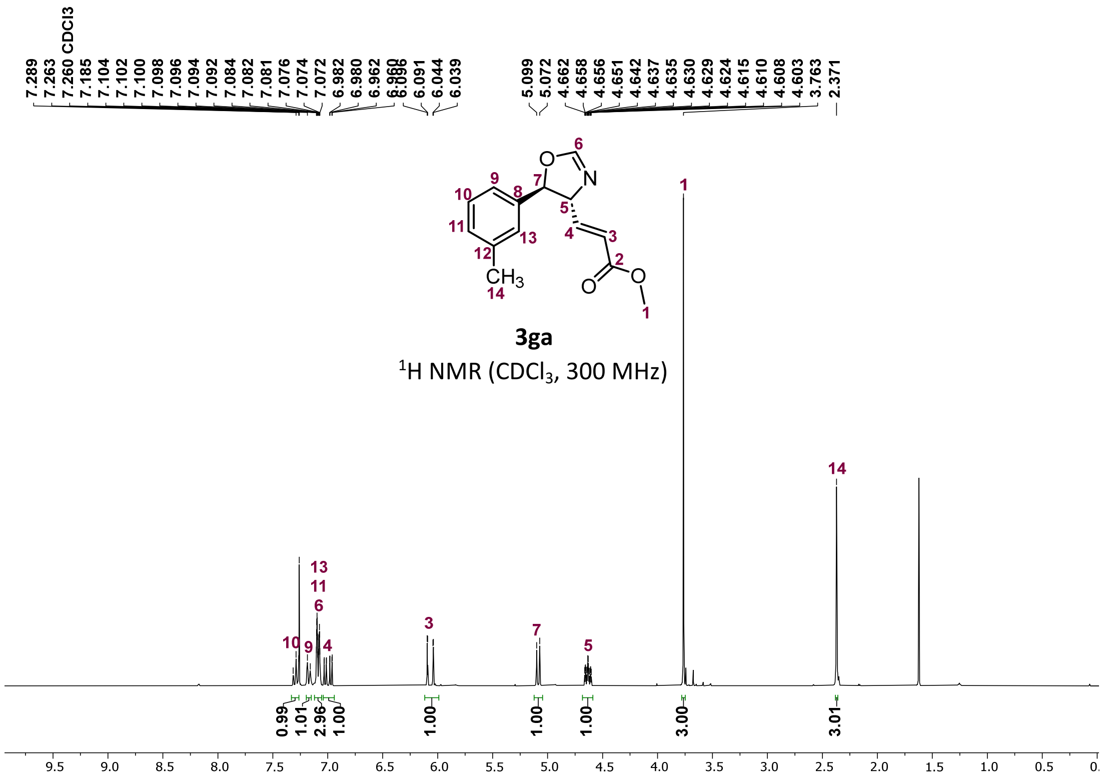
7.289
7.263
7.260 CDCl3
7.185
7.104
7.102
7.100
7.098
7.096
7.094
7.092
7.084
7.082
7.081
7.076
7.074
7.072
6.982
6.980
6.962
6.960
6.096
6.091
6.044
6.039
5.099
5.072
4.662
4.658
4.656
4.651
4.642
4.637
4.635
4.630
4.629
4.624
4.615
4.610
4.608
4.603
3.763
2.371
3ga
1H NMR (CDCl3, 300 MHz)
0.99
1.01
2.96
1.00
1.00
1.00
1.00
3.00
3.01

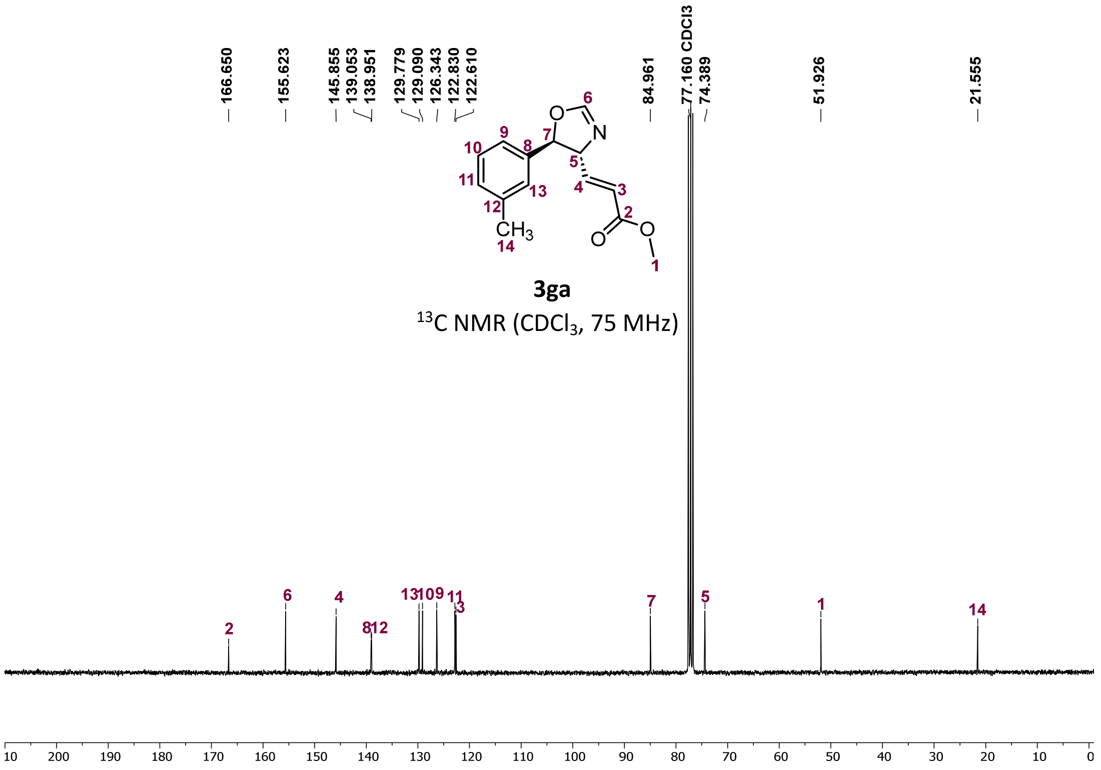
166.650
155.623
145.855
139.053
138.951
129.779
129.090
126.343
122.830
122.610
84.961
77.160 CDCl3
74.389
51.926
21.555
3ga
13C NMR (CDCl3, 75 MHz)

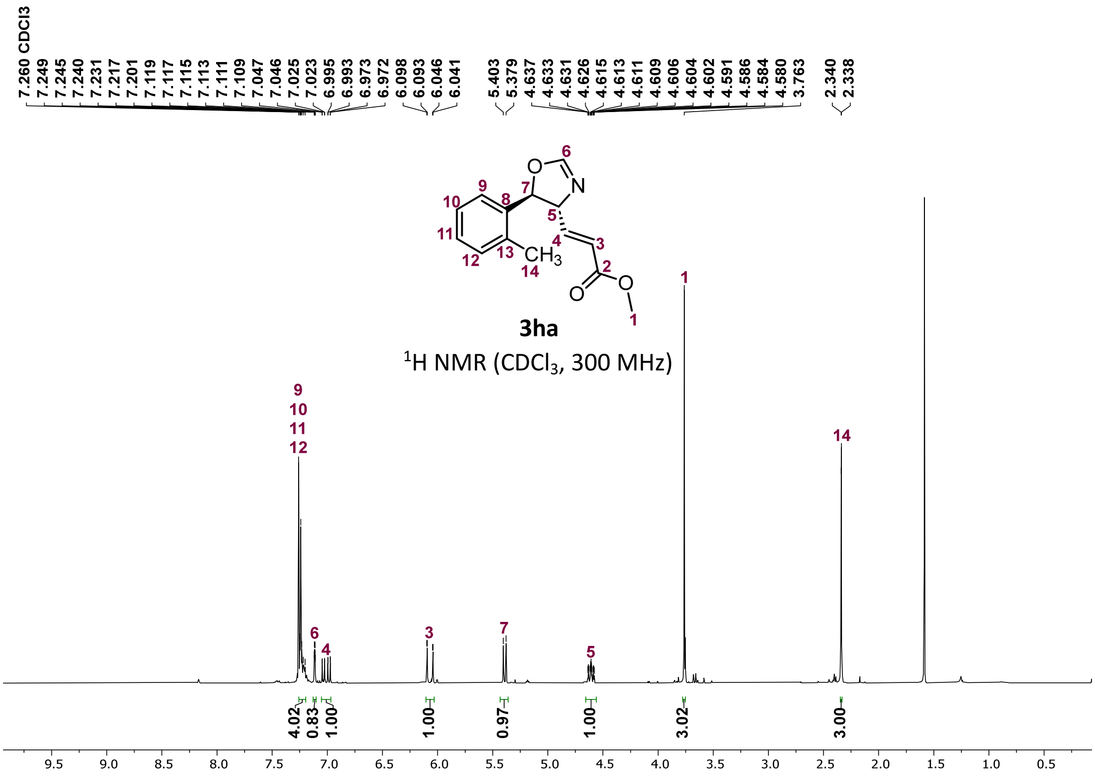

3ha

^{1}H NMR ($CDCl_3$, 300 MHz)

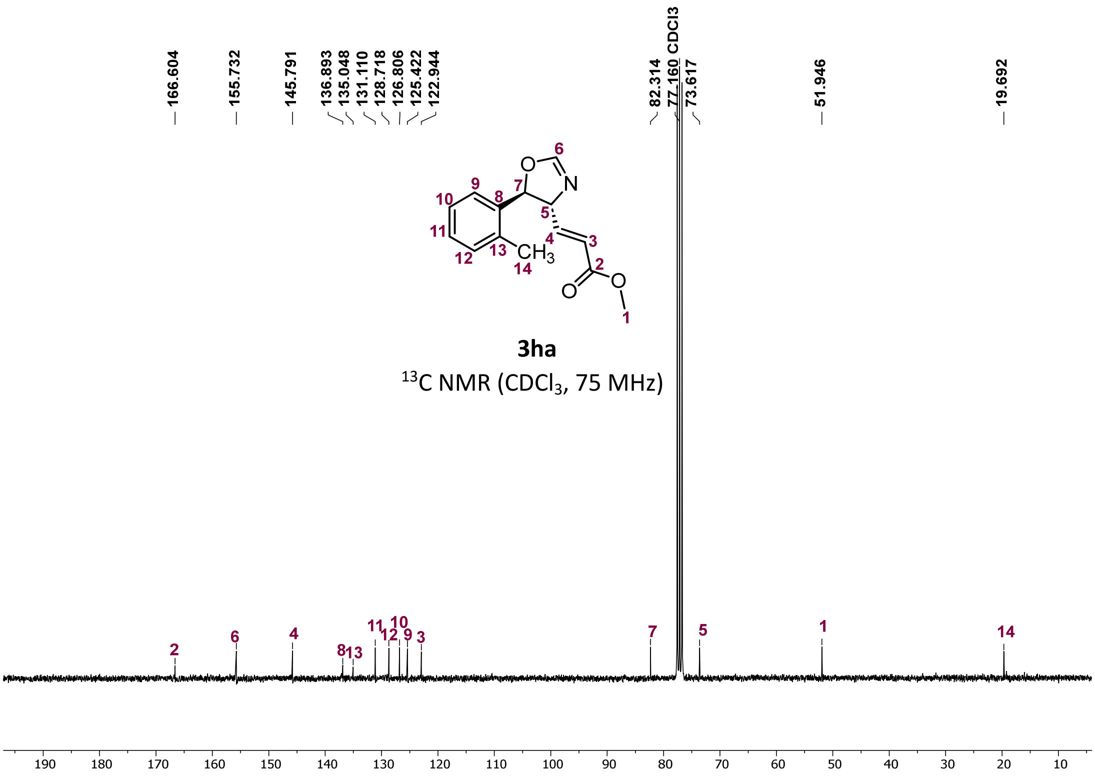

3ha

^{13}C NMR ($CDCl_3$, 75 MHz)

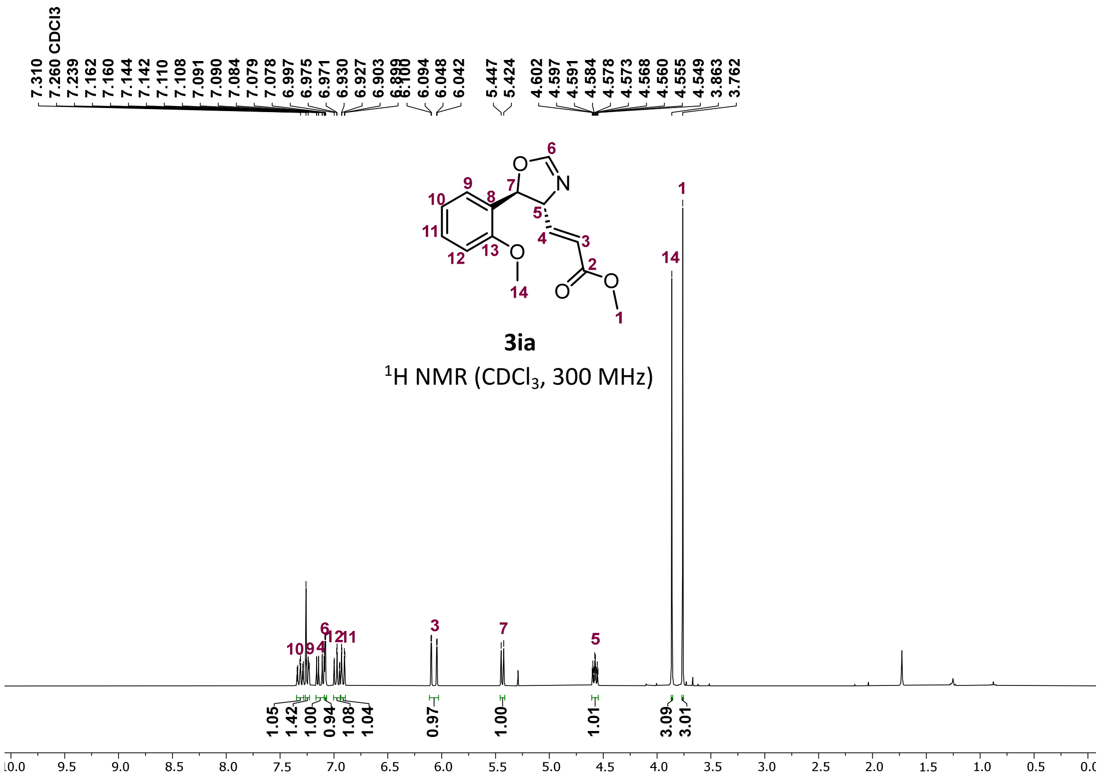
3ia
^{1}H NMR (CDCl$_3$, 300 MHz)

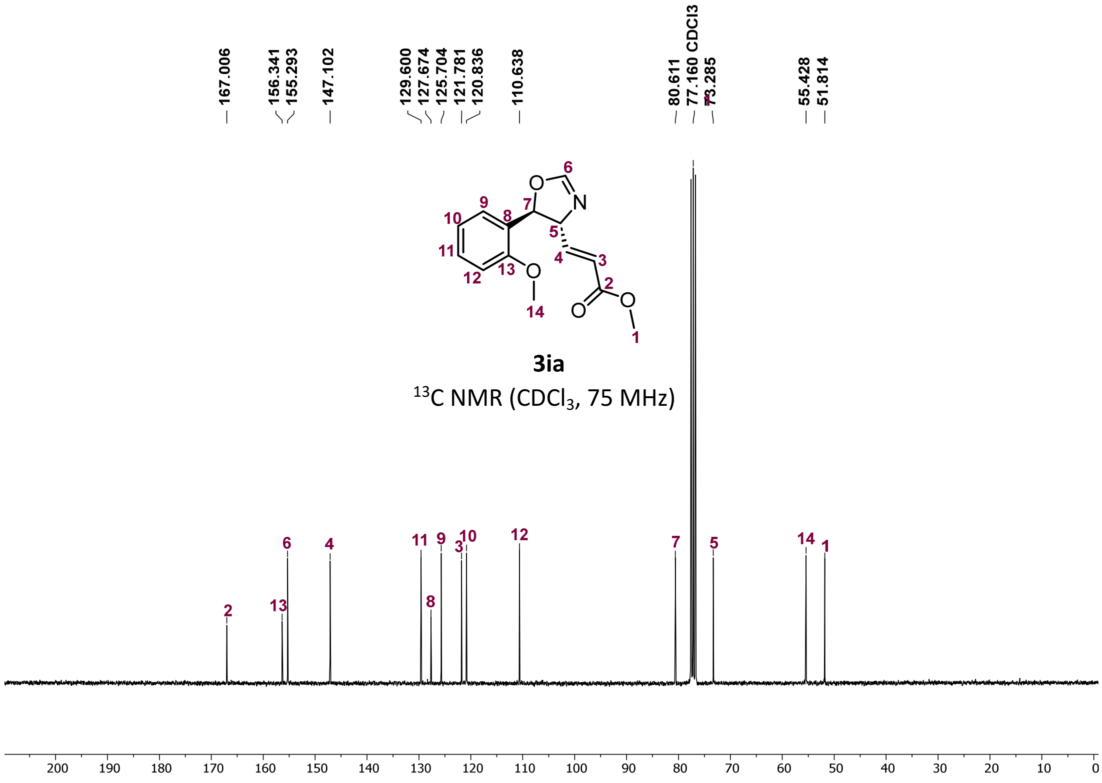
3ia
^{13}C NMR (CDCl$_3$, 75 MHz)

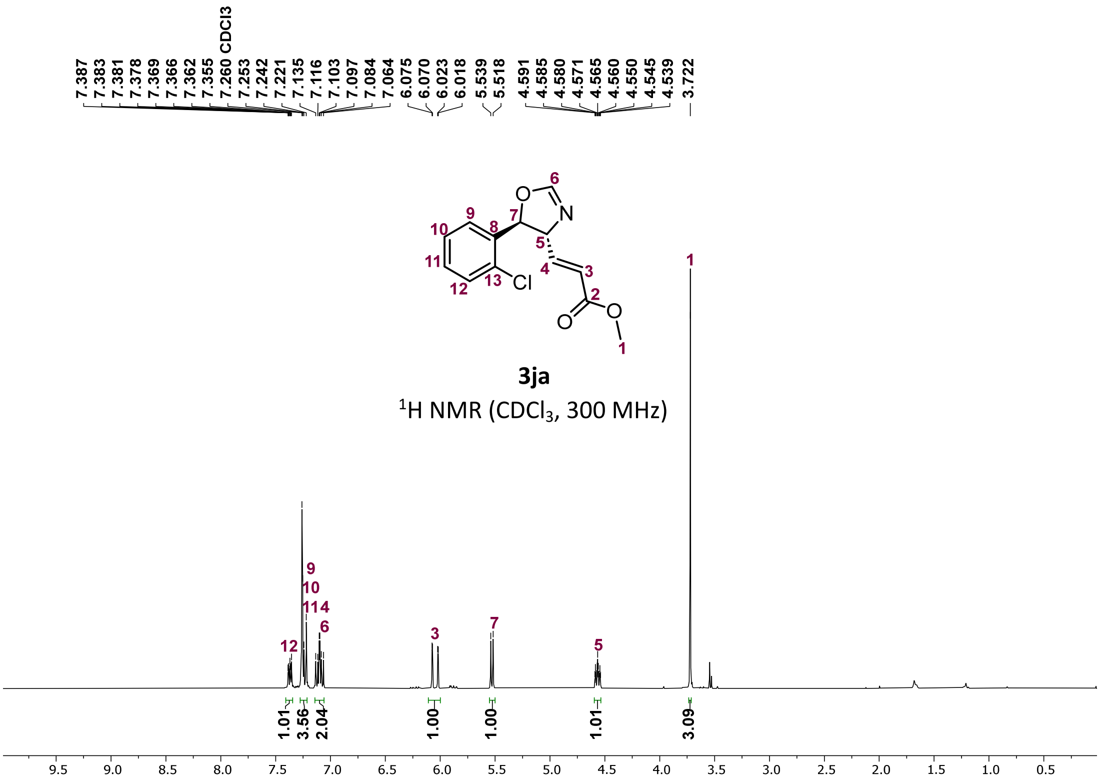
7.387 7.383 7.381 7.378 7.369 7.366 7.362 7.355 7.260 CDCl3 7.253 7.242 7.221 7.135 7.116 7.103 7.097 7.084 7.064 6.075 6.070 6.023 6.018 5.539 5.518 4.591 4.585 4.580 4.571 4.565 4.560 4.550 4.545 4.539 3.722
3ja
^{1}H NMR ($CDCl_3$, 300 MHz)
1.01 3.56 2.04 1.00 1.00 1.01 3.09
9.5 9.0 8.5 8.0 7.5 7.0 6.5 6.0 5.5 5.0 4.5 4.0 3.5 3.0 2.5 2.0 1.5 1.0 0.5

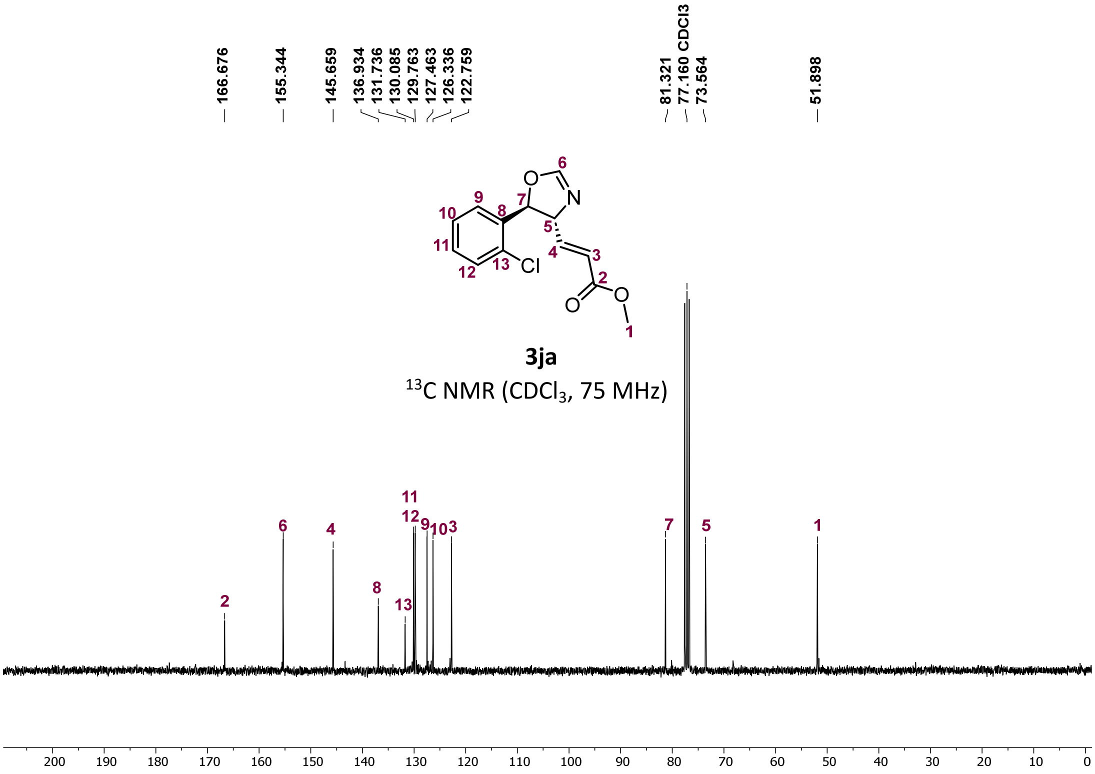
166.676 155.344 145.659 136.934 131.736 130.085 129.763 127.463 126.336 122.759 81.321 77.160 CDCl3 73.564 51.898
3ja
^{13}C NMR ($CDCl_3$, 75 MHz)
200 190 180 170 160 150 140 130 120 110 100 90 80 70 60 50 40 30 20 10 0

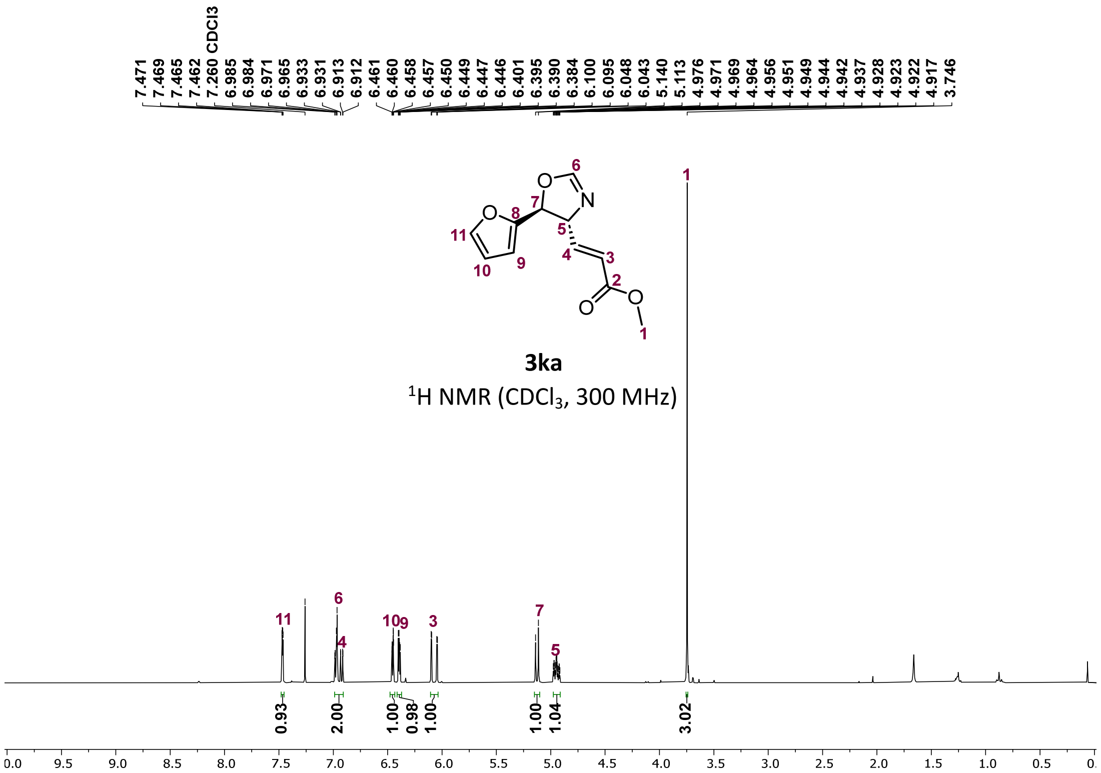
7.471 7.469 7.465 7.462 7.260 CDCl3 6.985 6.984 6.971 6.965 6.933 6.931 6.913 6.912 6.461 6.460 6.458 6.457 6.450 6.449 6.447 6.446 6.401 6.395 6.390 6.384 6.100 6.095 6.048 6.043 5.140 5.113 4.976 4.971 4.969 4.964 4.956 4.951 4.949 4.944 4.942 4.937 4.928 4.923 4.922 4.917 3.746
3ka
^{1}H NMR (CDCl$_3$, 300 MHz)
0.93 2.00 1.00 0.98 1.00 1.00 1.04 3.02

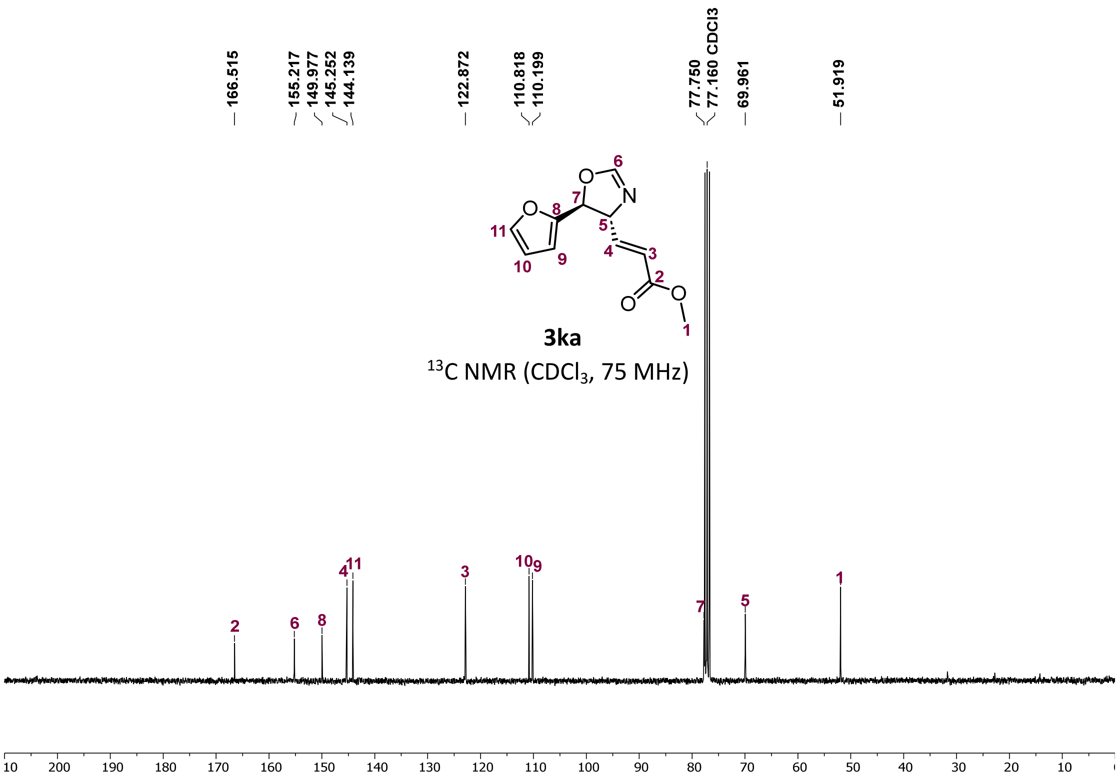
166.515 155.217 149.977 145.252 144.139 122.872 110.818 110.199 77.750 77.160 CDCl3 69.961 51.919
3ka
^{13}C NMR (CDCl$_3$, 75 MHz)

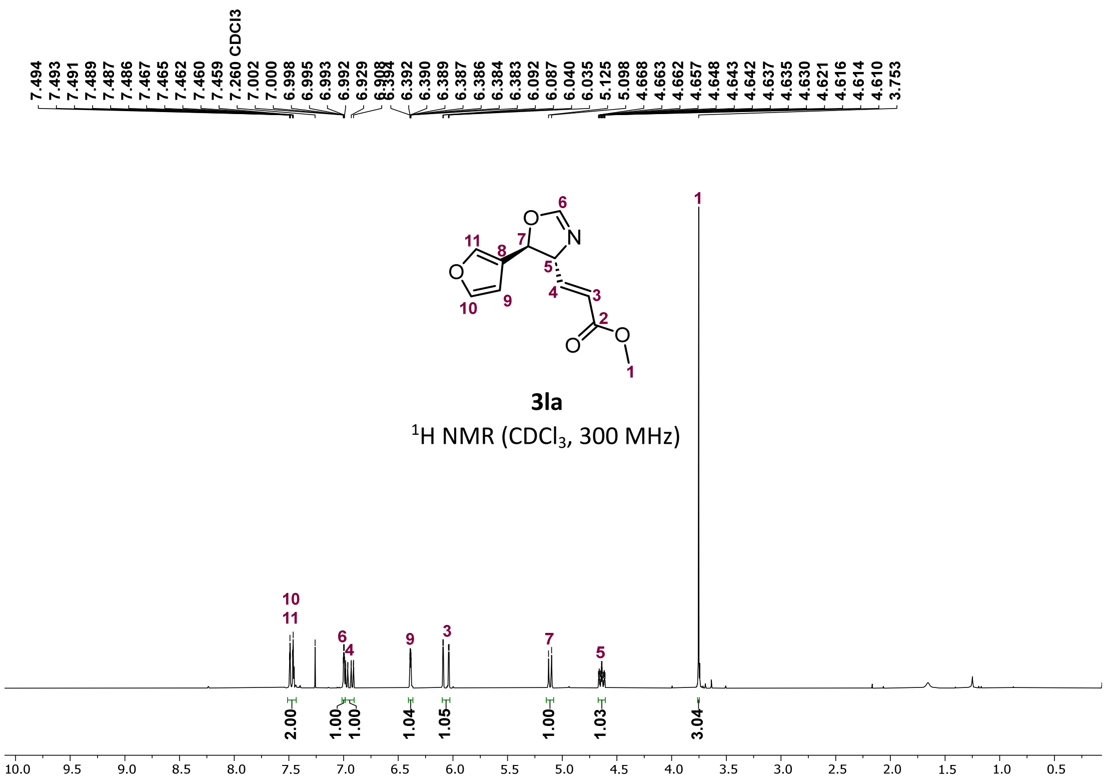
7.494
7.493
7.491
7.489
7.487
7.486
7.467
7.465
7.462
7.460
7.459
7.260 CDCl3
7.002
7.000
6.998
6.995
6.993
6.992
6.929
6.392
6.390
6.389
6.387
6.386
6.384
6.383
6.092
6.087
6.040
6.035
5.125
5.098
4.668
4.663
4.662
4.657
4.648
4.643
4.642
4.637
4.635
4.630
4.621
4.616
4.614
4.610
3.753
3la
^{1}H NMR (CDCl$_3$, 300 MHz)
2.00
1.00
1.00
1.04
1.05
1.00
1.03
3.04

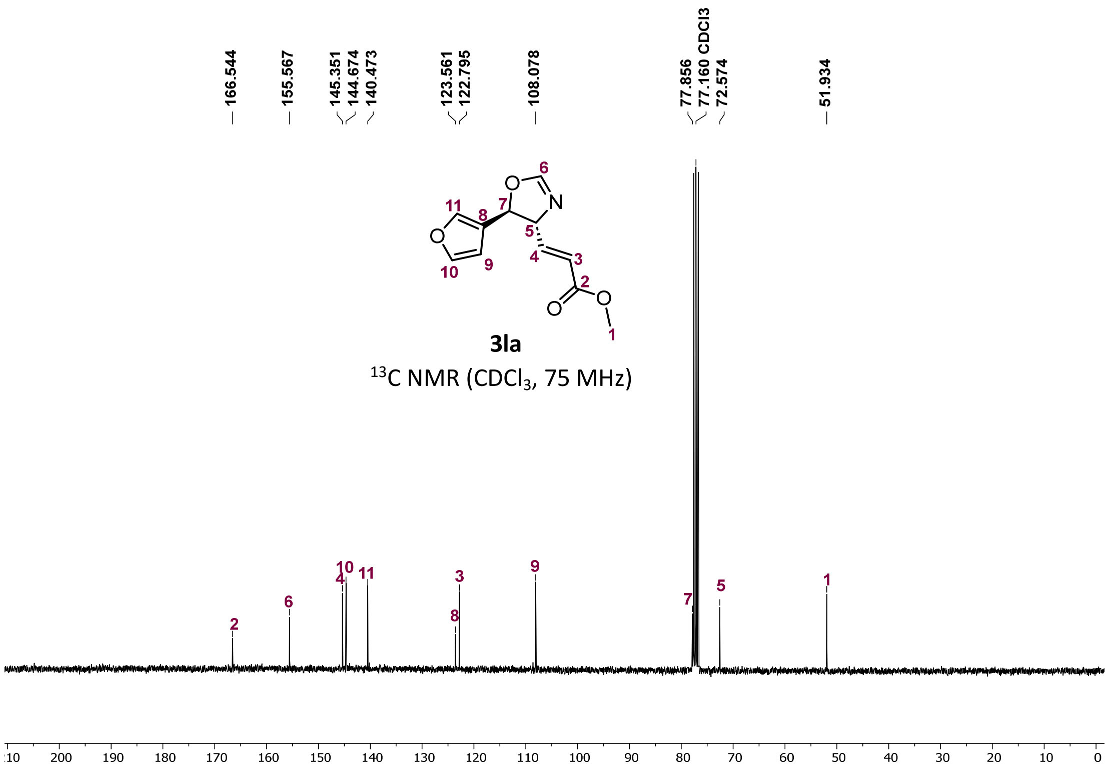
166.544
155.567
145.351
144.674
140.473
123.561
122.795
108.078
77.856
77.160 CDCl3
72.574
51.934
3la
^{13}C NMR (CDCl$_3$, 75 MHz)

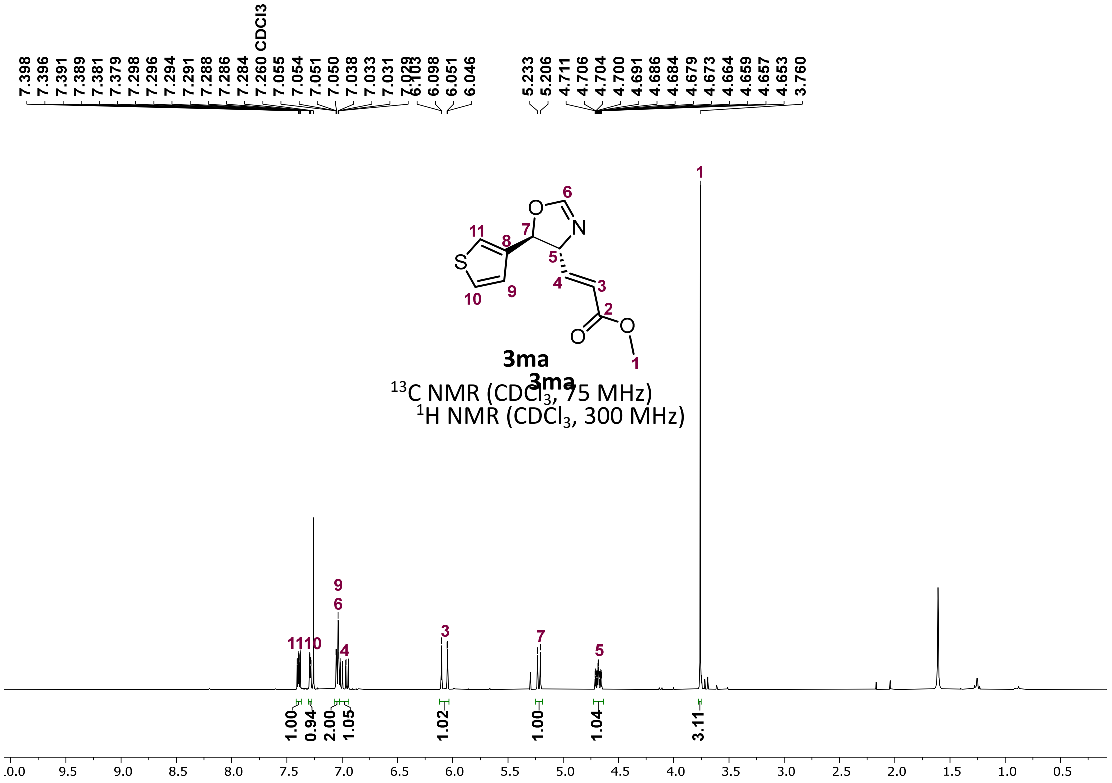
3ma
13C NMR (CDCl3, 75 MHz)
1H NMR (CDCl3, 300 MHz)
7.398 7.396 7.391 7.389 7.381 7.379 7.298 7.296 7.294 7.291 7.288 7.286 7.284 7.260 CDCl3 7.055 7.054 7.051 7.050 7.038 7.033 7.031 7.029 6.103 6.098 6.051 6.046
5.233 5.206 4.711 4.706 4.704 4.700 4.691 4.686 4.684 4.679 4.673 4.664 4.659 4.657 4.653 3.760
1.00 0.94 2.00 1.05 1.02 1.00 1.04 3.11
10.0 9.5 9.0 8.5 8.0 7.5 7.0 6.5 6.0 5.5 5.0 4.5 4.0 3.5 3.0 2.5 2.0 1.5 1.0 0.5

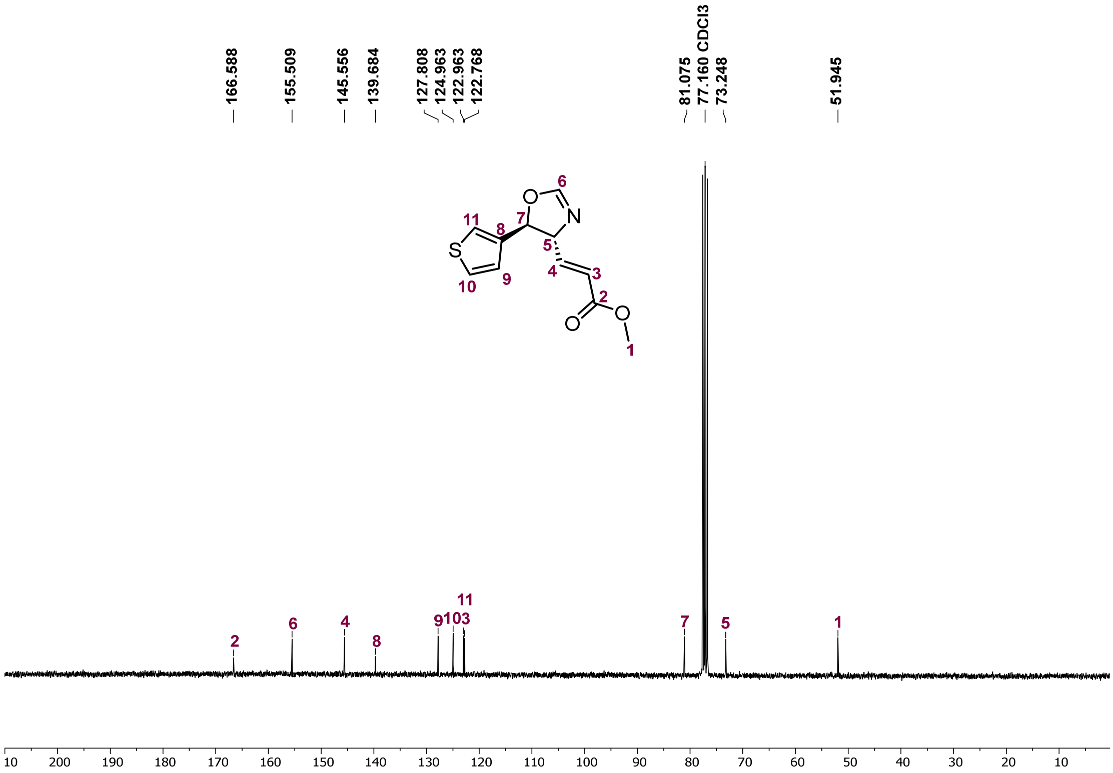
166.588 155.509 145.556 139.684 127.808 124.963 122.963 122.768 81.075 77.160 CDCl3 73.248 51.945
210 200 190 180 170 160 150 140 130 120 110 100 90 80 70 60 50 40 30 20 10

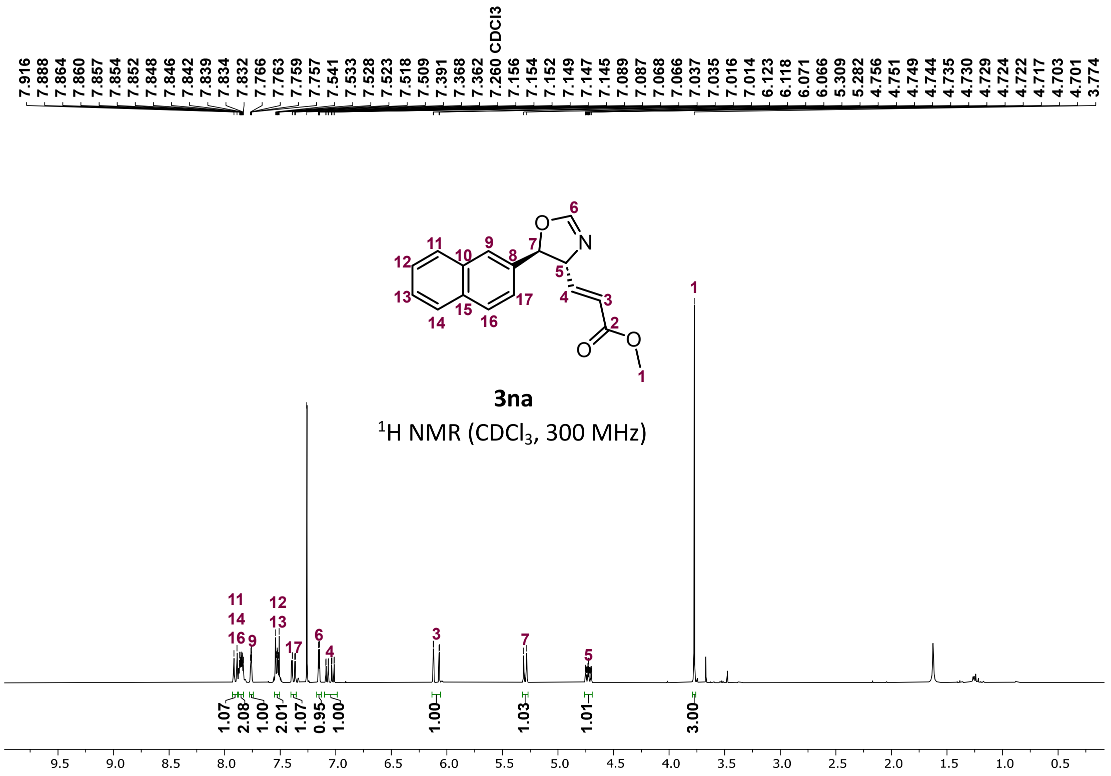
7.916 7.888 7.864 7.860 7.857 7.854 7.852 7.848 7.846 7.842 7.839 7.834 7.832 7.766 7.763 7.759 7.757 7.541 7.533 7.528 7.523 7.518 7.509 7.391 7.368 7.362 7.260 CDCl3 7.156 7.154 7.152 7.149 7.147 7.145 7.089 7.087 7.068 7.066 7.037 7.035 7.016 7.014 6.123 6.118 6.071 6.066 5.309 5.282 4.756 4.751 4.749 4.744 4.735 4.730 4.729 4.724 4.722 4.717 4.703 4.701 3.774
3na
^{1}H NMR ($CDCl_3$, 300 MHz)
1.07 2.08 1.00 2.01 1.07 0.95 1.00 1.00 1.03 1.01 3.00

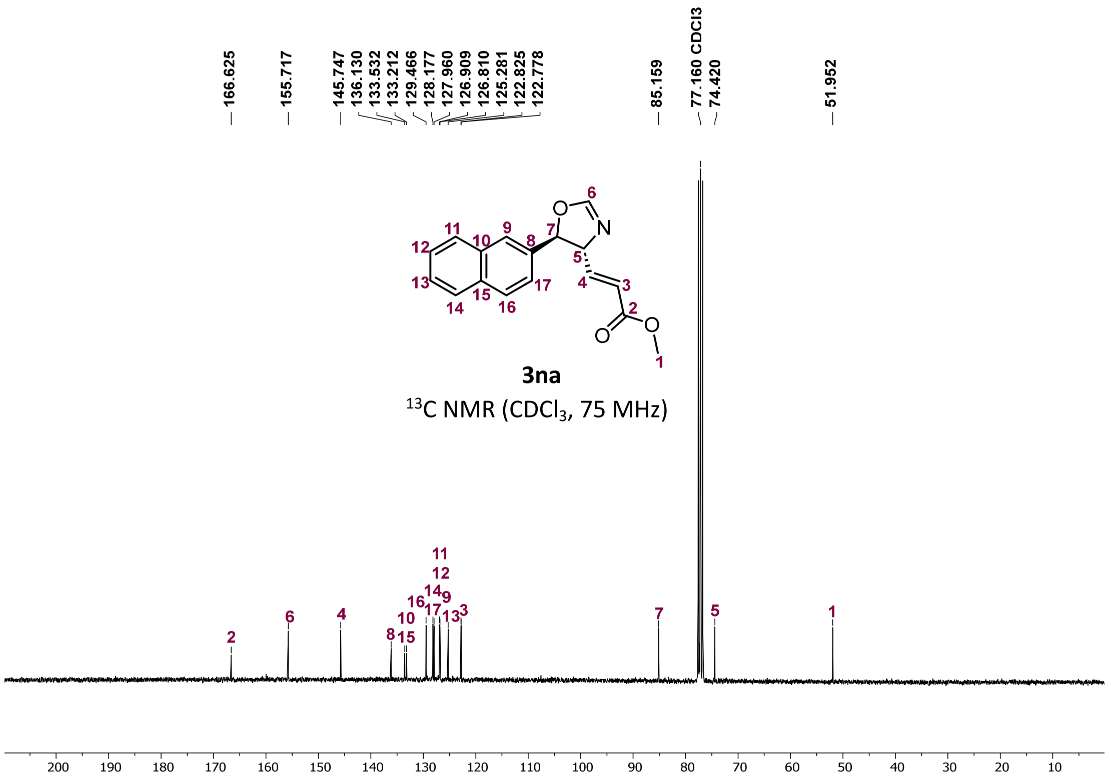
166.625 155.717 145.747 136.130 133.532 133.212 129.466 128.177 127.960 126.909 126.810 125.281 122.825 122.778 85.159 77.160 CDCl3 74.420 51.952
3na
^{13}C NMR ($CDCl_3$, 75 MHz)

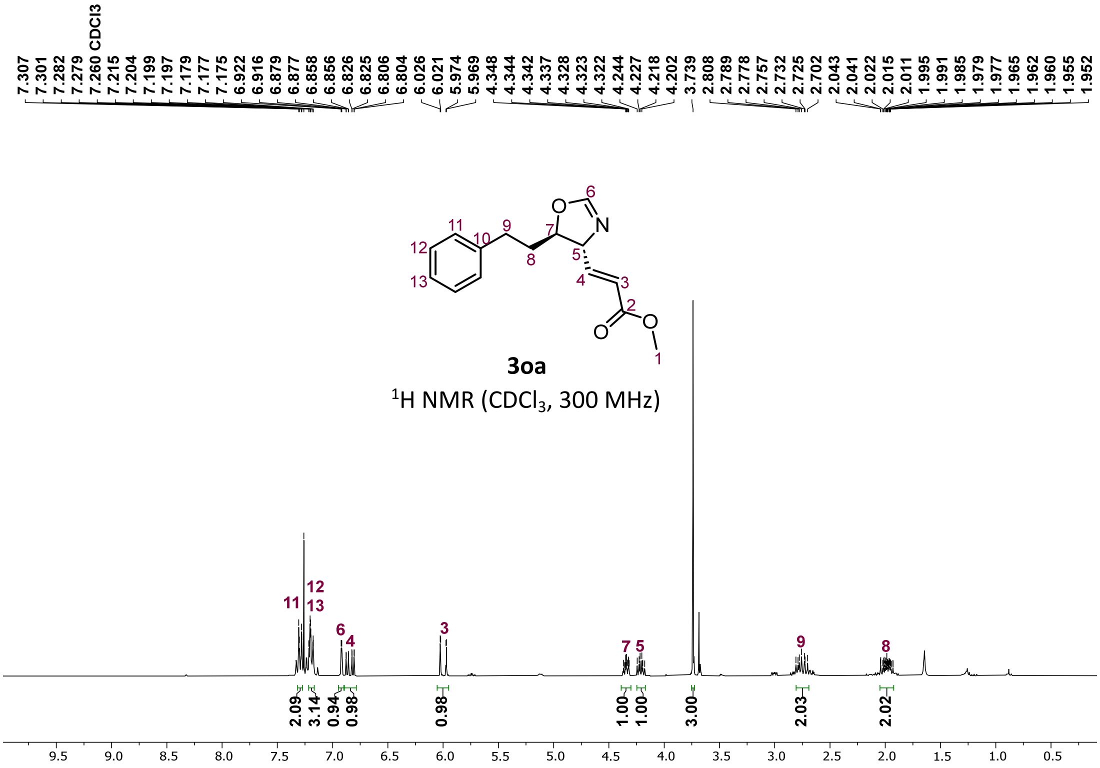

7.307
7.301
7.282
7.279
7.260 CDCl3
7.215
7.204
7.199
7.197
7.179
7.177
7.175
6.922
6.916
6.879
6.877
6.858
6.856
6.826
6.825
6.806
6.804
6.026
6.021
5.974
5.969
4.348
4.344
4.342
4.337
4.328
4.323
4.322
4.244
4.227
4.218
4.202
3.739
2.808
2.789
2.778
2.757
2.732
2.725
2.702
2.043
2.041
2.022
2.015
2.011
1.995
1.991
1.985
1.979
1.977
1.965
1.962
1.960
1.955
1.952
3oa
^{1}H NMR ($CDCl_3$, 300 MHz)
11
12
13
6
4
3
7
5
9
8
2.09
3.14
0.94
0.98
0.98
1.00
1.00
3.00
2.03
2.02
9.5
9.0
8.5
8.0
7.5
7.0
6.5
6.0
5.5
5.0
4.5
4.0
3.5
3.0
2.5
2.0
1.5
1.0
0.5

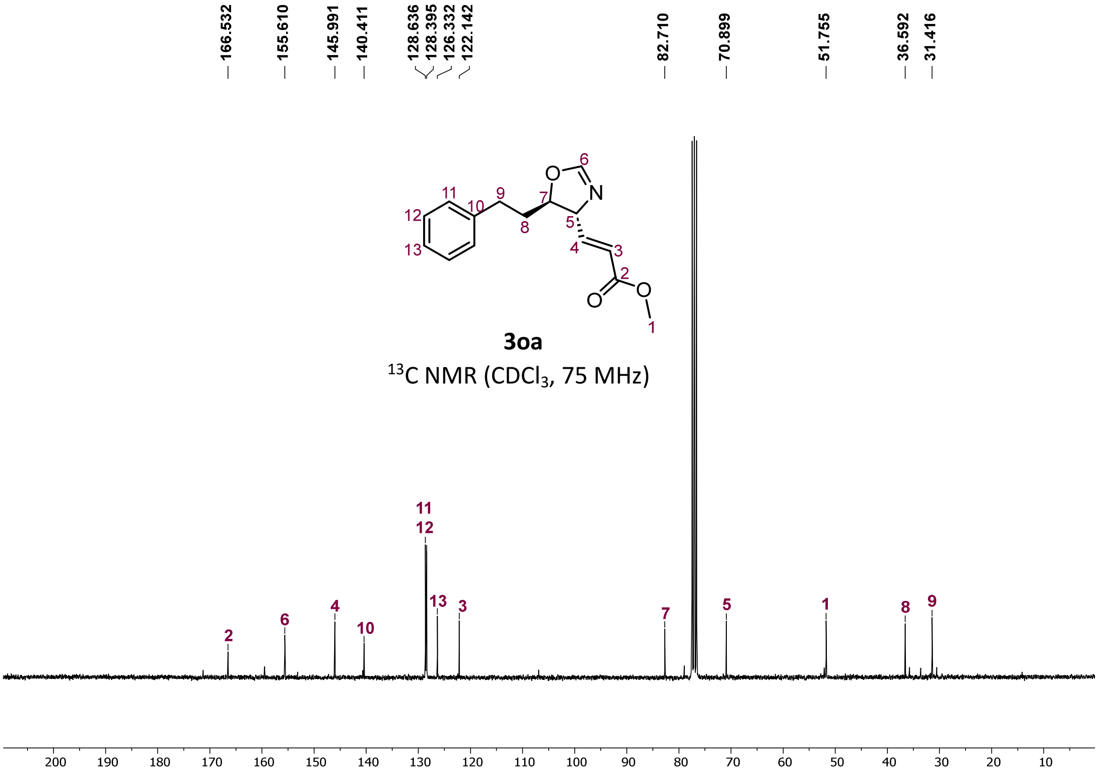

166.532
155.610
145.991
140.411
128.636
128.395
126.332
122.142
82.710
70.899
51.755
36.592
31.416
3oa
^{13}C NMR ($CDCl_3$, 75 MHz)
11
12
2
6
4
10
13
3
7
5
1
8
9
200
190
180
170
160
150
140
130
120
110
100
90
80
70
60
50
40
30
20
10

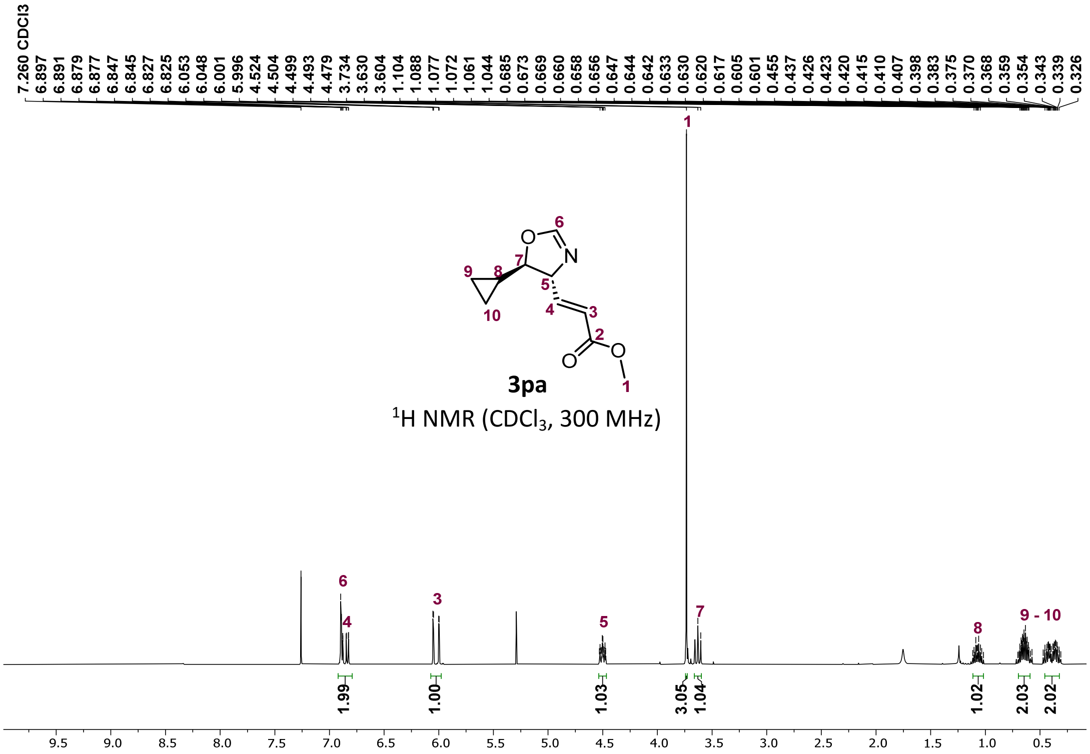
7.260 CDCl3
6.897
6.891
6.879
6.877
6.847
6.845
6.827
6.825
6.053
6.048
6.001
5.996
4.524
4.504
4.499
4.493
4.479
3.734
3.630
3.604
1.104
1.088
1.077
1.072
1.061
1.044
0.685
0.673
0.669
0.660
0.658
0.656
0.647
0.644
0.642
0.633
0.630
0.620
0.617
0.605
0.601
0.455
0.437
0.426
0.423
0.420
0.415
0.410
0.407
0.398
0.383
0.375
0.370
0.368
0.359
0.354
0.343
0.339
0.326
3pa
1H NMR (CDCl3, 300 MHz)
1.99
1.00
1.03
3.05
1.04
1.02
2.03
2.02

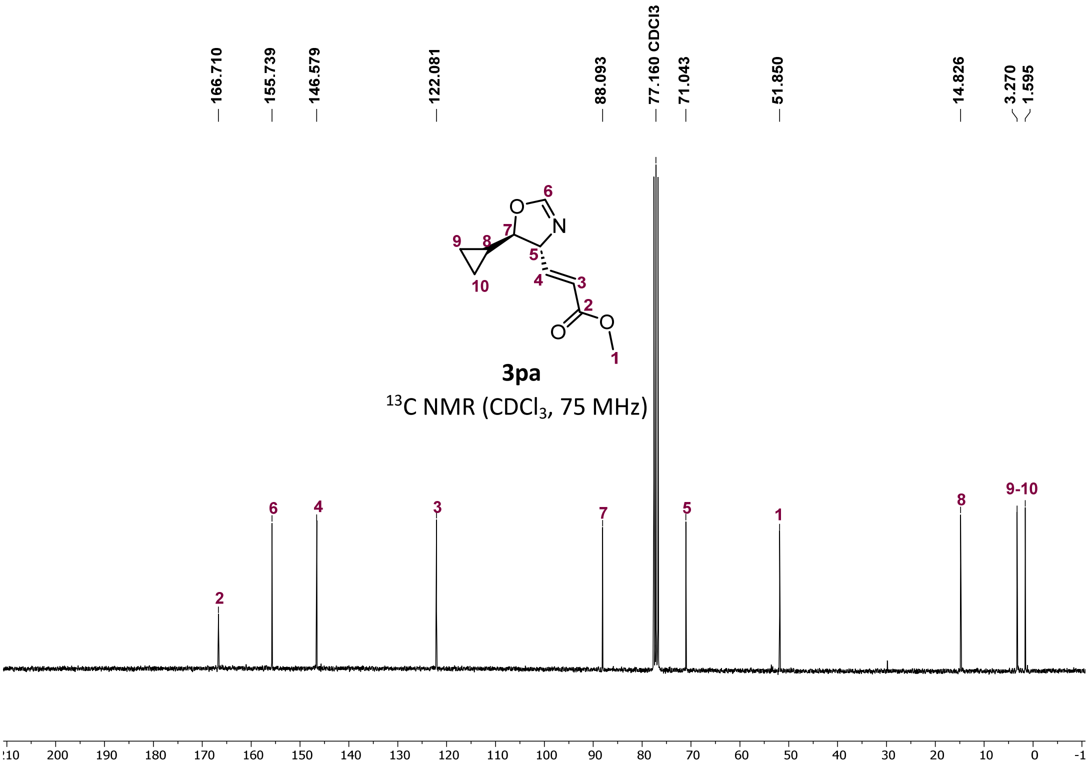
166.710
155.739
146.579
122.081
88.093
77.160 CDCl3
71.043
51.850
14.826
3.270
1.595
3pa
13C NMR (CDCl3, 75 MHz)

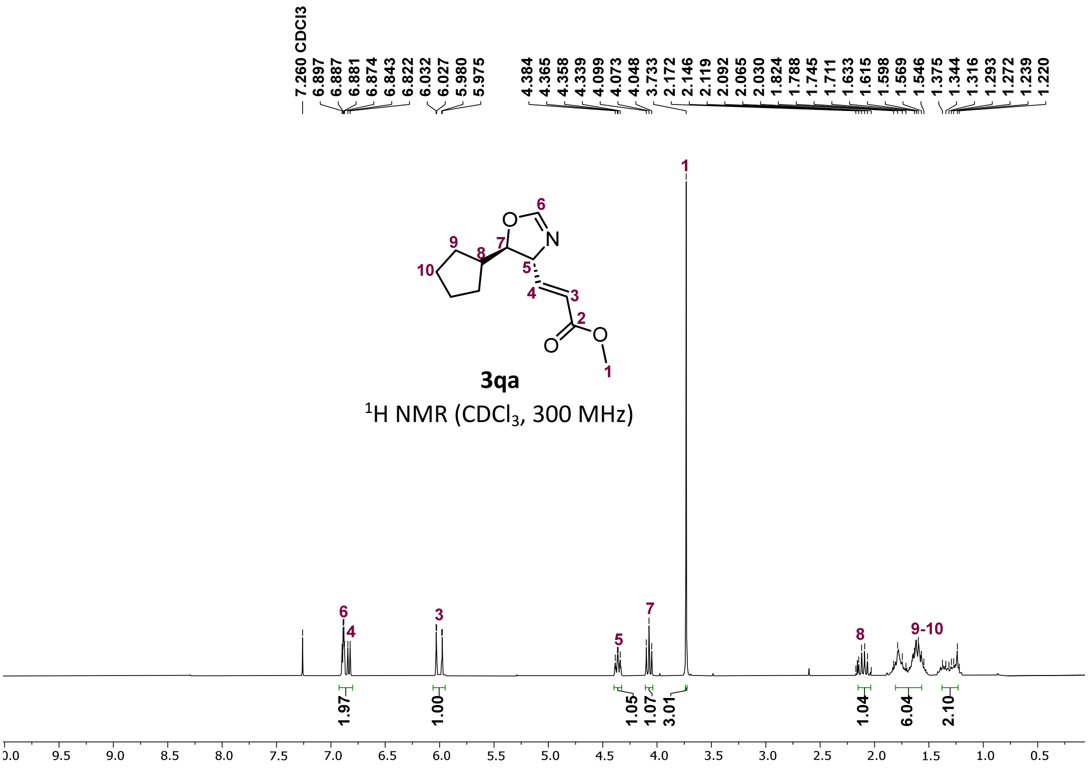
7.260 CDCl3
6.897
6.887
6.881
6.874
6.843
6.822
6.032
6.027
5.980
5.975
4.384
4.365
4.358
4.339
4.099
4.073
4.048
3.733
2.172
2.146
2.119
2.092
2.065
2.030
1.824
1.788
1.745
1.711
1.633
1.615
1.598
1.569
1.546
1.375
1.344
1.316
1.293
1.272
1.239
1.220
3qa
^{1}H NMR (CDCl$_3$, 300 MHz)
1.97
1.00
1.05
1.07
3.01
1.04
6.04
2.10
9-10

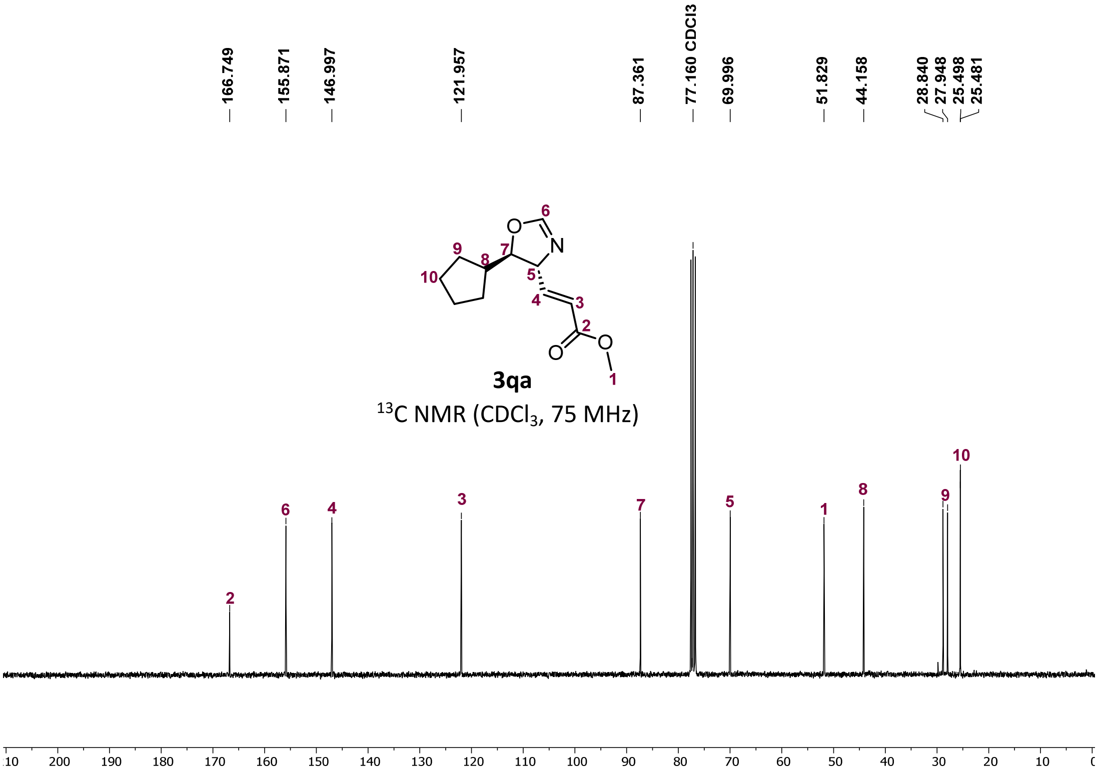
166.749
155.871
146.997
121.957
87.361
77.160 CDCl3
69.996
51.829
44.158
28.840
27.948
25.498
25.481
3qa
^{13}C NMR (CDCl$_3$, 75 MHz)

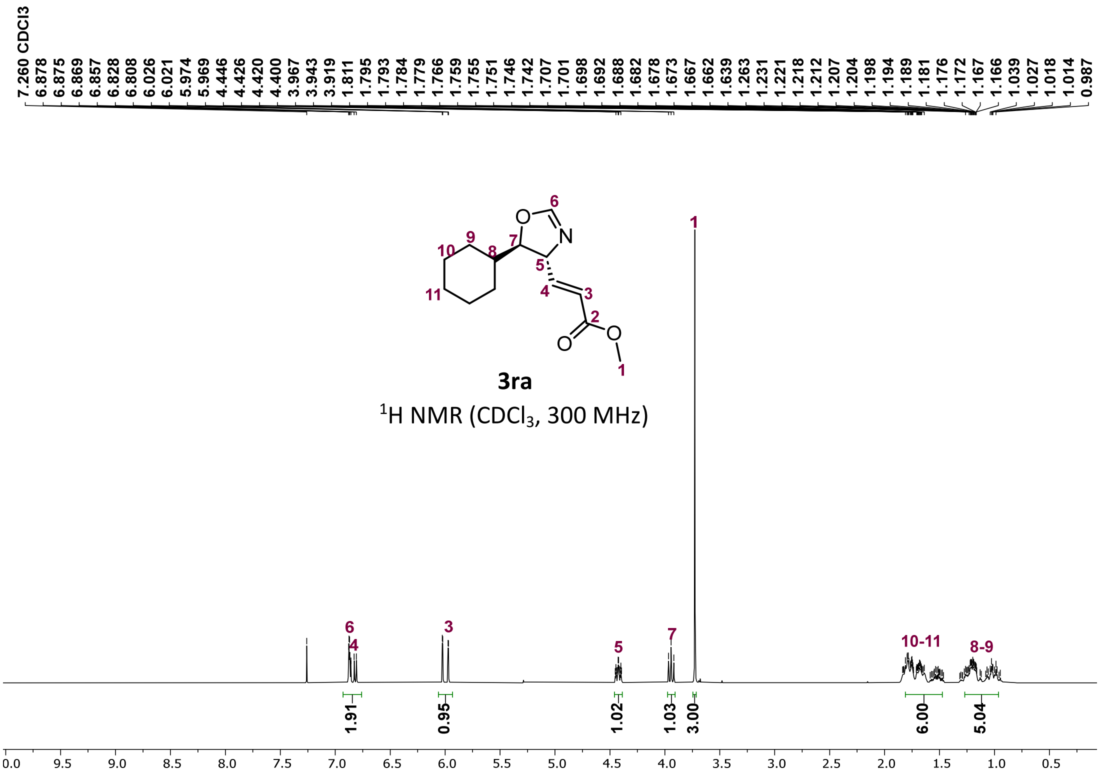

7.260 CDCl3
6.878
6.875
6.869
6.857
6.828
6.808
6.026
6.021
5.974
5.969
4.446
4.426
4.420
4.400
3.967
3.943
3.919
1.811
1.795
1.793
1.784
1.779
1.766
1.759
1.755
1.751
1.746
1.742
1.707
1.701
1.698
1.692
1.688
1.682
1.678
1.673
1.667
1.662
1.639
1.263
1.231
1.221
1.218
1.212
1.207
1.204
1.198
1.194
1.189
1.181
1.176
1.172
1.167
1.166
1.039
1.027
1.018
1.014
0.987
3ra
^{1}H NMR (CDCl$_3$, 300 MHz)
1.91
0.95
1.02
1.03
3.00
6.00
5.04

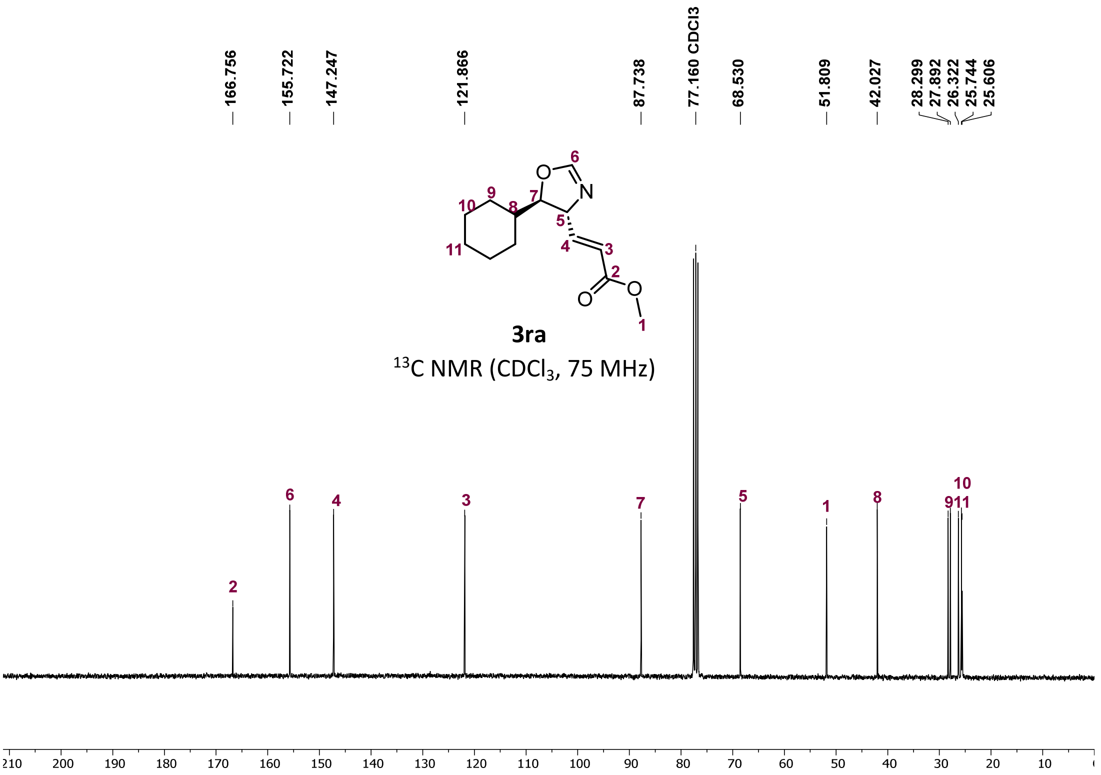

166.756
155.722
147.247
121.866
87.738
77.160 CDCl3
68.530
51.809
42.027
28.299
27.892
26.322
25.744
25.606
3ra
^{13}C NMR (CDCl$_3$, 75 MHz)

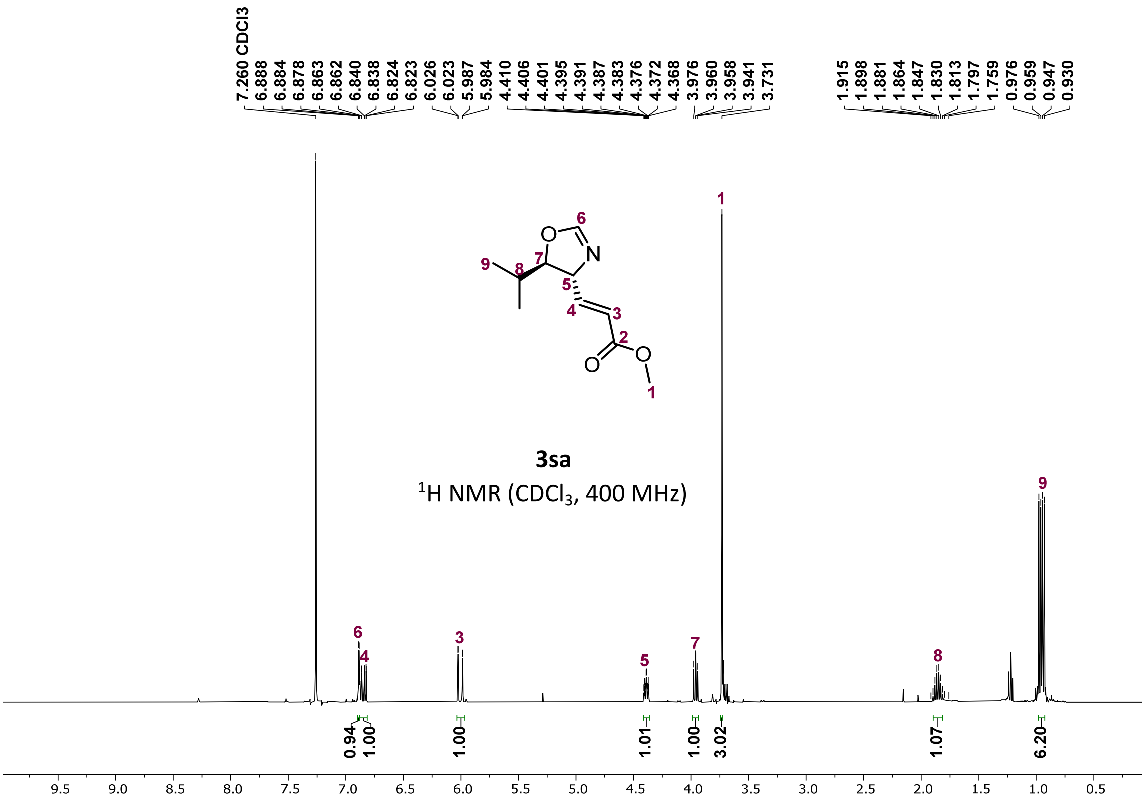
7.260 CDCl3
6.888
6.884
6.878
6.863
6.862
6.840
6.838
6.824
6.823
6.026
6.023
5.987
5.984
4.410
4.406
4.401
4.395
4.391
4.387
4.383
4.376
4.372
4.368
3.976
3.960
3.958
3.941
3.731
1.915
1.898
1.881
1.864
1.847
1.830
1.813
1.797
1.759
0.976
0.959
0.947
0.930
3sa
1H NMR (CDCl3, 400 MHz)
0.94
1.00
1.00
1.01
1.00
3.02
1.07
6.20
9.5
9.0
8.5
8.0
7.5
7.0
6.5
6.0
5.5
5.0
4.5
4.0
3.5
3.0
2.5
2.0
1.5
1.0
0.5

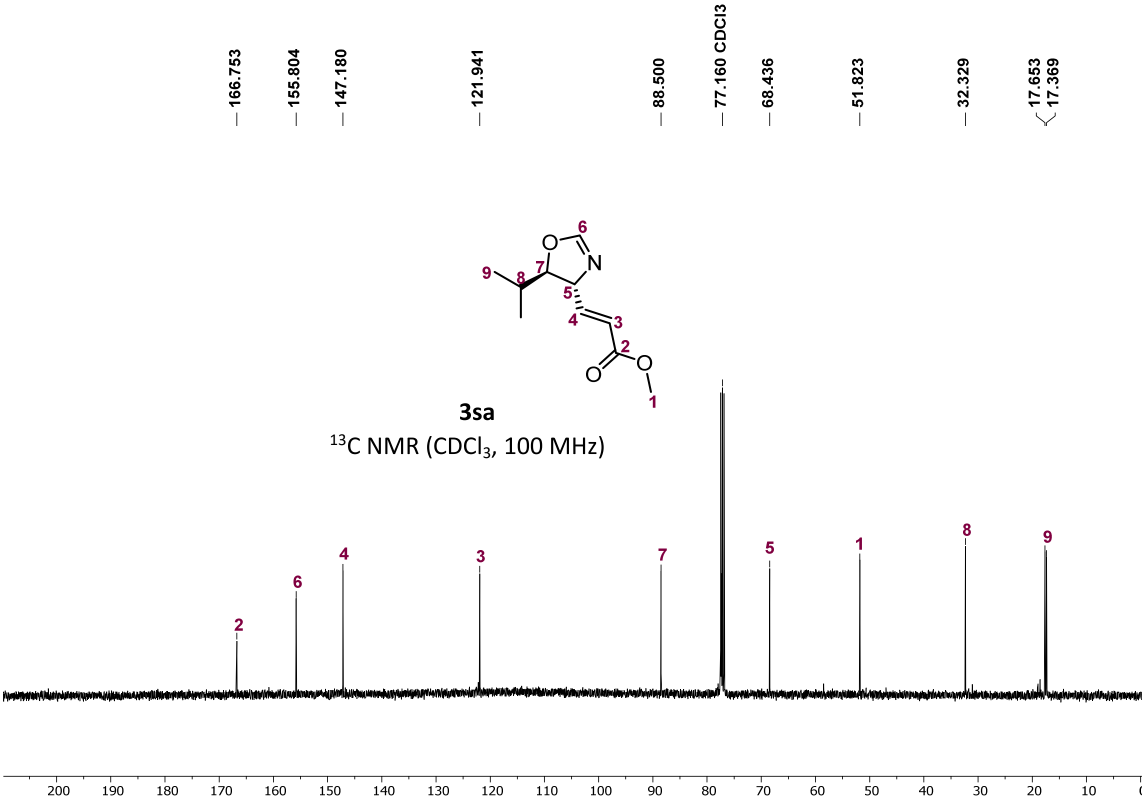
166.753
155.804
147.180
121.941
88.500
77.160 CDCl3
68.436
51.823
32.329
17.653
17.369
3sa
13C NMR (CDCl3, 100 MHz)
200
190
180
170
160
150
140
130
120
110
100
90
80
70
60
50
40
30
20
10

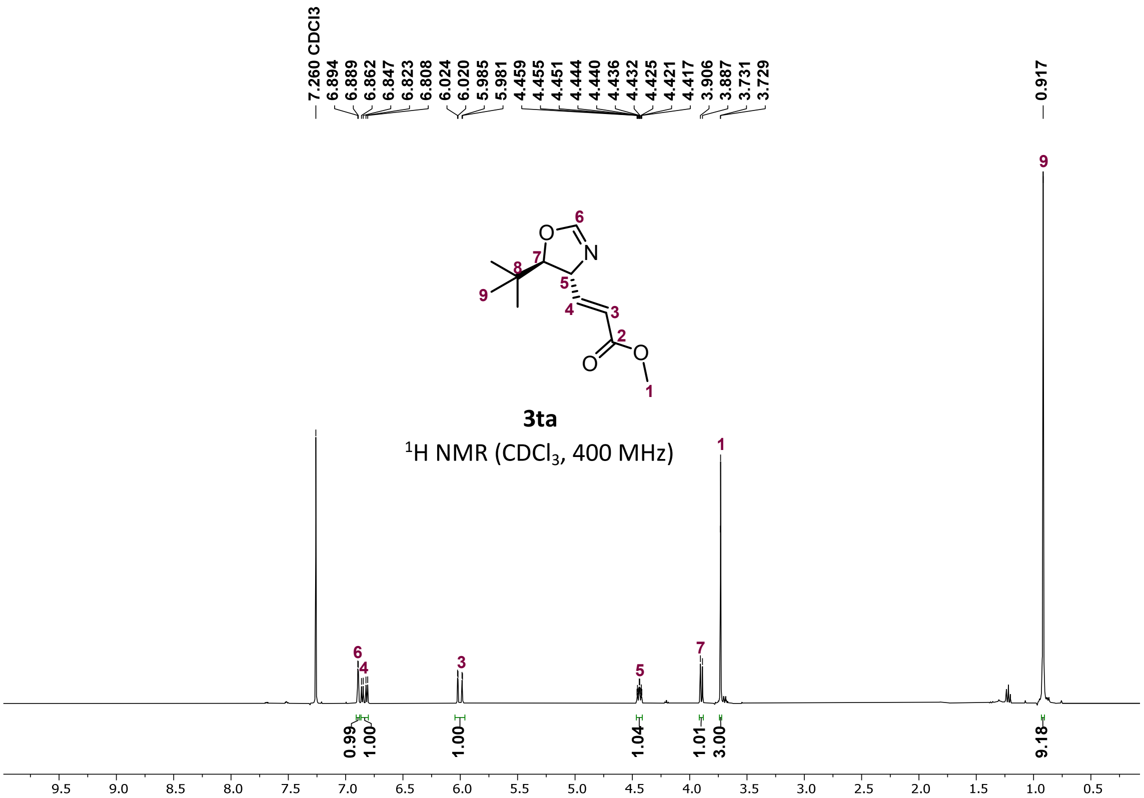
7.260 CDCl3
6.894
6.889
6.862
6.847
6.823
6.808
6.024
6.020
5.985
5.981
4.459
4.455
4.451
4.444
4.440
4.436
4.432
4.425
4.421
4.417
3.906
3.887
3.731
3.729
0.917
3ta
^{1}H NMR ($CDCl_3$, 400 MHz)
0.99
1.00
1.00
1.04
1.01
3.00
9.18
9.5 9.0 8.5 8.0 7.5 7.0 6.5 6.0 5.5 5.0 4.5 4.0 3.5 3.0 2.5 2.0 1.5 1.0 0.5

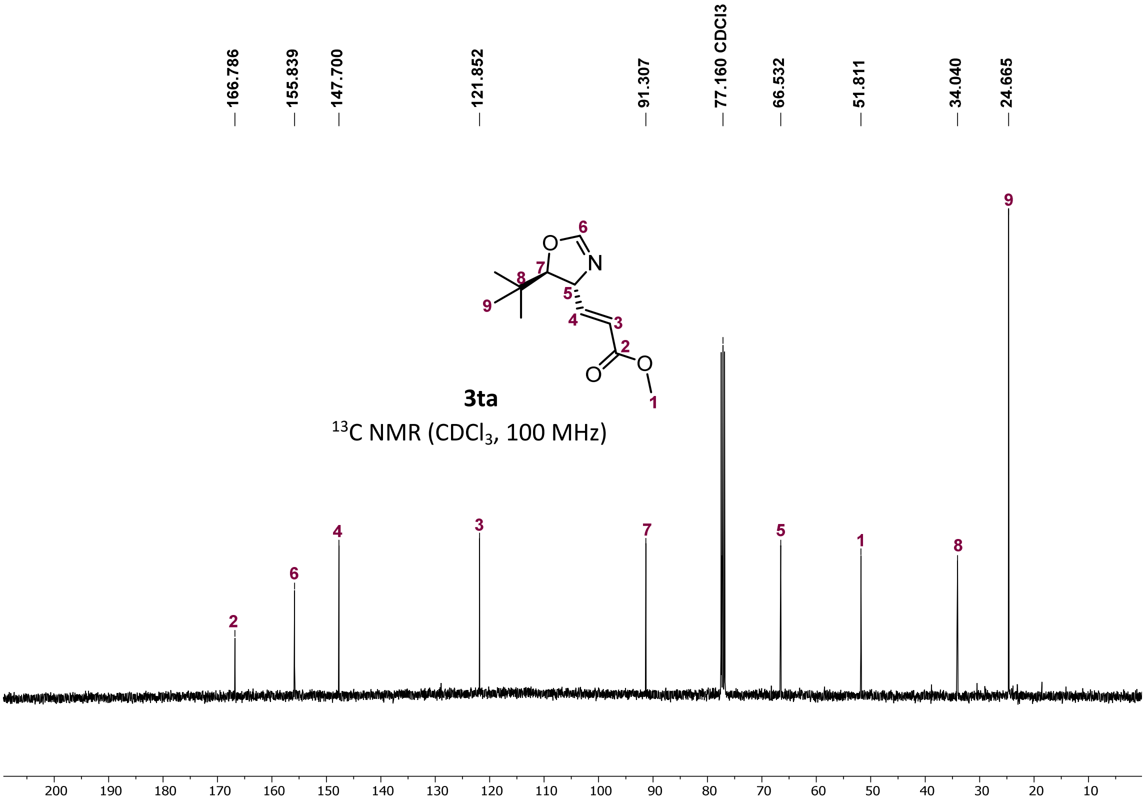
166.786
155.839
147.700
121.852
91.307
77.160 CDCl3
66.532
51.811
34.040
24.665
3ta
^{13}C NMR ($CDCl_3$, 100 MHz)
200 190 180 170 160 150 140 130 120 110 100 90 80 70 60 50 40 30 20 10

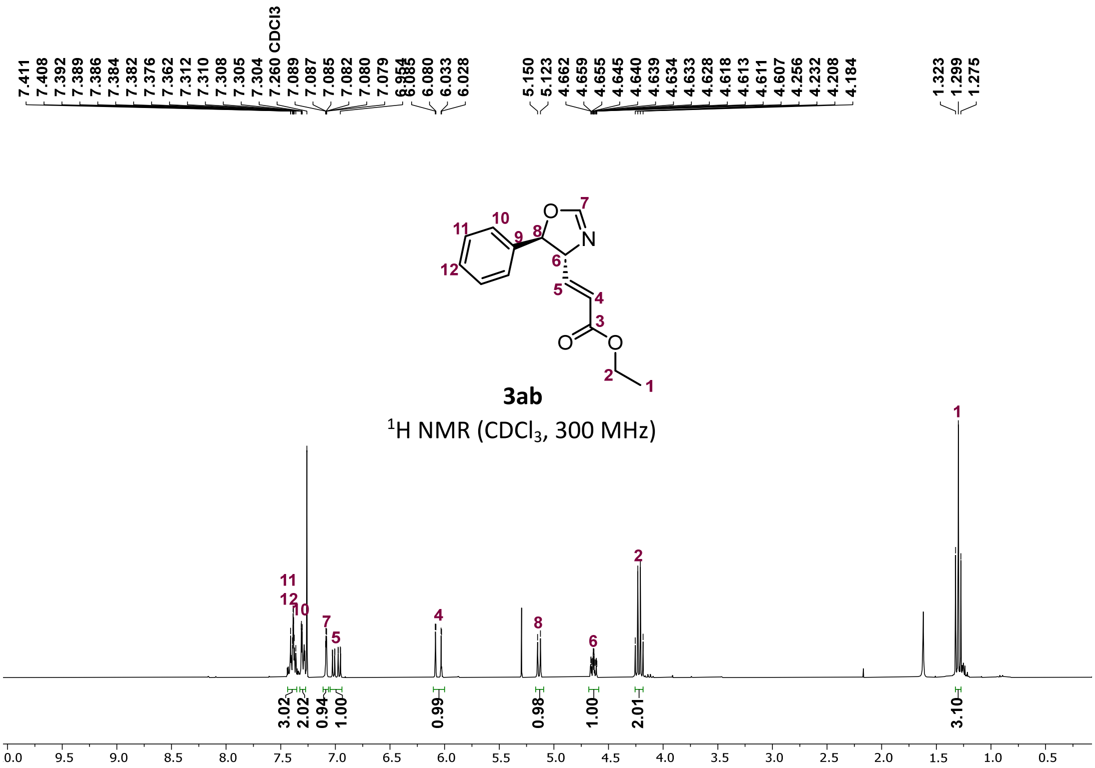
7.411 7.408 7.392 7.389 7.386 7.384 7.382 7.376 7.362 7.312 7.310 7.308 7.305 7.304 7.260 CDCl3 7.089 7.087 7.085 7.082 7.080 7.079 6.954 6.085 6.080 6.033 6.028
5.150 5.123 4.662 4.659 4.655 4.645 4.640 4.639 4.634 4.633 4.628 4.618 4.613 4.611 4.607 4.256 4.232 4.208 4.184
1.323 1.299 1.275
3ab
1H NMR (CDCl3, 300 MHz)
3.02 2.02 0.94 1.00 0.99 0.98 1.00 2.01 3.10
10.0 9.5 9.0 8.5 8.0 7.5 7.0 6.5 6.0 5.5 5.0 4.5 4.0 3.5 3.0 2.5 2.0 1.5 1.0 0.5

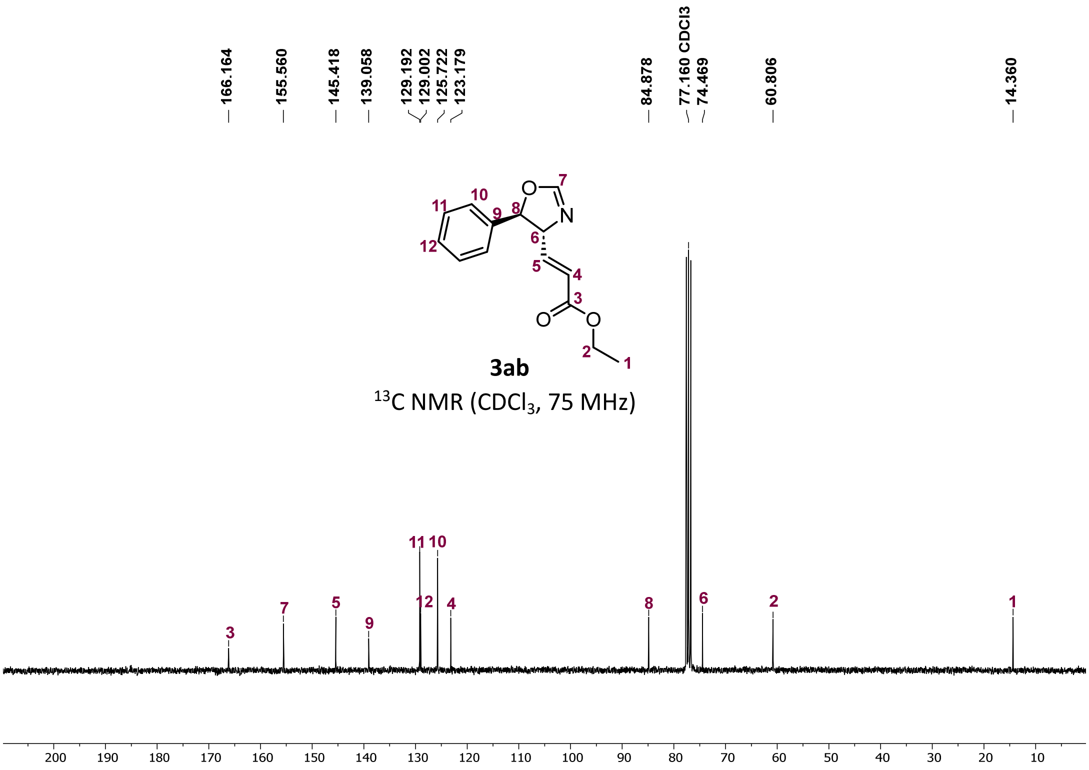
166.164 155.560 145.418 139.058 129.192 129.002 125.722 123.179 84.878 77.160 CDCl3 74.469 60.806 14.360
3ab
13C NMR (CDCl3, 75 MHz)
200 190 180 170 160 150 140 130 120 110 100 90 80 70 60 50 40 30 20 10

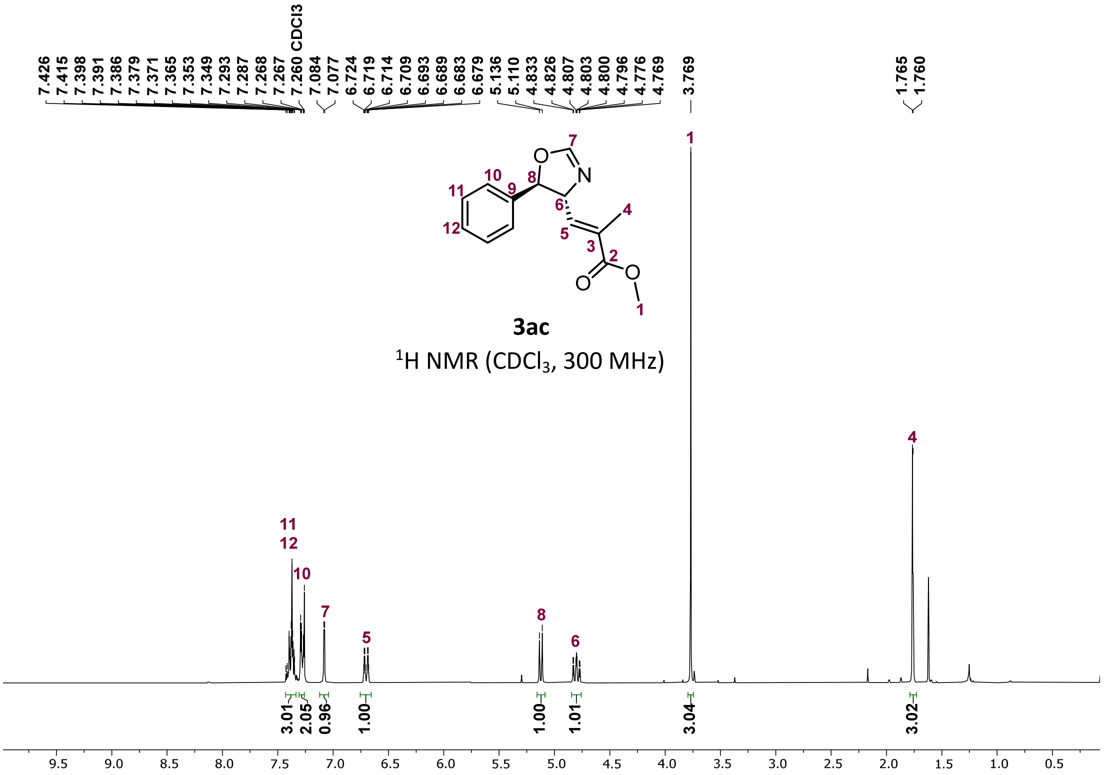
3ac
^{1}H NMR ($CDCl_3$, 300 MHz)

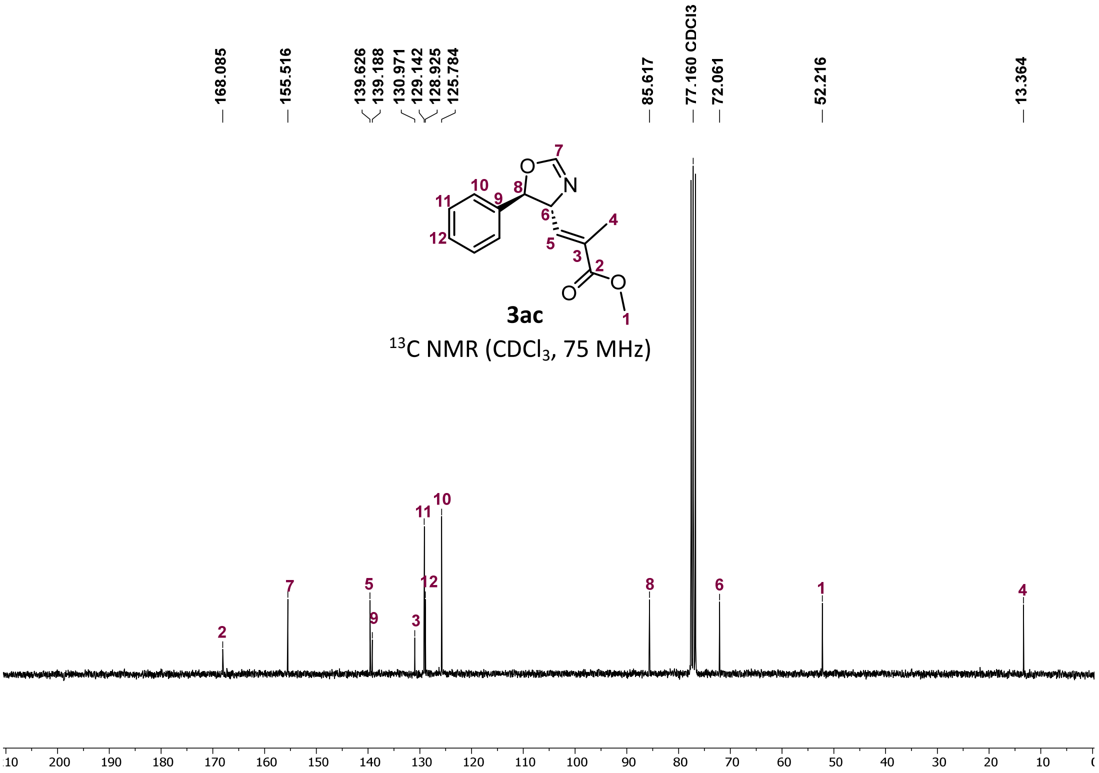
3ac
^{13}C NMR ($CDCl_3$, 75 MHz)

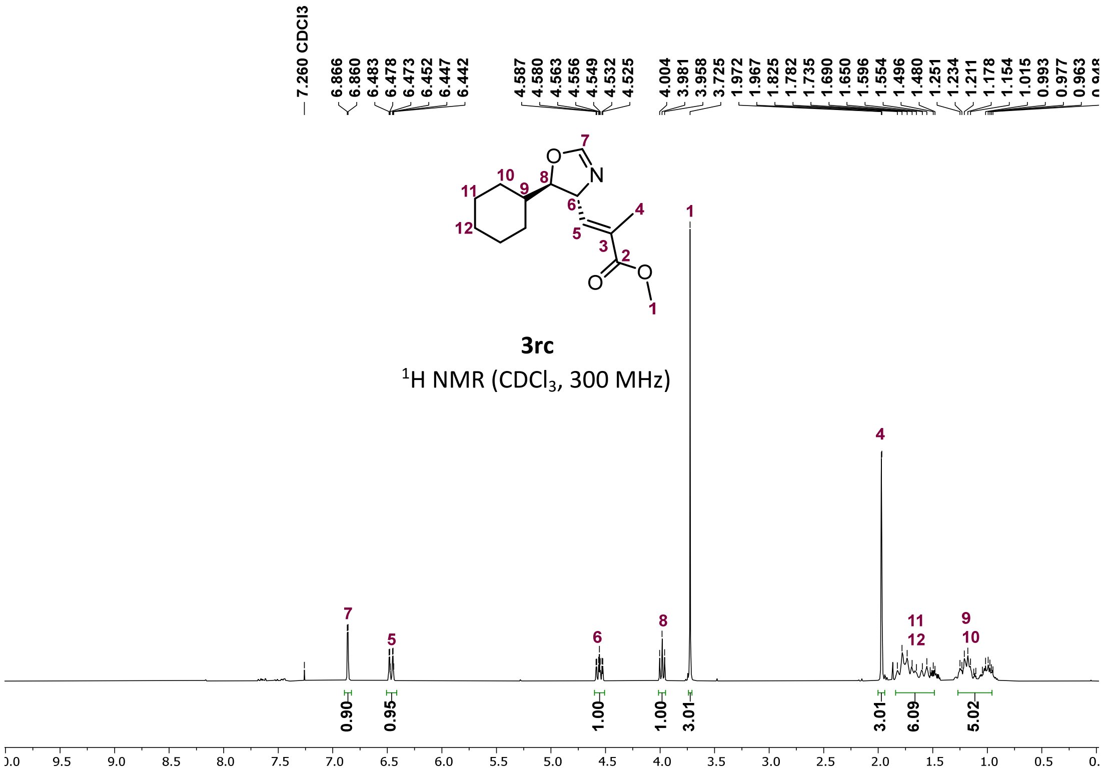
3rc
1H NMR (CDCl3, 300 MHz)

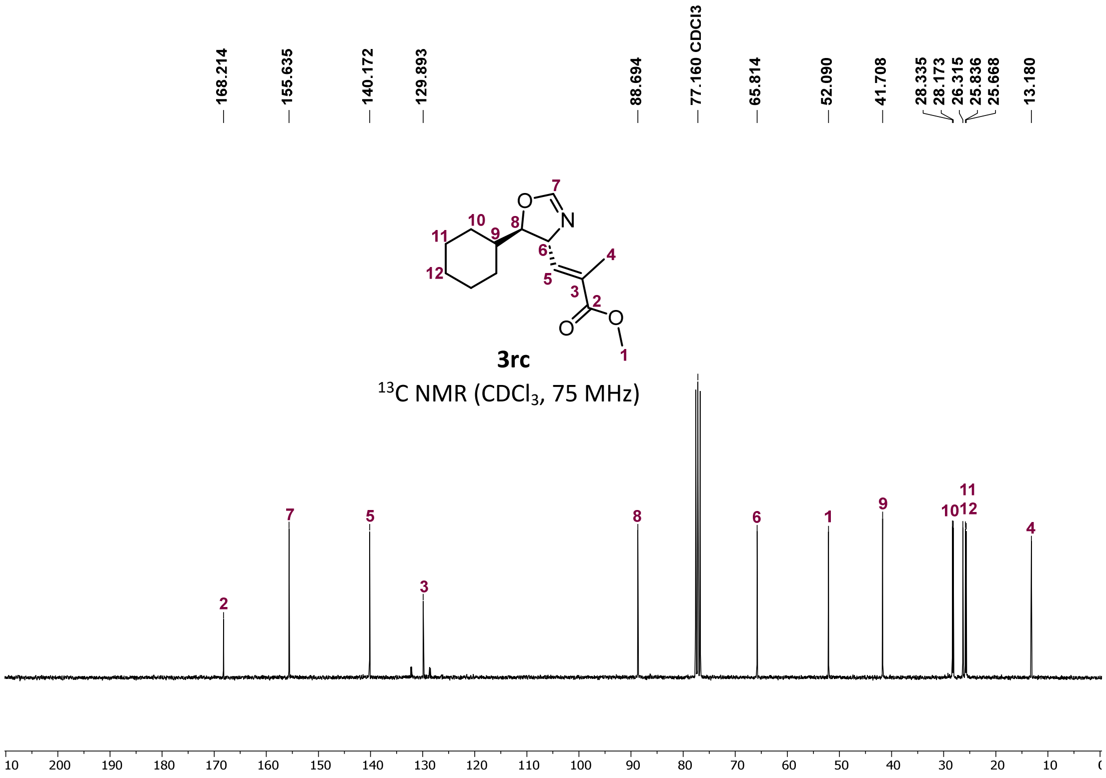
3rc
13C NMR (CDCl3, 75 MHz)

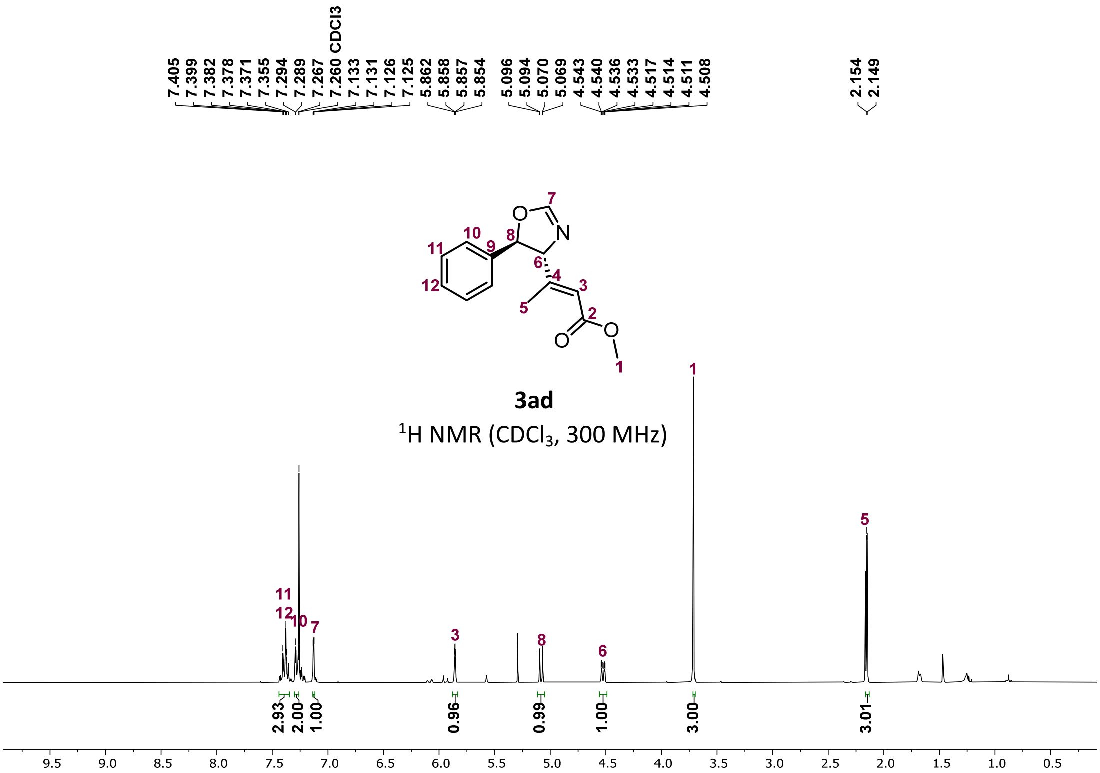

3ad

^{1}H NMR ($CDCl_3$, 300 MHz)

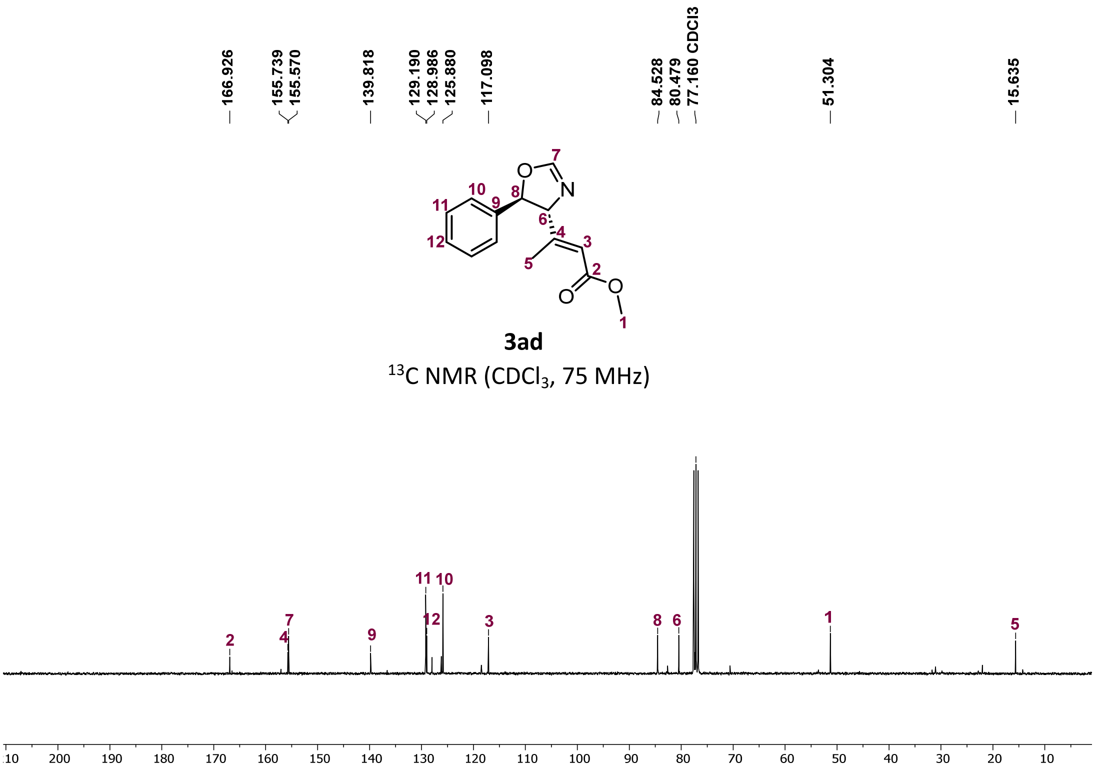

3ad

^{13}C NMR ($CDCl_3$, 75 MHz)

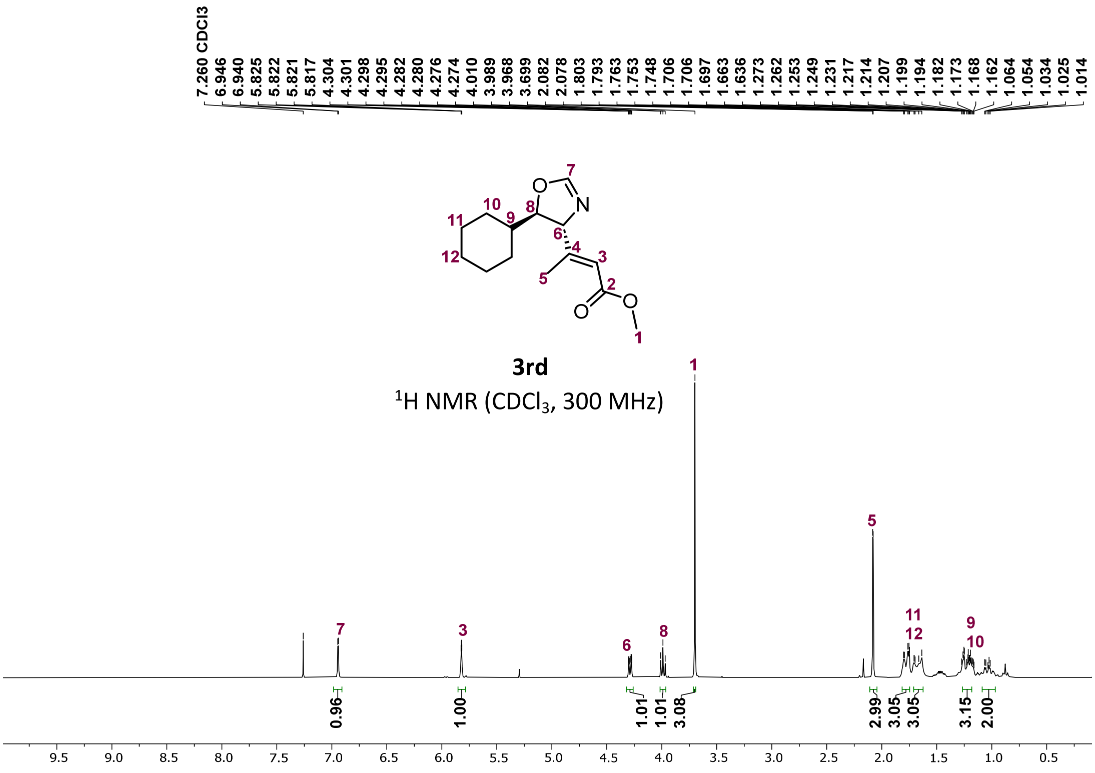

3rd

^{1}H NMR ($CDCl_3$, 300 MHz)

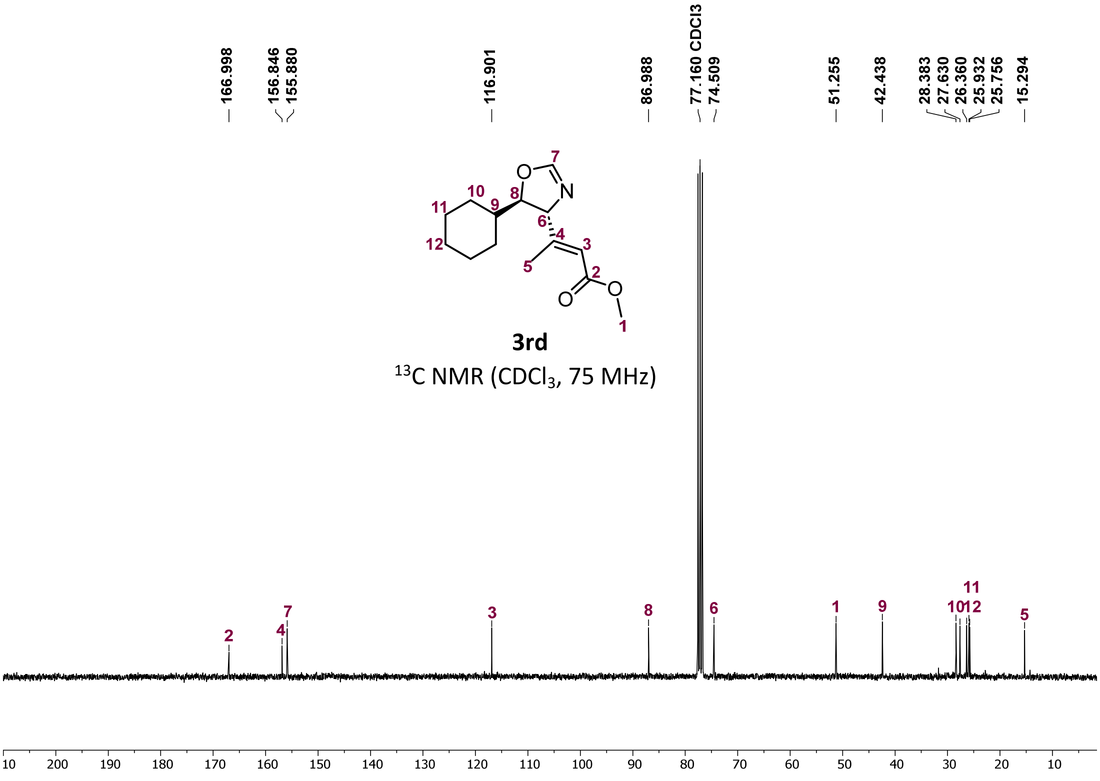

3rd

^{13}C NMR ($CDCl_3$, 75 MHz)

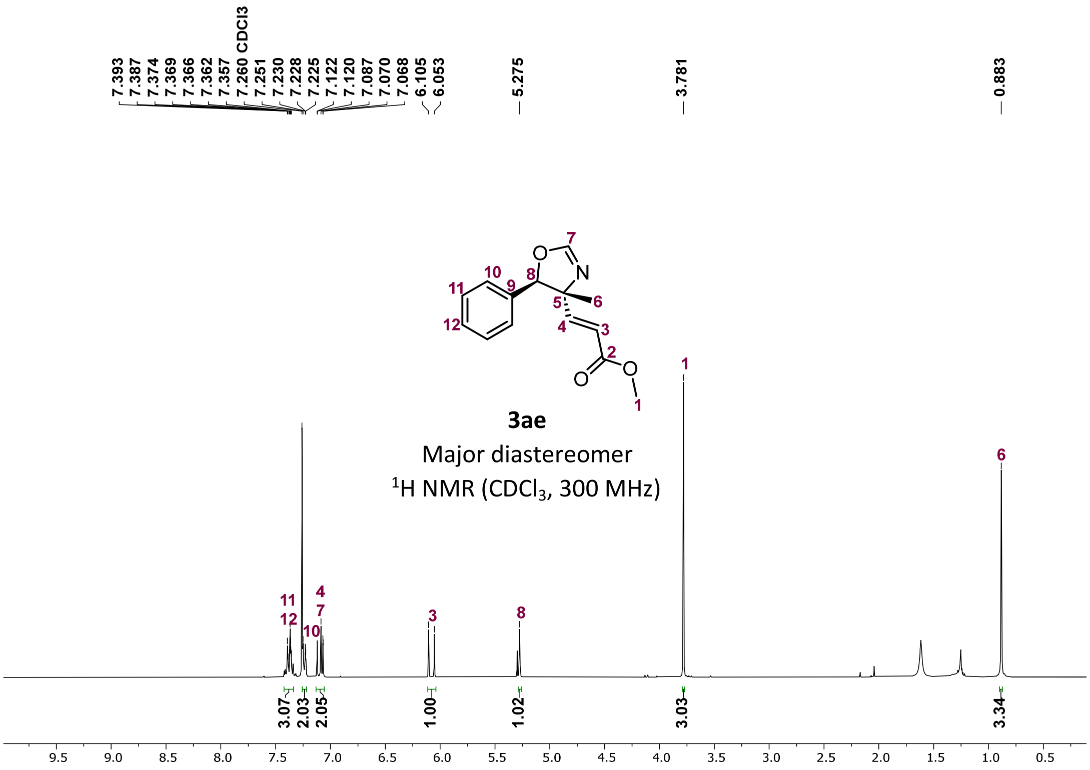
7.393
7.387
7.374
7.369
7.366
7.362
7.357
7.260 CDCl3
7.251
7.230
7.228
7.225
7.122
7.120
7.087
7.070
7.068
6.105
6.053
5.275
3.781
0.883
3ae
Major diastereomer
1H NMR (CDCl3, 300 MHz)
3.07
2.03
2.05
1.00
1.02
3.03
3.34

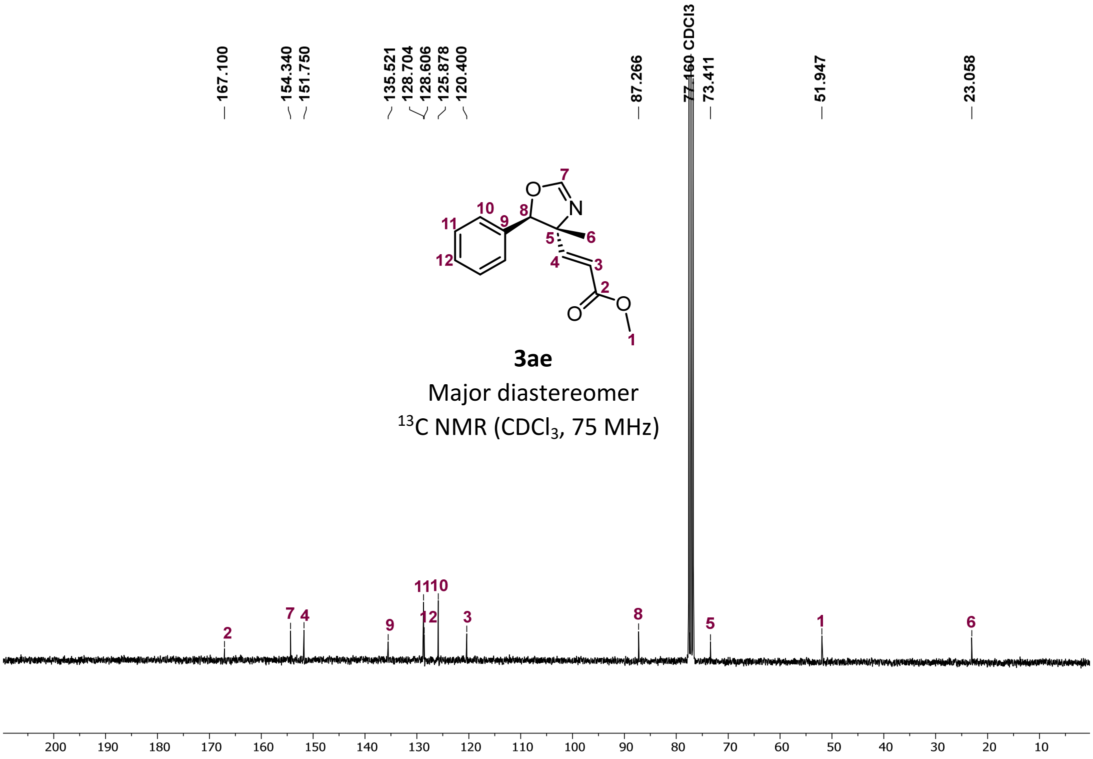
167.100
154.340
151.750
135.521
128.704
128.606
125.878
120.400
87.266
77.460 CDCl3
73.411
51.947
23.058
3ae
Major diastereomer
13C NMR (CDCl3, 75 MHz)

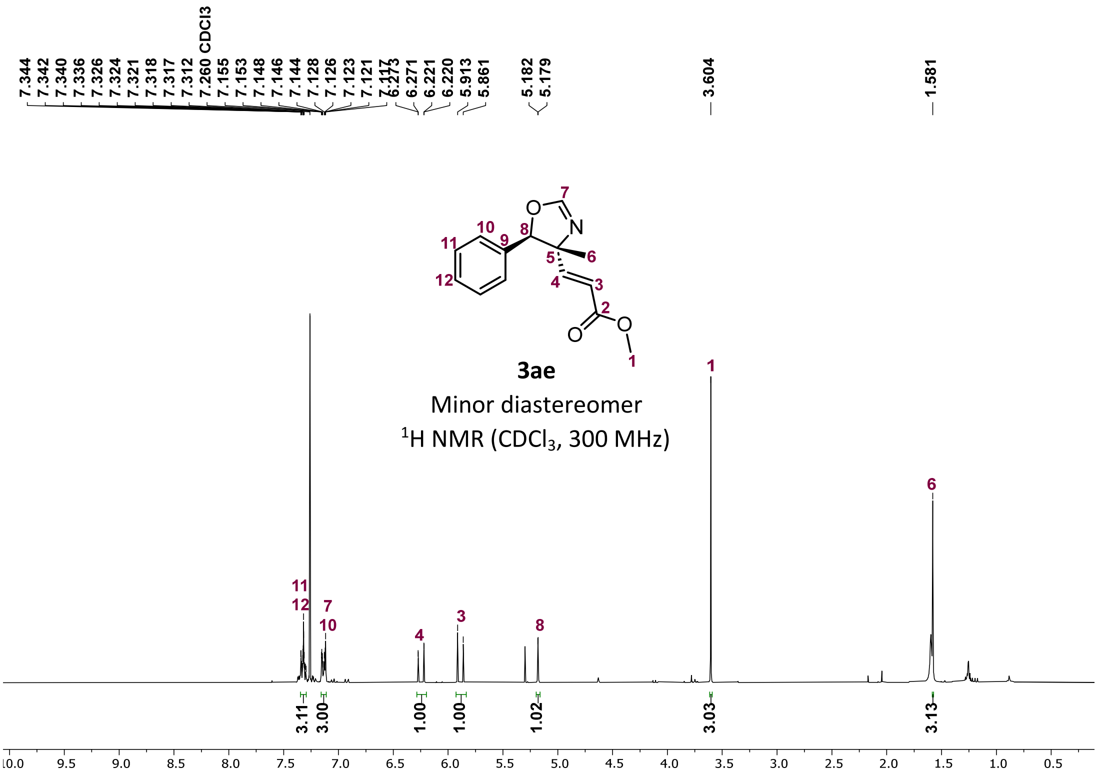
7.344 7.342 7.340 7.336 7.326 7.324 7.321 7.318 7.317 7.312 7.260 CDCl3 7.155 7.153 7.148 7.146 7.144 7.128 7.126 7.123 7.121 7.117 6.273 6.271 6.221 6.220 5.913 5.861 5.182 5.179 3.604 1.581
3ae
Minor diastereomer
¹H NMR (CDCl₃, 300 MHz)
3.11 3.00 1.00 1.00 1.02 3.03 3.13

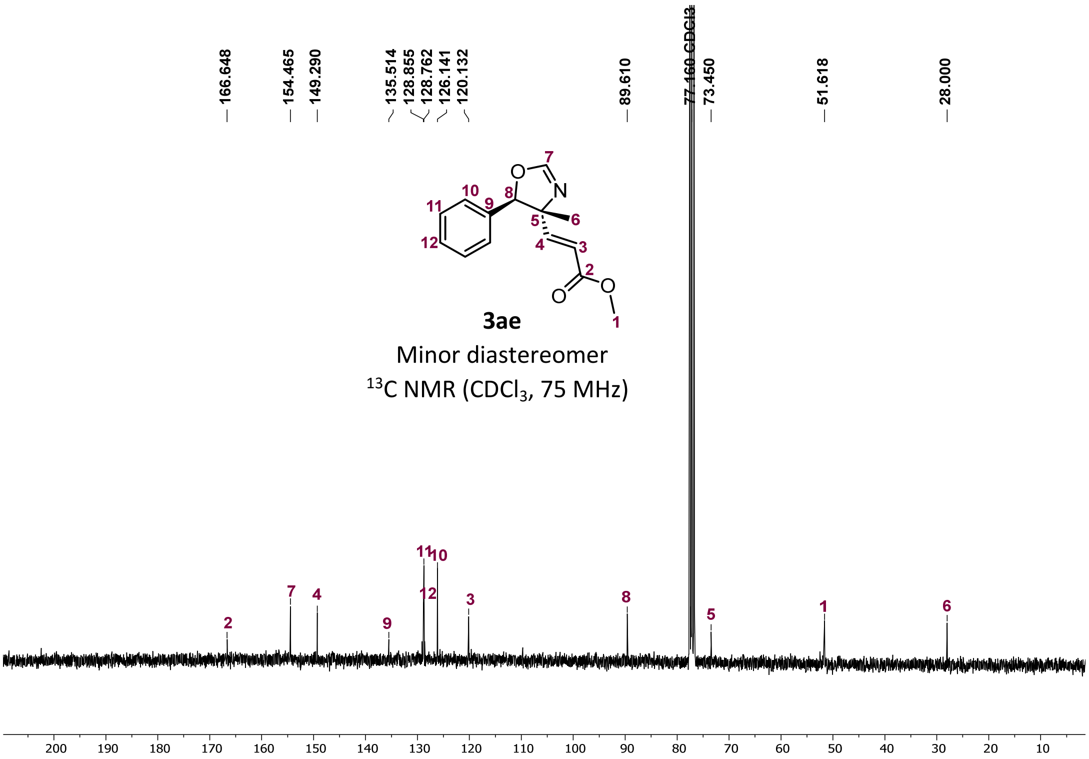
166.648 154.465 149.290 135.514 128.855 128.762 126.141 120.132 89.610 77.160 CDCl3 73.450 51.618 28.000
3ae
Minor diastereomer
¹³C NMR (CDCl₃, 75 MHz)

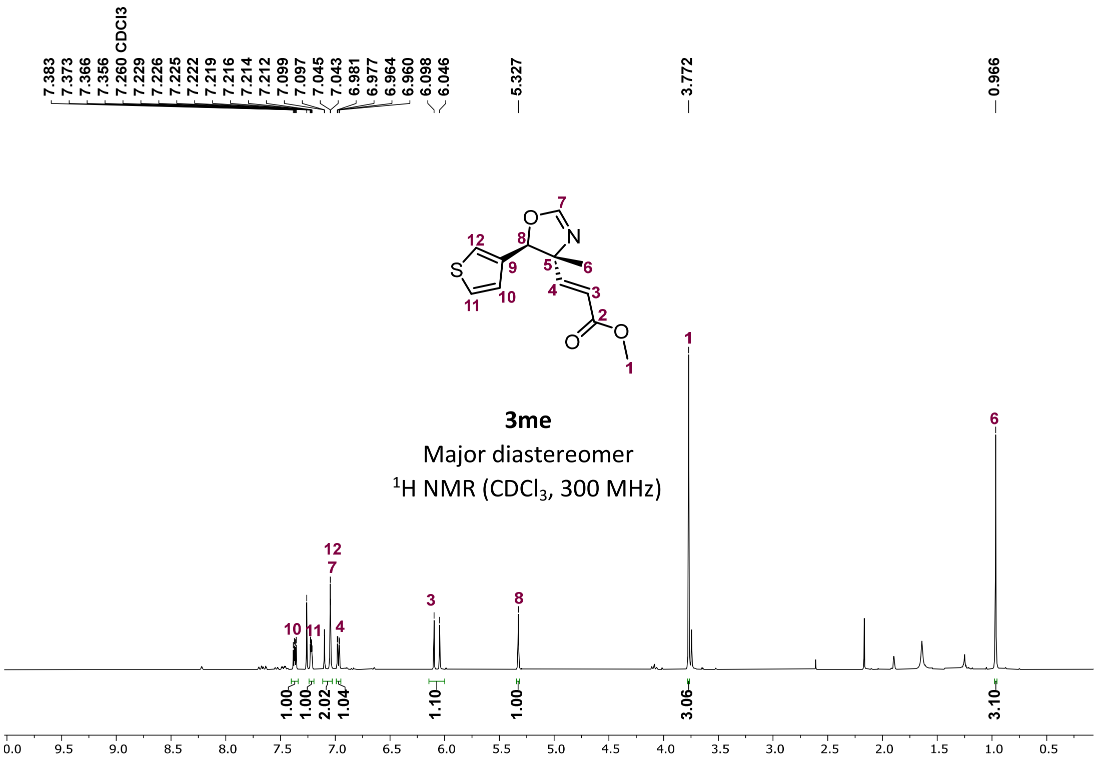

7.383
7.373
7.366
7.356
7.260 CDCl3
7.229
7.226
7.225
7.222
7.219
7.216
7.214
7.212
7.099
7.097
7.045
7.043
6.981
6.977
6.964
6.960
6.098
6.046
5.327
3.772
0.966
3me
Major diastereomer
1H NMR (CDCl3, 300 MHz)
1.00
1.00
2.02
1.04
1.10
1.00
3.06
3.10

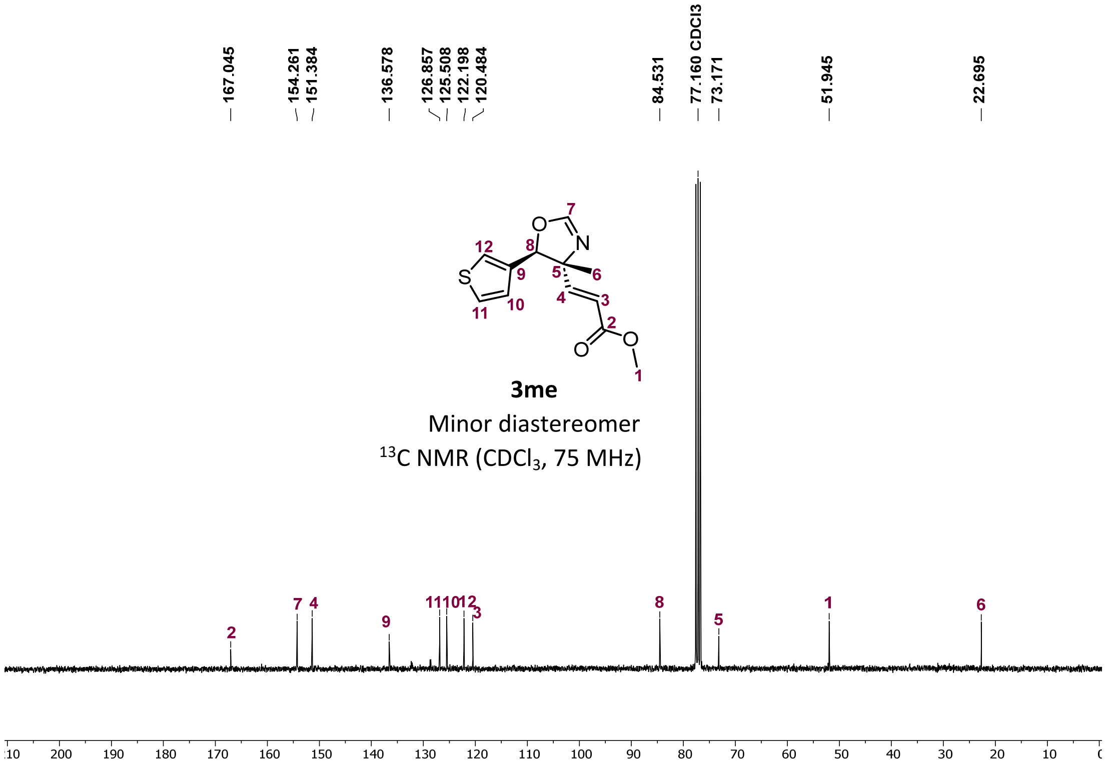

167.045
154.261
151.384
136.578
126.857
125.508
122.198
120.484
84.531
77.160 CDCl3
73.171
51.945
22.695
3me
Minor diastereomer
13C NMR (CDCl3, 75 MHz)

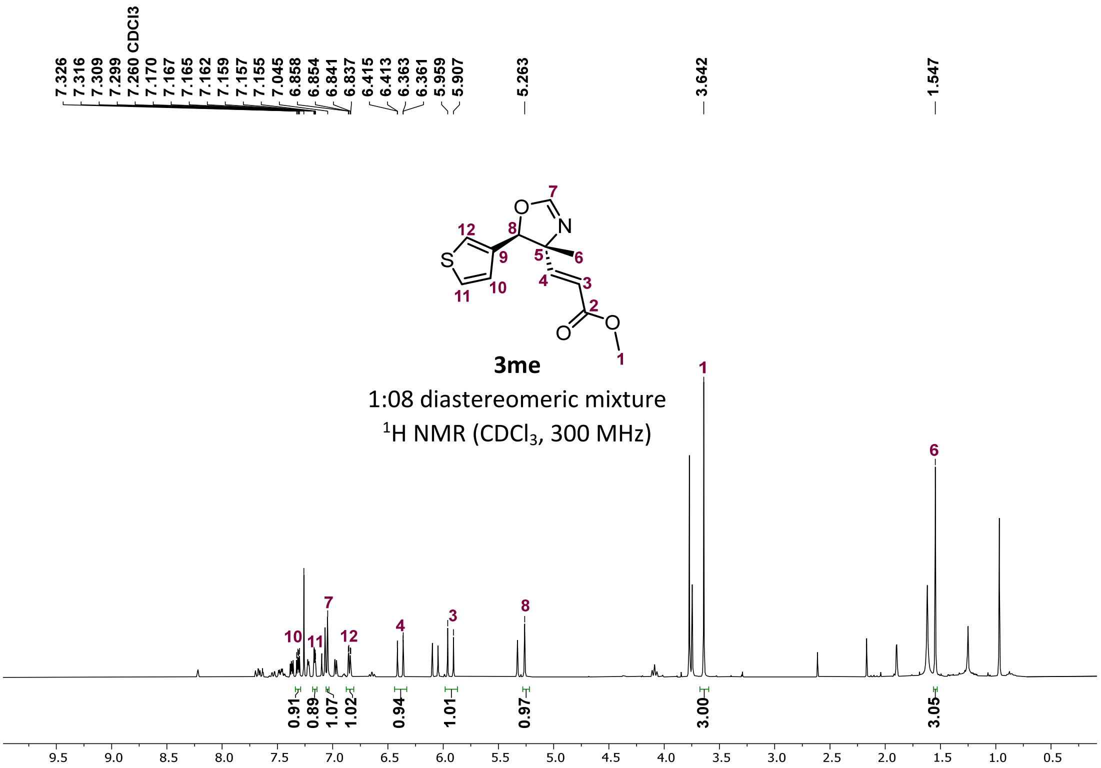

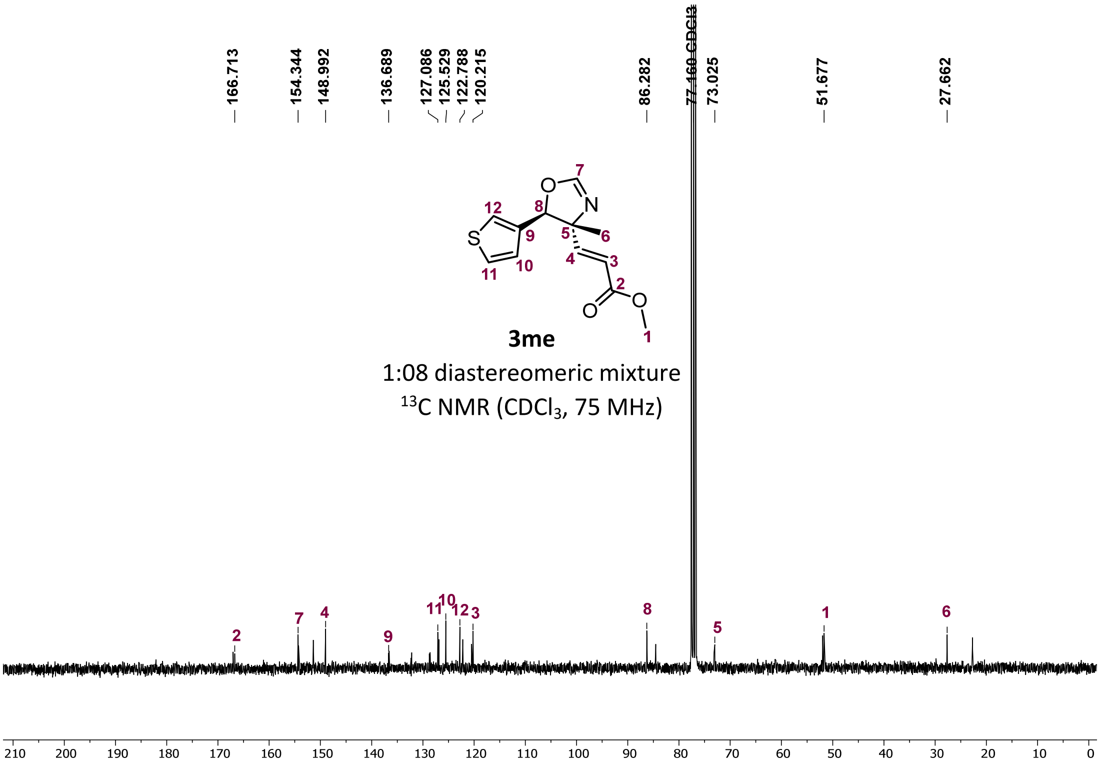

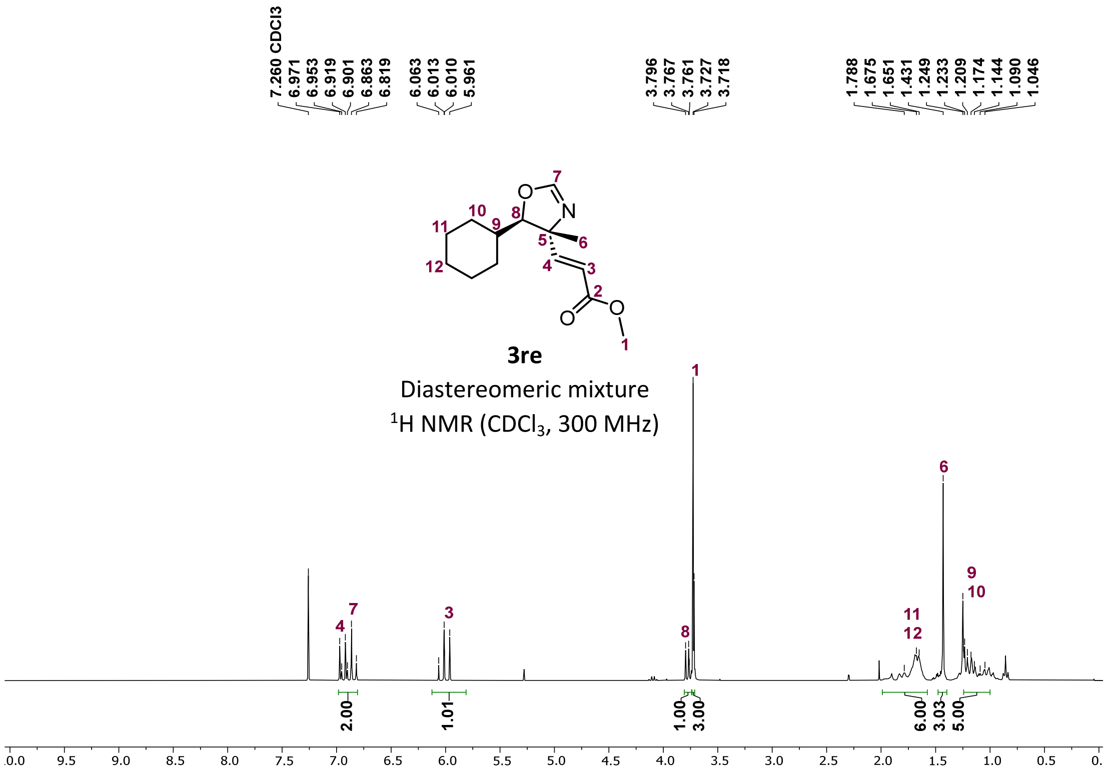
7.260 CDCl3
6.971
6.953
6.919
6.901
6.863
6.819
6.063
6.013
6.010
5.961
3.796
3.767
3.761
3.727
3.718
1.788
1.675
1.651
1.431
1.249
1.233
1.209
1.174
1.144
1.090
1.046
3re
Diastereomeric mixture
1H NMR (CDCl3, 300 MHz)
2.00
1.01
1.00
3.00
6.00
3.03
5.00

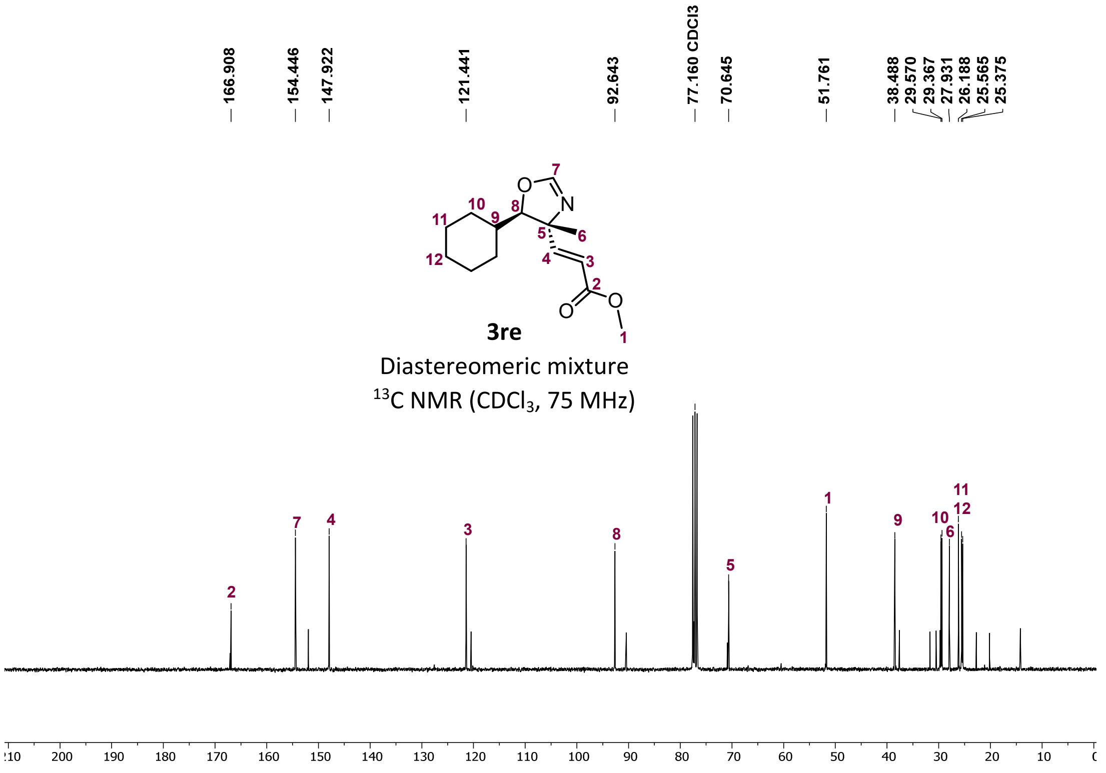
166.908
154.446
147.922
121.441
92.643
77.160 CDCl3
70.645
51.761
38.488
29.570
29.367
27.931
26.188
25.565
25.375
3re
Diastereomeric mixture
13C NMR (CDCl3, 75 MHz)

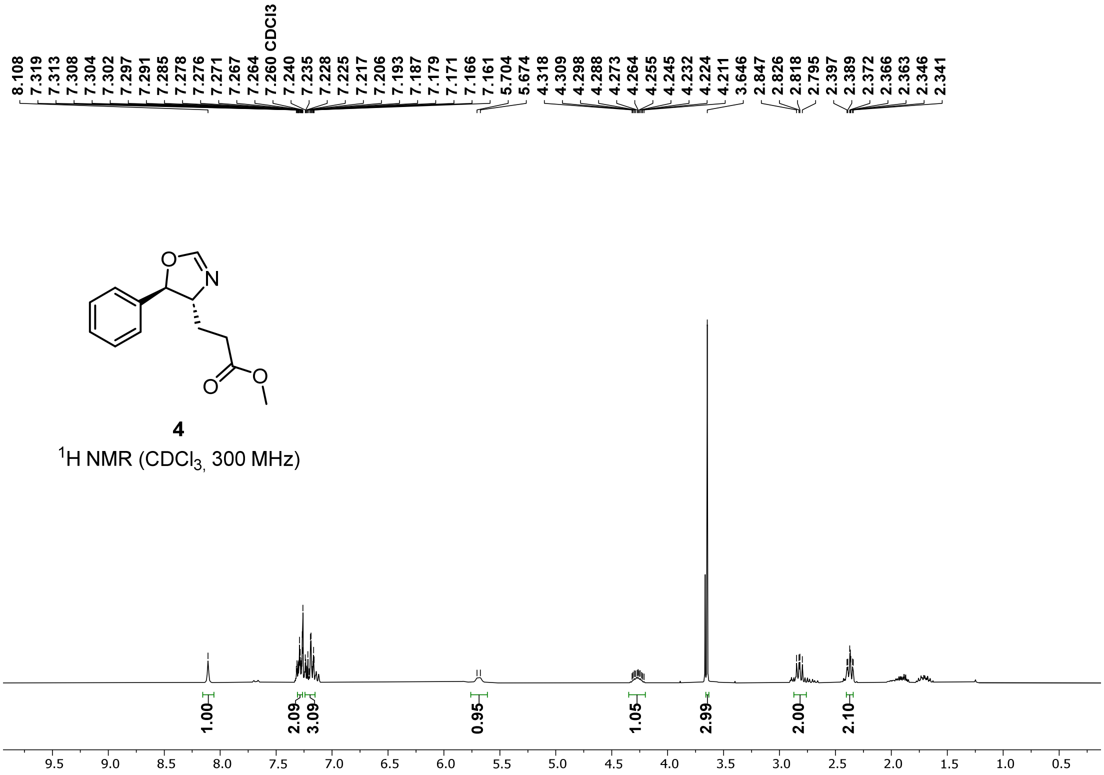

4

^{1}H NMR ($CDCl_3$, 300 MHz)

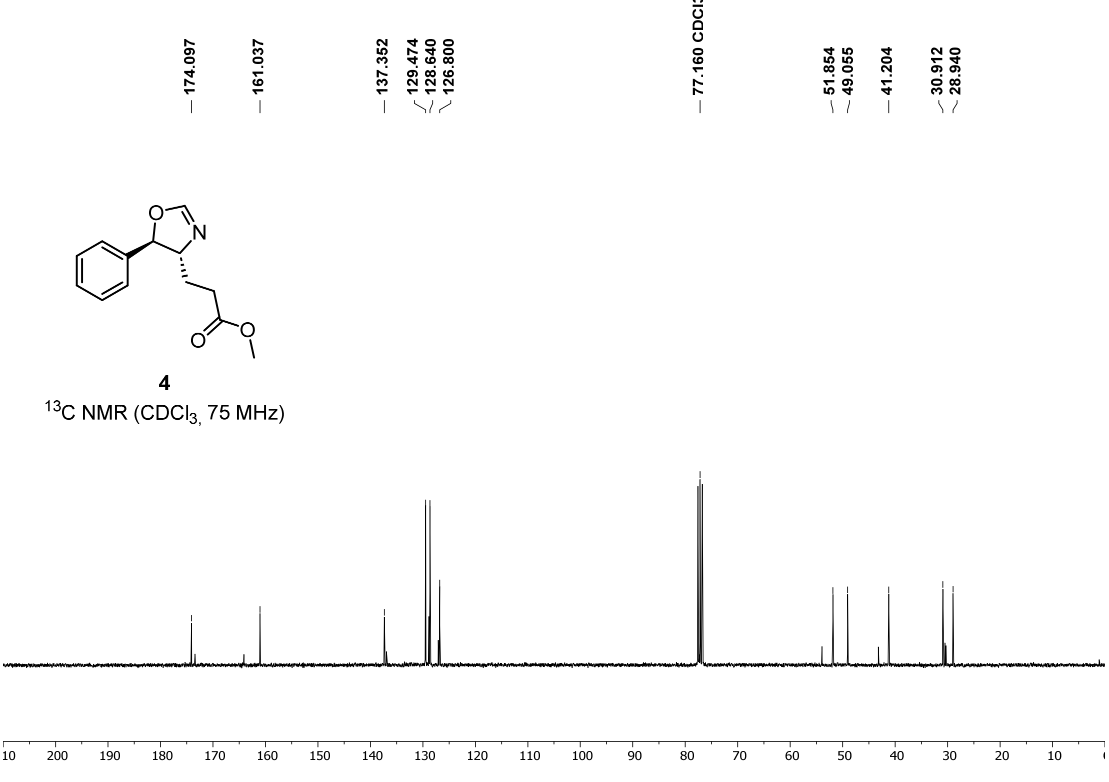

4

^{13}C NMR ($CDCl_3$, 75 MHz)

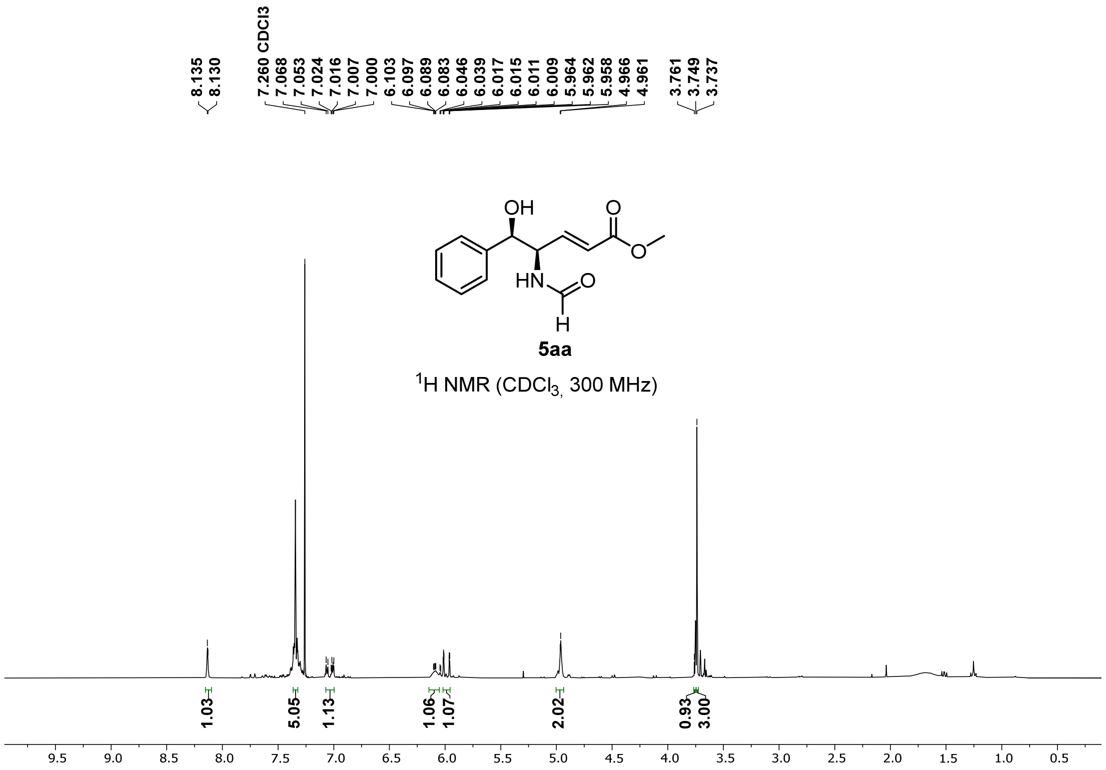
8.135
8.130
7.260 CDCl3
7.068
7.053
7.024
7.016
7.007
7.000
6.103
6.097
6.089
6.083
6.046
6.039
6.017
6.015
6.011
6.009
5.964
5.962
5.958
4.966
4.961
3.761
3.749
3.737
OH
O
O
HN
O
H
5aa
1H NMR (CDCl3, 300 MHz)
1.03
5.05
1.13
1.06
1.07
2.02
0.93
3.00
9.5
9.0
8.5
8.0
7.5
7.0
6.5
6.0
5.5
5.0
4.5
4.0
3.5
3.0
2.5
2.0
1.5
1.0
0.5

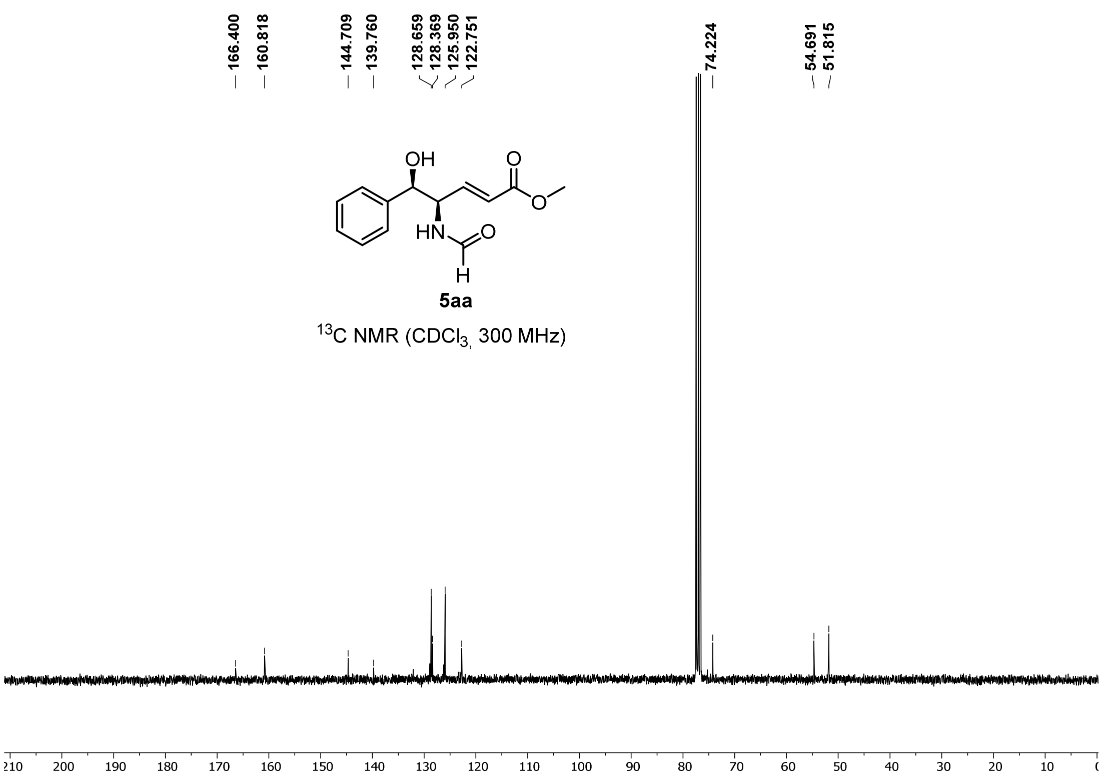
166.400
160.818
144.709
139.760
128.659
128.369
125.950
122.751
74.224
54.691
51.815
OH
O
O
HN
O
H
5aa
13C NMR (CDCl3, 300 MHz)
200
190
180
170
160
150
140
130
120
110
100
90
80
70
60
50
40
30
20
10

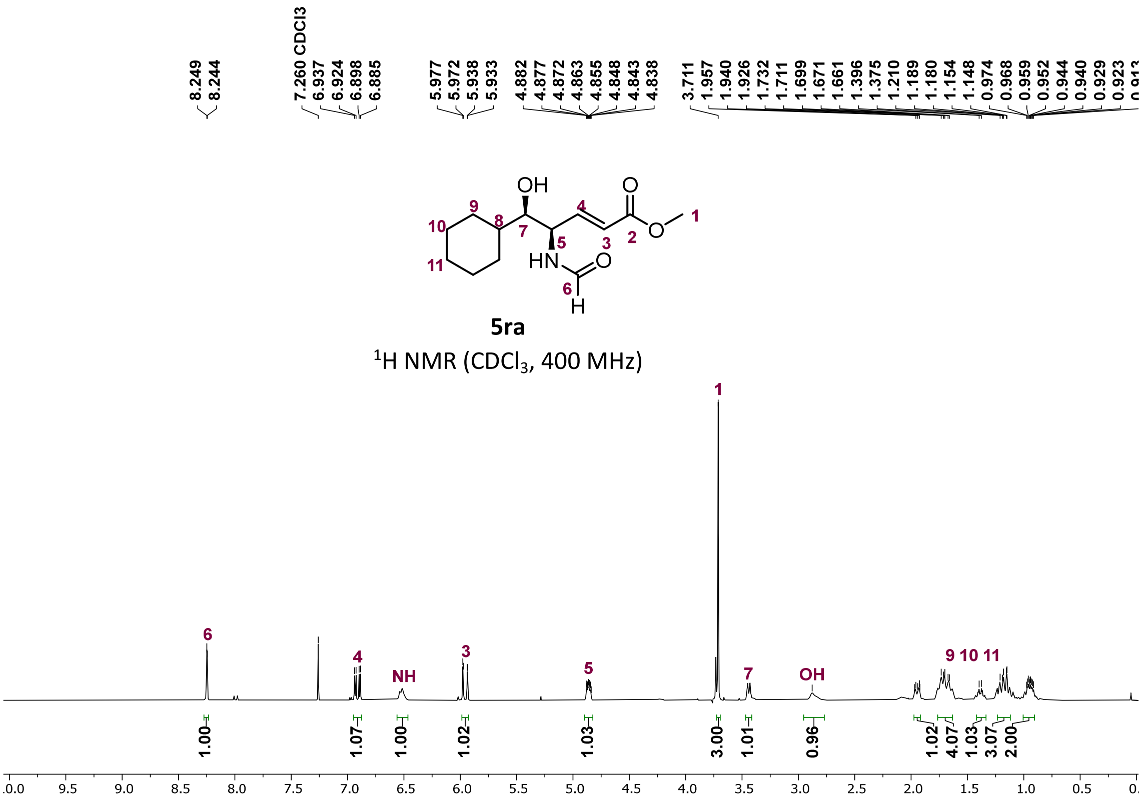
8.249
8.244
7.260 CDCl3
6.937
6.924
6.898
6.885
5.977
5.972
5.938
5.933
4.882
4.877
4.872
4.863
4.855
4.848
4.843
4.838
3.711
1.957
1.940
1.926
1.732
1.711
1.699
1.671
1.661
1.396
1.375
1.210
1.189
1.180
1.154
1.148
0.974
0.968
0.959
0.952
0.944
0.940
0.929
0.923
OH
HN
O
H
5ra
1H NMR (CDCl3, 400 MHz)
6
4
NH
3
5
1
7
OH
9 10 11
1.00
1.07
1.00
1.02
1.03
3.00
1.01
0.96
1.02
4.07
1.03
3.07
2.00

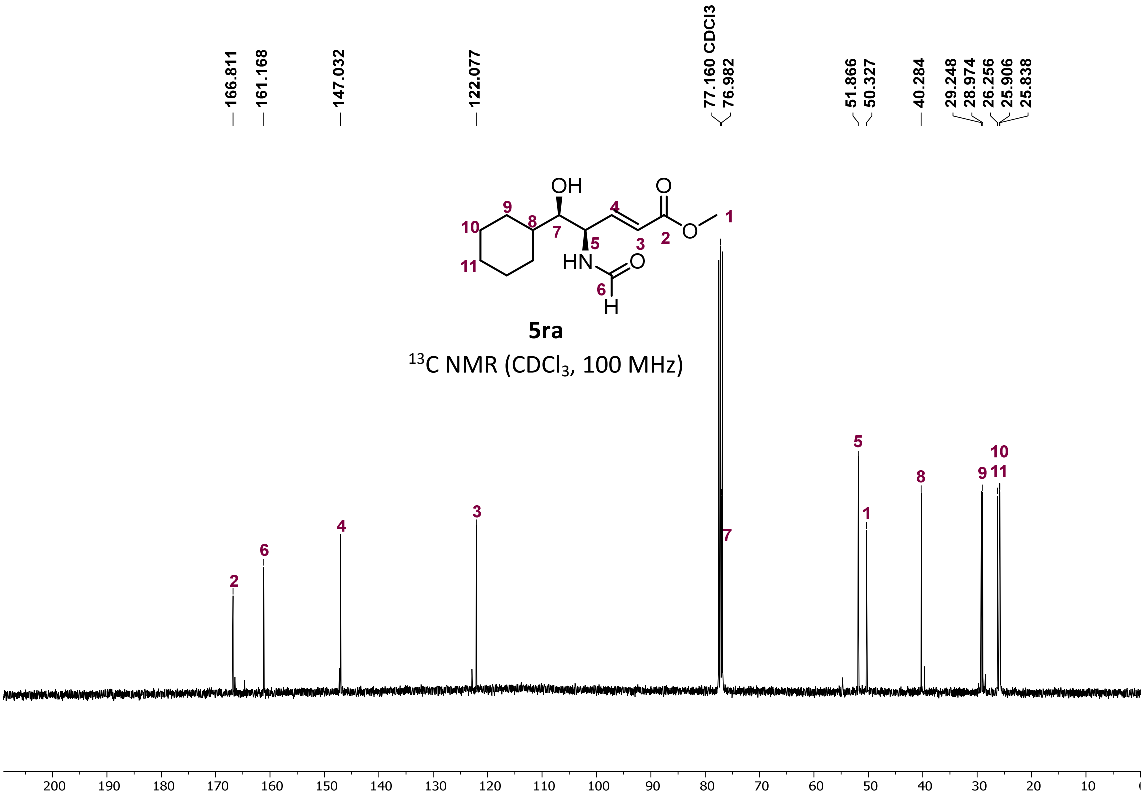
166.811
161.168
147.032
122.077
77.160 CDCl3
76.982
51.866
50.327
40.284
29.248
28.974
26.256
25.906
25.838
OH
HN
O
H
5ra
13C NMR (CDCl3, 100 MHz)
2
6
4
3
7
5
1
8
9
10
11

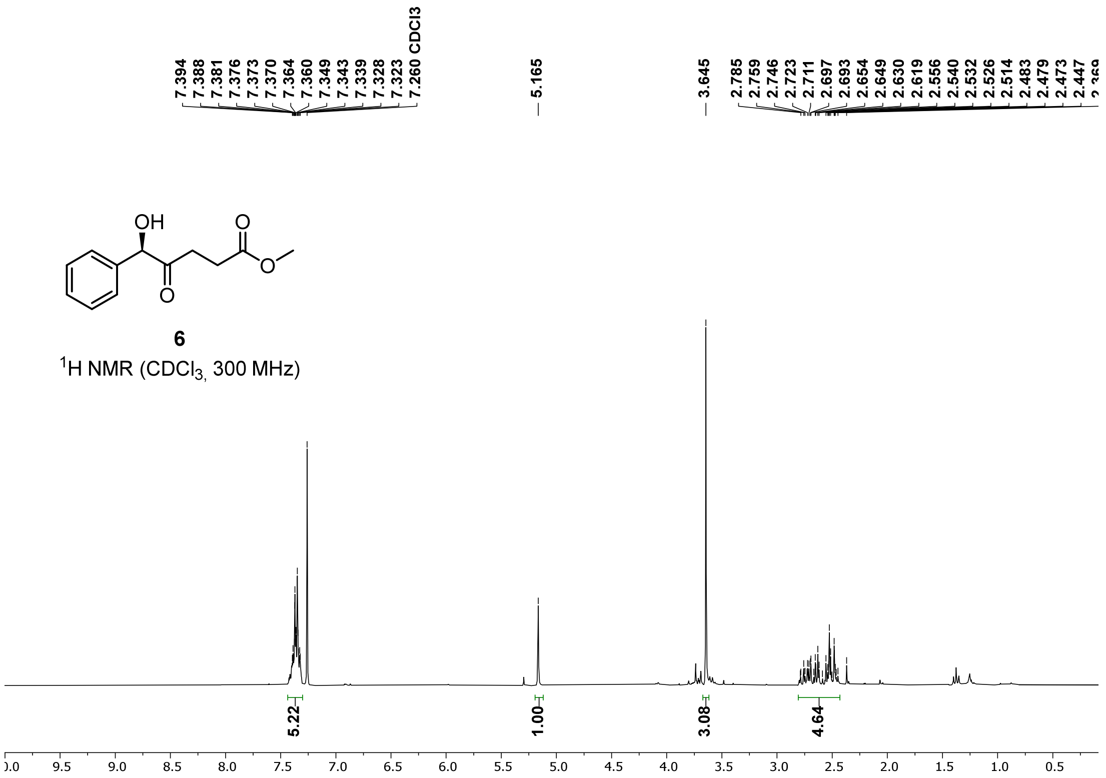

6

^{1}H NMR (CDCl$_3$, 300 MHz)

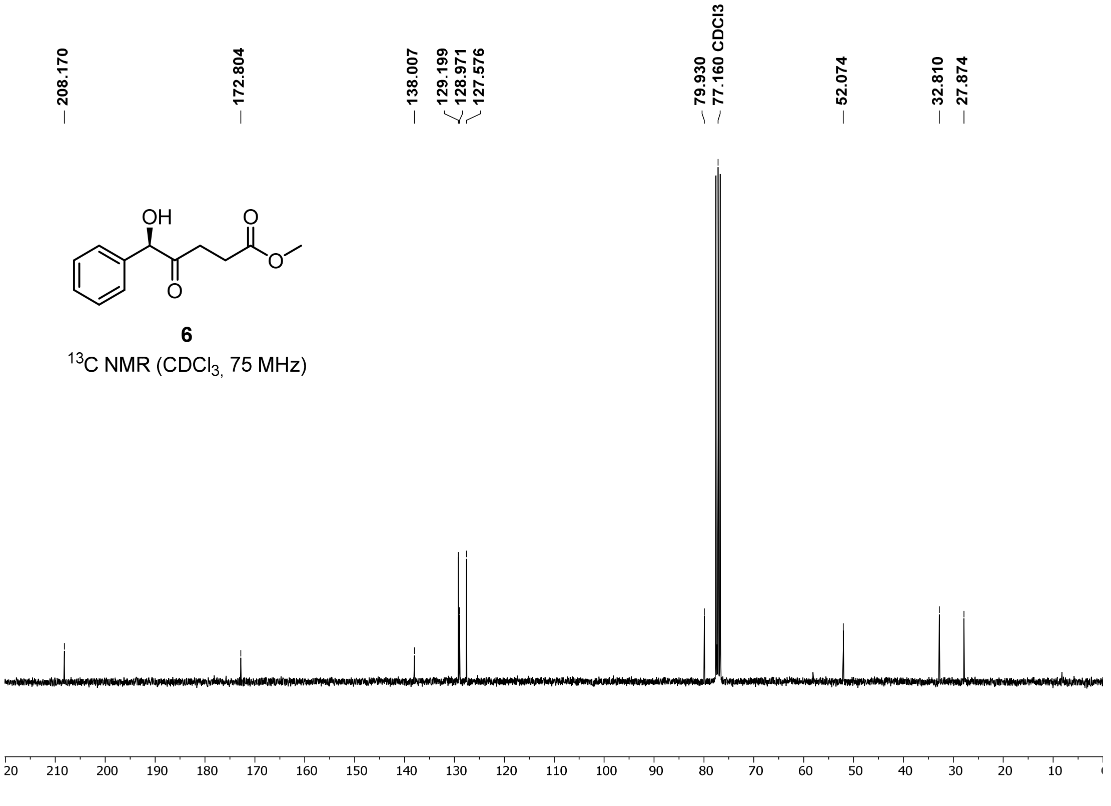

6

^{13}C NMR (CDCl$_3$, 75 MHz)

HPLC traces for compounds 3-6.

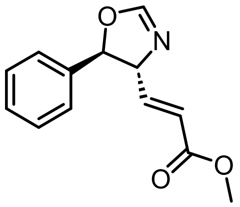

Racemic mixture 3aa

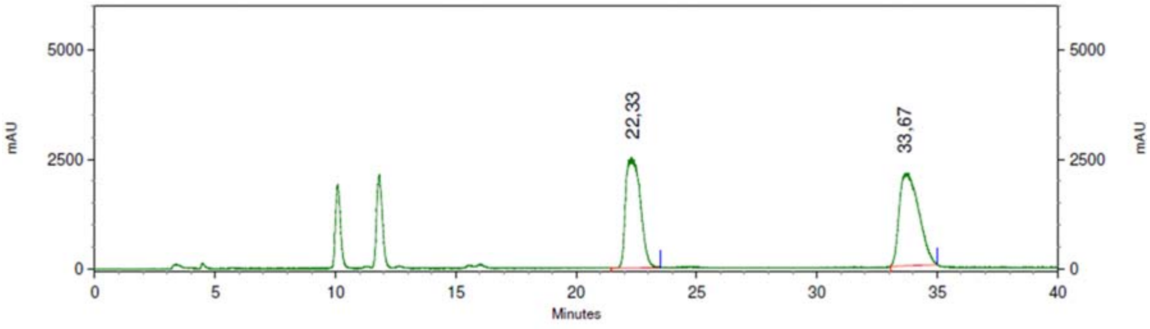

27: 202 nm, 4 nm
Results

Retention Time	Area	Area Percent
22,33	425998331	47,272
33,67	475158192	52,728

Enantioenriched compound 3aa

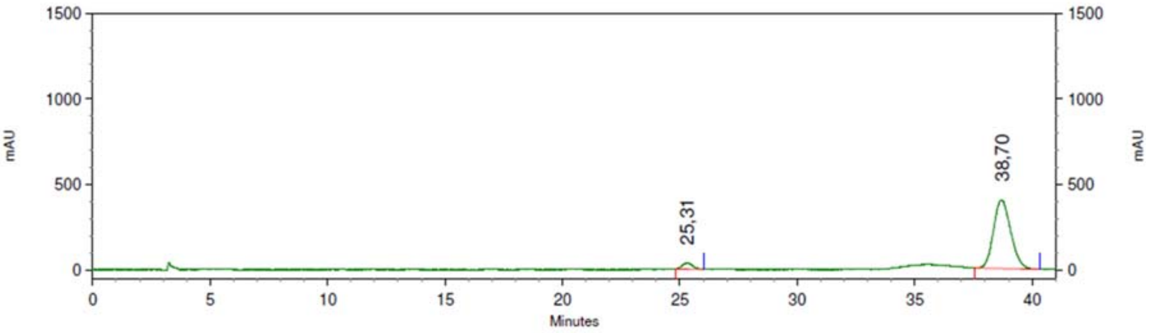

27: 202 nm, 4 nm
Results

Retention Time	Area	Area Percent
25,31	4493515	5,157
38,70	82645518	94,843

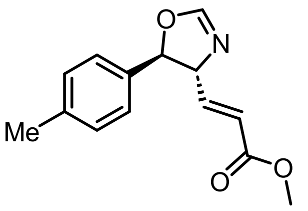

Racemic mixture 3ba

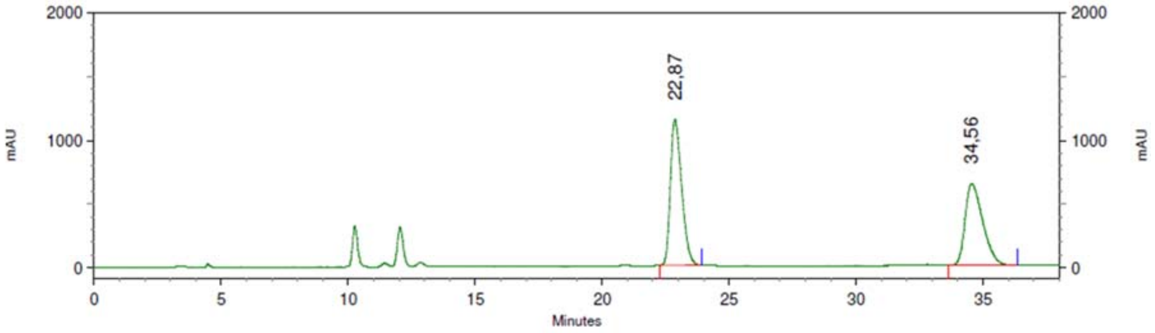

11: 231 nm, 4 nm
Results

Retention Time	Area	Area Percent
22,87	141417422	53,068
34,56	125065021	46,932

Enantioenriched compound 3ba

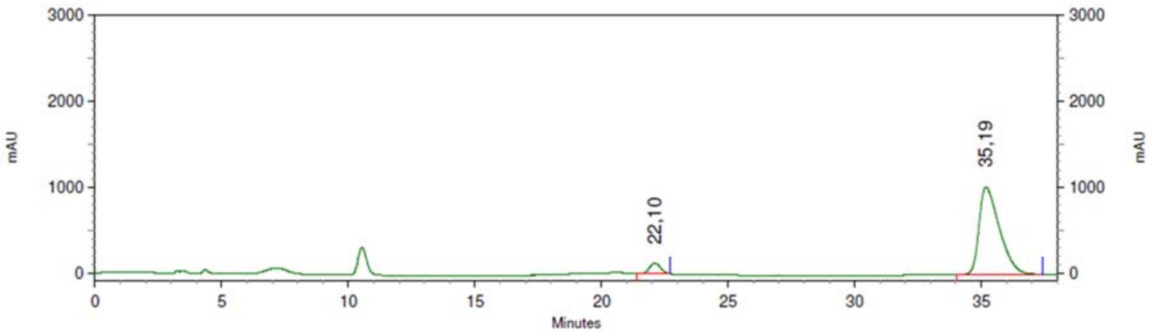

11: 231 nm, 4 nm
Results

Retention Time	Area	Area Percent
22,10	15346343	6,329
35,19	227114926	93,671

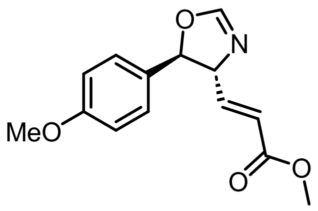

Racemic mixture 3ca

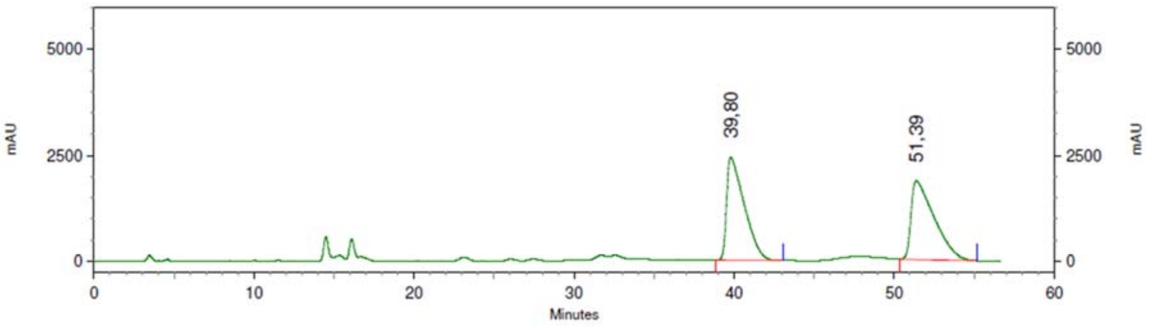

42: 240 nm, 4 nm
Results

Retention Time	Area	Area Percent
39,80	745284831	49,894
51,39	748447649	50,106

Enantioenriched compound 3ca

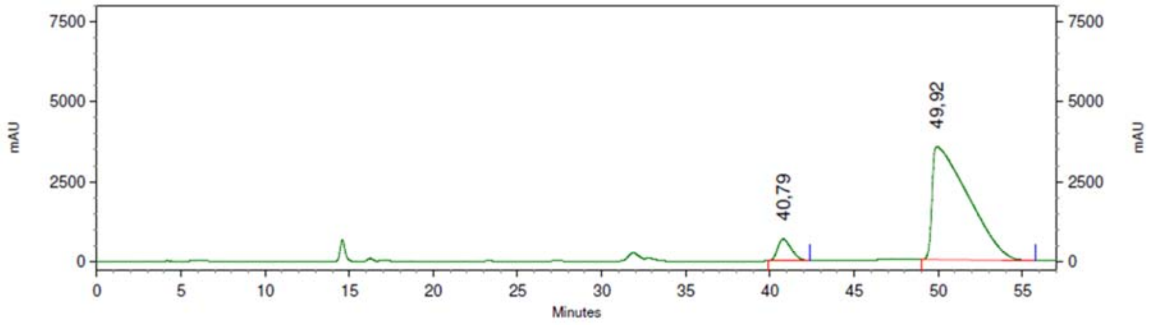

42: 240 nm, 4 nm
Results

Retention Time	Area	Area Percent
40,79	155749410	6,900
49,92	2101414784	93,100

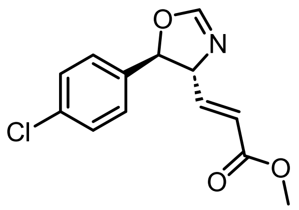

Racemic mixture 3da

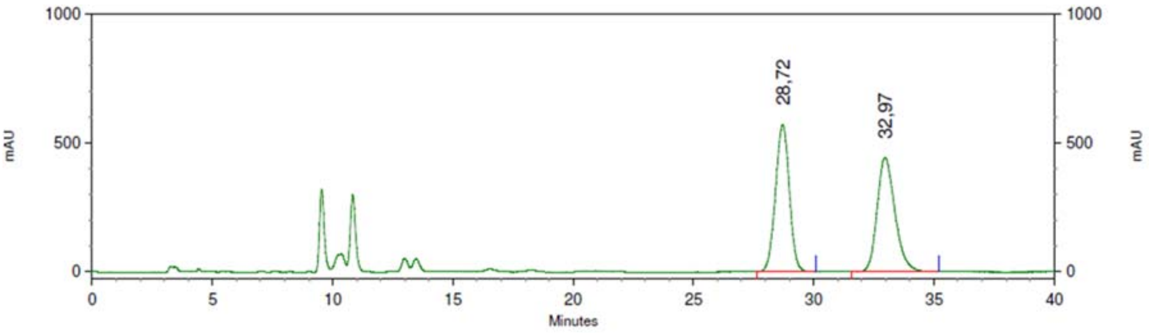

18: 224 nm, 4 nm
Results

Retention Time	Area	Area Percent
28,72	92358649	49,946
32,97	92556647	50,054

Enantioenriched compound 3da

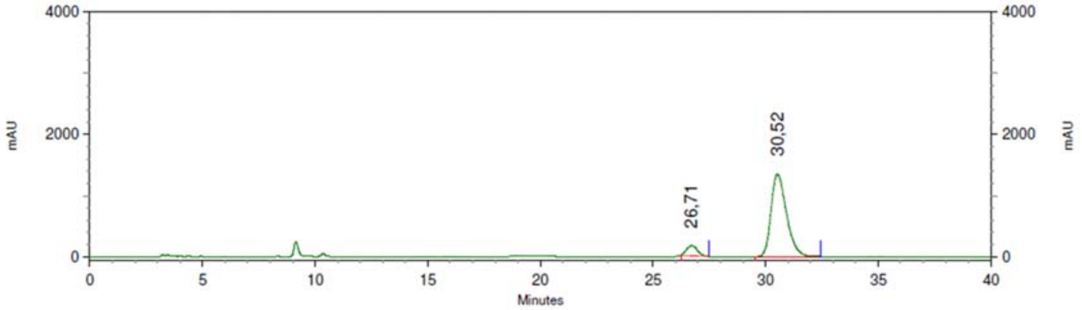

18: 224 nm, 4 nm
Results

Retention Time	Area	Area Percent
26,71	22084020	7,866
30,52	258671477	92,134

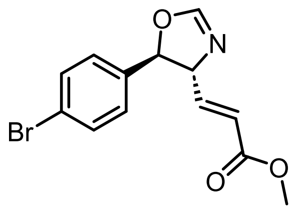

Racemic mixture 3ea

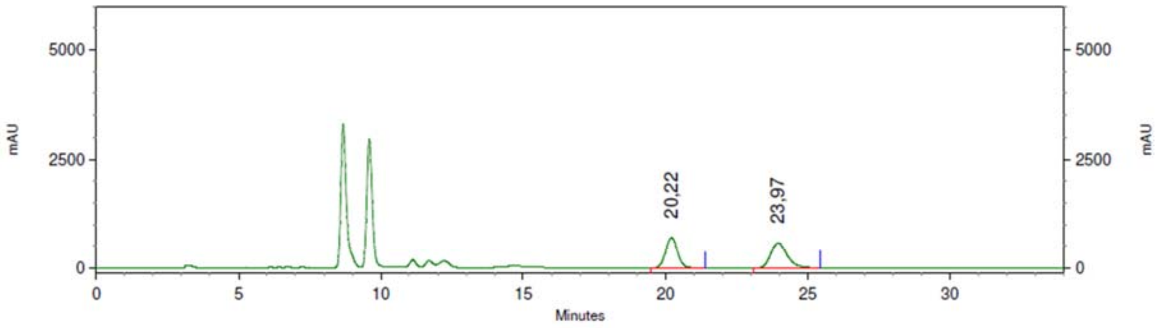

18: 224 nm, 4 nm
Results

Retention Time	Area	Area Percent
20,22	80455685	46,760
23,97	91604411	53,240

Enantioenriched compound 3ea

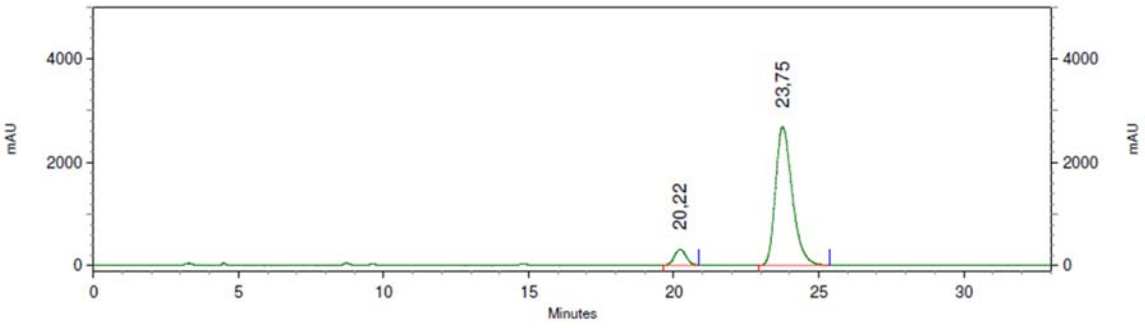

18: 224 nm, 4 nm
Results

Retention Time	Area	Area Percent
20,22	35604106	7,589
23,75	433570151	92,411

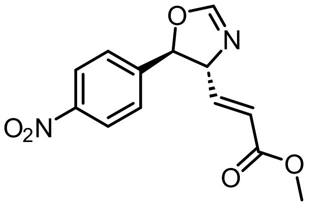

Racemic mixture 3fa

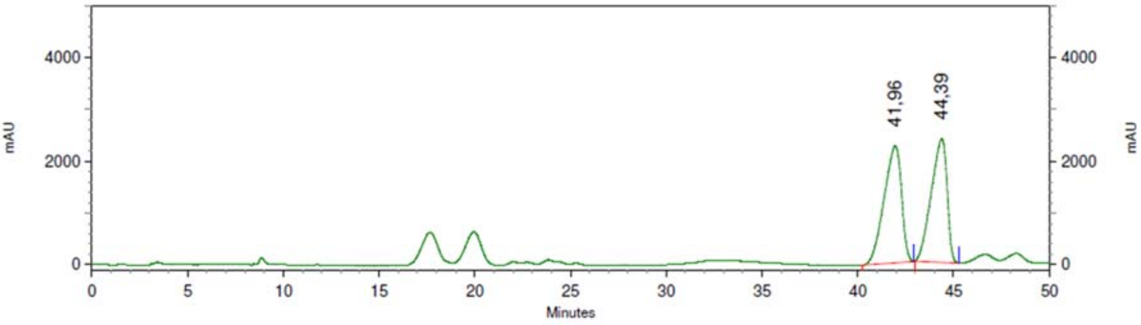

20: 237 nm, 4 nm
Results

Retention Time	Area	Area Percent
41,96	573864334	50,981
44,39	551788360	49,019

Enantioenriched compound 3fa

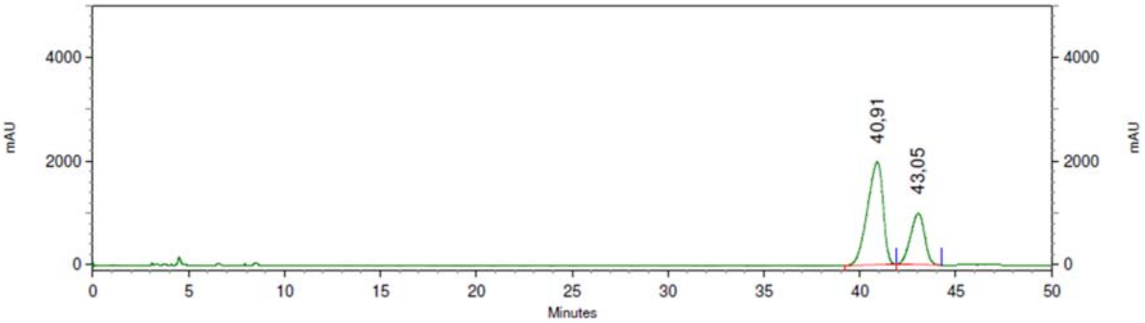

20: 237 nm, 4 nm
Results

Retention Time	Area	Area Percent
40,91	448553984	68,979
43,05	201725118	31,021

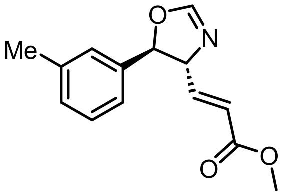

Racemic mixture 3ga

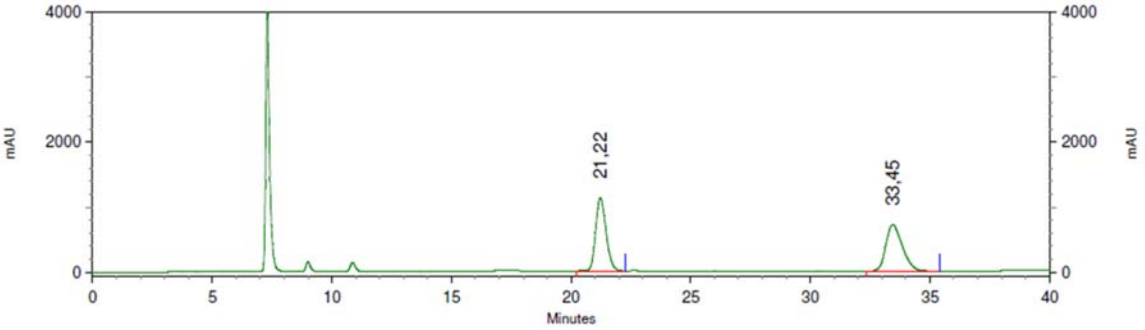

18: 224 nm, 4 nm
Results

Retention Time	Area	Area Percent
21,22	135172468	49,902
33,45	135705458	50,098

Enantioenriched compound 3ga

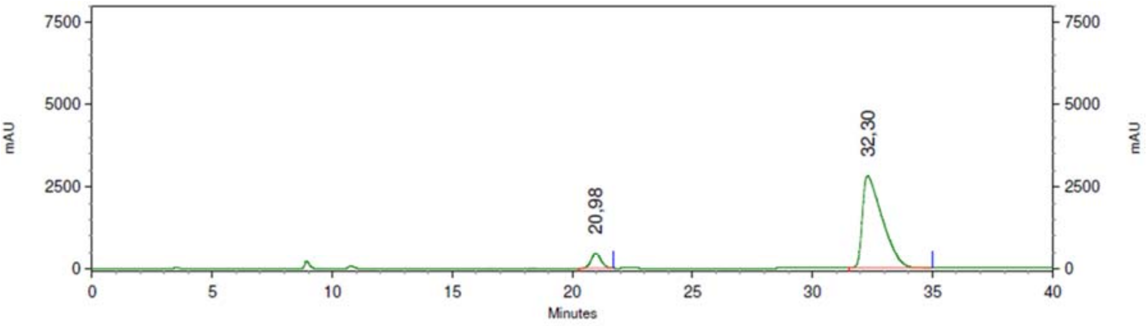

18: 224 nm, 4 nm
Results

Retention Time	Area	Area Percent
20,98	51964162	7,474
32,30	643272146	92,526

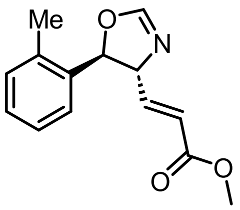

Racemic mixture 3ha

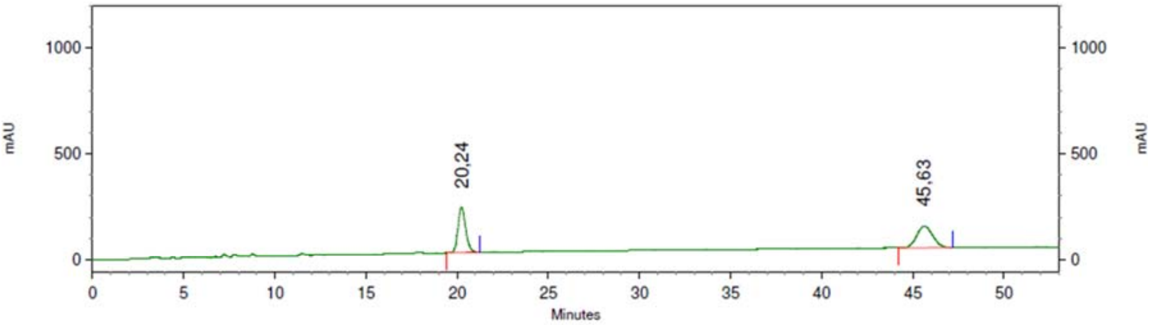

11: 231 nm, 4 nm
Results

Retention Time	Area	Area Percent
20,24	25080918	49,955
45,63	25125609	50,045

Enantioenriched compound 3ha

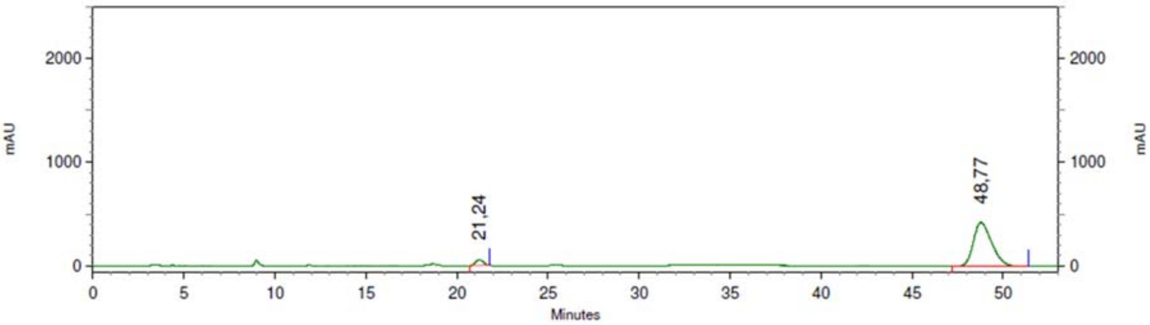

11: 231 nm, 4 nm
Results

Retention Time	Area	Area Percent
21,24	6328766	5,099
48,77	117781156	94,901

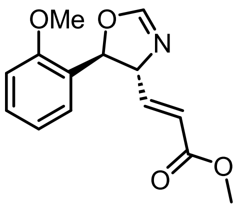

Racemic mixture 3ia

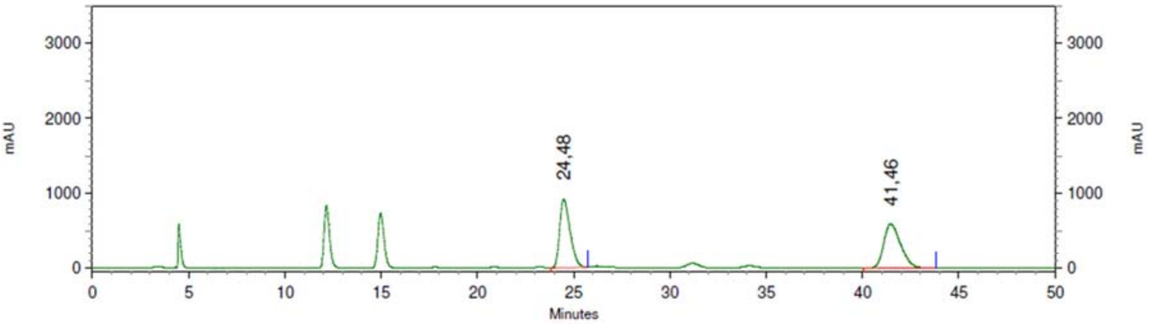

11: 231 nm, 4 nm
Results

Retention Time	Area	Area Percent
24,48	138564090	48,993
41,46	144262665	51,007

Enantioenriched compound 3ia

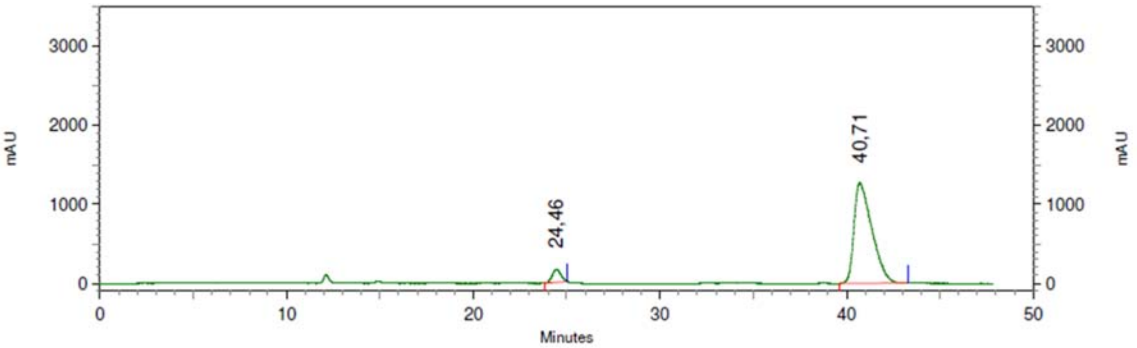

47: 231 nm, 4 nm
Results

Retention Time	Area	Area Percent
24,46	21066454	5,860
40,71	338455861	94,140

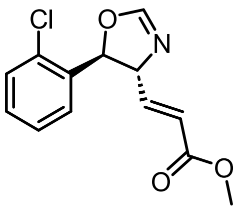

Racemic mixture 3ja

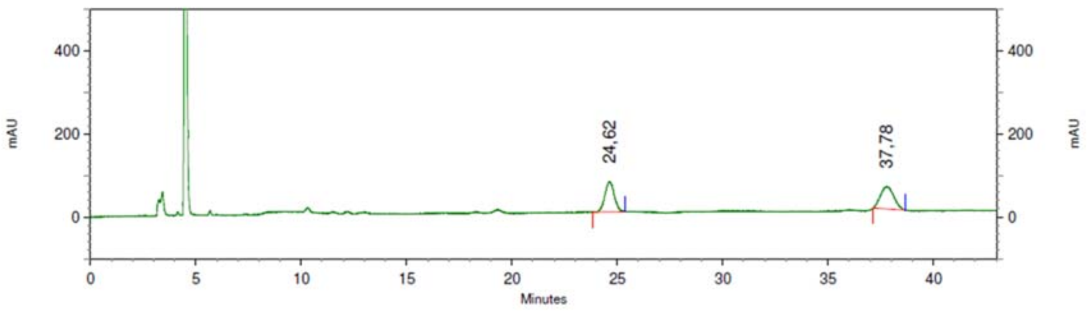

30: 202 nm, 4 nm
Results

Retention Time	Area	Area Percent
24,62	9066438	48,982
37,78	9443444	51,018

Enantioenriched compound 3ja

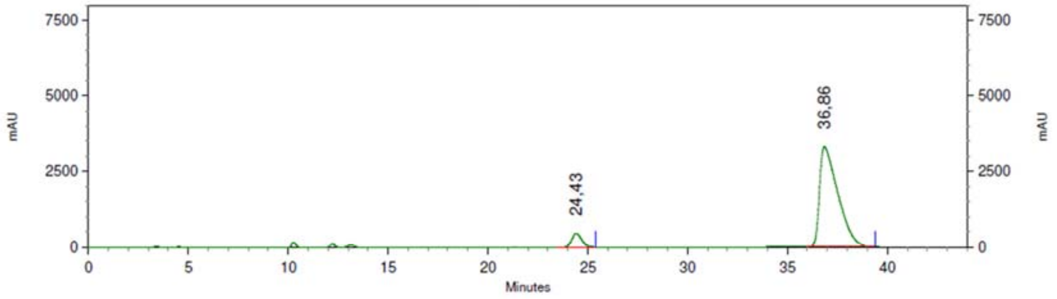

8: 219 nm, 4 nm Results

Retention Time	Area	Area Percent
24,43	58254922	6,529
36,86	833953340	93,471

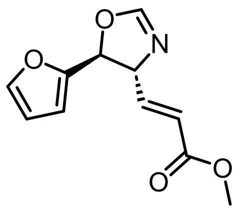

Racemic mixture 3ka

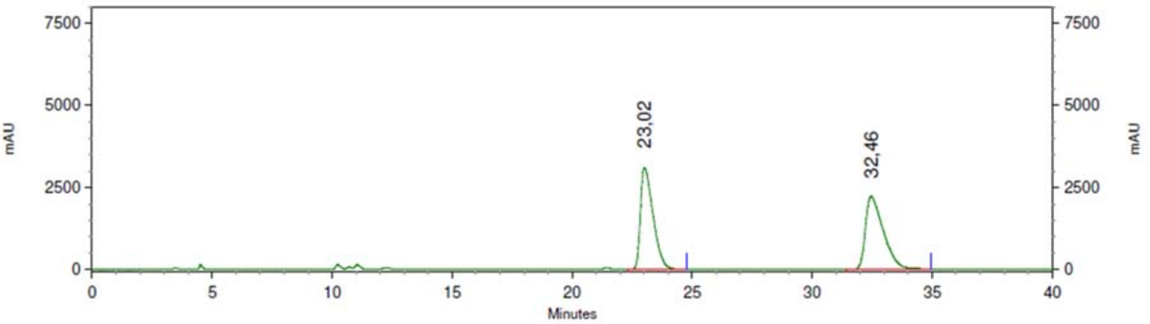

11: 231 nm, 4 nm
Results

Retention Time	Area	Area Percent
23,02	454095013	49,489
32,46	463479282	50,511

Enantioenriched compound 3ka

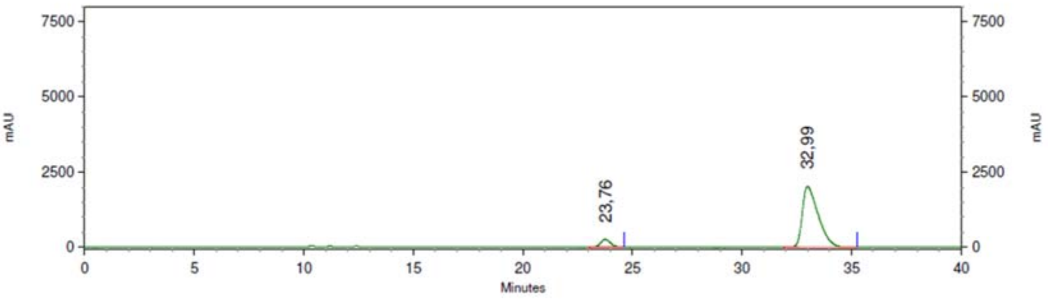

11: 231 nm, 4 nm
Results

Retention Time	Area	Area Percent
23,76	32491774	7,368
32,99	408494265	92,632

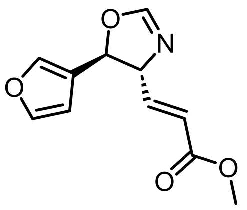

Racemic mixture 3la

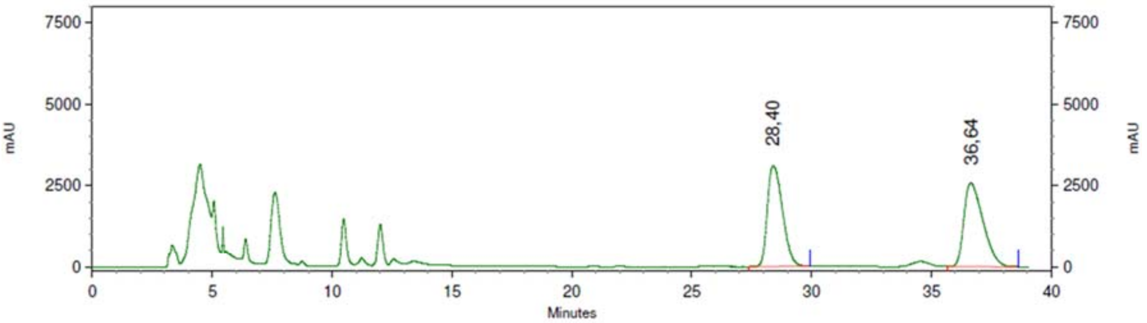

23: 206 nm, 4 nm
Results

Retention Time	Area	Area Percent
28,40	544585359	48,872
36,64	569734435	51,128

Enantioenriched compound 3la

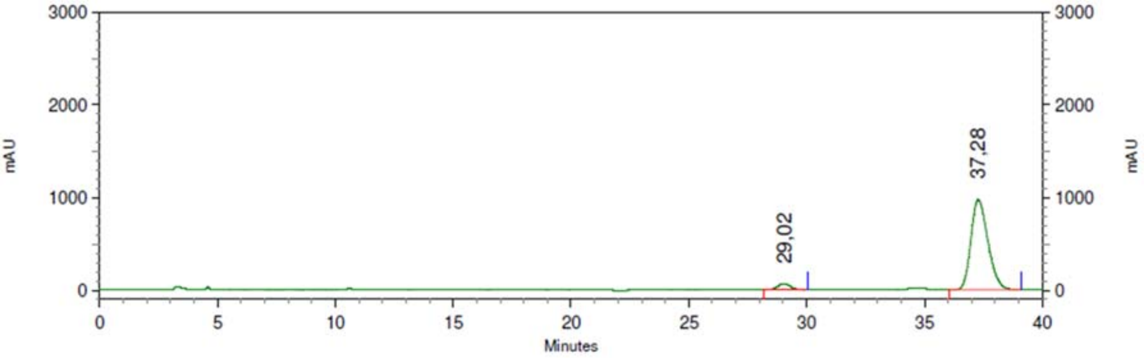

20: 206 nm, 4 nm
Results

Retention Time	Area	Area Percent
29,02	10716466	5,147
37,28	197508772	94,853

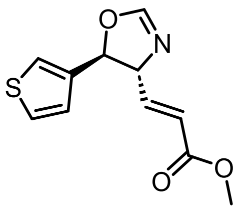

Racemic mixture 3ma

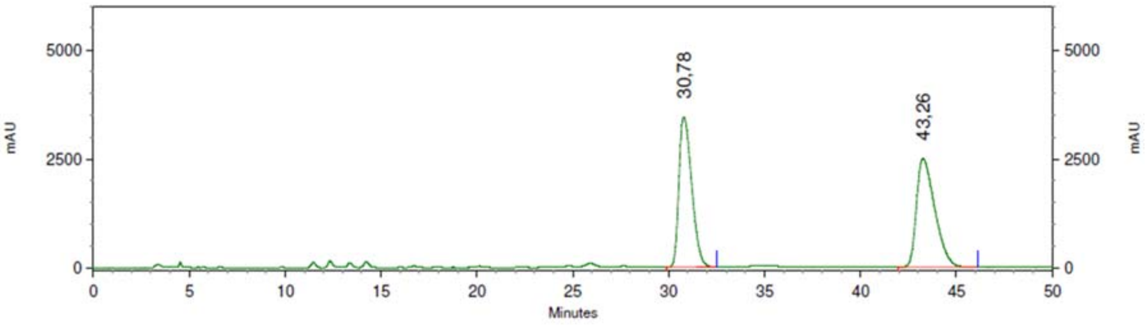

23: 206 nm, 4 nm
Results

Retention Time	Area	Area Percent
30,78	638709678	48,981
43,26	665292838	51,019

Enantioenriched compound 3ma

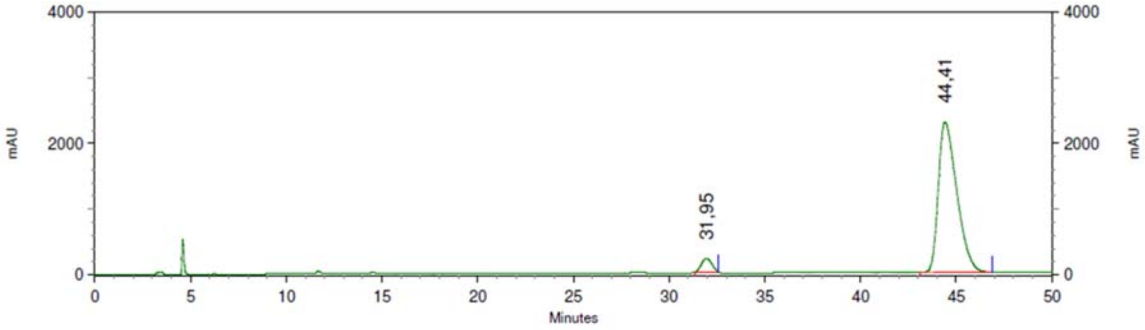

23: 206 nm, 4 nm
Results

Retention Time	Area	Area Percent
31,95	31505491	4,862
44,41	616526793	95,138

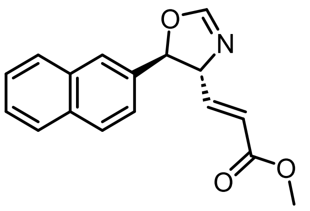

Racemic mixture 3na

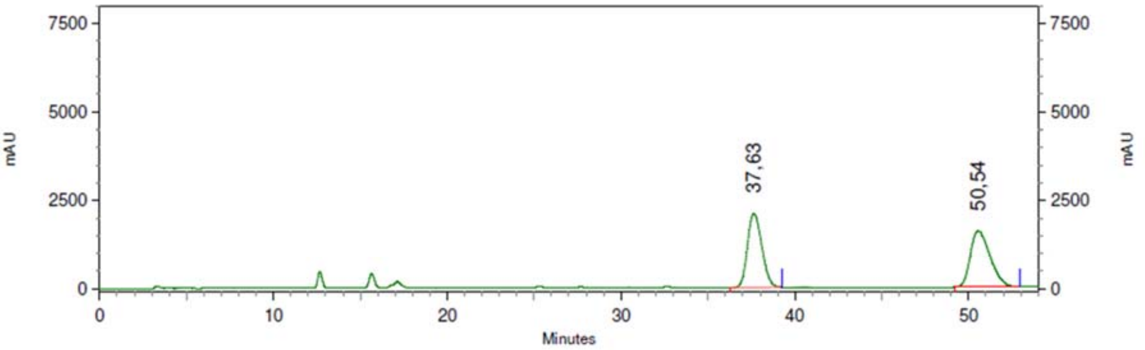

13: 202 nm, 4 nm
Results

Retention Time	Area	Area Percent
37,63	478158462	48,764
50,54	502392906	51,236

Enantioenriched compound 3na

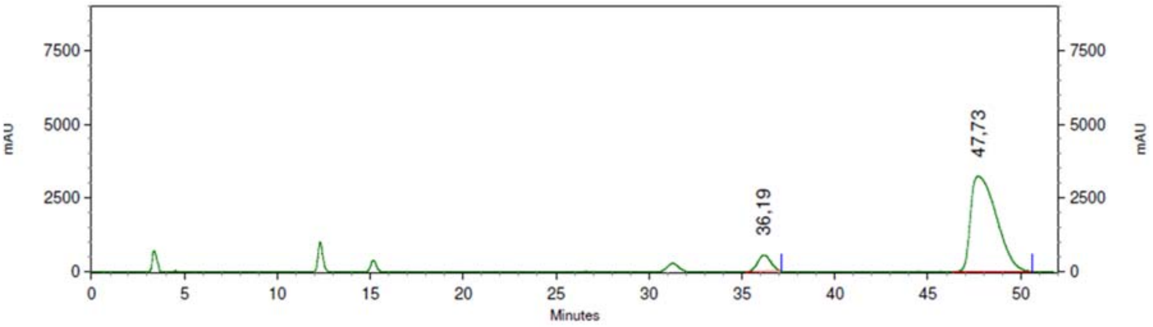

27: 202 nm, 4 nm
Results

Retention Time	Area	Area Percent
36,19	105119087	7,828
47,73	1237767282	92,172

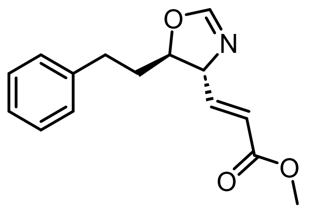

Racemic mixture 3oa

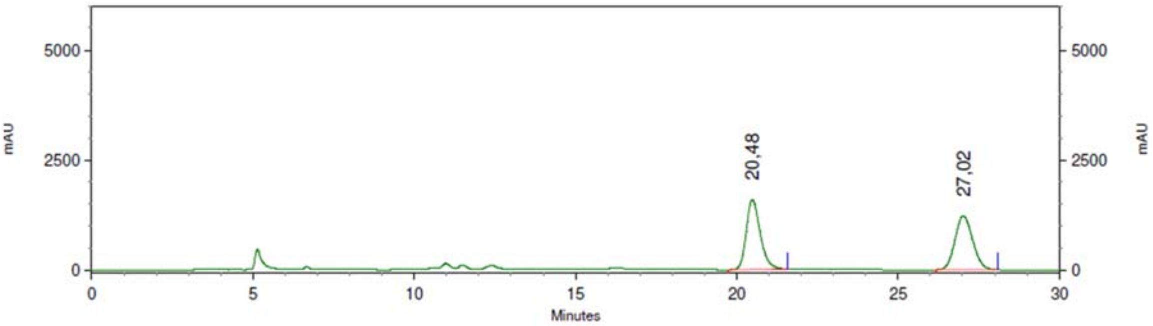

29: 243 nm, 4 nm
Results

Retention Time	Area	Area Percent
20,48	191280888	50,606
27,02	186696699	49,394

Enantioenriched compound 3oa

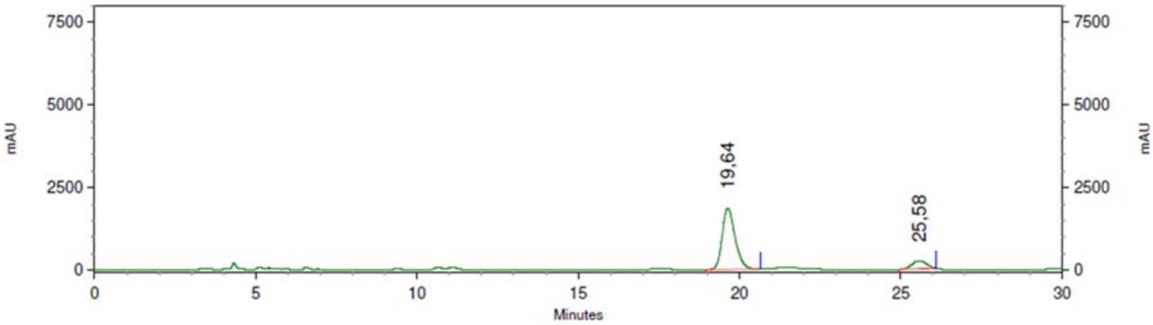

29: 243 nm, 4 nm
Results

Retention Time	Area	Area Percent
19,64	207285689	87,136
25,58	30601466	12,864

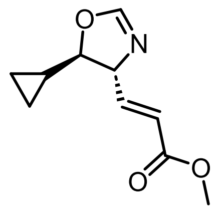

Racemic mixture 3pa

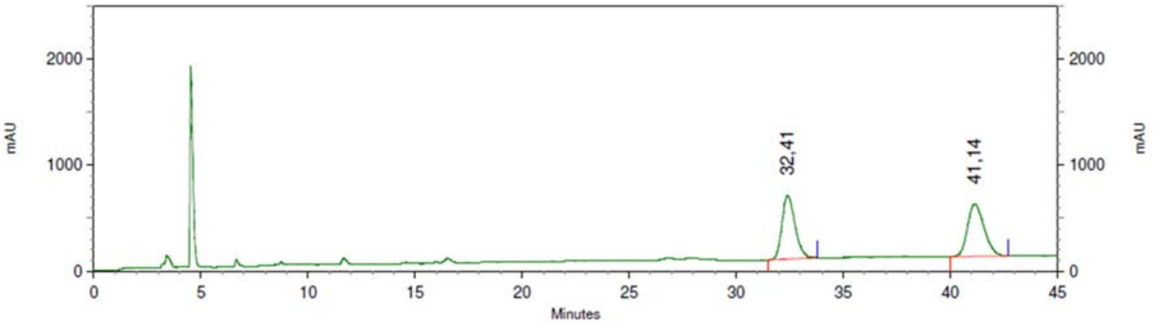

27: 202 nm, 4 nm
Results

Retention Time	Area	Area Percent
32,41	101871802	48,662
41,14	107473418	51,338

Enantioenriched compound 3pa

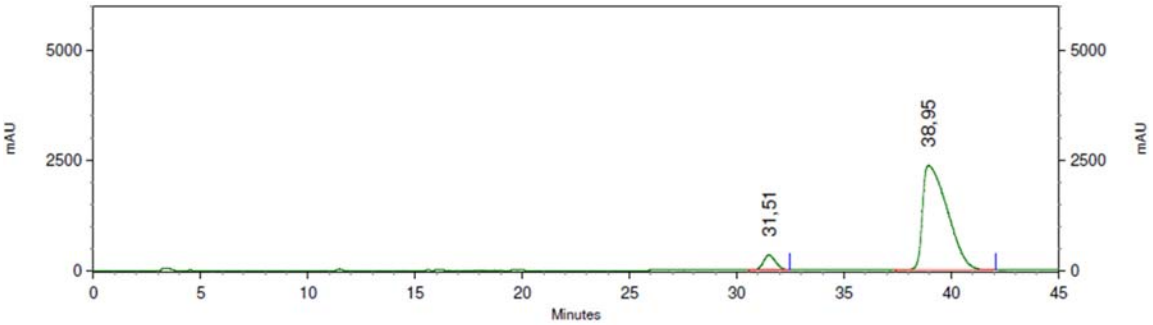

27: 202 nm, 4 nm
Results

Retention Time	Area	Area Percent
31,51	53440675	6,567
38,95	760394673	93,433

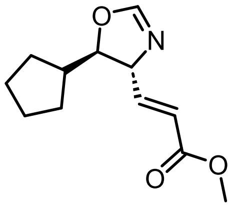

Racemic mixture 3qa

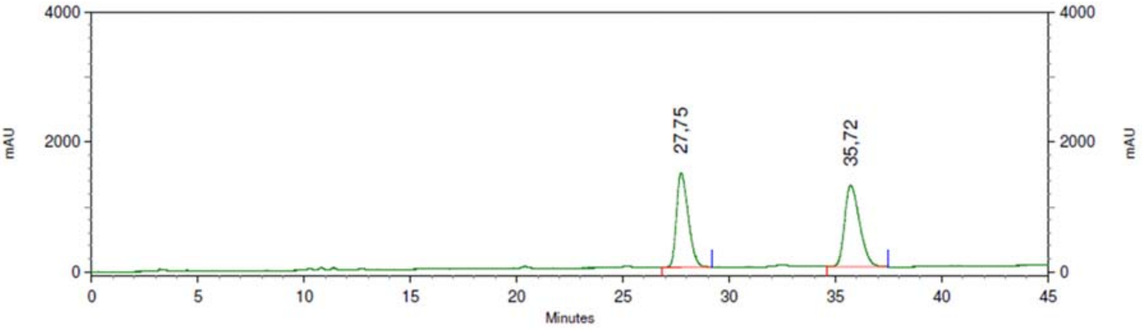

47: 203 nm, 4 nm
Results

Retention Time	Area	Area Percent
27,75	221468554	47,495
35,72	244833169	52,505

Enantioenriched compound 3qa

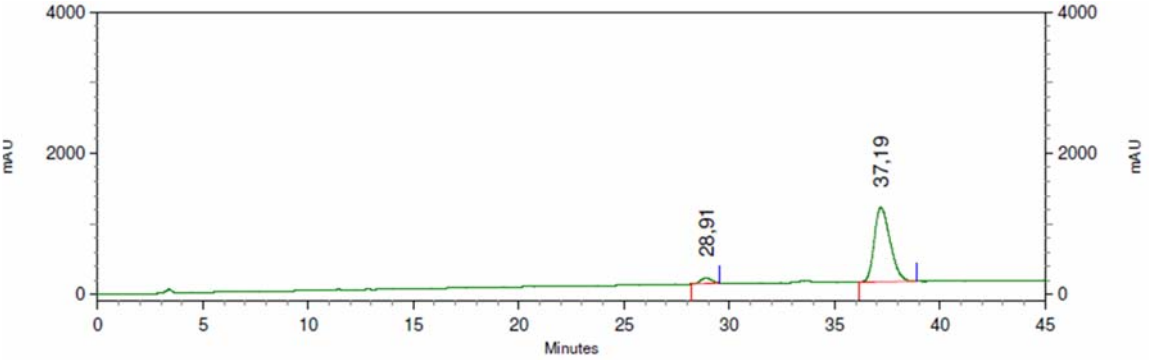

39: 203 nm, 4 nm
Results

Retention Time	Area	Area Percent
28,91	11849961	5,099
37,19	220534111	94,901

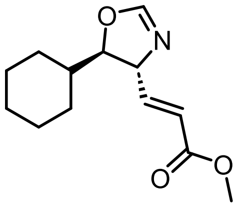

Racemic mixture 3ra

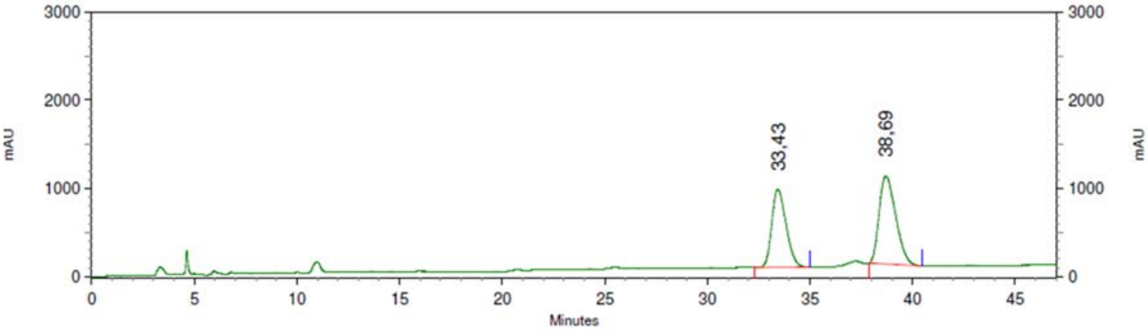

27: 202 nm, 4 nm
Results

Retention Time	Area	Area Percent
33,43	171744756	43,370
38,69	224258414	56,630

Enantioenriched compound 3ra

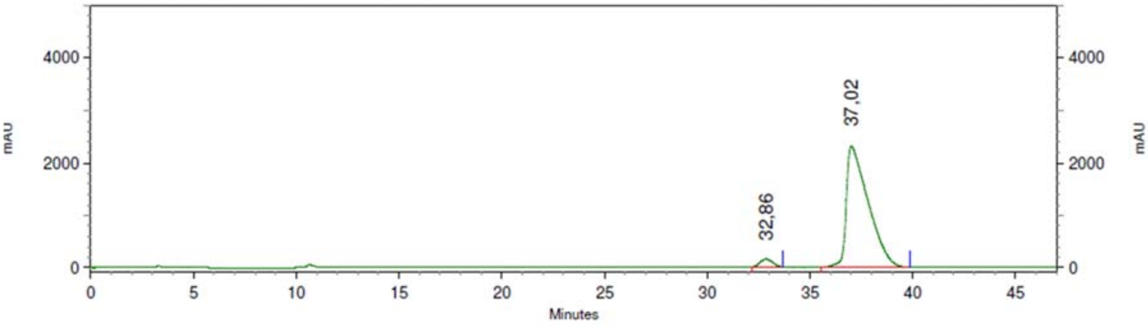

27: 222 nm, 4 nm
Results

Retention Time	Area	Area Percent
32,86	24977489	3,533
37,02	681947805	96,467

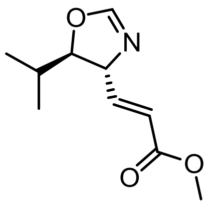

Racemic mixture 3sa

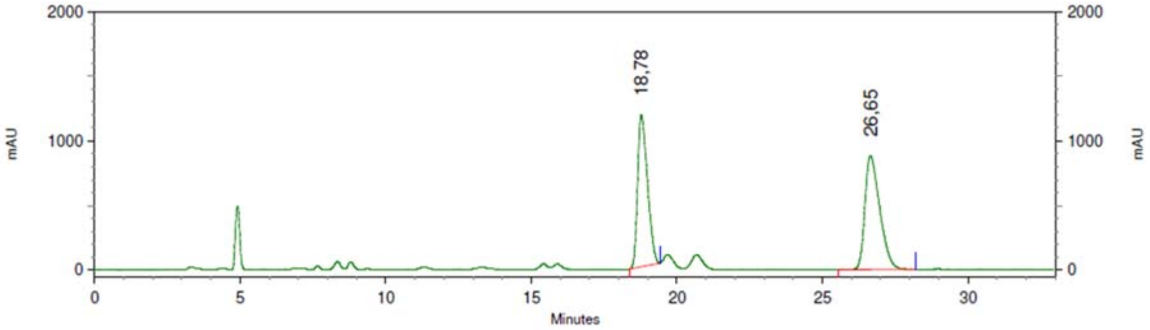

18: 224 nm, 4 nm
Results

Retention Time	Area	Area Percent
18,78	110350275	46,801
26,65	125438265	53,199

Enantioenriched compound 3sa

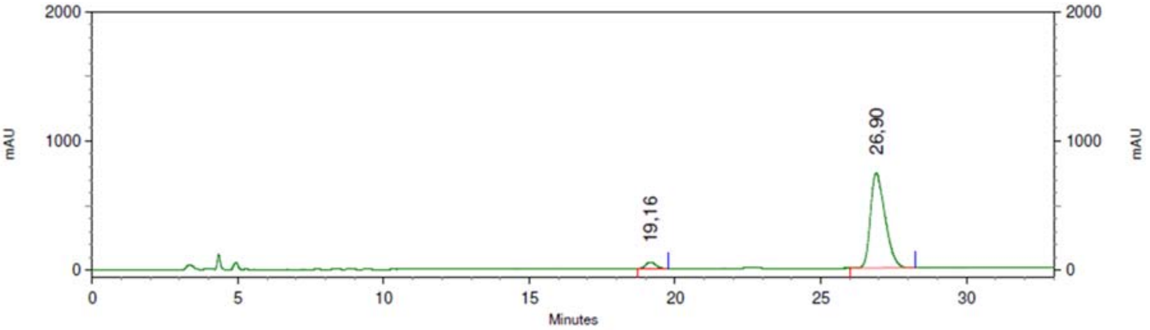

18: 224 nm, 4 nm
Results

Retention Time	Area	Area Percent
19,16	4750657	4,436
26,90	102333270	95,564

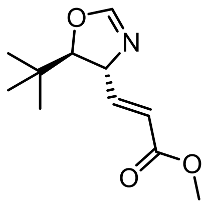

Racemic mixture 3ta

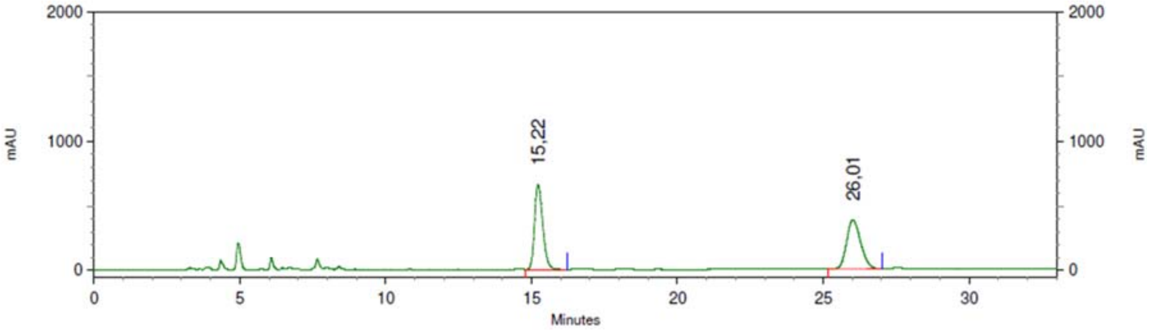

18: 224 nm, 4 nm
Results

Retention Time	Area	Area Percent
15,22	51556608	50,098
26,01	51354318	49,902

Enantioenriched compound 3ta

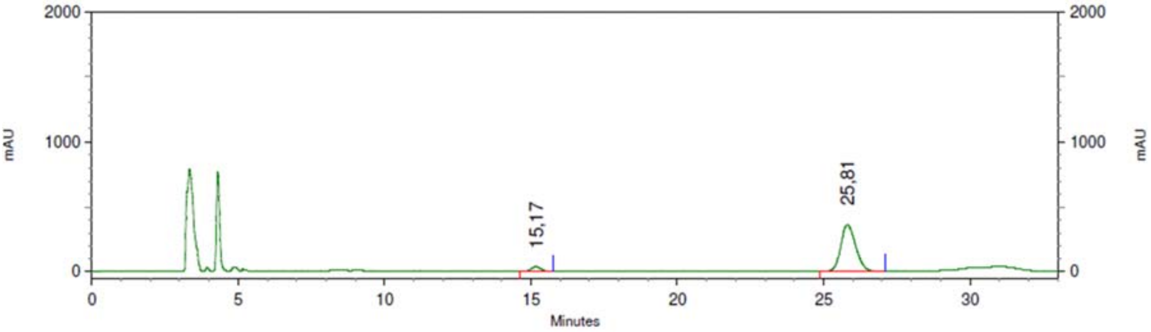

18: 224 nm, 4 nm
Results

Retention Time	Area	Area Percent
15,17	2961521	5,524
25,81	50648408	94,476

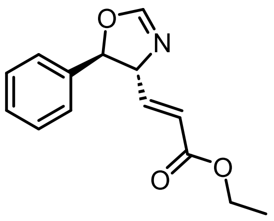

Racemic mixture 3ab

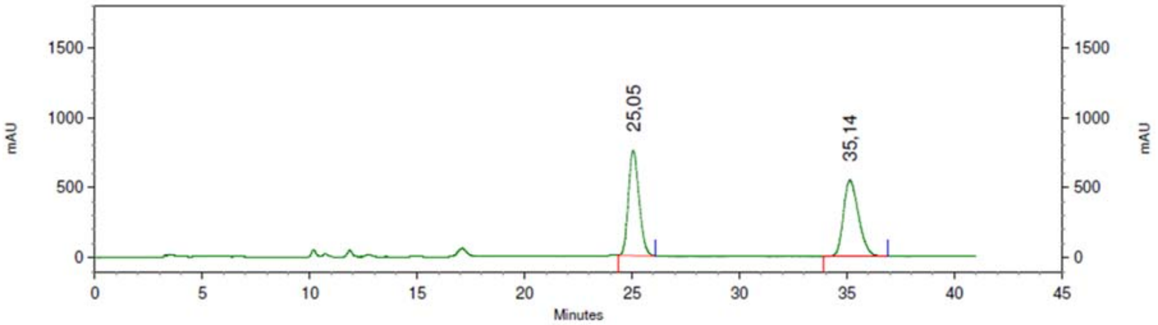

18: 224 nm, 4 nm
Results

Retention Time	Area	Area Percent
25,05	103486497	49,306
35,14	106400279	50,694

Enantioenriched compound 3ab

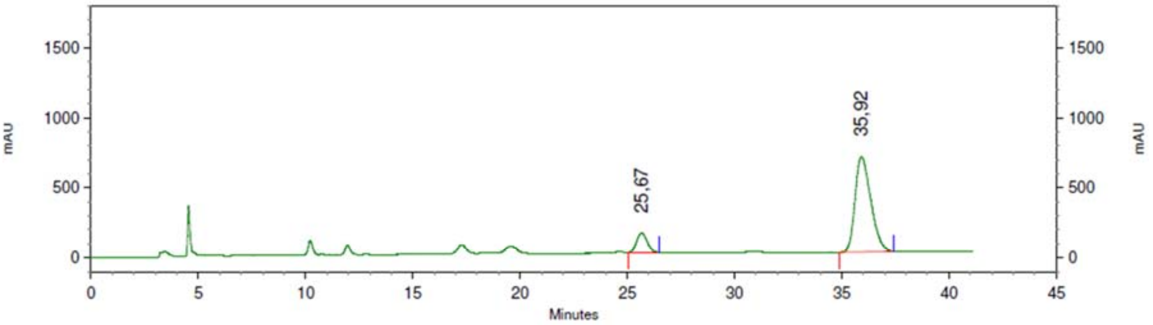

18: 224 nm, 4 nm
Results

Retention Time	Area	Area Percent
25,67	19272749	12,196
35.92	138750067	87.804

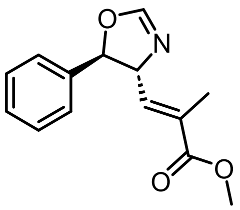

Racemic mixture 3ac

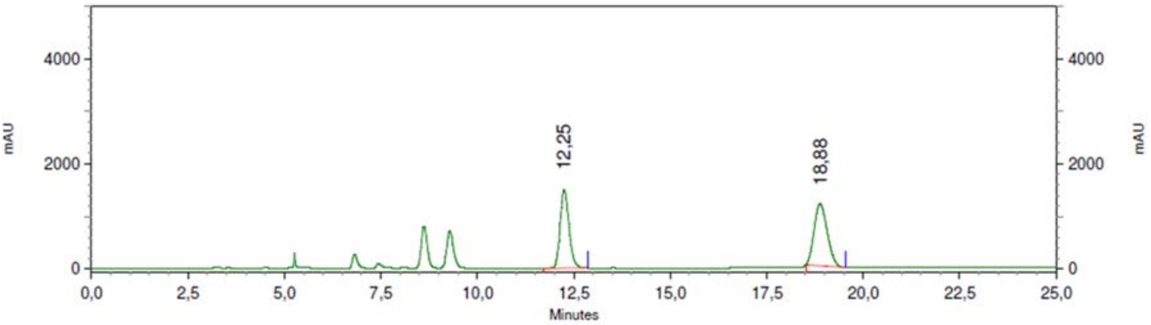

32: 215 nm, 4 nm
Results

Retention Time	Area	Area Percent
12,25	96122628	46,361
18,88	111210872	53,639

Enantioenriched compound 3ac

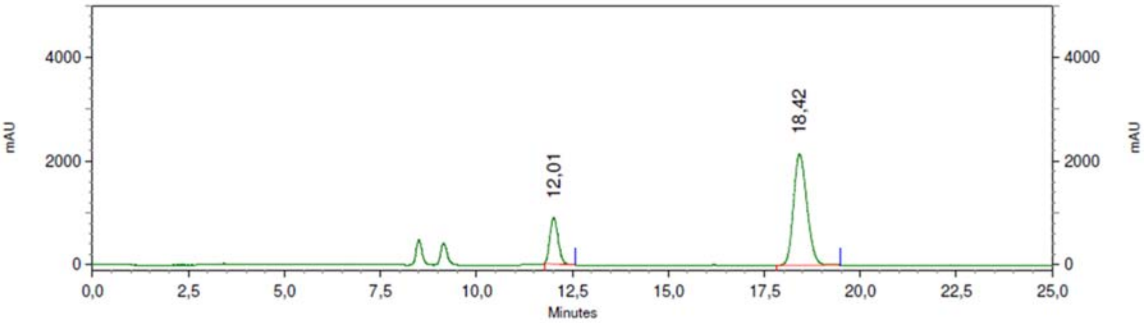

32: 215 nm, 4 nm
Results

Retention Time	Area	Area Percent
12,01	53324980	20,507
18,42	206705213	79,493

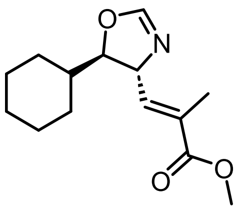

Racemic mixture 3rc

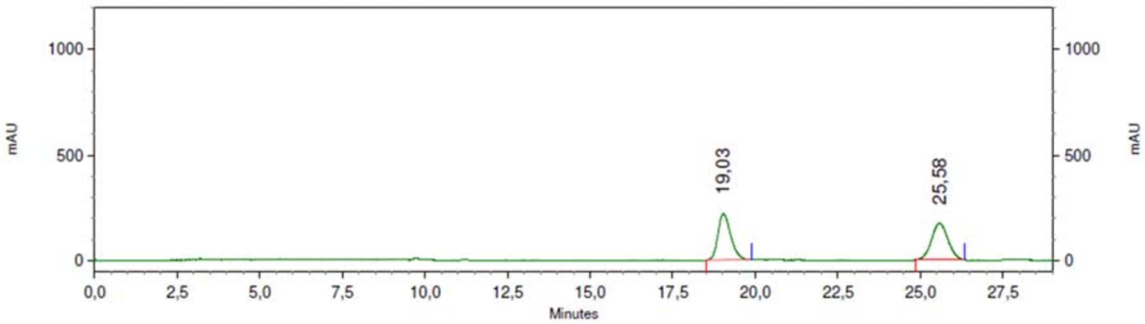

13: 230 nm, 4 nm
Results

Retention Time	Area	Area Percent
19,03	23594443	49,239
25,58	24323594	50,761

Enantioenriched compound 3rc

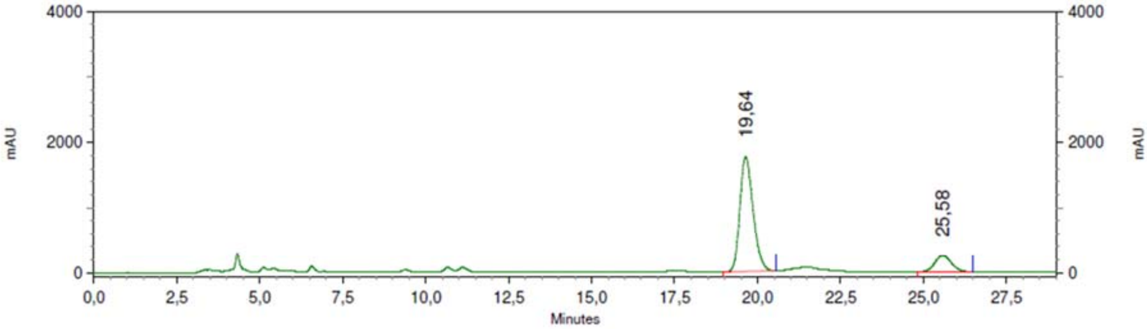

42: 240 nm, 4 nm
Results

Retention Time	Area	Area Percent
19,64	196029987	84,978
25,58	34653383	15,022

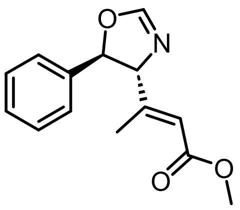

Racemic mixture 3ad

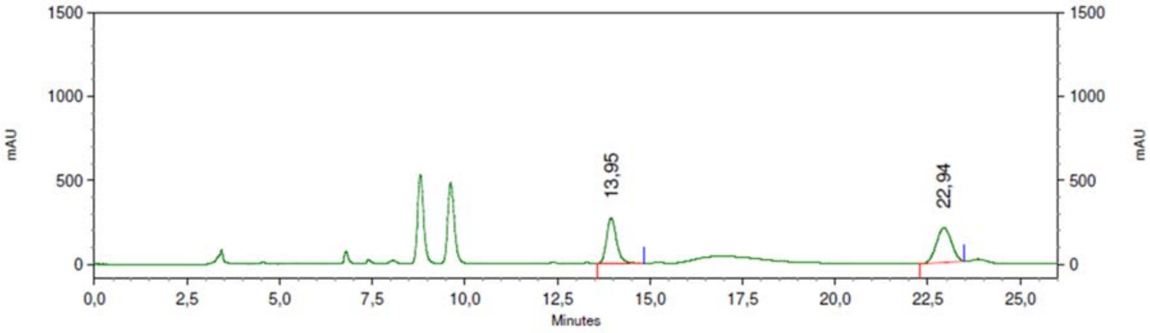

18: 224 nm, 4 nm
Results

Retention Time	Area	Area Percent
13,95	20144806	46,353
22,94	23314781	53,647

Enantioenriched compound 3ad

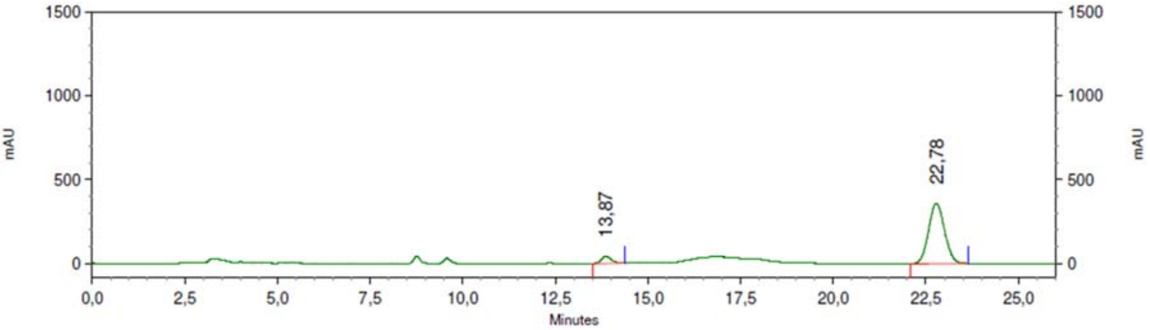

18: 224 nm, 4 nm
Results

Retention Time	Area	Area Percent
13,87	3140027	6,778
22,78	43188967	93,222

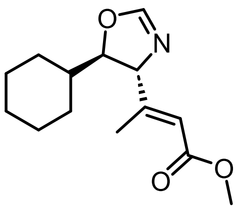

Racemic mixture 3rd

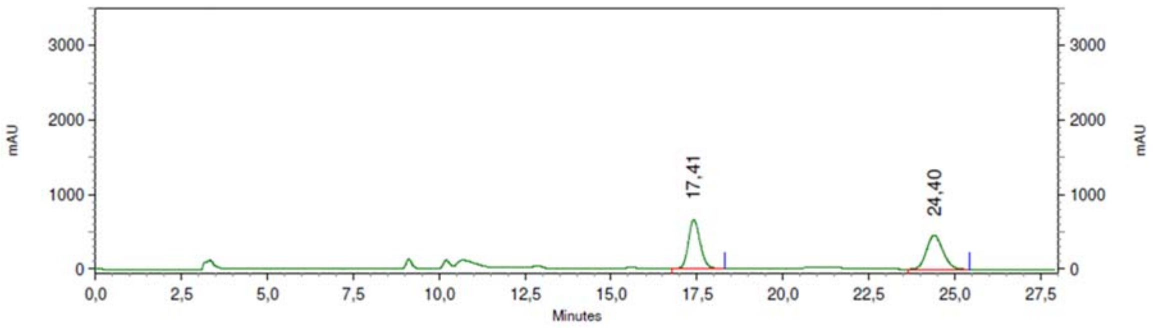

18: 224 nm, 4 nm
Results

Retention Time	Area	Area Percent
17,41	61905400	50,311
24,40	61140474	49,689

Enantioenriched compound 3rd

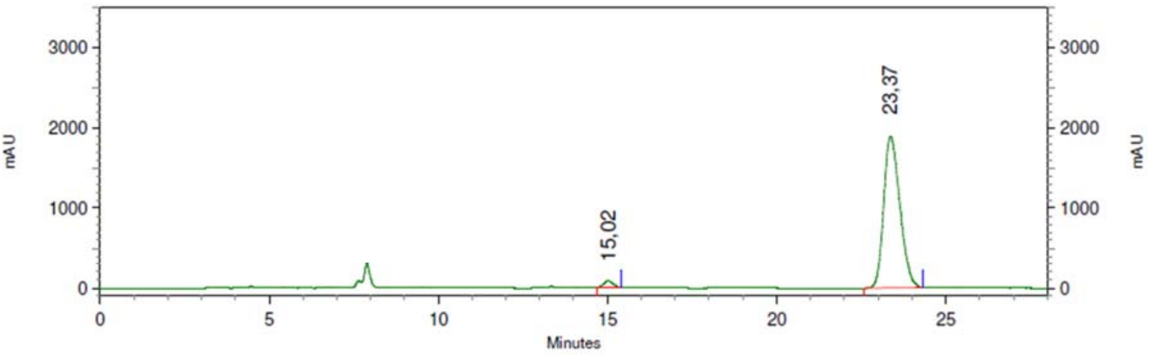

18: 224 nm, 4 nm
Results

Retention Time	Area	Area Percent
15,02	6280903	2,437
23,37	251442096	97,563

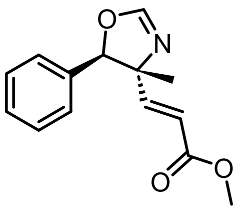

Racemic mixture (major diastereomer) 3ae

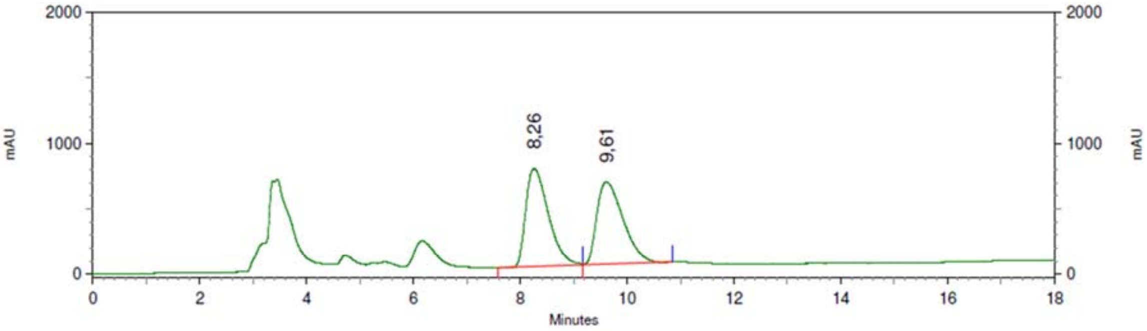

27: 202 nm, 4 nm
Results

Retention Time	Area	Area Percent
8,26	87950058	50,651
9,61	85690042	49,349

Enantioenriched compound (major diastereomer) 3ae

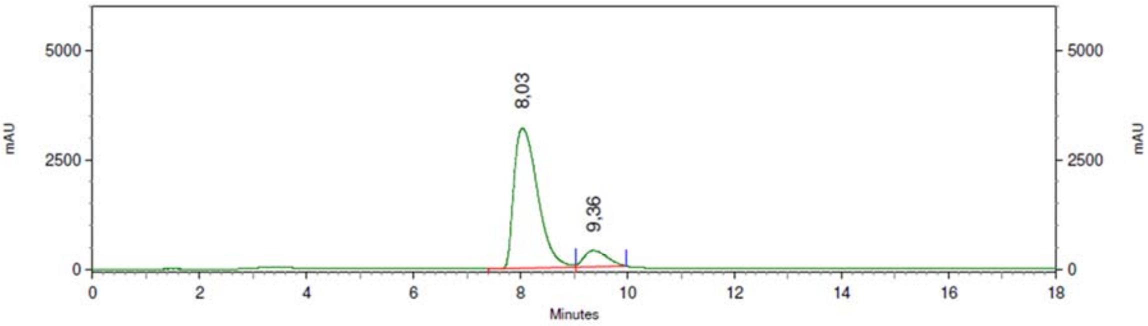

23: 206 nm, 4 nm
Results

Retention Time	Area	Area Percent
8,03	392269218	89,858
9,36	44273898	10,142

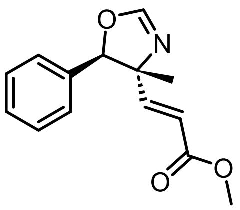

Racemic mixture (minor diastereomer) 3ae

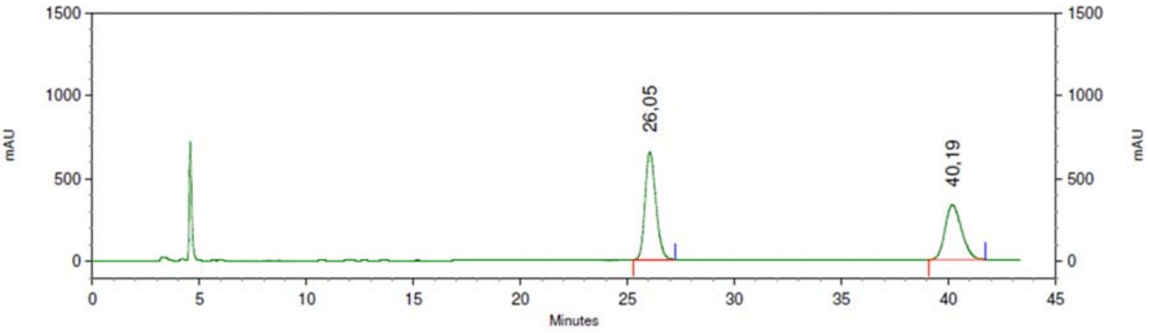

18: 224 nm, 4 nm
Results

Retention Time	Area	Area Percent
26,05	91657077	56,323
40,19	71078735	43,677

Enantioenriched compound (minor diastereomer) 3ae

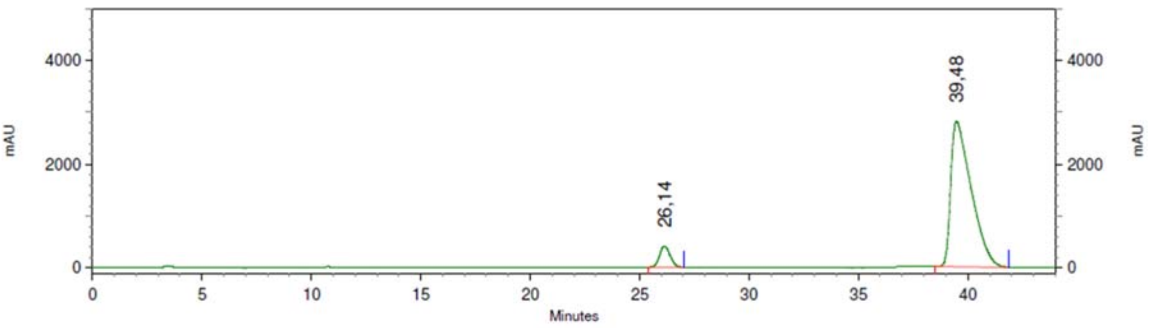

27: 218 nm, 4 nm
Results

Retention Time	Area	Area Percent
26,14	54938969	7,016
39,48	728062224	92,984

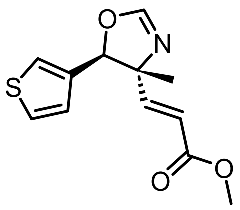

Racemic mixture (diastereomeric mixture) 3me

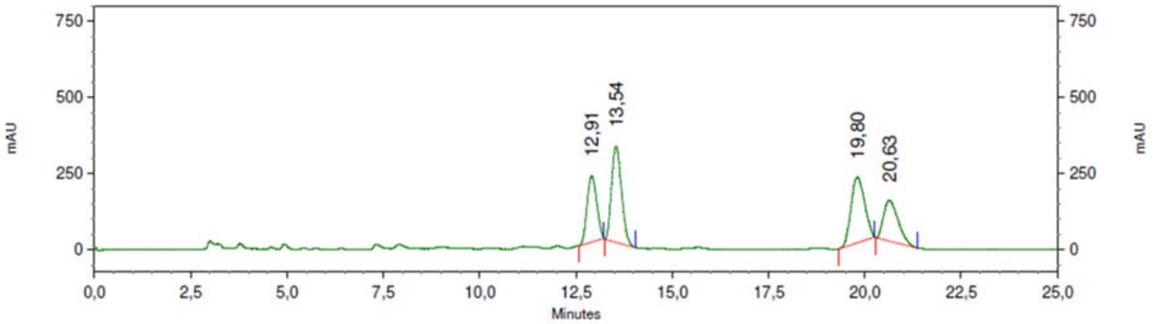


```
11: 225 nm, 4 nm
Results
```

Retention Time	Area	Area Percent
12,91	15166230	20,699
13,54	22823344	31,150
19,80	21124811	28,832
20,63	14155261	19,319

Enantioenriched compound (major diastereomer) 3me

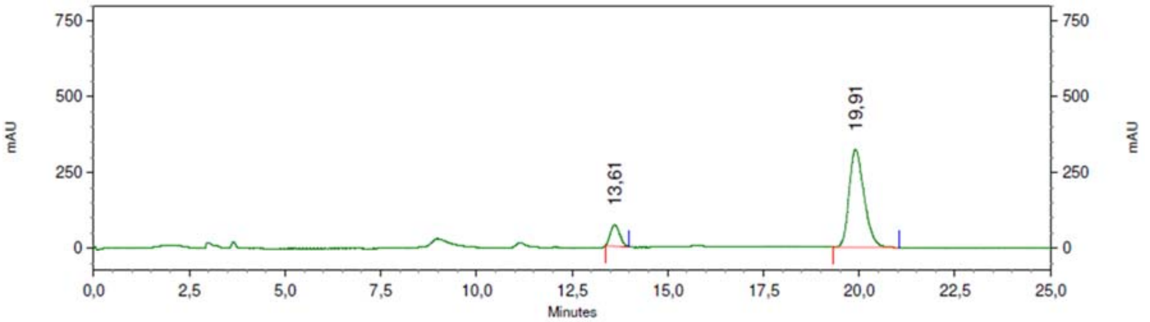


```
10: 223 nm, 4 nm
Results
```

Retention Time	Area	Area Percent
13,61	4872918	12,108
19,91	35372016	87,892

Enantioenriched compound (minor diastereomer) 3me

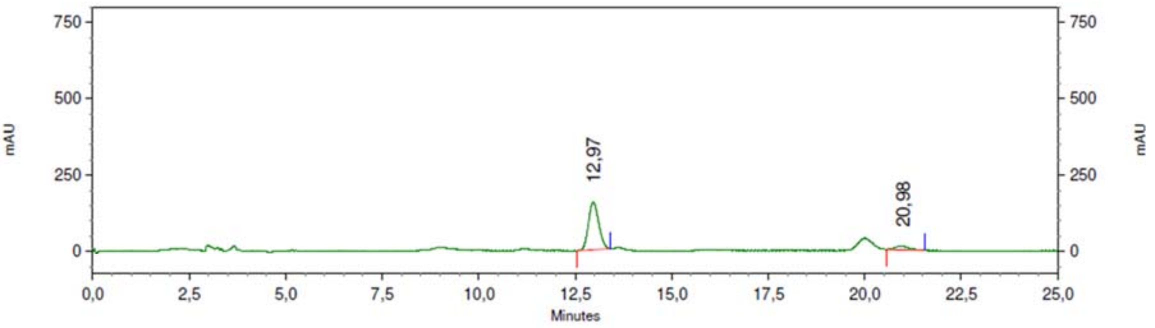

11: 225 nm, 4 nm
Results

Retention Time	Area	Area Percent
12,97	11621547	89,745
20,98	1327938	10,255

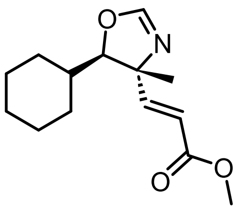

Racemic mixture (diastereomeric mixture) 3re

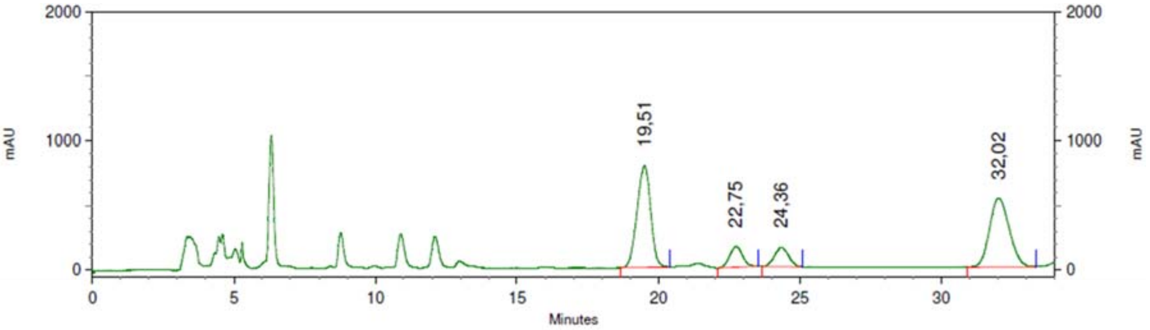

27: 202 nm, 4 nm
Results

Retention Time	Area	Area Percent
19,51	105786743	42,262
22,75	20396586	8,148
24,36	20710476	8,274
32,02	103418575	41,316

Enantioenriched product (diastereomeric mixture) 3re

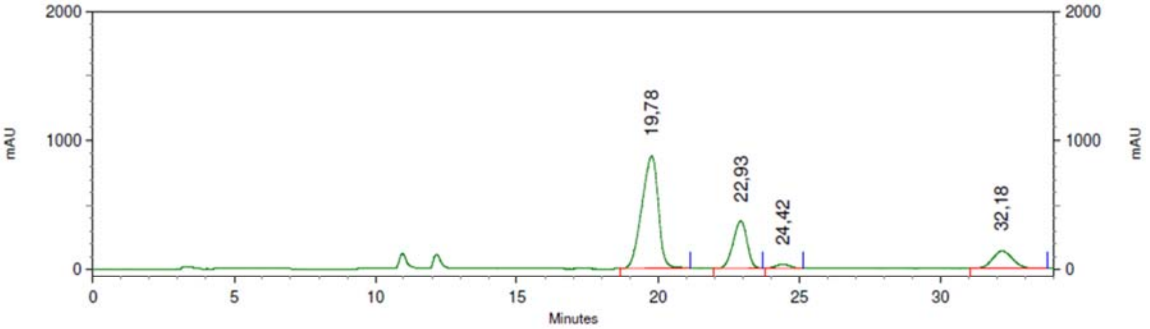

27: 226 nm, 4 nm
Results

Retention Time	Area	Area Percent
19,78	140724493	63,538
22,93	50331874	22,725
24,42	4283945	1,934
32,18	26139547	11,802

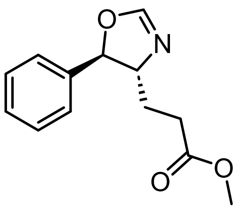

Racemic mixture 4

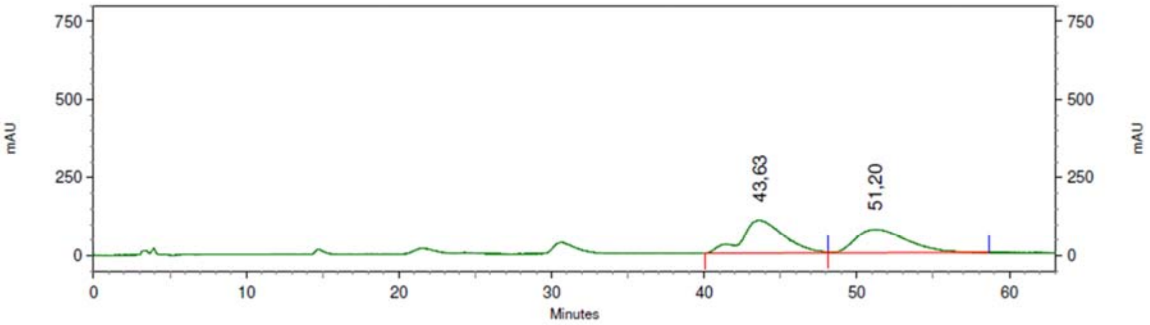

6: 217 nm, 4 nm Results

Retention Time	Area	Area Percent
43,63	82252845	53,674
51,20	70992004	46,326

Enantioenriched product 4

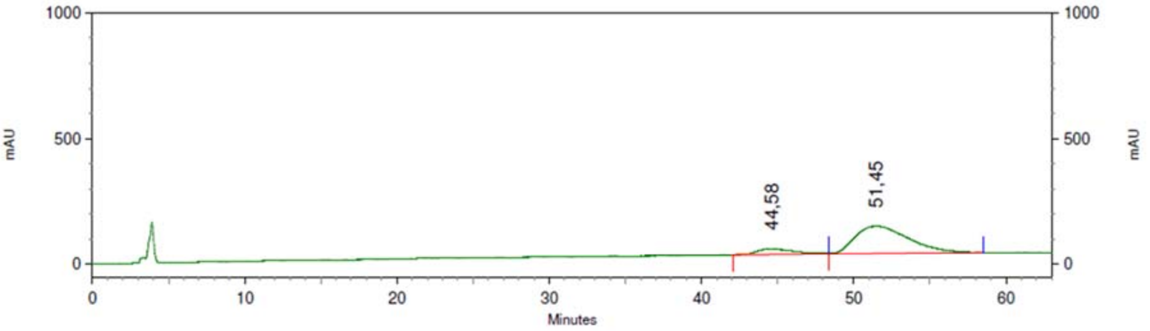

12: 211 nm, 4 nm
Results

Retention Time	Area	Area Percent
44,58	15084215	12,384
51,45	106722792	87,616

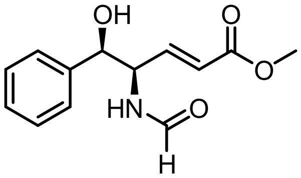

Racemic mixture 5aa

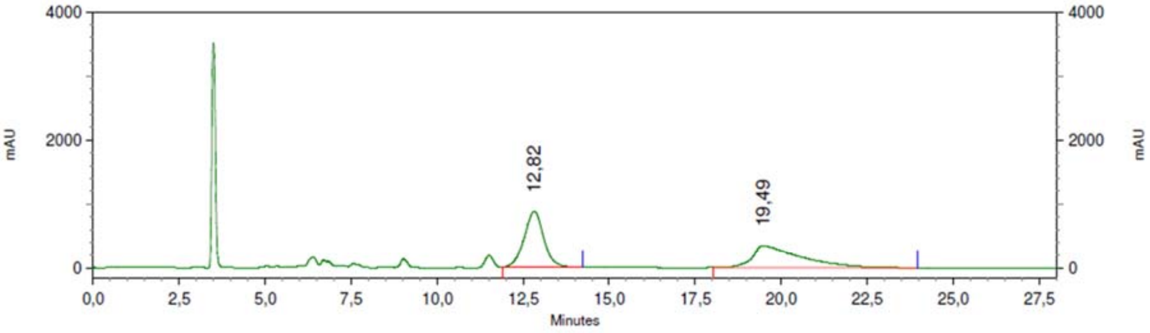

48: 211 nm, 4 nm
Results

Retention Time	Area	Area Percent
12,82	139076431	50,388
19,49	136934647	49,612

Enantioenriched compound 5aa

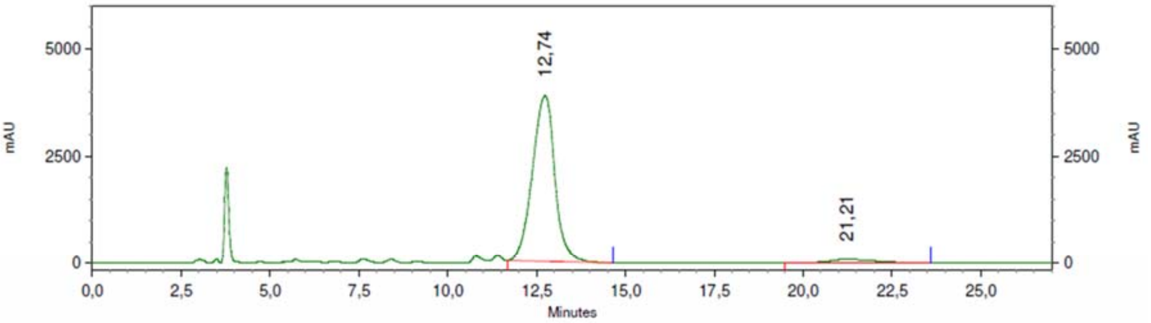

48: 211 nm, 4 nm
Results

Retention Time	Area	Area Percent
12,74	680474046	95,566
21,21	31574091	4,434

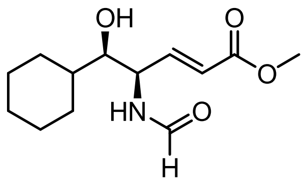

Racemic mixture 5ra

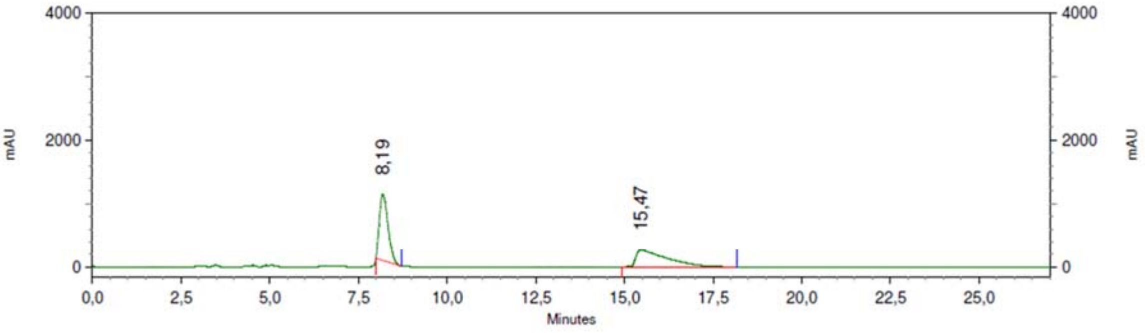

50: 223 nm, 4 nm
Results

Retention Time	Area	Area Percent
8,19	71156172	52,260
15,47	65002137	47,740

Enantioenriched compound 5ra

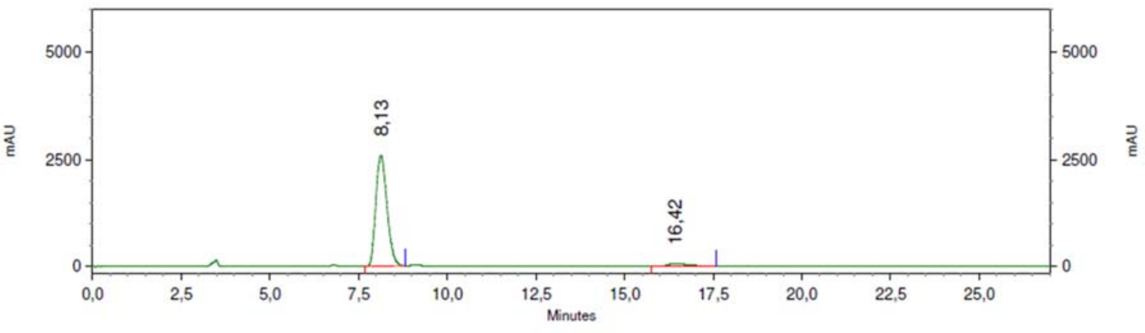

50: 223 nm, 4 nm
Results

Retention Time	Area	Area Percent
8,13	225993982	95,311
16,42	11118328	4,689

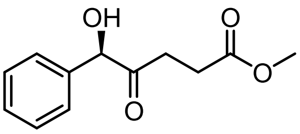

Enantioenriched compound 6.[4]

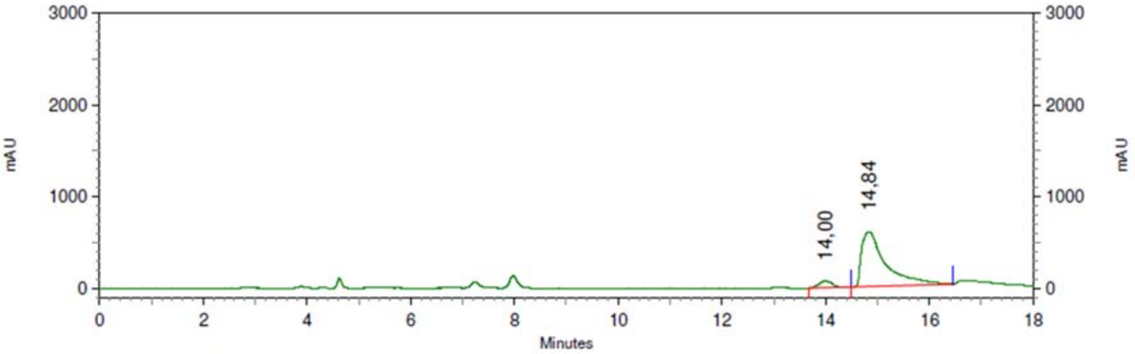

40: 236 nm, 4 nm
Results

Retention Time	Area	Area Percent
14,00	4586194	5,579
14,84	77619170	94,421

Optimization of the reaction conditions. Additional experiments

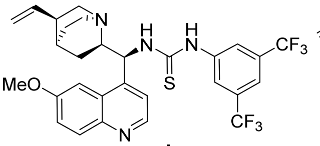

I

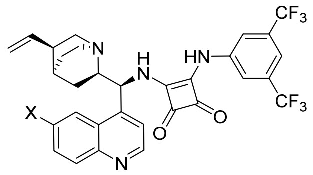

II, X = MeO
III, X= H

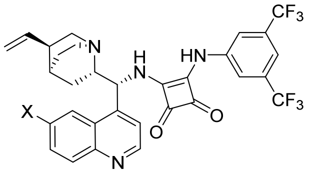

IV, X = MeO
V, X = H

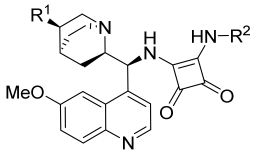

VI, R^1 = CH_2=CH, R^2 = ^{t}Bu
VII, R^1 = Et, R^2 = 2,6-^{i}Pr_2-C_6H_3

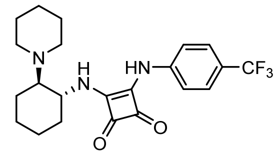

VIII

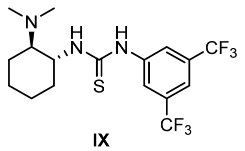

IX

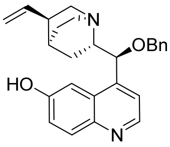

X

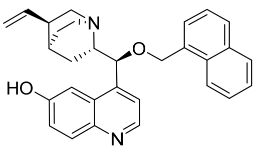

XI

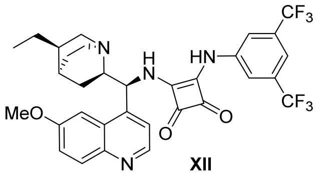

XII

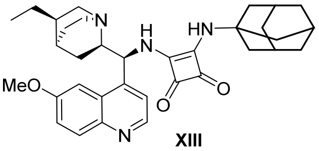

XIII

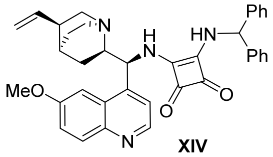

XIV

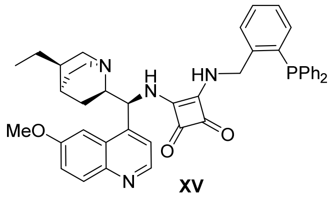

XV

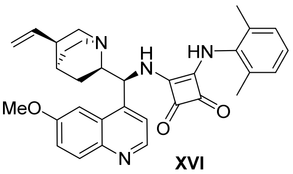

XVI

Table S1. Enantioselective reaction of benzaldehyde 1a with isocyano ester 2a. Catalyst optimization process.[a]

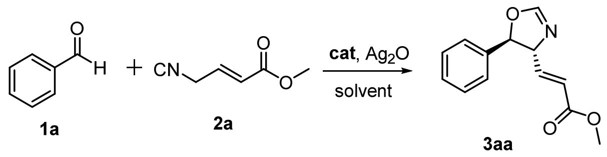

entry	cat	t (min)	yield (%)[b]	ee (%)[c]
1	**I**	40	45	1
2	**II**	20	67	38
3	**III**	40	57	22
4	**IV**	20	59	-29
5	**V**	40	56	-34
6	**VI**	20	68	54
7	**VII**	20	71	78
8	**VIII**	20	62	24
9	**IX**	40	37	2
10	**X**	20	65	6
11	**XI**	20	58	4
12	**XII**	20	66	38
13	**XIII**	50	43	23
14	**XIV**	20	62	50
15	**XV**	20	58	26
17	**XVI**	20	65	49

[a] Reaction conditions: **1a** (0.1 mmol), **2a** (0.11 mmol), **cat** (0.01 mmol), Ag_2O (0.005 mmol), CH_2Cl_2 (1 mL), rt. [b] Yield of isolated product after column chromatography. [c] Determined by chiral HPLC. Negative values indicated the opposite enantiomer.

Table S2. Enantioselective reaction of benzaldehyde 1a with isocyano ester 2a. Effect of solvent and temperature optimization process.[a]

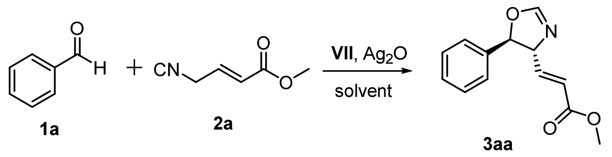

entry	solvent	T (°C)	t (min)	yield (%)[b]	ee (%)[c]
1	DCM	25	20	71	78
2	EtOAc	25	30	72	82
3	Toluene	25	20	70	86
4	THF	25	80	51	84
5	MTBE	25	20	61	85
6	*o*-Xylene	25	20	63	87
7	*m*-Xylene	25	20	60	88
8	*p*-Xylene	25	20	66	87
9	α,α,α-Trifluorotoluene	25	20	70	81
10	MTBE	25	20	61	85
11	Toluene	0	90	67	86
12	MTBE	0	720	59	84
13[d]	Toluene	25	20	70	86
14[d]	DCM	25	20	69	78

[a] Reaction conditions: **1a** (0.1 mmol), **2a** (0.11 mmol), **VII** (0.01 mmol), Ag_2O (0.005 mmol), solvent (1 mL), rt. [b] Yield of isolated product after column chromatography. [c] Determined by chiral HPLC. [d] 3 mL of solvent were used

Table S3. Enantioselective reaction of benzaldehyde 1a with isocyano ester 2a. Effect of the ratio squaramide/Ag_2O.[a]

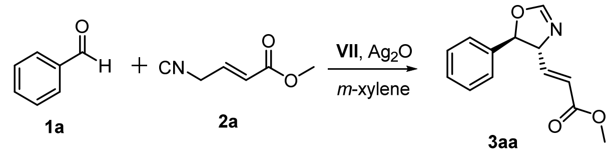

entry	**VII** (mol %)	Ag_2O (mol %)	t (min)	yield (%)[b]	ee (%)[c]
1	10	5	20	70	86
2	5	5	60	52	83
3	5	2.5	360	49	80
4	10	2.5	40	68	90
5	10	1.66	180	64	86
6	10	5	20	70	86
7	5	5	60	52	83

[a] Reaction conditions: **1a** (0.1 mmol), **2a** (0.11 mmol), **VII**, Ag_2O, *m*-xylene (1 mL), rt. [b] Yield of isolated product after column chromatography. [c] Determined by chiral HPLC.

Scheme S1. Proposed synergistic action of silver and bifunctional squaramide in the reaction of vinylogous isocyano esters and aldehydes.

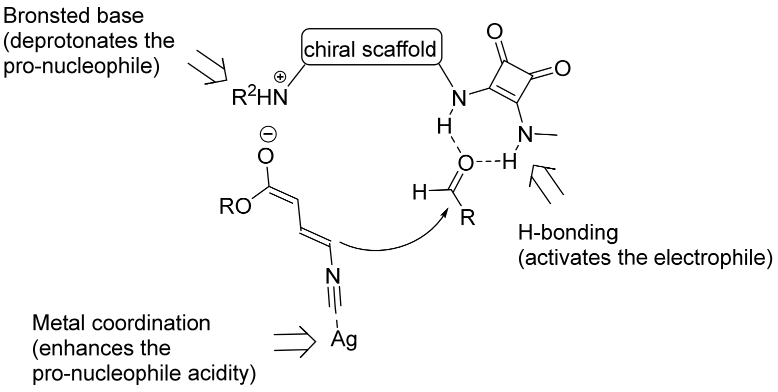

Scheme S2. Unsuccessful lactonization of compound **6**

During revision of the paper, one of the reviewers suggested to study the possible lactonization of compound **6**. Direct lactonization of the hydroxy ester under acidic conditions lead to a complex mixture. On the other hand, upon basic hydrolysis and acid treatment of the resulting acid with TsOH, a γ-lactone **S15** was obtained in 26% yield as a racemic. Spectroscopic data coincided with those reported in the literature.[6] ^{1}H NMR (300 MHz, $CDCl_3$) δ 7.98 (d, *J* = 8.0 Hz, 2H), 7.65 (t, *J* = 8.0 Hz, 1H), 7.52 (t, *J* = 8 Hz, 2H), 5.80 (m, 1H), 2.70-2.54 (m, 3H), 2.49 (m, 1H).

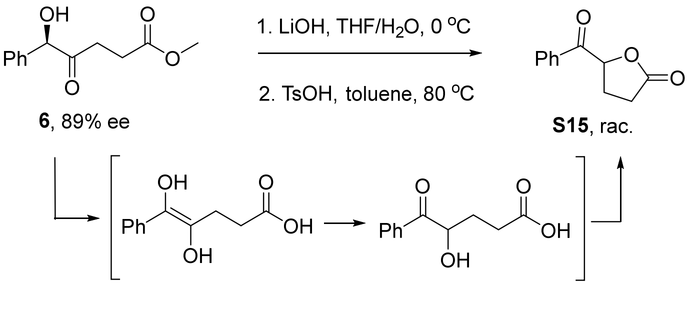

Reaction of acetophenone with compound 2a under the reaction conditions

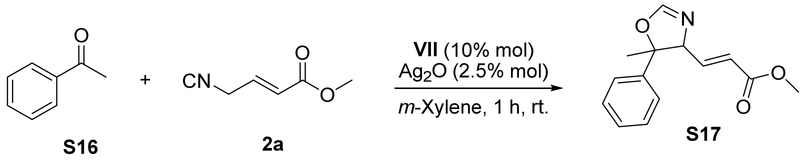

Preliminary studies using the developed conditions in the reaction of acetophenone (**S16**) and compound **2a** have provided the corresponding oxazoline **S17** in 64% yield as 6.2:3.8 diastereomer mixture with 70% ee and 64% ee for each diastereomer, respectively. Further research is underway.

The enantiomeric excess (minor isomer: 58%, major isomer: 70%) was determined by HPLC (Chiralpak IC), hexane:^{i}PrOH 80:20, 1 mL/min, minor diastereomer: minor enantiomer, t_r = 57.1 min, major enantiomer, t_r = 34.4 min; major diastereomer: minor enantiomer, tr = 17.6 min, major enantiomer, t_r = 18.7 min.

Major diastereomer: ^{1}H NMR (300 MHz, $CDCl_3$) δ 7.48-7.26 (5H, m, Ar), 7.06 (1H, br s, CH=N), 7.05 (dd, *J* = 15.0, 6.0 Hz, 1H, CH=), 6.23 (dd, *J* = 15.0, 2.0 Hz, 1H, =CH), 4.68 (1H, dt, *J* = 6.0, 2.0 Hz, 1H, HC-N), 3.78 (s, 3H, OMe), 1.51 (s, 3H, Me). ^{13}C NMR (75 MHz, $CDCl_3$) δ 166.6 (C), 154.8 (CH), 145.0 (C), 143.5 (CH), 129.0 (CH), 128.0 (CH), 124.0 (CH), 123.5 (CH), 88.6 (CH), 75.8 (C), 51.9 (CH_3), 24.1 (CH_3). HRMS (ESI) *m/z* 246.1127 $[M+H]^+$ $C_{14}H_{16}NO_3^+$ requires 246.1125.

Minor diastereomer: ^{1}H NMR (300 MHz, $CDCl_3$) δ 7.45-7.15 (m, 5H, Ar), 7.12 (d, *J* = 1.2 Hz, 1H, CH=N), 6.19 (dd, *J* = 15.6, 6.9 Hz, 1H, CH=), 5.83 (dd, *J* = 15.3, 1.2 Hz, 1H, =CH), 4.56 (dt, *J* = 6.9, 1.8 Hz, 1H, HC-N), 3.59 (s, 3H, OMe), 1.81 (s, 3H, Me).

References

---

[1] Martínez-Pardo, P.; Blay, G.; Escrivá-Palomo, A.; Sanz-Marco, A.; Vila, C.; Pedro, J. R. Catalytic Diastereo- and Enantioselective Synthesis of 2-Imidazolinones. *Org. Lett.* **2019**, *21*, 4063–4066.

[2] Ricardo, M. G.; Marrrero, J. F.; Valdés, O.; Rivera, D. G.; Wessjohann, L. A. A Peptide Backbone Stapling Strategy Enabled by the Multicomponent Incorporation of Amide *N*-Substituents. *Chem. Eur. J.* **2019**, *25*, 769–774.

[3] In some runs, we observed some isomerization of the doble bond to the position continuous to the isocyanide. We did not observe any difference of reactivity in these cases.

[4] a) Kreye, O.; Türünç, O.; Sehlinger, A.; Rackwitz, J.; Meier, M. A. R. Structurally Diverse Polyamides Obtained from Monomers Derived via the Ugi Multicomponent Reaction. *Chem. Eur. J*. **2012**, *18*, 5767-5776; b) Kreye, O.: Zyklisierende und verzweigende mehrfache Ugi-Reaktionen. Martin Luther University, Halle-Wittenberg (MLU), Germany, *Dissertation* **2009**, 122-123.

[5] Blay, G.; Hernández-Olmos, V.; Pedro, J. R. Enantioselective Henry Addition of Methyl 4-Nitrobutyrate to Aldehydes. Chiral Building Blocks for 2-Pyrrolidinones and Other Derivatives *Org. Lett*. **2010**, *12*, 3058–3061.

[6] Abazid, A. H.; Nachtsheim, B. J. A Triazole-Substituted Aryl Iodide with Omnipotent Reactivity in Enantioselective Oxidations. *Angew. Chem. Int. Ed.* **2020**, *59*, 1479-1484
